# Synthesis of Structurally Diverse N‐Substituted Quaternary‐Carbon‐Containing Small Molecules from α,α‐Disubstituted Propargyl Amino Esters

**DOI:** 10.1002/chem.201803143

**Published:** 2018-08-13

**Authors:** Natalia Mateu, Sarah L. Kidd, Leen Kalash, Hannah F. Sore, Andrew Madin, Andreas Bender, David R. Spring

**Affiliations:** ^1^ Department of Chemistry University of Cambridge Lensfield Rd Cambridge CB2 1EW UK; ^2^ AstraZeneca (UK) Ltd. 310 Cambridge Science Park, Milton Rd Cambridge CB4 0FZ UK

**Keywords:** diversity-oriented synthesis, drug discovery, medicinal chemistry, molecular diversity, quaternary stereocenters

## Abstract

N‐containing quaternary stereocenters represent important motifs in medicinal chemistry. However, due to their inherently sterically hindered nature, they remain underrepresented in small molecule screening collections. As such, the development of synthetic routes to generate small molecules that incorporate this particular feature are highly desirable. Herein, we describe the diversity‐oriented synthesis (DOS) of a diverse collection of structurally distinct small molecules featuring this three‐dimensional (3D) motif. The subsequent derivatisation and the stereoselective synthesis exemplified the versatility of this strategy for drug discovery and library enrichment. Chemoinformatic analysis revealed the enhanced sp^3^ character of the target library and demonstrated that it represents an attractive collection of biologically diverse small molecules with high scaffold diversity.

## Introduction

**Figure 1:**
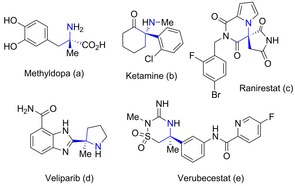
Examples of bioactive compounds containing a N‐substituted quaternary carbon.

N‐containing quaternary stereocenters are important motifs in medicinal chemistry and are present in significant essential medicines including the antihypertensive methyldopa (Aldomet®) and the anaesthetic ketamine (Ketalar®) (Figure 1 a,b).^1^ The presence of this particular stereocenter in the three small molecules currently under clinical evaluation, Ranirestat,^2^ Veliparib^3^ and Verubecestat^4^ (Figure 1 c–e), further highlight its relevance in drug discovery contexts. The biological activity of such compounds has been generally shown to be intrinsically related to their absolute configuration.^5^−^10^ For instance, investigations have concluded that the presence of a N‐containing quaternary (*R*)‐stereocenter within the core structure is necessary for interaction of the substituents with the pockets of the β‐secretase (BACE1) binding site.^9^, ^10^ Moreover, the presence of such restricted elements within a small molecule can provide conformational restrictions owing to their sterically hindered nature, thereby increasing the molecular complexity of a given molecule, which has been shown to be desirable chemical feature for bioactive molecules.^11^, ^12^

Studies have shown that increased complexity (e.g. sp^3^‐rich cycles or quaternary carbons) can correlate to an increase in the selectivity, potency and metabolic stability of drug candidates, along with the successful progression from clinical stages to drug approval.^13^, ^14^ However, due to their congested nature, the incorporation of N‐containing quaternary stereocenters into small molecules poses a synthetic challenge for organic chemists.^15^, ^16^, ^17^, ^18^, ^19^ As a result, small molecules bearing this particular feature are still under‐represented in probe and drug discovery screening collections.

From a synthetic perspective, despite all the advances made in the field of asymmetric catalysis to generate α‐branched amino‐containing compounds,^16^, ^17^, ^18^, ^19^ the majority of the reported reactions are based on simple scaffolds with restricted structural diversity and there has been a limited exploration into the construction of such motifs into more complex molecules. It has, however, been demonstrated that the use of α,α‐disubstituted amino esters as subunits to prepare complex molecules bearing this sterically hindered motif can be particularly effective. Thus, some examples in the literature have validated this strategy by the target‐oriented synthesis (TOS) of biologically active small molecules.^20^ More recently, allyl‐containing cyclic quaternary amino esters have also been used for the diversity‐oriented synthesis (DOS) of small molecule collections.^21^, ^22^ However, structurally diverse, sp^3^‐rich and complex libraries are still under‐represented in probe and drug discovery screening collections.

In addition to these deficiencies, within the literature, significant synthetic challenges have been identified in relation to the development of chemistries which tolerate polar functionalities such as amines, N‐heterocycles and unprotected polar groups.^23^ Moreover, within a fragment‐based drug discovery context, there have been recent calls for the design and synthesis of novel compounds that feature multiple 3D growth vectors whilst incorporating polar functionality for molecular recognition.^24^ In response to this, notable examples of efficient syntheses of diverse sp^3^‐rich libraries have been recently published,^25^, ^26^, ^27^, ^28^, ^29^, ^30^, ^31^, ^32^ however there is still an outstanding need for diverse libraries featuring this key N‐containing quaternary stereocenter motif.

**Figure 2:**
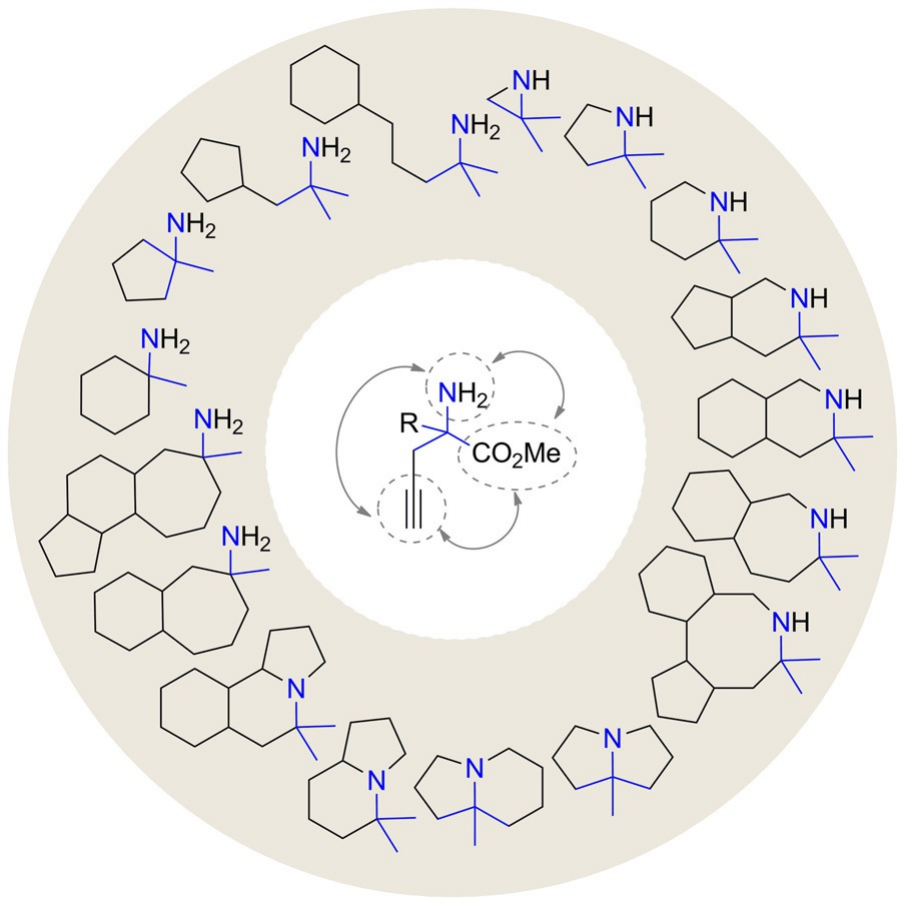
Synthetic versatility of α,α‐disubstituted amino esters for the DOS of N‐substituted quaternary carbon containing small molecules.

With these points in mind, we envisioned that α,α‐ disubstituted propargyl amino esters could act as a versatile and pluripotent platform for the DOS of structurally diverse screening collection featuring a stereogenic N‐containing quaternary carbon (Figure 2). In this manner, the reactivity of its three functional vectors, namely the terminal alkyne, the amine as well and the ester moieties could be exploited in both inter‐ and intramolecular reactions.^33^

## Results and Discussion

### Synthesis of the DOS library

**Scheme 1:**
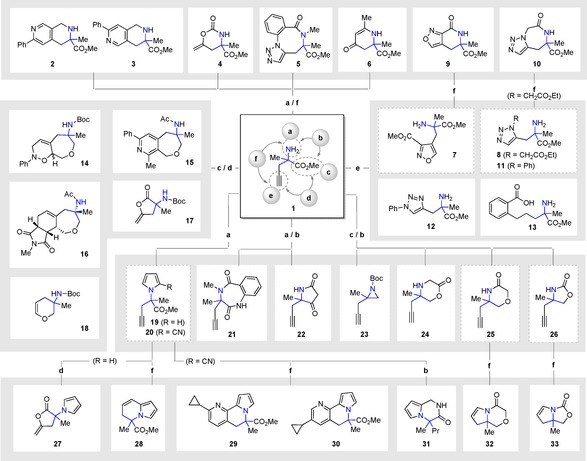
Synthesis of structurally diverse N‐substituted quaternary carbon containing small molecules from α,α‐disubstituted amino ester **1**: a) amine derivatisation; b) pairing of the amino and ester moieties; c) ester derivatisation; d) pairing of the alkyne and ester moieties; e) alkyne derivatisations; f) pairing of the amino and alkyne moieties. For reaction conditions see Supporting Information.

To validate our hypothesis, we initially selected the racemic α‐methyl propargylglycine **1** (R=Me) as a model substrate to perform the reagent‐based DOS outlined in this work (Scheme 1). Our investigations began via alkylation of the amino group in **1** through the introduction of a *tert*‐butyl carbamate, propargyl, 2‐azido benzoyl or an acyl group to generate the first set of highly functionalised intermediates (S2–6, for all intermediates see Scheme S1). Subsequent pairing of the functionalities present in these intermediates with the terminal alkyne could be achieved through different metal‐catalysed cyclisations (Scheme 1, steps a/f). Accordingly, Co‐induced [2+2+2] cyclotrimerisation afforded **2** and **3**, whilst hydroamination using gold catalysis yielded **4** and **6**. Finally, intramolecular Ru‐mediated click chemistry gave rise to compound **5**.

In a similar fashion, Ru‐mediated 1,5‐click chemistry could be used to generate acyclic compounds **7** and **8**, through intermolecular reaction of a protected form of **1** with the commercially available chloro‐oxime or the corresponding azido ester. In turn, lactamisation of the ester moieties within **7** and **8** with the quaternary amine (Scheme 1, steps e/f) afforded the rigidified scaffolds **9** and **10**. Further exploitation of the terminal alkyne handle within **1** via Ru‐ or Cu‐catalysis afforded the acyclic 1,5‐ and 1,4‐phenyl substituted triazoles **11** and **12,** respectively. Finally, Sonogashira cross‐coupling of the terminal alkyne followed by hydrogenation afforded **13** (Scheme 1, step e).

Next, an exploration into intramolecular cyclisations between the ester functionality within **1** and the terminal alkyne was undertaken (Scheme 1, steps c/d). Reduction of the carboxylic moiety into the corresponding alcohol provided the opportunity to introduce additional unsaturated moieties through the alkylation of the newly generated hydroxyl group to afford the allylic‐ and propargylic intermediates. Following this, metal‐ catalysed intramolecular cyclisations such as Co‐induced [2+2+2] cyclotrimerisations, when starting from the dialkyne intermediate or a sequence of ring‐closing eynyne metathesis followed by Diels–Alder cycloaddition, when starting from the allylic intermediate, afforded novel bi‐ and tricyclic oxepine‐containing scaffolds **14**, **15** and **16**. Additionally, ester hydrolysis of **1** to the carboxylic acid followed by the Cu‐promoted intramolecular cyclisation afforded **17**, whilst Ru‐mediated cyclisation of the amino alcohol intermediate with the terminal alkyne generated **18**.

Smaller propargyl‐containing fragments featuring different polar functionalities could also be synthesised via pairing the amino and ester moieties within **1**. Pyrrole **19** was prepared via Paal‐Knorr reaction and a nitrile‐based secondary branch point was introduced to afford **20** (Scheme 1, step a, see Supporting Information). Benzodiazepine **21** was prepared starting from the azido benzoyl intermediate followed by azide reduction to facilitate the lactamisation reaction with the ester functionality (Scheme 1, steps a/b). Again, acylation of **1** proved useful in generating acyclic intermediates which could in turn be cyclized with the ester functionality. For example, Dieckmann condensation of the malonyl derivative followed by decarboxylation, yielded the substituted pyrrolidone **22**. Moreover, reduction of the ester to a hydroxyl also provided the opportunity to cyclise between this moiety and the amino group (Scheme 1, steps c/b). In this manner, using a single *N*‐Boc protected amino alcohol, aziridine **23** and oxazolidinone **26** could be both synthesized. Finally, two further propargyl‐containing scaffolds were formed by N‐acylation followed by hydroxyl‐mediated cyclisation or vice versa, O‐acylation followed by nitrogen‐mediated cyclisation, to yield morpholinones **24** and **25**, respectively.

The reactivity of both **19** and **20** could be further exploited to generate five different scaffolds in a synthetically efficient manner. Unexpectedly, when extending the reaction time for the formation of **19**, an alternative intramolecular cyclisation was observed between the terminal alkyne and the in situ hydrolysed methyl ester, giving rise to **27**. Moreover, gold catalysis promoted the intramolecular cyclisation between the terminal alkyne and a pyrrole within **19** to afford dihydroindolizine **28**. We next sought to explore the reactivity of cyano functionality within **20**. Accordingly, a novel one‐step approach to synthesise tricyclic pyrrole‐containing scaffolds was developed via Co‐catalysed [2+2+2] cyclotrimerization methodology. Thus, starting from **20**, CpCo(CO)_2_ and cyclopropylacetylene could be utilised to furnish fused heterocycles **29** and **30**. Further scaffold exploration using this approach is currently being investigated in our research group. Additionally, Pd‐catalysed reduction of the nitrile moiety within **20** allowed the intramolecular cyclisation affording the propyl‐ substituted pyrrolopiperazine **31**.

Finally, the terminal alkyne within **25** and **26** could be further modified to generate two conformationally restricted small molecules **32** and **33** via vinyl iodide formation followed by Buchwald coupling.^34^

**Figure 3:**
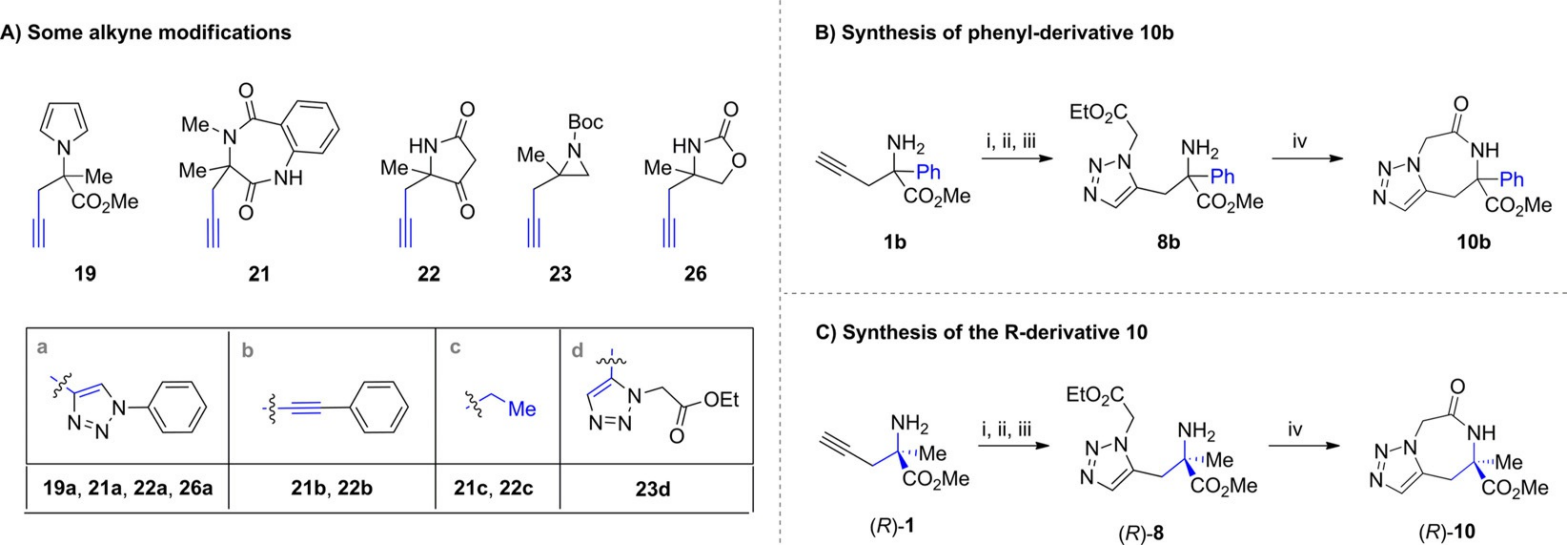
(A) Exemplification of the synthesis of other derivatives via alkyne modifications. Reaction conditions: **19 a**, **21 a, 22 a, 26 a**: azidobenzene, CuSO_4_⋅5 H_2_O, *t*BuOH:H_2_O, rt, 72–92 %; **21 b** and **22 b**: PdCl_2_(PPh_3_)_2_, CuI, Et_3_N, benzyl 2‐iodobenzoate, DMF, rt, 16 h, 60 % and 26 %, respectively; **21 c** and **22 c**: Pd/C, H_2_, MeOH, rt, 2 h, 69 % and 87 %, respectively; **23 d**: ethyl 2‐azidoacetate, Cp*RuCl(COD), PhMe, rt −60 °C, 21 h, 42 % (B) Synthesis of the phenyl‐containing derivative. Reaction conditions: i) Boc_2_O, THF, 70 °C, o/n, 84 %; ii) ethyl 2‐azidoacetate, Cp*RuCl(COD), PhMe, 80 °C, 1 h, 87 %; iii) TFA, CH_2_Cl_2,_ 2 h then NaHCO_3_, (quantitative yield); iv) PhMe, 150 °C, o/n, 76 %. (C) Synthesis of an enantiopure component of the library. Reaction conditions: i) Boc_2_O, THF, 70 °C, o/n, 90 %; ii) ethyl 2‐azidoacetate, Cp*RuCl(COD), PhMe, 50 °C, 1 h, 76 %; iii) TFA, CH_2_Cl_2_, 2 h then NaHCO_3_, 93 %; iv) PhMe, 150 °C, o/n, 79 %.

To demonstrate the synthetic potential of the propargyl‐containing warheads for library enumeration, the alkyne handle within **19**, **21**–**23** and **26** was further modified (Figure 3 A). Thus, Cu‐catalysed click reaction gave the 1,4‐substituted triazoles **19 a**, **21 a**, **22 a**, and **26 a**; Sonogashira cross‐coupling afforded **21 b** and **22 b**; hydrogenation of the alkyne produced saturated derivatives **21 c** and **22 c** and, finally, Ru‐mediated click reaction afforded the 1,5‐ triazole **23 d**.

Using the strategy outlined above we synthesised a structurally diverse library of 40 compounds based around 27 molecular frameworks relevant for drug discovery (see Figure S1) featuring a N‐containing quaternary stereocenter. Each compound was obtained in no more than five synthetic steps (with an average of only three steps) starting from **1**, which demonstrates the efficiency of this process. Importantly, the flexibility of this methodology was further exemplified via the synthesis of the phenyl‐derivative **10′** (Figure 3 B) following the same reaction conditions used to generate the methyl derivative **10**. The ability to synthesise the asymmetric version of any compound from the library was also validated through the synthesis of the optically pure triazolodiazepine (*R*)‐**10** (Figure 3 C) from (*R*)‐**1** (see Supporting Information).

### Chemoinformatic assessment

**Figure 4:**
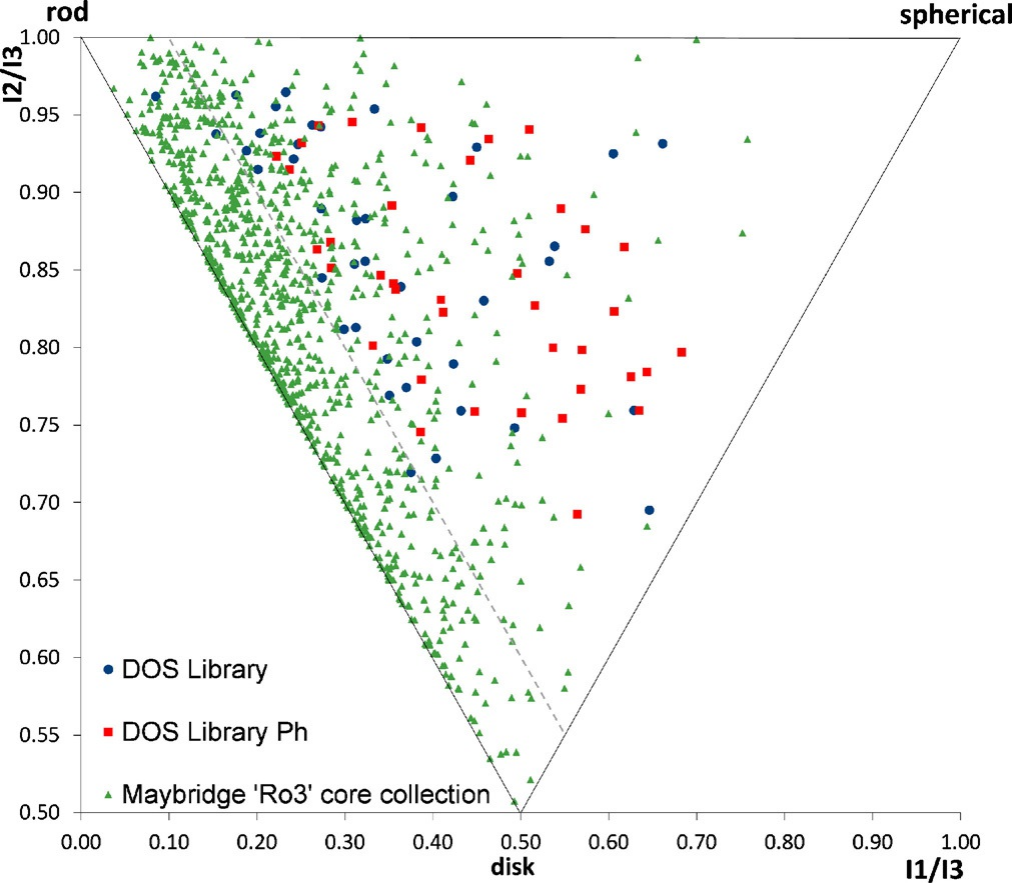
Comparative PMI plot analysis of DOS library (blue circles), Maybridge core 1000‐member “Ro3” Fragment library (green triangles) and the DOS Ph virtual library (red squares). Compounds within “flatland” (represented by npr1+npr2 value <1.1, dashed line) could also be identified; the further from this area the molecules move the more they extend into three‐dimensional space.^36^.

In order to assess the molecular shape distribution of the resulting library, a principal moments of inertia (PMI) analysis was conducted (Figure 4).^35^ Pleasingly, analysis of the DOS library showed a broad distribution of molecular shape space, with 93 % of the library out of “flatland”^36^ and possessing considerable 3D character. In order to investigate the influence of the substituent at the quaternary centre on the shape, a virtual collection based on the phenyl‐derivatives (DOS Library Ph, see Supporting Information) was plotted in the same graph (Figure 4). Comparative analysis between the two DOS libraries highlighted a shift to the right‐hand side of the plot and more 3D character when modifying the quaternary substituent. These results suggest that installation and substituent modification of the N‐containing quaternary sp^3^ stereocenter represents a useful strategy for “tuning” the 3D molecular shape (and thus target binding profile) of a given molecule. Finally, the commercially available Maybridge “Rule of three” (Ro3) core fragment library comprising 1000 screening compounds (see Supporting Information and Figure 4) was analysed and compared to the DOS library. Importantly, only 28 % of the Maybridge library appeared out of “flatland” with a high proportion of “disk” and “rod” type compounds, which highlights the increased diversity and more 3D character displayed by the DOS library. Interestingly, only two compounds within the Maybridge collection featured a N‐containing quaternary carbon, exemplifying the limited inclusion of this moiety within traditional screening collections.

In addition to PMI analysis, calculation of the mean values for physicochemical properties related to the “rule of three” guidelines^37^ commonly adopted within fragment library generation (Table 1) was conducted. Notably the DOS library featured a low mean SlogP (1.37), higher mean number of chiral centres (1.1) and low fraction of aromatic atoms per molecule (0.29), compared to commercial libraries (see Supporting Information), demonstrating the amenability of the DOS library to fragment‐based drug discovery approaches.^38^

**Table 1:** Mean physicochemical properties of fragment collections.

Property^[a]^	Ideal range^[b]^	This work	Maybridge “Ro3” core collection	Chembridge
SlogP	0–3	1.37	1.92	1.31
*M*_W_	<300	237	182	222
HBA	<3	2.63	1.83	1.81
HBD	<3	0.78	1.01	1.04
chiral centres	–	1.10	0.14	0.27
fraction Ar	–	0.29	0.52	0.42

[a] *M*_W_=molecular weight, HBA=number of hydrogen bond acceptors, HBD=number of hydrogen bond donors. [b] Ideal range based on guidelines of Fragment “rule of three”.^37^ Green=inside ideal range. Yellow=extreme of ideal range.

**Figure 5:**
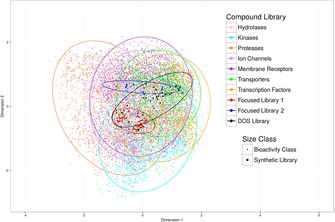
Bioactive space coverage of the DOS library, compared to two targeted compound libraries and seven different bioactivity classes of common protein targets obtained from ChEMBL (based on Morgan fingerprints and Multi‐Dimensional Scaling, MDS; ellipses cover 90 % of the data in each class. It can be seen that the DOS library displays significantly wider coverage in bioactivity space than the two targeted libraries, covering all common classes of drug targets used for comparison.

Whilst PMIs are a common way of assessing shape diversity, the relationship to bioactivity coverage appears to be more complex.^39^ To assess the coverage of our DOS library in bioactivity space, we compared it to two previously reported focused synthetic libraries,^40^, ^41^ as well as bioactive compounds retrieved from ChEMBL, a database of binding and functional data for a large number of drug‐like bioactive compounds. The molecular weight range of the bioactive compounds selected for the analysis was between 200 and 700, and the compounds covered different target classes.^42^ Then, their Morgan fingerprints^43^ were generated for multi‐dimensional scaling (MDS). Our DOS library (Figure 5, shown in black) covers a wider area of biologically relevant chemical space compared to the targeted libraries, overlapping with areas occupied by kinase inhibitors, ion channels blockers, membrane receptors ligands and ligands of other protein classes.

Currently, the DOS library is being screened via high‐throughput X‐ray crystallography methods in collaboration with the XChem fragment screening facility at the Diamond Light Source synchrotron. Hits against three different target classes including hydrolases, the TGF‐beta family of growth factors, and peptidases have been successfully identified. Further biological results from these initial campaigns will be published in due course.

## Conclusion

In summary, we have developed a new, efficient reagent‐based DOS approach using α‐methyl‐α‐propargyl amino esters to generate a library of 40 structurally diverse small molecules. Importantly, throughout this sp^3^‐rich library we have installed a N‐containing quaternary stereocenter, polar functionalities and N‐based heterocycles, answering key calls from within the field. The resultant library also adheres to the fragment rule of three guidelines, highlighting the amenability to this method of screening. Furthermore, we have demonstrated the scope of our library through modifications to the alkyne‐containing scaffolds, the synthesis of a phenyl‐containing derivative and the asymmetric version of an analogue, all of which can be applied to library enumeration. Finally, cheminformatic analysis has shown a broad molecular shape distribution, the importance of the quaternary sp^3^ carbon in modifying the molecular shape and the increased diversity and 3D character of the library when compared to a commercial library. The analysis of the resulting bioactivity space coverage showcases that the target library represents an attractive screening collection of biologically diverse small molecules.

## Experimental Section

### General remarks

All reagents and solvents were purchased from commercial sources and used without further purification unless otherwise stated. All the experiments were carried out under a nitrogen atmosphere unless otherwise stated. Melting points were measured using a Büchi B545 melting point apparatus and are uncorrected. Thin layer chromatography (TLC) was performed on precoated Merck silica gel GF_254_ plates. Flash column chromatography was performed on silica gel (230–400 mesh). ^1^H NMR and ^13^C NMR were recorded on a Bruker Avance 500 MHz instrument in CDCl_3_ and MeOD. HRMS was recorded on a Micromass Q‐TOF mass spectrometer or a Waters LCT Premier Time of Flight mass spectrometer.

**Methyl 3‐methyl‐7‐phenyl‐1,2,3,4‐tetrahydro‐2,6‐naphthyridine‐3‐carboxylate (2) and methyl 3‐methyl‐6‐phenyl‐1,2,3,4‐tetrahydro‐2,7‐naphthyridine‐3‐carboxylate (3)**: A solution of methyl 2‐((*tert*‐butoxycarbonyl)amino)‐2‐methylpent‐4‐ynoate (S2) (150 mg, 0.62 mmol) in DMF (3.0 mL) was added to a suspension of sodium hydride (60 % in mineral oil, 30 mg, 0.74 mmol) in DMF(3.2 mL) cooled to 0 °C. After 10 minutes stirring, propargyl bromide (80 wt.% in toluene, 0.138 minutes stirring, propargyl bromide (80 wt.% in toluene, 0.138 mL, 1.24 mmol) was added and the mixture was warmed to room temperature and stirred for 4 hours. Then, the mixture was diluted with saturated aqueous solution of NH_4_Cl and the aqueous layer was extracted with EtOAc (3×). The combined organic layers were washed with brine, dried with Na_2_SO_4_, filtered, and the solvents were evaporated. The crude product was purified by flash column chromatography (silica gel petroleum ether/EtOAc, 5:1) to yield 168 mg of methyl 2‐((*tert*‐butoxycarbonyl)(prop‐2‐yn‐1‐yl)amino)‐2‐methylpent‐4‐ynoate (**S3**) (97 % yield) as a colourless oil. *R*_f_=0.40 (petroleum ether/EtOAc; 5:1); ^1^H NMR (400 MHz, CDCl_3_): *δ*=1.43 (s, 9 H), 1.70 (s, 3 H), 1.99 (t, *J=*2.6 Hz, 1 H), 2.20 (t, *J=*2.4 Hz, 1 H), 2.73 (dd, *J=*17.1, 2.5 Hz, 1 H), 3.12 (d, *J=*16.6 Hz, 1 H), 3.69 (s, 3 H), 4.02 (d, *J=*18.5 Hz, 1 H), 4.26 ppm (br s, 1 H); ^13^C NMR (101 MHz, CDCl_3_): *δ*=22.0, 27.0, 28.3, 34.1, 52.4, 62.5, 70.9, 71.1, 80.1, 80.9, 81.7, 154.3, 174.0 ppm; HRMS (ESI): *m*/*z* calcd for C_15_H_22_NO_4_ [*M*+H]^+^: 280.1543; found: 280.1534.

CpCo(CO)_2_ (6.4 mg, 0.036 mmol) and benzonitrile (0.05 mL, 0.48 mmol) were added to a solution of **S3** (68 mg, 0.24 mmol) in toluene (2.4 mL), previously degassed with argon for 15 minutes, and the mixture was heated at 110 °C for 36 hours. Then, the solvent was removed under reduced pressure and the crude product was purified by column chromatography (silica gel; petroleum ether/EtOAc, gradient from 5:1 to 3:2). The resulting inseparable mixture of regioisomers (ca. 40:60 ratio as determined by ^1^H NMR) was dissolved in CH_2_Cl_2_ (1 mL) and TFA (0.5 mL) was then added to the solution. After 1 hour stirring the solvent was removed under reduced pressure. The crude product was dissolved in EtOAc (5 mL) and a saturated aqueous solution of NaHCO_3_ (5 mL) was added and the mixture was stirred for 10 minutes. Then, the organic layer was separated, dried over Na_2_SO_4_, filtered and concentrated in vacuo. The crude product was purified by flash column chromatography (silica gel; gradient from EtOAc:MeOH 1:0 to 9:1) to afford 20 mg of **2** (30 % yield) and 18 mg of **3** (26 % yield) both as colourless oils.

*Data of minor regioisomer* (**2**): *R*_f_=0.21 (EtOAc): ^1^H NMR (400 MHz, CDCl_3_): *δ*=1.48 (s, 3 H), 2.37 (br s, 1 H), 2.83 (d, *J=*16.7 Hz, 1 H), 3.35 (d, *J=*16.7 Hz, 1 H), 3.69 (s, 3 H), 4.05–4.18 (m, 2 H), 7.39 (ddd, *J=*7.3, 3.7, 1.3 Hz, 1 H), 7.41–7.49 (m, 3 H), 7.90–7.98 (m, 2 H), 8.37 ppm (s, 1 H); ^13^C NMR (101 MHz, CDCl_3_): *δ*=26.6, 37.3, 42.6, 52.5, 57.9, 120.6, 126.9, 128.3, 128.8, 128.8, 139.4, 143.2, 147.8, 155.3, 175.9 ppm; HRMS (ESI): *m*/*z* calcd for C_17_H_19_N_2_O_2_ [*M*+H]^+^: 283.1441; found: 283.1431

*Data of major regioisomer* (**3**): *R*_f_=0.32 (EtOAc): ^1^H NMR (400 MHz, CDCl_3_): *δ*=1.48 (s, 3 H), 2.03 (br s, 1 H), 2.80 (d, *J=*16.2 Hz, 1 H), 3.33 (d, *J=*16.2 Hz, 1 H), 3.68 (s, 3 H), 4.08 (d, *J=*17.0 Hz, 1 H), 4.19 (d, *J=*17.1 Hz, 1 H), 7.34–7.41 (m, 2 H), 7.41–7.48 (m, 2 H), 7.86–7.97 (m, 2 H), 8.43 ppm (s, 1 H); ^13^C NMR (101 MHz, CDCl_3_): *δ*=26.4, 34.7, 44.7, 52.6, 58.1, 117.8, 126.9, 127.8, 128.8, 129.0, 139.4, 143.3, 150.2, 155.1, 175.8 ppm; HRMS (ESI): *m*/*z* calcd for C_17_H_19_N_2_O_2_ [*M*+H]^+^: 283.1441; found: 283.1431.

**Methyl 2‐methyl‐2‐(1*H*‐pyrrol‐1‐yl)pent‐4‐ynoate (19)**: NaOAc (97 mg, 1.19 mmol) was added to a stirred solution of **1** (140 mg, 0.99 mmol) in a mixture 5:3:1 of DCE:H_2_O:AcOH (2.24 mL) and the reaction was stirred at 90 °C. After 5 minutes, 2,5‐dimethoxytetrahydrofuran (0.13 mL, 0.99 mmol) was added and the mixture was stirred at the same temperature overnight. Then, the reaction was cooled to room temperature, diluted with EtOAc and washed with saturated aqueous solution of NaCl. The organic layer was dried over Na_2_SO_4_, filtered and the solvents were concentrated in vacuo. The crude product was purified by flash column chromatography (silica gel; petroleum ether/EtOAc, 9:1) to yield 115 mg of **19** (61 % yield) as a white solid. *R*_f_=0.28 (petroleum ether/EtOAc, 9:1); m.p. 55–57 °C; ^1^H NMR (400 MHz, CDCl_3_): *δ*=1.94 (s, 3 H), 2.05 (t, *J=*2.6 Hz, 1 H), 3.00 (dd, *J=*16.8, 2.6 Hz, 1 H), 3.07 (dd, *J=*16.8, 2.6 Hz, 1 H), 3.74 (s, 3 H), 6.21 (t, *J=*2.2 Hz, 2 H), 6.83 ppm (t, *J=*2.2 Hz, 2 H); ^13^C NMR (101 MHz, CDCl_3_): *δ*=22.8, 30.0, 53.1, 63.1, 72.3, 78.5, 108.8, 118.7, 172.0 ppm; HRMS (ESI): *m*/*z* calcd for C_11_H_14_NO_2_ [*M*+H]^+^: 192.1019; found: 192.1013.

**Methyl 2‐(2‐cyano‐1*H*‐pyrrol‐1‐yl)‐2‐methylpent‐4‐ynoate (20)**: Chlorosulfonyl isocyanate (0.047 mL, 0.55 mmol) was added drop‐wise to a solution of **19** (70 mg, 0.37 mmol) in MeCN (3.6 mL) at 0 °C. After 1 hour stirring, DMF (0.14 mL, 1.85 mmol) was added and the mixture was stirred for 2 hours. Then, saturated aqueous solution of NaHCO_3_ was added and the reaction mixture was extracted with EtOAc (3x). The combined organic layers were dried over Na_2_SO_4_, filtered and the solvents were concentrated in vacuo. The crude product was purified by flash column chromatography (silica gel; petroleum ether/EtOAc, 4:1) to yield 57 mg of **20** (71 % yield) as colourless oil. *R*_f_=0.29 (petroleum ether/EtOAc; 5:1); mp 64–66 °C; ^1^H NMR (400 MHz, CDCl_3_): *δ*=1.88–2.08 (m, 4 H), 3.09 (dd, *J=*17.5, 2.7 Hz, 1 H), 3.31 (dd, *J=*17.5, 2.1 Hz, 1 H), 3.84 (s, 3 H), 6.19 (dd, *J=*3.8, 3.0 Hz, 1 H), 6.93 (dd, *J=*3.9, 1.5 Hz, 1 H), 7.07 ppm (dd, *J=*2.8, 1.6 Hz, 1 H); ^13^C NMR (101 MHz, CDCl_3_): *δ*=23.8, 29.5, 53.7, 64.5, 72.8, 77.2, 102.9, 108.5, 114.1, 123.2, 125.5, 171.5 ppm; HRMS (ESI): *m*/*z* calcd for C_12_H_12_N_2_NaO_2_ [*M*+Na]^+^: 239.0791; found: 239.0783.

**3‐Methyl‐5‐methylene‐3‐(1*H*‐pyrrol‐1‐yl)dihydrofuran‐2(3*H*)‐one (27)**: NaOAc (26 mg, 0.32 mmol) was added to a stirred solution of **19** (30 mg, 0.16 mmol) in a mixture 5:3: of DCE:H_2_O:AcOH (1.6 mL) and the reaction was stirred at 90 °C for 24 hours. Then, the reaction was cooled to room temperature, diluted with EtOAc and washed with saturated aqueous solution of NaCl. The organic layer was dried over Na_2_SO_4_, filtered and the solvents were concentrated in vacuo. The crude product was purified by flash column chromatography to yield 12 mg of **27** (42 % yield) as a colourless oil. *R*_f_=0.22 (petroleum ether/EtOAc, 9:1); ^1^H NMR (400 MHz, CDCl_3_): *δ*=1.85 (s, 3 H), 3.11 (dt, *J=*16.2, 1.5 Hz, 1 H), 3.39 (dt, *J=*16.2, 1.8 Hz, 1 H), 4.51 (dt, *J=*3.2, 1.7 Hz, 1 H), 4.91 (dt, *J=*2.8, 2.1 Hz, 1 H), 6.24 (t, *J=*2.2 Hz, 2 H), 6.86 ppm (t, *J=*2.1 Hz, 2 H); ^13^C NMR (101 MHz, CDCl_3_): *δ*=24.2, 41.5, 61.6, 91.2, 109.7, 118.4, 151.3, 173.5 ppm; HRMS (ESI): *m*/*z* calcd for C_10_H_12_NO_2_ [*M*+H]^+^: 178.0861; found: 178.0863.

**Methyl 5‐methyl‐5,6‐dihydroindolizine‐5‐carboxylate (28)**: A solution of AuSPhos(MeCN)SbF_6_ (4.8 mg, 5 mol %) in DCE (0.1 mL) was added drop‐wise to a solution of **19** (21 mg, 0.11 mmol) and ethanol (0.032 mL, 0.55 mmol) in DCE (1.0 mL). The resulting solution was heated for 2 hours at 40 °C and then the solvent was removed under reduced pressure. The crude product was purified by flash column chromatography (silica gel; petroleum ether/EtOAc, 4:1) to yield 17 mg of **28** (81 % yield) as a colourless oil. *R*_f_=0.24 (petroleum ether/EtOAc, 5:1); ^1^H NMR (400 MHz, CDCl_3_): *δ*=1.80 (s, 3 H), 2.49 (dt, *J=*16.9, 2.7 Hz, 1 H), 2.95 (dd, *J=*16.9, 6.1 Hz, 1 H), 3.64 (s, 3 H), 5.58–5.69 (m, 1 H), 6.12 (d, *J=*2.5 Hz, 1 H), 6.22 (t, *J=*3.2 Hz, 1 H), 6.46 (dd, *J=*9.7, 2.8 Hz, 1 H), 6.85 ppm (s, 1 H); ^13^C NMR (101 MHz, CDCl_3_): *δ*=24.0, 35.8, 52.9, 61.3, 107.4, 108.9, 117.1, 118.9, 120.8, 130.3, 174.0 ppm; HRMS (ESI): *m*/*z* calcd for C_11_H_14_NO_2_ [*M*+H]^+^: 192.1019; found: 192.1012.

**Methyl 2‐cyclopropyl‐6‐methyl‐5,6‐dihydropyrrolo[1,2‐*h*][1,7]naphthyridine‐6‐carboxylate (29) and methyl 3‐cyclopropyl‐6‐methyl‐5,6‐dihydropyrrolo[1,2‐*h*][1,7]naphthyridine‐6‐carboxylate (30)**: CpCo(CO)_2_ (13.3 mg, 0.038 mmol) and cyclopropylacetylene (0.048 mL, 0.57 mmol) were added to a solution of **20** (40 mg, 0.19 mmol) in toluene (3.5 mL), previously degassed with argon for 15 minutes, and the mixture was heated at 110 °C for 36 hours. Then, the solvent was removed under reduced pressure and the crude product was purified by column chromatography (silica gel; gradient from petroleum/EtOAc, gradient from 4:1 to 2:1) to afford 14 mg of 29 (27 % yield) and 5 mg of 30 (10 % yield) both as white solids.

*Data of major regioisomer* (**29**): *R*_f_=0.34 (petroleum ether/EtOAc; 4:1); mp 108–110 °C; ^1^H NMR (400 MHz, CDCl_3_): *δ*=0.94 (dd, *J=*8.2, 3.3 Hz, 2 H), 0.99–1.12 (m, 2 H), 1.88 (s, 3 H), 1.95–2.07 (m, 1 H), 3.06 (d, *J=*15.4 Hz, 1 H), 3.43 (d, *J=*15.5 Hz, 1 H), 3.54 (s, 3 H), 6.33 (t, *J=*3.2 Hz, 1 H), 6.79 (d, *J=*7.8 Hz, 1 H), 6.90–6.99 (m, 2 H), 7.28 ppm (d, *J=*7.8 Hz, 1 H); ^13^C NMR (101 MHz, CDCl_3_): *δ*=9.7, 17.2, 24.3, 39.4, 53.0, 61.2, 107.9, 110.1, 117.8, 120.0, 120.2, 131.5, 135.1, 146.9, 161.7, 173.1 ppm; HRMS (ESI): *m*/*z* calcd for C_17_H_19_N_2_O_2_ [*M*+H]^+^: 283.1441; found: 283.1435.

*Data of minor regioisomer* (**30**): *R*_f_=0.23 (petroleum ether/EtOAc; 2:1); ^1^H NMR (400 MHz, CDCl_3_): *δ*=0.67–0.75 (m, 2 H), 0.97–1.03 (m, 2 H), 1.82–1.92 (m, 4 H), 3.08 (d, *J=*15.5 Hz, 1 H), 3.43 (d, *J=*15.5 Hz, 1 H), 3.54 (s, 3 H), 6.35 (t, *J=*3.0 Hz, 1 H), 6.94 (s, 2 H), 7.07 (s, 1 H), 8.25 ppm (s, 1 H); ^13^C NMR (101 MHz, CDCl_3_): *δ*=8.9, 9.0, 13.1, 24.3, 39.8, 53.1, 61.0, 107.6, 110.3, 120.0, 123.5, 131.0, 132.2, 136.4, 145.3, 147.2, 173.0 ppm; HRMS (ESI): *m*/*z* calcd for C_17_H_19_N_2_O_2_ [*M*+H]^+^: 283.1441; found: 283.1440.

**4‐Methyl‐4‐propyl‐1,2‐dihydropyrrolo[1,2‐*a*]pyrazin‐3(4*H*)‐one (31)**: PtO_2_ (34 mg, 0.15 mmol) was added to a solution of **20** (35 mg, 0.15 mmol) in MeOH (3 mL) and the reaction mixture was stirred under H_2_ atmosphere (1 atm) for 16 h. Then, the resulting suspension was filtered and the filtrate was concentrated under reduced pressure. The crude product was purified by flash column chromatography (silica gel; petroleum ether/EtOAc, 1:1) to yield 10 mg of **31** (35 % yield) as colourless oil. *R*_f_=0.24 (petroleum ether/EtOAc; 1:1); ^1^H NMR (400 MHz, CDCl_3_): *δ*=0.82 (t, *J=*7.2 Hz, 3 H), 0.88–1.01 (m, 1 H), 1.11–1.23 (m, 1 H), 1.72 (d, *J=*4.5 Hz, 3 H), 1.82 (ddd, *J=*13.6, 10.3, 4.3 Hz, 1 H), 2.16 (ddd, *J=*13.7, 12.1, 4.6 Hz, 1 H), 4.52–4.63 (m, 2 H), 5.93 (d, *J=*2.0 Hz, 1 H), 6.25 (t, *J=*3.2 Hz, 1 H), 6.35 (s, 1 H), 6.69 ppm (dd, *J=*2.4, 1.8 Hz, 1 H); ^13^C NMR (126 MHz, CDCl_3_): *δ*=14.0, 17.4, 26.4, 39.8, 43.7, 62.9, 102.8, 109.8, 115.9, 122.1, 172.2 ppm; HRMS (ESI): *m*/*z* calcd for C_11_H_17_N_2_O [*M*+H]^+^: 193.1335; found: 193.1328.

## Conflict of interest

The authors declare no conflict of interest.

## Supporting information

As a service to our authors and readers, this journal provides supporting information supplied by the authors. Such materials are peer reviewed and may be re‐organized for online delivery, but are not copy‐edited or typeset. Technical support issues arising from supporting information (other than missing files) should be addressed to the authors.

Supplementary
