# Supplementary material for: Synthesis of Structurally Diverse N‐Substituted Quaternary‐Carbon‐Containing Small Molecules from α,α‐Disubstituted Propargyl Amino Esters
Source: Chemistry. 2018 Aug 13;24(51):13681–7. doi: 10.1002/chem.201803143 (PMC6220872; doi:10.1002/chem.201803143)
Supplement: Supplementary file 1 — Supplementary [file CHEM-24-13681-s001.pdf]

CHEMISTRY
A European Journal

Supporting Information

Synthesis of Structurally Diverse N-Substituted Quaternary-Carbon-Containing Small Molecules from α,α-Disubstituted Propargyl Amino Esters

Natalia Mateu+,*[a] Sarah L. Kidd+,[a] Leen Kalash,[a] Hannah F. Sore,[a] Andrew Madin,[b] Andreas Bender,[a] and David R. Spring*[a]

chem_201803143_sm_miscellaneous_information.pdf

Synthesis of structurally diverse *N*-substituted quaternary carbon containing small molecules from α,α-disubstituted propargyl amino esters

Natalia Mateu‡, Sarah L. Kidd‡, Leen Kalash, Hannah Sore, Andrew Madin, Andreas Bender and David R. Spring**

Department of Chemistry, University of Cambridge

Lensfield Road, Cambridge, CB2 1EW, UK

*E-mail: spring@ch.cam.ac.uk

*E-mail: nm462@cam.ac.uk

SUPPORTING INFORMATION

TABLE OF CONTENTS

SUPPLIMENTARY FIGURES 2

GENERAL REMARKS 6

PROCEDURES AND ANALYTICAL DATA 8

STEREOCHEMICAL AND STRUCTURAL ASSIGNMENTS 60

Compounds 2 and 3 60

Compound 14 61

Compound 16 61

COMPUTATIONAL ANALYSIS 63

A) Principal Moment of Inertia (PMI) 63

General details 63

B) Computational evaluation of physicochemical properties 76

General details 76

C) Multi-Dimensional Scaling (MDS) Plot Generation 78

Selection of ChEMBL 20 compounds 78

Multi-Dimensional Scaling (MDS) analysis 78

NMR SPECTRA 79

SUPPLIMENTARY FIGURES

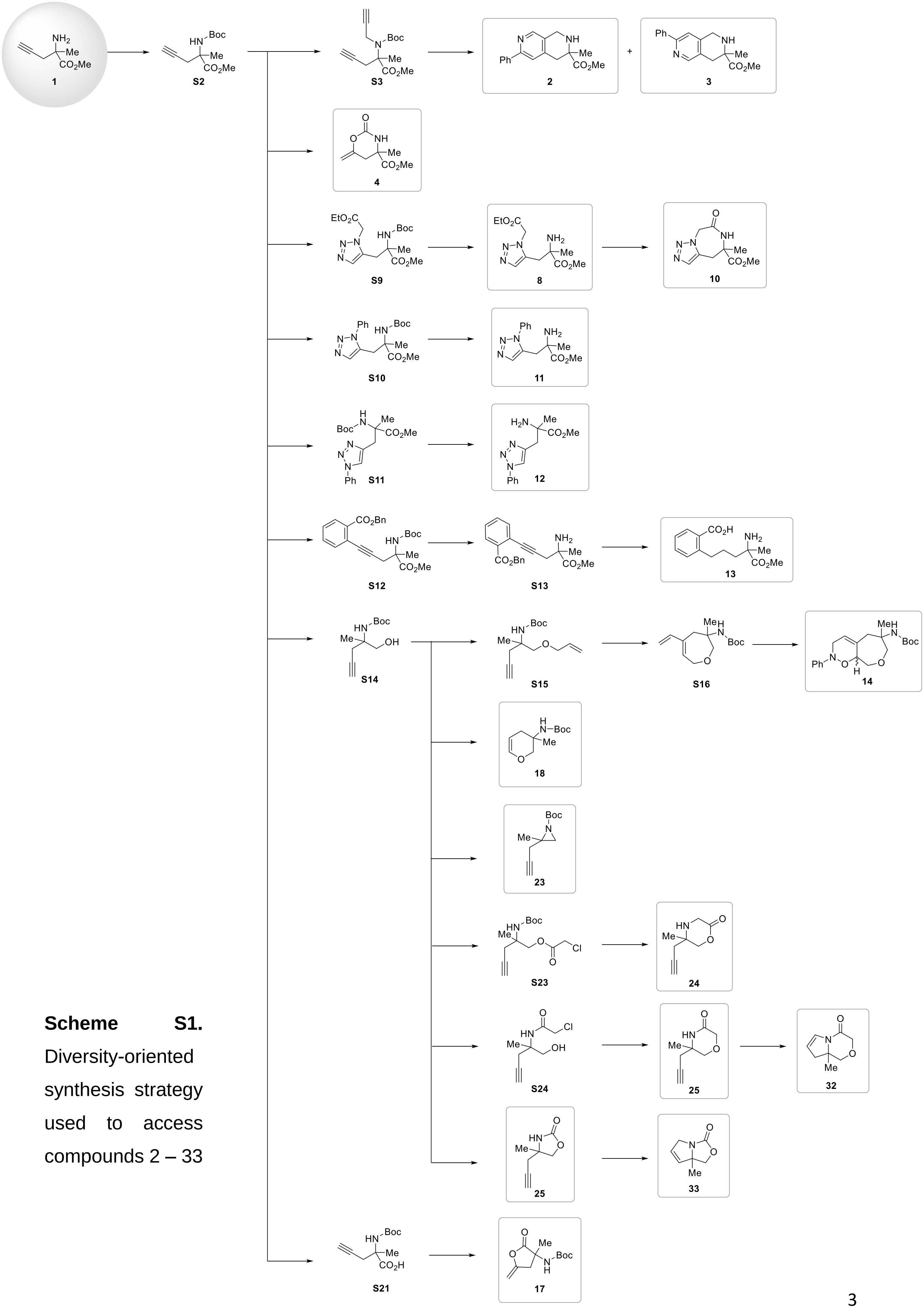

Scheme S1. Diversity-oriented synthesis strategy used to access compounds 2 – 33

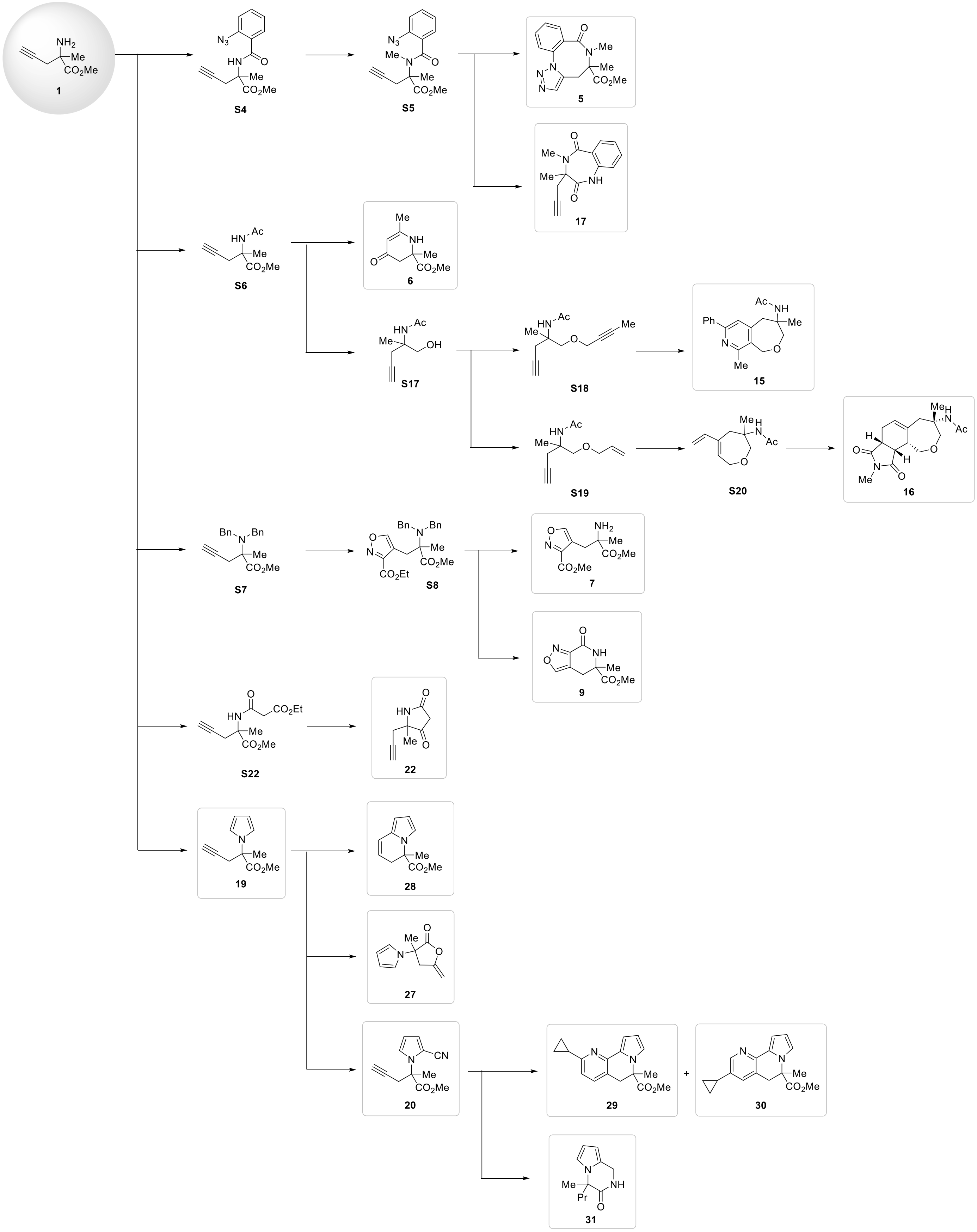

Scheme S1. Diversity-oriented synthesis strategy used to access compounds 2 – 33 (cont.)

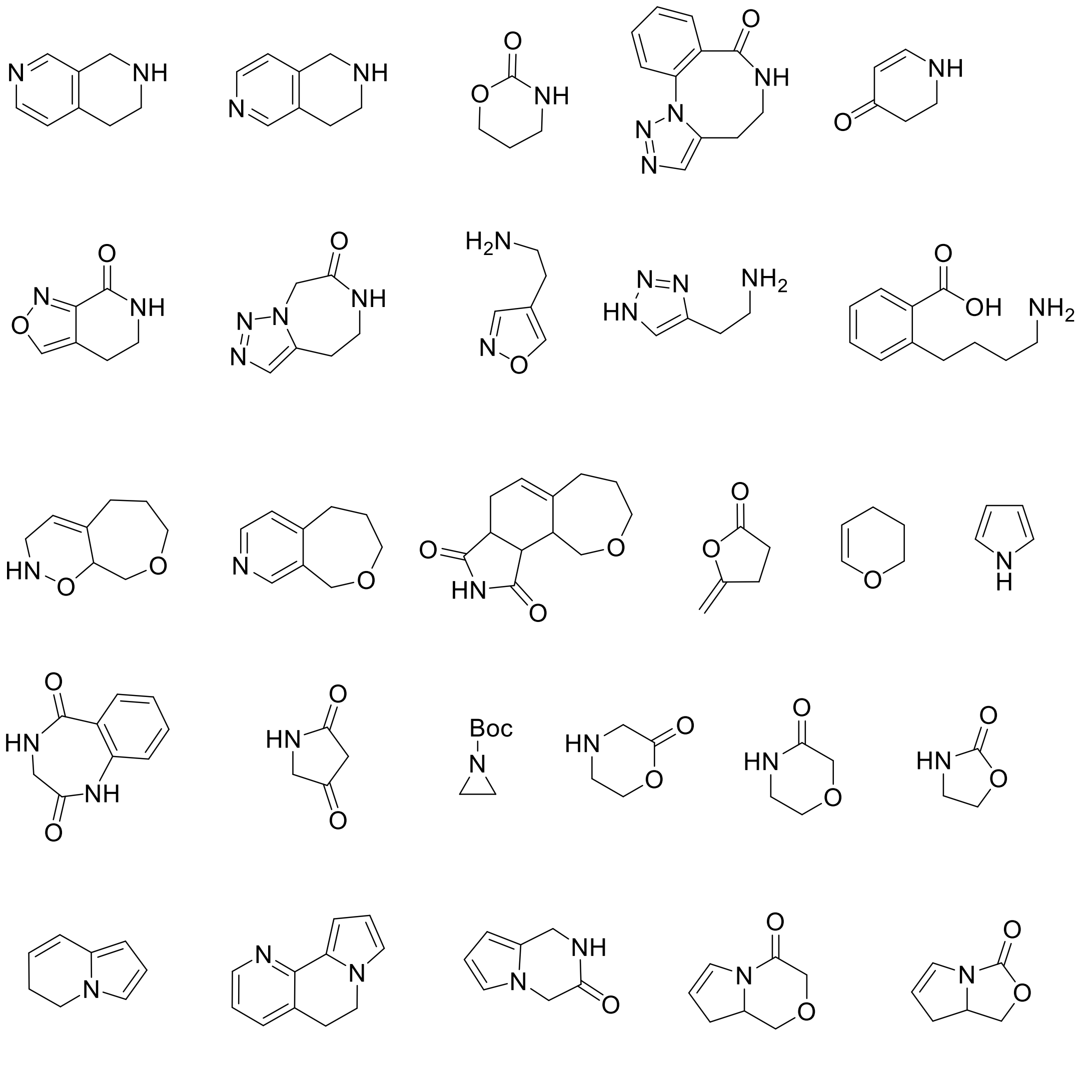

Figure S1. The 27 molecular frameworks featured within DOS library**.**

GENERAL REMARKS

All non-aqueous reactions were performed in dry glassware under a stream of Argon using anhydrous solvents. Tetrahydrofuran was dried over sodium wire and distilled from a mixture of lithium aluminium hydride and calcium hydride with triphenylmethane as the indicator. Dichloromethane, toluene and methanol were all distilled from calcium hydride. Petroleum ether was distilled before use and refers to the fraction between 40-60 ºC; anhydrous DMF and DCE were purchased from Aldrich and used as received. All reagents were obtained from commercial suppliers and used without further purification. Reactions were carried out at ambient temperature unless otherwise stated. All temperatures below 0 ºC are achieved with an external bath: those of 0 ºC were maintained using an ice/water bath, those of lower temperatures using a dry ice/ DMF bath.

Yields refer to chromatographically and spectroscopically pure compounds unless otherwise stated. Analytical thin layer chromatography (TLC) was performed on commercially available glass pre-coated Merck Kiesel gel 60 F254 plates. Visualisation was by quenching of UV fluorescence (λ max = 254 nm) or by staining with potassium permanganate. R_f values are quoted to the nearest 0.01. Where possible, reactions were monitored using TLC. Flash column chromatography was performed using slurry-packed SiO_2 (Merck Grade 9385, 230-400 mesh) under a positive pressure of N_2. Melting points were obtained on a Buchi B-545 melting point apparatus and are uncorrected. Optical rotations were measured on an Anton Paar MCP 100 Modular Compact Polarimeter. Proton nuclear magnetic resonance spectra (^{1}H NMR) were recorded using an internal deuterium lock at ambient probe temperatures (unless otherwise stated) on the following instruments: Bruker DPX-400 (400 MHz), Bruker Avance 400 QNP (400 MHz), Bruker BB 500 (500 MHz) and Bruker Avance 500 Cryo Ultrashield (500 MHz). Chemical shifts (δH) are referenced to the residual non-deuterated solvent peak and quoted in parts per million (ppm) to the nearest 0.01 ppm. Coupling constants are quoted in Hertz to the nearest 0.1 Hz. Data are reported in the format: chemical shift, integration, multiplicity [app = apparent; br = broad; s =s singlet; d = doublet; t

= triplet; q = quartet; quin = quintet; m = multiplet; or as a combination of these, e.g. dd], coupling constant(s), assignment. Proton assignments are determined either on the basis of unambiguous chemical shift, coupling patterns, by patterns observed in the two-dimensional experiments (1H-1H COSY, HMBC and HMQC) or by analogy to fully interpreted spectra for related compounds. Carbon nuclear magnetic resonance spectra (^{13}C NMR) were recorded by broadband proton spin decoupling at ambient probe temperatures (unless otherwise stated) using an internal deuterium lock on the following instruments: Bruker DPX-400 (100 MHz), Bruker Avance 400 QNP (100 MHz), Bruker BB 500 (125 MHz) and Bruker Avance 500 Cryo Ultrashield (125 MHz). Chemical shifts (δC) are referenced to the residual non-deuterated solvent peak and quoted in parts per million (ppm) to the nearest 0.1 ppm. Assignments are supported by either chemical shift, APT/DEPT, two dimensional experiments (HMBC and HMQC) or by analogy to fully interpreted spectra for related compounds. High resolution mass spectrometry (HRMS), measurements were carried out on a Micromass LCT Premier spectrometer using electron spray ionisation (ESI) techniques. Masses are quoted within the error limits of ±5ppm mass units.

PROCEDURES AND ANALYTICAL DATA

Synthesis of methyl 2-amino-2-methylpent-4-ynoate (1)

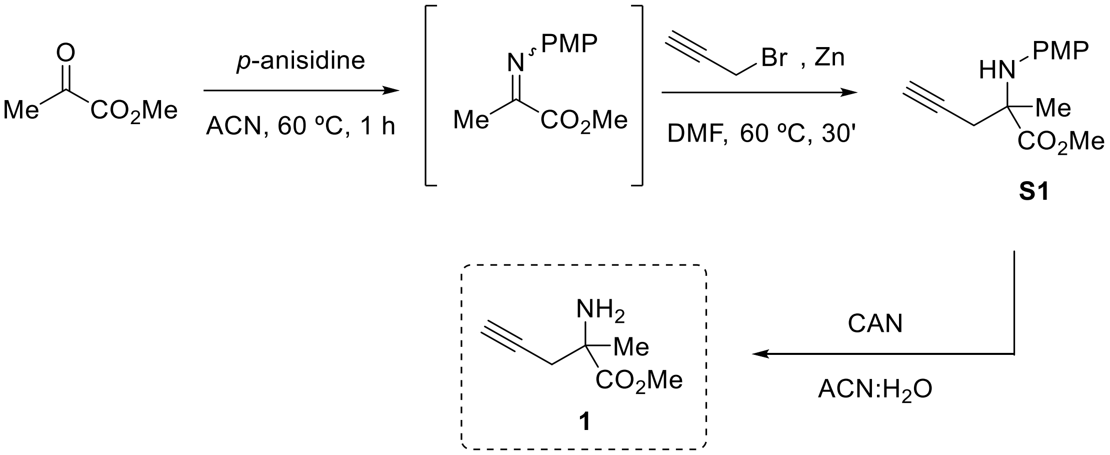

Following a modified version of a reported procedure,[1] methyl pyruvate (4.85 mL, 53.6 mmol) and *p*-anisidine (4.40 g, 35.7 mmol) were dissolved in MeCN (35 mL) and the mixture was heated at 50 ºC for 1 hour. Then, the solvent was removed under reduced pressure and the crude product was dissolved in a mixture of petroleum ether/CH_2Cl_2 (10:1). The grey precipitate formed was removed by filtration and the filtrate was concentrated *in vacuo*. The resulting green oil was dissolved in DMF (158 mL) and the mixture was cooled to 0 ºC. Propargyl bromide (80% wt. % in toluene, 4.77 mL, 42.9 mmol) and activated zinc powder (3.5 g, 53.6 mmol) were added. The reaction mixture was then warmed to room temperature and then heated to 60 ºC. After 1 hour the medium was cooled to 0 °C, hydrolysed with a saturated aqueous solution of NH_4Cl and extracted with EtOAc (3x). The combined organic layers were washed with brine, dried with Na_2SO_4, filtered, and the solvent evaporated. The residue was purified by flash column chromatography (silica gel; petroleum ether/EtOAc, 5:1) to afford 5.65 g of **S1** (64% yield) as a yellow oil. The spectroscopic data are in agreement with those previously reported in the literature.[1] HRMS (ESI): *m/z* calcd for $C_{14}H_{18}NO_3$ $[M+H]^+$: 248.1287; found: 248.1275.

[1] S. Fustero, P. Bello, J. Miro, M. Sánchez-Roselló, M. A. Maestro, J. Gonzalez, C. del Pozo, *Chem. Comm*. **2013**, *49*, 1336.

A solution of CAN (6.20 g, 12.4 mmol) in H_2O (39 mL) was added drop-wise over 15 minutes to a stirred solution of **S1** (1.50 g, 6.20 mmol) in MeCN (39 mL) cooled to 0 ºC. After 2 hours stirring, the resulting solution was treated with 2N HCl to achieve pH 1. The aqueous phase was washed with EtOAc (3x) and basified to pH 10-12 by the addition of Na_2CO_3. The resulting suspension was extracted with CH_2Cl_2 (3x), dried over Na_2SO_4, filtered, and the solvents evaporated under reduced pressure to yield 570 mg of **1** (65% yield) as an orange oil without further purification. The spectroscopic data are in agreement with those previously reported in the literature.[2] HRMS (ESI): *m/z* calcd for $C_7H_{12}NO_2$ $[M+H]^+$: 142.0863; found: 142.0865.

Synthesis of methyl 2-((*tert*-butoxycarbonyl)amino)-2-methylpent-4-ynoate (S2)

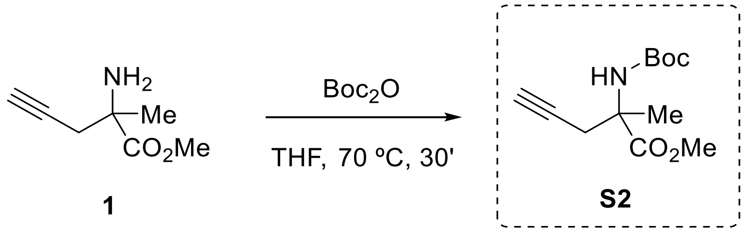

Following an improved version of a reported procedure,[3] Boc_2O (465 mg, 2.13 mmol) was added to a solution of **1** (200 mg, 1.42 mmol) in THF (7 mL) and the resulting mixture was stirred at 70 ºC overnight (in a sealed tube). Then, the solvent was evaporated under reduced pressure and the crude product was purified by flash column chromatography (silica gel; petroleum ether/EtOAc, 4:1) to yield 339 mg of **S2** (99% yield) as a white solid. The spectroscopic data are in agreement with those previously reported in the literature for (*S*)-*N*-Boc-a-propargylalanine methyl ester.[3] HRMS (ESI): *m/z* calcd for $C_{12}H_{20}NO_4$ $[M+H]^+$: 242.1392; found: 242.1382.

[2] J. A. Bajgrowicz, B. Cossec, C. Pigiere, R. Jacquier, P. Viallefont, *Tetrahedron Letters* **1983**, 24, 3721.
[3] H. Sogawa, M. Shiotsuki, F. Sanda, *J. Polym. Sci. Part A: Polym. Chem.* **2012**, *50*, 2008.

Synthesis of methyl 3-methyl-7-phenyl-1,2,3,4-tetrahydro-2,6-naphthyridine-3-carboxylate (2) and methyl 3-methyl-6-phenyl-1,2,3,4-tetrahydro-2,7-naphthyridine-3-carboxylate (3)

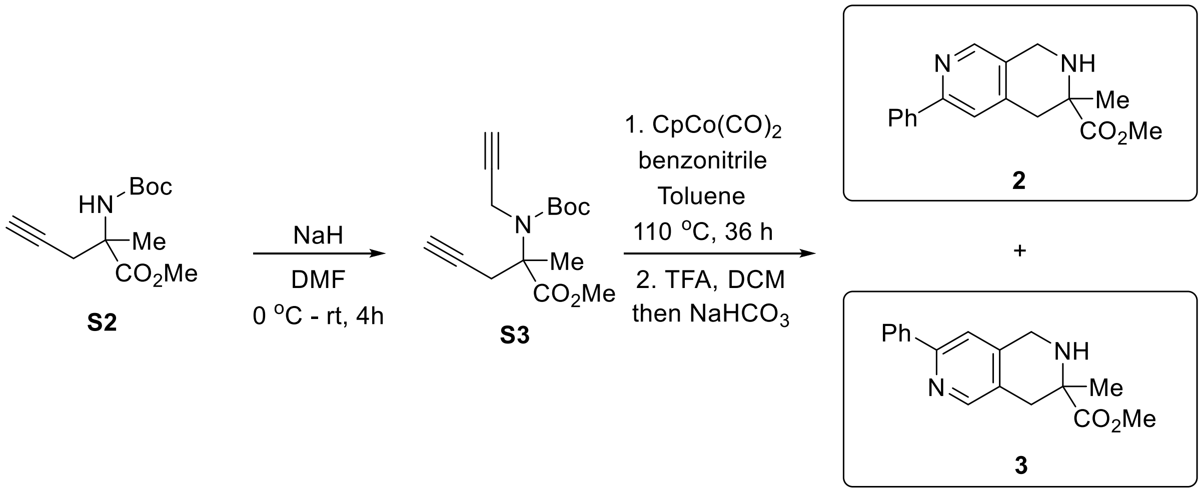

A solution of **S2** (150 mg, 0.62 mmol) in DMF (3.0 mL) was added to a suspension of sodium hydride (60% in mineral oil, 30 mg, 0.74 mmol) in DMF (3.2 mL) cooled to 0 ºC. After 10 minutes stirring, propargyl bromide (80 wt. % in toluene, 0.138 mL, 1.24 mmol) was added and the mixture was warmed to room temperature and stirred for 4 hours. Then, the mixture was diluted with saturated aqueous solution of NH_4Cl and the aqueous layer was extracted with EtOAc (3x). The combined organic layers were washed with brine, dried with Na_2SO_4, filtered, and the solvents were evaporated. The crude product was purified by flash column chromatography (silica gel; petroleum ether/EtOAc, 5:1) to yield 168 mg of methyl 2-((*tert*-butoxycarbonyl)(prop-2-yn-1-yl)amino)-2-methylpent-4-ynoate (**S3**) (97% yield) as a colourless oil. R_f = 0.40 (petroleum ether/EtOAc; 5:1); ^{1}H NMR (400 MHz, $CDCl_3$) δ 1.43 (s, 9H), 1.70 (s, 3H), 1.99 (t, *J* = 2.6 Hz, 1H), 2.20 (t, *J* = 2.4 Hz, 1H), 2.73 (dd, *J* = 17.1, 2.5 Hz, 1H), 3.12 (d, *J* = 16.6 Hz, 1H), 3.69 (s, 3H), 4.02 (d, *J* = 18.5 Hz, 1H), 4.26 (br s, 1H); ^{13}C NMR (101 MHz, $CDCl_3$) δ 22.0, 27.0, 28.3, 34.1, 52.4, 62.5, 70.9, 71.1, 80.1, 80.9, 81.7, 154.3, 174.0; HRMS (ESI): *m/z* calcd for $C_{15}H_{22}NO_4$ $[M+H]^+$: 280.1543; found: 280.1534.

$CpCo(CO)_2$ (6.4 mg, 0.036 mmol) and benzonitrile (0.05 mL, 0.48 mmol) were added to a solution of **S3** (68 mg, 0.24 mmol) in toluene (2.4 mL), previously degassed with argon for

15 minutes, and the mixture was heated at 110 ºC for 36 hours. Then, the solvent was removed under reduced pressure and the crude product was purified by column chromatography (silica gel; petroleum ether/EtOAc, gradient from 5:1 to 3:2). The resulting inseparable mixture of regioisomers (ca. 40:60 ratio as determined by ^{1}H NMR) was dissolved in CH_2Cl_2 (1 mL) and TFA (0.5 mL) was then added to the solution. After 1 hour stirring the solvent was removed under reduced pressure. The crude product was dissolved in EtOAc (5 mL) and a saturated aqueous solution of $NaHCO_3$ (5 mL) was added and the mixture was stirred for 10 minutes. Then, the organic layer was separated, dried over Na_2SO_4, filtered and concentrated *in vacuo*. The crude product was purified by flash column chromatography (silica gel; gradient from EtOAc:MeOH 1:0 to 9:1) to afford 20 mg of **2** (30% yield) and 18 mg of **3** (26% yield) both as a colourless oils.

Data of minor regioisomer (2):

R_f = 0.21 (EtOAc): ^{1}H NMR (400 MHz, $CDCl_3$) δ 1.48 (s, 3H), 2.37 (br s, 1H), 2.83 (d, *J* = 16.7 Hz, 1H), 3.35 (d, *J* = 16.7 Hz, 1H), 3.69 (s, 3H), 4.05 – 4.18 (m, 2H), 7.39 (ddd, *J* = 7.3, 3.7, 1.3 Hz, 1H), 7.41 – 7.49 (m, 3H), 7.90 – 7.98 (m, 2H), 8.37 (s, 1H); ^{13}C NMR (101 MHz, $CDCl_3$) δ 26.6, 37.3, 42.6, 52.5, 57.9, 120.6, 126.9, 128.3, 128.8, 128.8, 139.4, 143.2, 147.8, 155.3, 175.9; HRMS (ESI): *m/z* calcd for $C_{17}H_{19}N_2O_2$ $[M+H]^+$: 283.1441; found: 283.1431.

Data of major regioisomer (3):

R_f = 0.32 (EtOAc): ^{1}H NMR (400 MHz, $CDCl_3$) δ 1.48 (s, 3H), 2.03 (br s, 1H), 2.80 (d, *J* = 16.2 Hz, 1H), 3.33 (d, *J* = 16.2 Hz, 1H), 3.68 (s, 3H), 4.08 (d, *J* = 17.0 Hz, 1H), 4.19 (d, *J* = 17.1 Hz, 1H), 7.34 – 7.41 (m, 2H), 7.41 – 7.48 (m, 2H), 7.86 – 7.97 (m, 2H), 8.43 (s, 1H); ^{13}C NMR (101 MHz, CDCl3) δ 26.4, 34.7, 44.7, 52.6, 58.1, 117.8, 126.9, 127.8, 128.8, 129.0, 139.4, 143.3, 150.2, 155.1, 175.8; HRMS (ESI): *m/z* calcd for $C_{17}H_{19}N_2O_2$ $[M+H]^+$: 283.1441; found: 283.1431.

Synthesis of methyl 4-methyl-6-methylene-2-oxo-1,3-oxazinane-4-carboxylate (4)

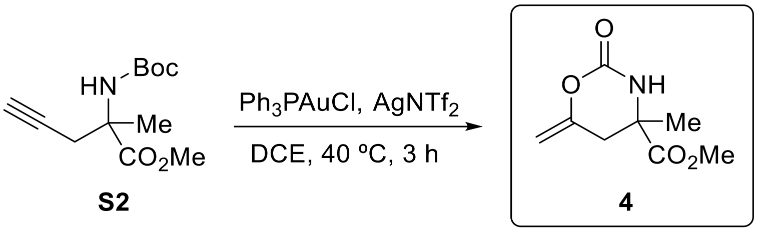

A suspension of Ph_3PAuCl (2 mg, 5 mol%) and $AgNTf_2$ (1.6 mg, 5 mol%) in DCE (0.1 mL) was added drop-wise to a stirred solution of **S2** (20 mg, 0.083 mmol) in DCE (1 mL) and the resulting solution was stirred at 40 ºC for 3 hours. The solvent was removed under reduced pressure and the crude product was purified by flash column chromatography (silica gel; petroleum ether/EtOAc, 1:1) to afford 10 mg of **4** (65% yield) as a colourless oil. R_f = 0.23 (petroleum ether/EtOAc, 1:1); mp 66 – 68 ºC; ^{1}H NMR (400 MHz, $CDCl_3$) δ 1.50 (s, 3H), 2.57 (d, J = 14.3 Hz, 1H), 2.82 (d, J = 14.3 Hz, 1H), 3.78 (s, 3H), 4.38 (s, 1H), 4.79 (d, J = 1.1 Hz, 1H), 6.31 (br s, 1H); ^{13}C NMR (101 MHz, $CDCl_3$) δ 25.5, 35.2, 53.4, 56.9, 96.3, 149.8, 150.4, 172.6; HRMS (ESI): m/z calcd for $C_8H_{12}NO_4$ $[M+H]^+$: 186.0761; found: 186.0764.

Synthesis of methyl 5,6-dimethyl-7-oxo-4,5,6,7-tetrahydrobenzo[*g*][1,2,3]triazolo[1,5-*a*][1,5]diazocine-5-carboxylate (5)

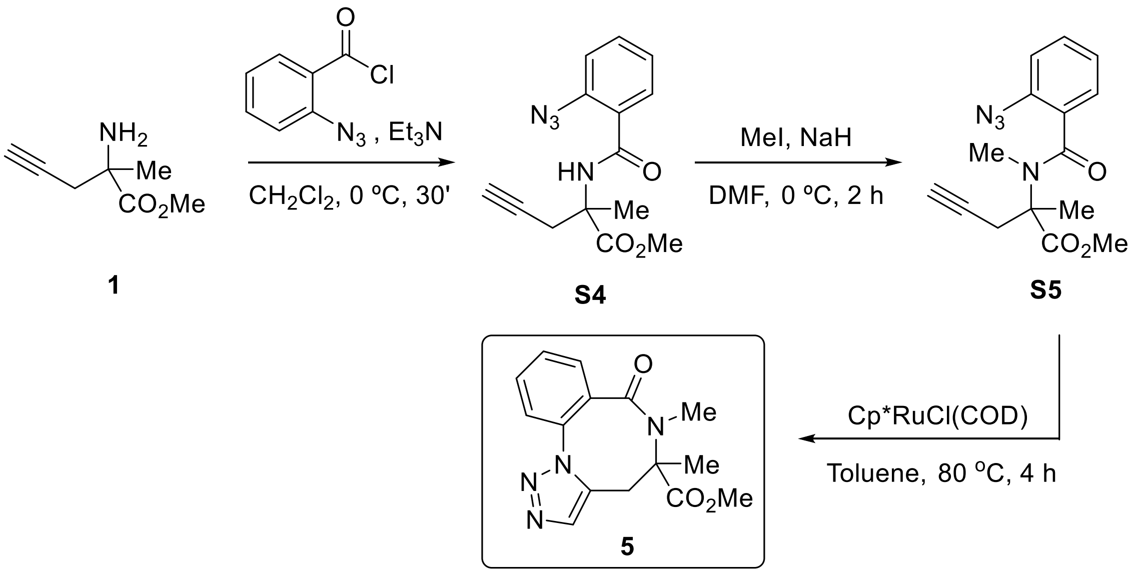

2-Azidobenzoyl chloride (120 mg, 0.66 mmol) and Et_3N (0.09 mL, 0.66 mmol) were added to a solution of **1** (62 mg, 0.44 mmol) in CH_2Cl_2 (2.2 mL) cooled to 0 ºC. After 30 minutes stirring, the mixture was diluted with saturated aqueous solution of NH_4Cl and the aqueous

layer was extracted with CH_2Cl_2 (3x). The organic layer was separated, dried over Na_2SO_4, filtered and the solvents were concentrated *in vacuo*. The crude product was purified by flash column chromatography (silica gel; petroleum ether/EtOAc, 2:1) to yield 104 mg of methyl 2-(2-azidobenzamido)-2-methylpent-4-ynoate (**S4**) (83% yield) as a white solid. R_f = 0.33 (petroleum ether/EtOAc, 2:1); mp 115 – 117 ºC; ^{1}H NMR (400 MHz, $CDCl_3$) δ 1.72 (s, 3H), 2.03 (t, *J* = 2.6 Hz, 1H), 3.08 (d, *J* = 2.6 Hz, 2H), 3.79 (s, 3H), 7.23 (dd, *J* = 13.4, 7.7 Hz, 2H), 7.51 (td, *J* = 7.9, 1.5 Hz, 1H), 8.13 (dd, *J* = 7.9, 1.4 Hz, 1H), 8.29 (br s, 1H); ^{13}C NMR (101 MHz, $CDCl_3$) δ 23.0, 26.8, 53.1, 59.3, 71.3, 118.6, 124.6, 125.3, 132.4, 132.8, 137.5, 163.8, 173.4; HRMS (ESI): *m/z* calcd for $C_{14}H_{14}N_4NaO_3$ $[M+Na]^+$: 309.0958; found: 309.0952.

Sodium hydride (60% in mineral oil, 12.0 mg, 0.30 mmol) was added to a solution of **S4** (55.0 mg, 0.20 mmol) in DMF (1 mL) cooled to 0 ºC. After 10 min stirring, iodomethane (0.025 mL, 0.40 mmol) was added and the mixture was stirred for 2 hours. Then, the mixture was diluted with saturated aqueous solution of NH_4Cl and the aqueous layer was extracted with EtOAc (3x). The organic layer was separated, dried over Na_2SO_4, filtered and the solvents were concentrated *in vacuo*. The crude product was purified by flash column chromatography (silica gel; petroleum ether/EtOAc, 4:1) to yield 57 mg of methyl 2-(2-azido-*N*-methylbenzamido)-2-methylpent-4-ynoate (**S5**) (95% yield) as a white solid. R_f = 0.24 (petroleum ether/EtOAc, 4:1); mp 94 – 96 ºC; ^{1}H NMR (400 MHz, $CDCl_3$) δ 1.68 (s, 3H), 2.03 (s, 1H), 2.65 – 2.90 (br s, 1H), 3.00 (s, 3H), 3.51 (d, *J* = 17.2 Hz, 1H), 3.76 (s, 3H), 7.08 – 7.33 (m, 3H), 7.32 – 7.51 (m, 1H); ^{13}C NMR (101 MHz, $CDCl_3$) δ 21.9, 25.4, 34.5, 52.6, 62.0, 70.6, 80.5, 118.7, 125.2, 128.0, 128.9, 130.6, 136.7, 169.0, 173.4; HRMS (ESI): *m/z* calcd for $C_{15}H_{16}N_4NaO_3$ $[M+Na]^+$: 323.1115; found: 323.1108.

A solution of **S5** (30 mg, 0.10 mmol) in toluene (2 mL) was degassed for 15 minutes with an argon purge. Cp*RuCl(COD) (3.8 mg, 10 mol%) was added and the mixture was stirred at 80 ºC for 4 hours. Then, the solvent was removed under reduced pressure and the crude

product was purified by flash column chromatography over silica gel (silica gel; EtOAc) to yield 16 mg of **5** (53% yield) as a white solid. R_f = 0.26 (EtOAc); mp 176 – 178 °C; ^{1}H NMR (400 MHz, $CDCl_3$) δ 1.75 (s, 3H), 3.02 (s, 3H), 3.24 (d, *J* = 16.6 Hz, 1H), 3.45 (s, 3H), 3.89 (d, *J* = 16.5 Hz, 1H), 7.43 – 7.65 (m, 5H); ^{13}C NMR (101 MHz, $CDCl_3$) δ 29.1, 30.4, 32.0, 53.3, 64.0, 126.1, 130.0, 130.9, 131.6, 132.9, 133.2, 133.5, 133.8, 168.5, 173.1; HRMS (EI): *m/z* calcd for $C_{15}H_{17}N_4O_3$ $[M+H]^+$: 301.1295; found: 301.1288.

Synthesis of methyl 2-acetamido-2-methylpent-4-ynoate (S6)

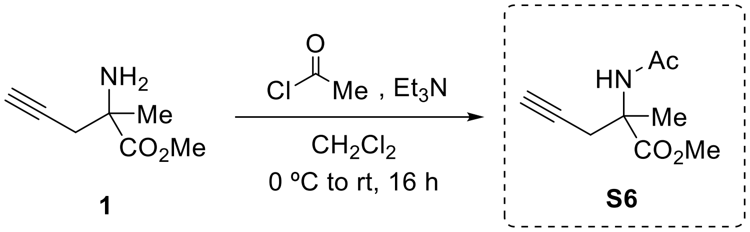

To a solution of **1** (50 mg, 0.35 mmol) in CH_2Cl_2 (1 mL) cooled to 0 °C, was added acetyl chloride (0.037 mL, 0.53 mmol) and Et_3N (0.10 mL, 0.70 mmol) and the mixture warmed to room temperature and stirred for 16 hours. Saturated aqueous solution of NH_4Cl was added and the organic layer was separated. The aqueous layer was extracted with CH_2Cl_2 (2x) and the combined organic layers were dried over Na_2SO_4, filtered and concentrated *in vacuo*. The crude product was purified by flash column chromatography (silica gel; petroleum ether/EtOAc; 4:1) to afford 58 mg of **S6** (89% yield) as a white solid. R_f = 0.35 (petroleum ether/EtOAc, 1:2); mp 100 – 102 °C; ^{1}H NMR (400 MHz, $CDCl_3$) δ 1.53 (s, 3H), 1.95 – 1.99 (m, 4H), 2.87 – 2.99 (m, 2H), 3.72 (s, 3H), 6.43 (br s, 1H); ^{13}C NMR (101 MHz, $CDCl_3$) δ 22.8, 23.4, 26.3, 52.9, 58.5, 71.1, 79.6, 169.8, 173.5; HRMS (ESI): *m/z* calcd for $C_9H_{13}NNaO_3$ $[M+Na]^+$: 206.0788; found: 206.0778.

Synthesis of methyl 2,6-dimethyl-4-oxo-1,2,3,4-tetrahydropyridine-2-carboxylate (6)

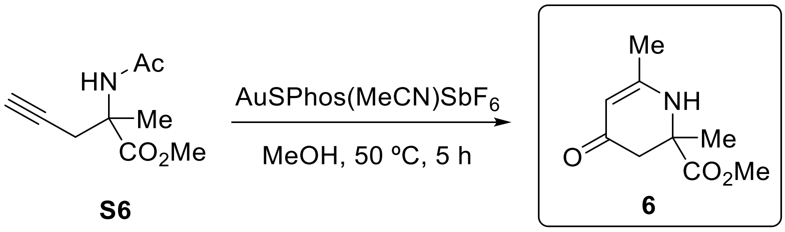

To a stirred solution of **S6** (20 mg, 0.109 mmol) in MeOH (2.3 mL), AuSPhos(MeCN)SbF_6 (4.8 mg, 5 mol%) was added and the resulting solution was heated at 50 °C for 5 hours. Then, the solvent was removed under reduced pressure and the crude product was purified by flash column chromatography (silica gel; EtOAc) to yield 15 mg of **6** (75% yield) as a white solid. R_f = 0.20 (EtOAc); mp 111 – 113 °C; ^{1}H NMR (400 MHz, $CDCl_3$) δ 1.50 (s, 3H), 2.00 (s, 3H), 2.49 (d, *J* = 16.3 Hz, 1H), 2.83 (d, *J* = 16.3 Hz, 1H), 3.76 (s, 3H), 4.97 (s, 1H), 5.14 (br s, 1H); ^{13}C NMR (101 MHz, $CDCl_3$) δ 21.5, 24.3, 44.0, 53.2, 60.5, 100.0, 160.6, 174.7, 190.6; HRMS (EI): *m/z* calcd for $C_9H_{14}NO_3$ $[M+H]^+$: 184.0968; found: 184.0967.

Synthesis of methyl 4-(2-amino-3-methoxy-2-methyl-3-oxopropyl)isoxazole-3-carboxylate (7)

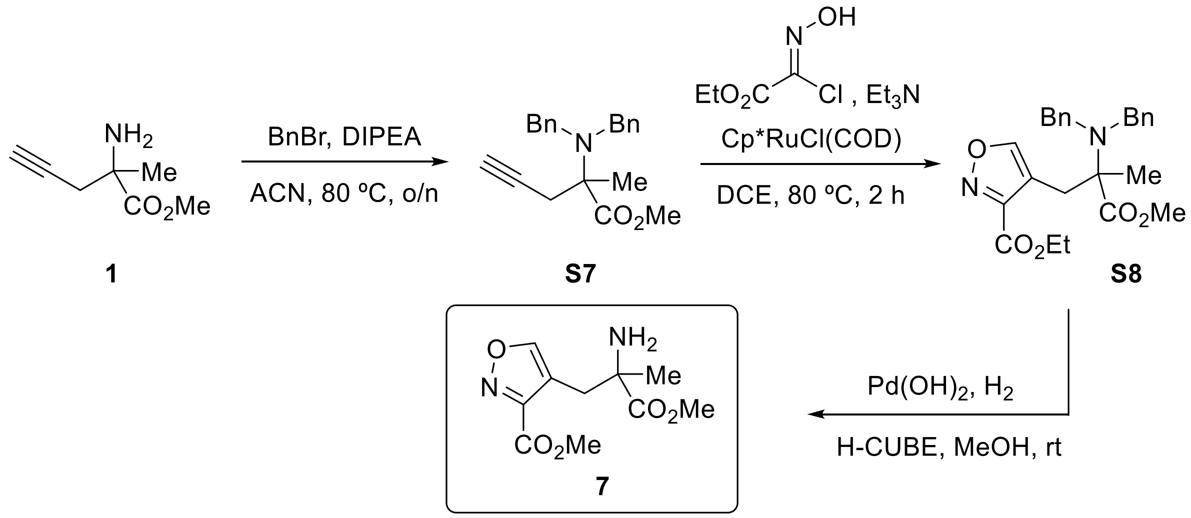

Benzyl bromide (0.56 mL, 4.68 mmol) and DIPEA (0.82 mL, 4.68 mmol) were added to a solution of **1** (220 mg, 1.56 mmol) in MeCN (10 mL) and the reaction mixture was heated at 80 °C overnight (in a sealed tube). Then, the solvent was removed *in vacuo* and the resulting residue was diluted in EtOAc. The organic layer was washed with saturated aqueous

solution of $NaHCO_3$, dried over Na_2SO_4, filtered and concentrated *in vacuo*. The crude product was purified by flash column chromatography (silica gel; petroleum ether/EtOAc; 9:1) to afford 347 mg of methyl 2-(dibenzylamino)-2-methylpent-4-ynoate (**S7**) (69% yield) as a colourless oil. R_f = 0.36 (petroleum ether/EtOAc; 9:1); ^{1}H NMR (400 MHz, $CDCl_3$) δ 1.52 (s, 3H), 2.10 (t, *J* = 2.7 Hz, 1H), 2.75 (qd, *J* = 16.8, 2.6 Hz, 2H), 3.73 (s, 3H), 3.86 (s, 4H), 7.11 – 7.16 (m, 2H), 7.18 – 7.24 (m, 4H), 7.31 – 7.36 (m, 4H); ^{13}C NMR (101 MHz, $CDCl_3$) δ 22.4, 28.2, 51.8, 54.9, 66.7, 71.3, 81.0, 126.7, 128.0, 128.5, 141.0, 174.3; HRMS (ESI): *m/z* calcd for $C_{21}H_{24}NO_2$ $[M+H]^+$: 322.1807; found: 322.1818.

A solution of **S7** (40 mg, 0.12 mmol) in DCE (1.2 mL) was degassed for 15 minutes with an argon purge. Cp*RuCl(COD) (10 mol%, 4.60 mg, 0.012 mmol), ethyl 2-chloro-2-(hydroxyimino)acetate (20 mg, 0.13 mmol) and Et_3N (0.021 mL, 0.15 mmol) were added and the mixture was stirred at 80 ºC for 2 hours. Then, the solvent was removed under reduced pressure and the crude product was purified by flash column chromatography (silica gel; petroleum ether/EtOAc, 6:1) to yield 30 mg of ethyl 4-(2-(dibenzylamino)-3-methoxy-2-methyl-3-oxopropyl)isoxazole-3-carboxylate (**S8**) (57% yield) as a colourless oil. R_f = 0.30 (petroleum ether/EtOAc, 6:1); ^{1}H NMR (400 MHz, $CDCl_3$) δ 1.27 (s, 3H), 1.41 (t, *J* = 7.1 Hz, 3H), 3.29 (d, *J* = 14.8 Hz, 1H), 3.41 (d, *J* = 14.8 Hz, 1H), 3.64 (s, 3H), 3.81 – 3.93 (m, 4H), 4.41 (q, *J* = 7.1 Hz, 2H), 7.10 – 7.17 (m, 2H), 7.17 – 7.27 (m, 8H), 8.38 (s, 1H); ^{13}C NMR (101 MHz, $CDCl_3$) δ 14.3, 21.1, 28.2, 51.8, 55.0, 62.1, 68.1, 116.3, 126.9, 128.2, 128.6, 140.8, 154.5, 159.5, 160.8, 174.7; HRMS (ESI): *m/z* calcd for $C_{25}H_{29}N_2O_5$ $[M+H]^+$: 437.2071; found: 437.2066.

A solution of **S8** (15 mg, 0.034 mmol) in MeOH (1.7 mL) was hydrogenated in an H-CUBE reactor (1ml/min, $Pd(OH)_2$ 20% cartridge, full H_2, room temperature). The solvent was evaporated *in vacuo* and the crude product was purified by flash column chromatography (silica gel; EtOAc) to yield 6 mg of **7** (72% yield) as a white solid. R_f = 0.35 (EtOAc 100%); mp 173 – 175 ºC; ^{1}H NMR (400 MHz, $CDCl_3$) δ 1.35 (s, 3H), 1.64 (br s, 2H), 3.03 (d, *J* = 14.6

Hz, 1H), 3.15 (d, J = 14.6 Hz, 1H), 3.71 (s, 3H), 3.97 (s, 3H), 8.45 (s, 1H); ^{13}C NMR (126 MHz, $CDCl_3$) δ 26.5, 32.0, 52.6, 52.8, 58.0, 115.9, 154.5, 159.0, 161.4, 177.1; HRMS (EI): *m/z* calcd for $C_{10}H_{15}N_2O_5$ $[M+H]^+$: 243.0975; found: 243.0969.

Synthesis of methyl 2-amino-3-(1-(2-ethoxy-2-oxoethyl)-1*H*-1,2,3-triazol-5-yl)-2-methylpropanoate (8)

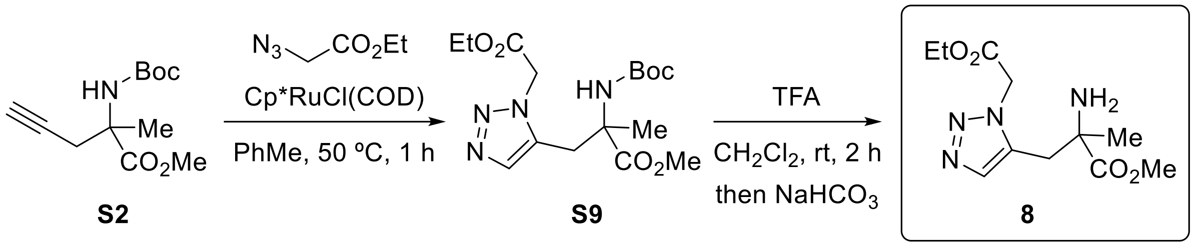

A solution of **S2** (1.0 g, 4.14 mmol) and ethyl 2-azidoacetate (803 mg, 6.22 mmol) in toluene (24 mL) was degassed for 15 minutes with an argon purge. Cp*RuCl(COD) (38 mg, 0.1 mmol) was added and the mixture was stirred at 50 ºC for 1 hour. Then, the solvent was removed under reduced pressure and the crude product was purified by flash column chromatography (silica gel; petroleum ether/EtOAc, gradient from 1:1 to 1:2) to yield 1.51 g of 2-((*tert*-butoxycarbonyl)amino)-3-(1-(2-ethoxy-2-oxoethyl)-1*H*-1,2,3-triazol-5-yl)-2-methylpropanoate (**S9**) (99% yield) as a white solid. R_f = 0.25 (petroleum ether/EtOAc, 1:1); mp 67 – 69 ºC; ^{1}H NMR (400 MHz, $CDCl_3$) δ 1.27 (t, J = 7.1 Hz, 3H), 1.41 (s, 9H), 1.53 (s, 3H), 3.39 (d, J = 15.5 Hz, 1H), 3.53 (d, J = 15.5 Hz, 1H), 3.78 (s, 3H), 4.22 (q, J = 7.1 Hz, 2H), 5.13 (s, 2H), 5.27 (s, 1H), 7.41 (s, 1H); ^{13}C NMR (101 MHz, $CDCl_3$) δ 14.2, 24.3, 28.4, 29.3, 48.9, 53.2, 59.2, 62.4, 80.6, 133.5, 133.8, 154.7, 166.6, 173.7; HRMS (EI): *m/z* calcd for $C_{16}H_{27}N_4O_6$ $[M+H]^+$: 371.125; found: 371.1927.

TFA (10 mL) was added to a solution of **S9** (1.51 g, 4.08 mmol) in CH_2Cl_2 (30 mL) and after 2 hours stirring the solvent was removed under reduced pressure. The crude product was dissolved in EtOAc and a saturated aqueous solution of $NaHCO_3$ was added and the mixture was stirred for 10 minutes. Then, the organic layer was separated, dried over Na_2SO_4,

filtered and concentrated *in vacuo* to afford 880 mg **8** (80% yield) as a white solid without further purification. R_f = 0.22 (EtOAc); mp 73 – 75 °C; ^{1}H NMR (400 MHz, $CDCl_3$) δ 1.27 (t, *J* = 7.1 Hz, 3H), 1.38 (s, 3H), 1.73 (br s, 2H), 2.83 (d, *J* = 14.9 Hz, 1H), 3.15 (d, *J* = 14.9 Hz, 1H), 3.71 (s, 3H), 4.22 (q, *J* = 7.1 Hz, 2H), 5.20 – 5.48 (m, 2H), 7.48 (s, 1H); ^{13}C NMR (101 MHz, $CDCl_3$) δ 14.2, 27.0, 34.1, 49.5, 52.8, 58.3, 62.3, 133.9, 133.9, 166.9, 176.6; HRMS (EI): *m/z* calcd for $C_{11}H_{18}N_4NaO_4$ $[M+Na]^+$: 293.1220; found: 293.1212.

Synthesis of methyl 5-methyl-7-oxo-4,5,6,7-tetrahydroisoxazolo[3,4-*c*]pyridine-5-carboxylate (9)

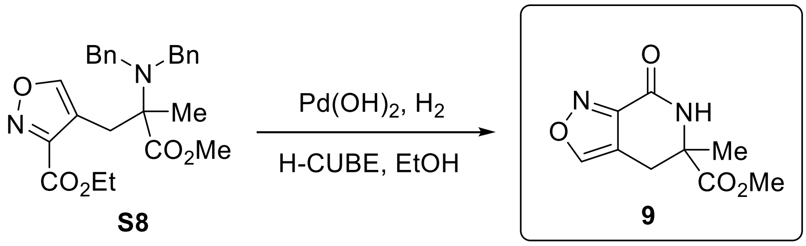

A solution of **S8** (15 mg, 0.034 mmol) in EtOH (1.7 mL) was hydrogenated in an H-CUBE reactor (1ml/min, $Pd(OH)_2$ 20% cartridge, full H_2, room temperature). The solvent was evaporated *in vacuo* in a warmed bath at 40 °C. The crude product was purified by flash column chromatography (silica gel; petroleum ether/EtOAc, 1:2) to yield 5 mg of **9** (69% yield) as a white solid. R_f = 0.24 (petroleum ether/EtOAc, 1:2); mp 174 – 176 °C; ^{1}H NMR (500 MHz, $CDCl_3$) δ 1.59 (s, 3H), 2.96 (dd, *J* = 15.6, 1.2 Hz, 1H), 3.32 (d, *J* = 15.6 Hz, 1H), 3.75 (s, 3H), 6.41 (br s, 1H), 8.38 (t, *J* = 1.1 Hz, 1H); ^{13}C NMR (126 MHz, $CDCl_3$) δ 26.2, 28.4, 53.6, 61.0, 113.9, 153.4, 155.3, 158.3, 172.9; HRMS (ESI): *m/z* calcd for $C_9H_{10}NaN_2O_4$ $[M+Na]^+$: 233.0533; found: 233.0526.

Synthesis of methyl 5-methyl-7-oxo-5,6,7,8-tetrahydro-4*H*-[1,2,3]triazolo[1,5-*d*][1,4]diazepine-5-carboxylate (10)

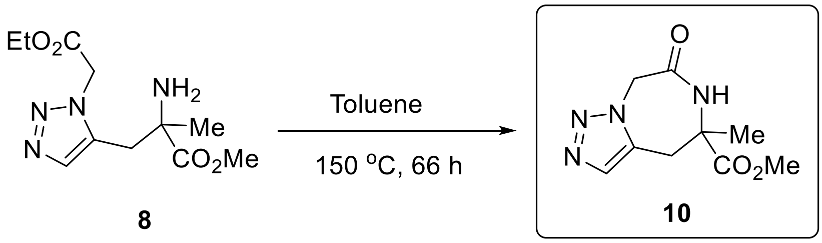

A stirred solution of **8** (200 mg, 0.74 mmol) in toluene (12 mL) was heated at 150º C for 66 hours. Then, the solvent was removed under reduced pressure and the crude product was purified by flash column chromatography (silica gel; EtOAc/MeOH, gradient from 1:0 to 9:1) to afford 140 mg of **10** (84% yield) as a white solid. R_f = 0.18 (EtOAc 100%); mp 147 – 149 ºC; ^{1}H NMR (400 MHz, $CDCl_3$) δ 1.54 (s, 3H), 3.34 (d, J = 15.8 Hz, 1H), 3.56 (d, J = 15.7 Hz, 1H), 3.70 (s, 3H), 5.28 (m, 2H), 6.90 (br s, 1H), 7.54 (s, 1H); ^{13}C NMR (101 MHz, $CDCl_3$) δ 27.4, 31.0, 52.7, 53.7, 60.8, 131.9, 133.6, 165.2, 172.1; HRMS (EI): *m/z* calcd for $C_9H_{13}N_4O_3$ $[M+H]^+$: 225.0982; found: 225.0978.

Synthesis of methyl 2-amino-2-methyl-3-(1-phenyl-1*H*-1,2,3-triazol-5-yl)propanoate (11)

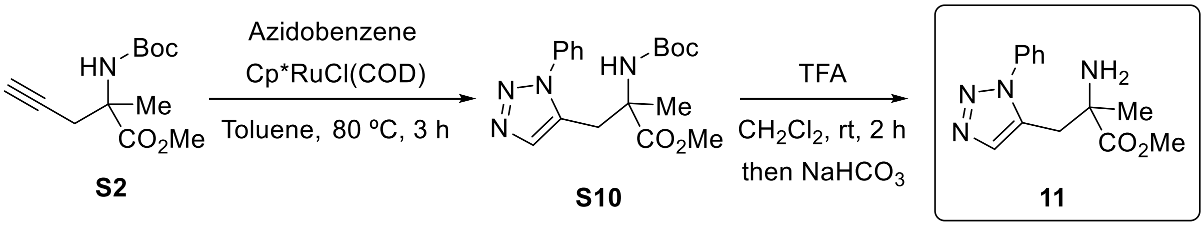

A solution of **S2** (50 mg, 0.20 mmol) and azidobenzene (0.5 M in *tert*-butyl methyl ether, 0.62 mL, 0.31 mmol) in toluene (1 mL) was degassed for 15 minutes with an argon purge. Cp*RuCl(COD) (3.7 mg, 0.01 mmol) was added and the mixture was stirred at 80 ºC for 3 hours. Then, the solvent was removed under reduced pressure and the crude product was purified by flash column chromatography (silica gel; petroleum ether/EtOAc, 1:1) to yield 46 mg of methyl 2-((*tert*-butoxycarbonyl)amino)-2-methyl-3-(1-phenyl-1*H*-1,2,3-triazol-5-yl)propanoate (**S10**) (64% yield) as a colourless oil. R_f = 0.24 (petroleum ether/EtOAc, 1:1); ^{1}H NMR (400 MHz, $CDCl_3$) δ1.34 (s, 3H), 1.40 (s, 9H), 3.45 (d, J = 15.5 Hz, 1H), 3.52 – 3.71

(m, 4H), 5.30 (s, 1H), 7.37 – 7.41 (m, 2H), 7.49 – 7.57 (m, 3H), 7.59 (s, 1H); ^{13}C NMR (101 MHz, $CDCl_3$) δ 24.1, 28.4, 29.4, 53.0, 58.8, 80.4, 125.9, 129.7, 129.8, 133.4, 133.7, 136.3, 154.3, 173.6; HRMS (EI): *m/z* calcd for $C_{18}H_{24}N_4NaO_4$ $[M+Na]^+$: 383.1695; found: 383.1698.

TFA (0.4 mL) was added to a solution of **S10** (46 mg, 0.13 mmol) in CH_2Cl_2 (1 mL) and after 2 hours stirring the solvent was removed under reduced pressure. The crude product was dissolved in EtOAc and a saturated aqueous solution of $NaHCO_3$ was added and the mixture was stirred for 10 minutes. Then, the organic layer was separated, dried over Na_2SO_4, filtered and concentrated *in vacuo*. The crude product was purified by flash column chromatography (silica gel; EtOAc/MeOH, from gradient 1:0 to 20:1) to afford 21 mg **11** (62% yield) as a white solid without further purification. R_f = 0.19 (EtOAc); mp 65 – 67 ºC; ^{1}H NMR (400 MHz, $CDCl_3$) δ 1.28 (s, 3H), 1.65 (s, 2H), 2.96 (d, *J* = 15.0 Hz, 1H), 3.20 (d, *J* = 15.0 Hz, 1H), 3.64 (s, 3H), 7.45 – 7.50 (m, 2H), 7.50 – 7.60 (m, 3H), 7.63 (s, 1H); ^{13}C NMR (101 MHz, $CDCl_3$) δ 26.8, 34.2, 52.7, 58.0, 126.2, 129.6, 129.8, 133.4, 133.7, 136.3, 176.7; HRMS (EI): *m/z* calcd for $C_{13}H_{17}N_4O_2$ $[M+H]^+$: 261.1346; found: 261.1336.

Synthesis of methyl 2-amino-2-methyl-3-(1-phenyl-1*H*-1,2,3-triazol-4-yl)propanoate (12)

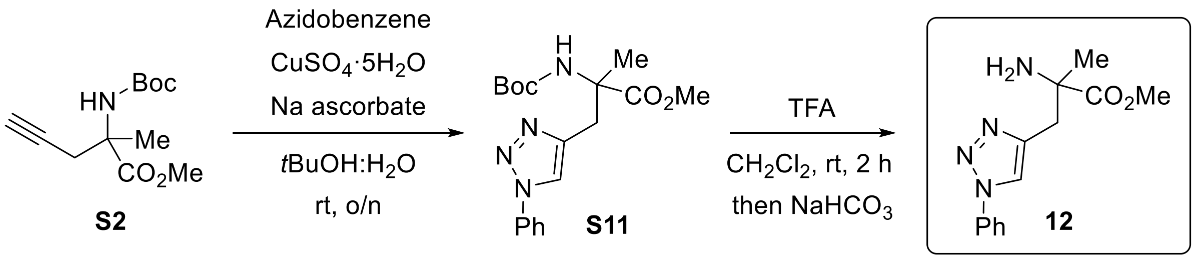

$CuSO_4·5H_2O$ (6.47 mg, 5 mol%) and sodium ascorbate (31 mg, 0.155 mmol) were added to a solution of **S2** (125 mg, 0.52 mmol) and azidobenzene (0.5 M in *tert*-butyl methyl ether, 2.08 mL, 1.04 mmol) in a mixture 1:1 of *t*-BuOH:H_2O (2 mL) and the reaction stirred overnight. Then, the reaction was concentrated *in vacuo* and the aqueous layer was extracted with EtOAc (3x). The combined organic layer was washed with 5% aqueous

solution of NH_4OH, dried over Na_2SO_4, filtered and concentrated *in vacuo*. The crude product was purified by flash column chromatography (silica gel; petroleum ether/EtOAc, 2:1) to yield 177 mg of methyl 2-((*tert*-butoxycarbonyl)amino)-2-methyl-3-(1-phenyl-1*H*-1,2,3-triazol-4-yl)propanoate (**S11**) (95% yield) as white solid. R_f = 0.23 (petroleum ether/EtOAc, 2:1); mp 99 – 101 ºC; ^{1}H NMR (400 MHz, $CDCl_3$) δ 1.43 (s, 9H), 1.60 (s, 3H), 3.45 (d, *J* = 14.4 Hz, 1H), 3.52 (d, *J* = 14.6 Hz, 1H), 3.79 (s, 3H), 5.44 (br s, 1H), 7.42 (t, *J* = 7.4 Hz, 1H), 7.51 (t, *J* = 7.7 Hz, 2H), 7.70 (d, *J* = 7.8 Hz, 2H), 7.75 (s, 1H); ^{13}C NMR (101 MHz, $CDCl_3$) δ 23.7, 28.3, 32.6, 52.8, 59.2, 79.7, 120.3, 120.8, 128.6, 129.7, 137.0, 143.6, 154.0, 174.3; HRMS (ESI): *m/z* calcd for $C_{18}H_{25}N_4O_4$ $[M+H]^+$: 361.1870; found: 361.1868.

TFA (0.4 mL) was added to a solution of **S11** (50 mg, 0.14 mmol) in CH_2Cl_2 (1 mL) and after 2 hours stirring the solvent was removed under reduced pressure. The crude product was dissolved in EtOAc and a saturated aqueous solution of $NaHCO_3$ was added and the mixture was stirred for 10 minutes. Then, the organic layer was separated, dried over Na_2SO_4, filtered and concentrated *in vacuo*. The crude product was purified by flash column chromatography (silica gel; EtOAc/MeOH, 10:2) to yield to afford 36 mg **12** (98% yield) as a white solid. R_f = 0.36 (EtOAc/MeOH, 10:2); mp 72 – 74 ºC; ^{1}H NMR (400 MHz, $CDCl_3$) δ 1.44 (s, 3H), 1.89 (br s, 2H), 3.05 (d, *J* = 14.4 Hz, 1H), 3.28 (d, *J* = 14.4 Hz, 1H), 3.73 (s, 3H), 7.47 – 7.53 (m, 1H), 7.67 – 7.78 (m, 2H), 7.67 – 7.78 (m, 2H), 7.83 (s, 1H); ^{13}C NMR (101 MHz, $CDCl_3$) δ 26.9, 36.8, 52.6, 58.1, 120.5, 120.7, 128.7, 129.8, 137.2, 144.2, 177.4; HRMS (EI): *m/z* calcd for $C_{13}H_{17}N_4O_2$ $[M+H]^+$: 261.1346; found: 261.1346.

Synthesis of 2-(4-amino-5-methoxy-4-methyl-5-oxopentyl)benzoic (13)

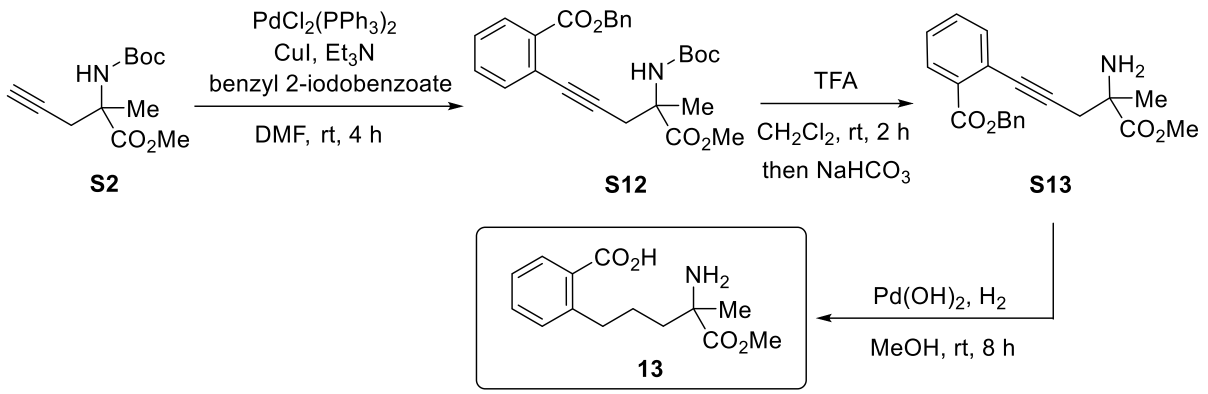

A solution of **S2** (500 mg, 2.1 mmol) in DMF (12 mL) was added over a period of 4 hours to a mixture of $PdCl_2(PPh_3)_2$ (74 mg, 5 mol%), CuI (40 mg, 10 mol%), Et_3N (1.48 mL), and benzyl 2-iodobenzoate (1.08 g, 3.18 mmol) in degassed DMF (25 mL). After 4 hours, the reaction mixture was diluted with EtOAc and washed with saturated aqueous solution of $NaHCO_3$ and brine. The organic layer was dried over Na_2SO_4, filtered and the solvents were removed *in vacuo*. The crude product was purified by flash column chromatography (silica gel; petroleum ether/EtOAc, 4:1) to yield 710 mg of benzyl 2-(4-((*tert*-butoxycarbonyl)amino)-5-methoxy-4-methyl-5-oxopent-1-yn-1-yl)benzoate (**S12**) (75% yield). R_f = 0.36 (petroleum ether/EtOAc, 3:1); mp 74 – 76 °C; ^{1}H NMR (400 MHz, $CDCl_3$) δ 1.43 (s, 9H), 1.61 (s, 3H), 2.93 (d, *J* = 17.1 Hz, 1H), 3.20 (d, *J* = 17.1 Hz, 1H), 3.77 (s, 3H), 5.37 (q, *J* = 12.5 Hz, 2H), 5.72 (br s, 1H), 7.27 – 7.49 (m, 7H), 7.51 (d, *J* = 7.2 Hz, 1H), 7.95 (d, *J* = 7.3 Hz, 1H); ^{13}C NMR (101 MHz, $CDCl_3$) δ 23.4, 28.4, 28.4, 52.7, 58.7, 67.0, 79.9, 82.1, 91.1, 124.3, 127.7, 128.3, 128.4, 128.7, 130.4, 131.8, 131.9, 134.3, 136.0, 154.9, 165.9, 173.9 (two carbon signals overlap at 28.4); HRMS (ESI): *m/z* calcd for $C_{26}H_{29}NNaO_6$ $[M+Na]^+$: 474.1887; found: 474.1877.

TFA (0.80 mL) was added to a solution of **S12** (75 mg, 0.167 mmol) in CH_2Cl_2 (1.6 mL) and after 2 hours stirring the solvent was removed under reduced pressure. The crude product was dissolved in EtOAc and a saturated aqueous solution of $NaHCO_3$ was added and the mixture was stirred for 10 minutes. Then, the organic layer was separated, dried over Na_2SO_4, filtered and concentrated *in vacuo*. The crude product was purified by flash column

chromatography (silica gel; petroleum ether/EtOAc, 1:2) to afford 48 mg benzyl 2-(4-amino-5-methoxy-4-methyl-5-oxopent-1-yn-1-yl)benzoate (**S13**) (82% yield) as a colourless oil. R_f = 0.22 (petroleum ether/EtOAc, 1:2); ^{1}H NMR (400 MHz, $CDCl_3$) δ 1.44 (s, 3H), 2.28 (br s, 2H), 2.68 (d, J = 16.7 Hz, 1H), 2.89 (d, J = 16.7 Hz, 1H), 3.74 (s, 3H), 5.35 (s, 2H), 7.28 – 7.46 (m, 7H), 7.50 (dd, J = 7.7, 0.9 Hz, 1H), 7.93 (dd, J = 7.9, 0.9 Hz, 1H); ^{13}C NMR (101 MHz, $CDCl_3$) δ 26.2, 32.4, 52.6, 58.1, 67.0, 82.3, 91.1, 124.1, 127.7, 128.3, 128.4, 128.7, 130.4, 131.8, 131.8, 134.4, 136.0, 165.8, 176.5; HRMS (ESI): *m/z* calcd for $C_{21}H_{22}NO_4$ $[M+H]^+$: 352.1543; found: 352.1530.

$Pd(OH)_2$ (10% wt, 27 mg, 0.19 mmol) was added to a degassed solution of **S13** (65 mg, 0.19 mmol) in MeOH (3.8 mL) and the mixture was stirred under H_2 atmosphere (1 atm) for 8 hours. Then, the mixture was filtered through celite and the filtrate was concentrated *in vacuo* to afford 40 mg of **13** (80% yield) as a colourless oil without further purification. ^{1}H NMR (400 MHz, MeOD) δ 1.41 – 1.55 (m, 4H), 1.62 – 1.79 (m, 1H), 1.79 – 2.01 (m, 2H), 2.89 – 3.00 (m, 2H), 3.75 (s, 3H), 7.24 (t, J = 7.1 Hz, 2H), 7.40 (t, J = 7.1 Hz, 1H), 7.86 (d, J = 7.2 Hz, 1H); ^{13}C NMR (101 MHz, MeOD) δ 22.6, 26.8, 34.9, 38.2, 53.9, 61.1, 127.4, 131.1, 132.1, 132.2, 133.2, 144.2, 170.8, 172.6; HRMS (ESI): *m/z* calcd for $C_{14}H_{20}NO_4$ $[M+H]^+$: 266.1387; found: 266.1377.

Synthesis of *tert*-butyl (1-hydroxy-2-methylpent-4-yn-2-yl)carbamate (S14)

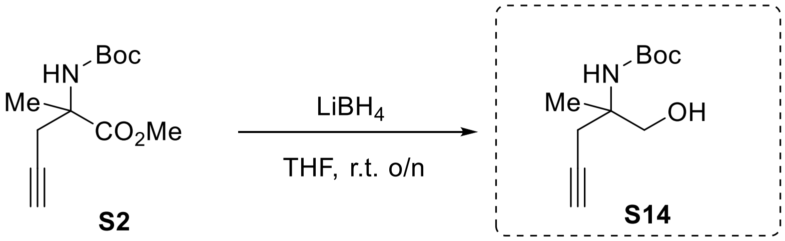

To a solution of **S2** (1.62 g, 6.70 mmol) in THF (30 mL) at 0 ºC was added $LiBH_4$ (438 mg, 20.00 mmol) and the reaction stirred overnight. Then, the reaction mixture was quenched with H_2O and extracted with EtOAc (3x). The combined organics were dried over Na_2SO_4,

filtered and concentrated *in vacuo*. The crude product was purified by flash column chromatography (silica gel; petroleum ether/EtOAc, 3:1) to afford 1.36g of **S14** (96%) as a white solid. R_f = 0.35 (petroleum ether/EtOAc, 4:1); mp 51 – 52 °C; ^{1}H NMR (400 MHz, $CDCl_3$) δ 1.32 (s, 3H), 1.46 (s, 9H), 2.07 (t, *J* = 2.7 Hz, 1H), 2.48 (dd, *J* = 16.8, 2.6 Hz, 1H), 2.72 – 2.78 (m, 1H), 3.70 (s, 2H), 4.90 (s, 1H); ^{13}C NMR (101 MHz, $CDCl_3$) δ 22.6, 26.7, 28.3, 55.9, 69.1, 71.2, 80.1, 80.2, 156.0; HRMS (ESI): *m/z* calcd for $C_{11}H_{18}NO_3$ [M+H]$^+$: 214.1443; found 214.1432.

Synthesis of *tert*-butyl (6-methyl-2-phenyl-3,5,6,7,9,9a-hexahydro-2*H*-oxepino[4,3-*e*][1,2]oxazin-6-yl)carbamate (14)

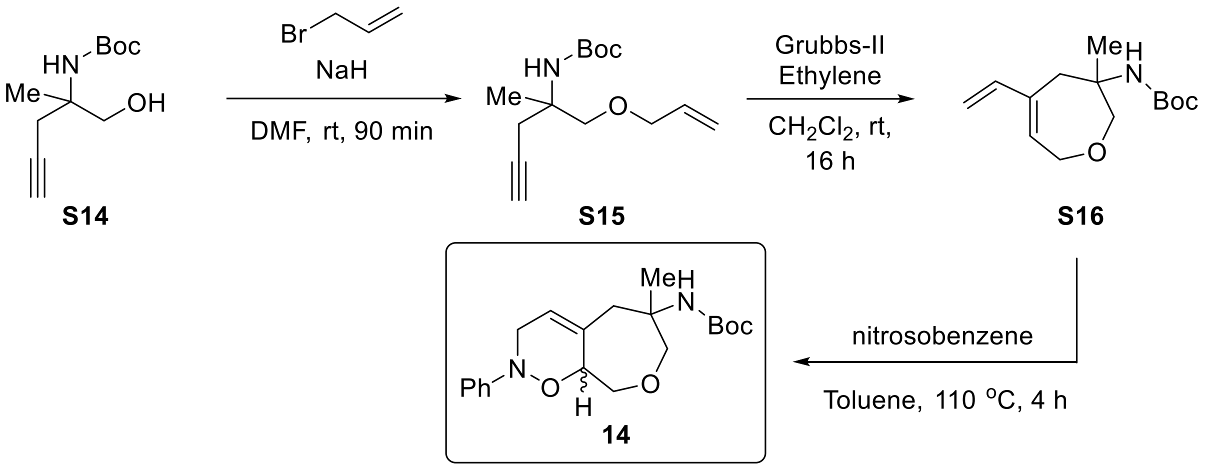

To a solution of **S14** (40 mg, 0.188 mmol) in DMF (2 mL) at 0 °C was added sodium hydride (60% dispersion in mineral oil, 8.2 mg, 0.206 mmol). After 15 minutes, allyl bromide (45.5 uL, 0.376 mmol) was added and the reaction stirred for 90 minutes. Then, the reaction was diluted with H_2O and extracted with EtOAc (2x). The combined organic layers were dried over Na_2SO_4, filtered and concentrated *in vacuo*. The crude product was purified by flash column chromatography (silica gel; petroleum ether/ EtOAc, 3:2) to give 28 mg of *tert*-butyl (1-(allyloxy)-2-methylpent-4-yn-2-yl)carbamate (**S15**) (59%) as a colourless oil. R_f = 0.54 (petroleum ether/EtOAc, 9:1); ^{1}H NMR (400 MHz, $CDCl_3$) δ 1.41 (s, 3H), 1.45 (s, 9H), 2.01 (t, *J* = 2.7 Hz, 1H), 2.59 (dd, *J* = 16.7, 2.7 Hz, 1H), 2.76 – 2.86 (m, 1H), 3.45 (d, *J* = 9.1 Hz,

1H), 3.60 (d, *J* = 9.1 Hz, 1H), 4.03 (d, *J* = 5.6 Hz, 2H), 4.87 (s, 1H), 5.20 (dd, *J* = 10.4, 1.3 Hz, 1H), 5.29 (dt, *J* = 17.2, 1.5 Hz, 1H), 5.91 (ddt, *J* = 16.0, 10.8, 5.6 Hz, 1H); ^{13}C NMR (101 MHz, $CDCl_3$) δ 21.5, 26.3, 28.4, 54.6, 70.5, 72.3, 74.0, 79.2, 80.7, 117.0, 134.6, 154.7; HRMS (ESI): *m/z* calcd for $C_{14}H_{23}NO_3Na$ $[M+H]^+$: 276.1576, found 276.1570.

A solution of **S15** (150 mg, 0.592 mmol) in CH_2Cl_2 (10 mL) was degassed with an argon purge. Grubbs-II (50 mg, 0.0592 mmol) was added the reaction was degassed with ethylene and stirred under this atmosphere for 18 hours. Then, the reaction was concentrated *in vacuo* and purified by flash column chromatography (silica gel; petroleum ether/EtOAc, 19:1) to give 60mg of *tert*-butyl (3-methyl-5-vinyl-2,3,4,7-tetrahydrooxepin-3-yl)carbamate (**S16**) (40%) as a colourless oil. R_f = 0.3 (petroleum ether/EtOAc, 8:1); ^1H (400 MHz, $CDCl_3$) δ 1.37 (s, 3H), 1.38 (s, 9H), 2.39 (d, J = 15.0 Hz, 1H), 3.18 (d, J = 14.0 Hz, 1H), 3.49 (d, J = 12.3 Hz,1H), 3.90 (d, J = 12.3 Hz, 1H), 4.12 (dd, J = 15.2, 4.9 Hz, 1H), 4.28 (dd, J = 15.0, 6.1 Hz, 1H), 4.88 (bs, 1H), 5.04 (d, J = 10.9 Hz, 1H), 5.33 (d, J = 17.4 Hz, 1H), 5.86 (t, J = 5.5 Hz, 1H), 6.31 (dd, J = 17.4, 10.9 Hz, 1H); ^{13}C NMR (101 MHz, $CDCl_3$) δ 23.4, 28.4, 35.5, 52.8, 69.1, 78.9, 81.6, 113.8, 129.9, 139.5, 140.0, 154.9; HRMS (ESI): *m/z* calcd for $C_{14}H_{23}NO_3Na$ $[M+H]^+$: 276.1576, found 276.1585.

To a solution of **S16** (60 mg, mmol) in toluene (2.5 mL) was added nitrosobenzene (51 mg, mmol) and the reaction heated to 110 ºC for 4 hours. Then, the reaction was concentrated under reduced pressure to give a mixture of diastereomers (dr *ca.* 46:36:10:0 determined by ^1H NMR). The major isomer was isolated by flash column chromatography (silica gel; petroleum ether/EtOAc 22:3) to give 26 mg of **14** (31%) as a yellow oil.[4] Data for isolated diastereomer **14**: R_f = 0.15 (petroleum ether/EtOAc 22:3); ^1H NMR (400 MHz, $CDCl_3$) δ 1.29 (s, 3H), 1.44 (s, 9H), 2.62 – 2.75 (m, 2H), 3.27 (d, *J* = 12.7 Hz, 1H), 3.62 (dd, *J* = 11.4, 9.5 Hz, 1H), 3.99 (dd, *J* = 11.4, 6.6 Hz, 2H), 4.23 – 4.31 (m, 2H), 4.48 – 4.56 (m, 1H), 4.76 (s, 1H), 5.70 (d, *J* = 2.2 Hz, 1H), 6.9 – 6.96 (m, 1H), 7.03 (dd, *J* = 8.7, 1.0 Hz, 2H), 7.26 – 7.29

[4] The diastereomeric ratio was determined by ^1H NMR. Only three out of the four possible diastereomers were detected by ^1H NMR.

(m, 2H); ^{13}C (101 MHz, $CDCl_3$) δ 22.3, 28.4, 45.7, 55.1, 61.8, 67.1, 72.0, 78.2, 80.9, 115.7, 121.8, 123.2, 129.0, 133.1, 147.6,154.7; HRMS (ESI): *m/z* calcd for $C_{20}H_{28}N_2O_4Na$ $[M+H]^+$: 383.1941, found 383.1931.

Synthesis of *N*-(1-hydroxy-2-methylpent-4-yn-2-yl)acetamide (S17)

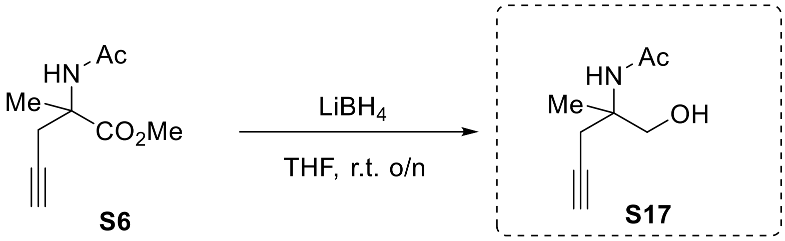

To a solution of **S6** (810 mg, 4.42 mmol) in THF (44 mL) at 0 ºC was added $LiBH_4$ (192 mg, 8.8 mmol) and the reaction stirred for 16 hours. Then, the reaction was quenched with water and extracted with EtOAc (3x). The combined organic extracts were washed with brine, dried over Na_2SO_4, filtered and concentrated *in vacuo* to give 568 mg of **S17** (83%) as a white solid without further purification. R_f = 0.22 (petroleum ether/EtOAc, 4:1); mp 107 – 109 ºC; ^1H NMR (400 MHz, $CDCl_3$) δ 1.35 (s, 3H), 2.05 (s, 3H), 2.10 (t, *J* = 2.6 Hz, 1H), 2.45 (dd, *J* = 16.9, 2.6 Hz, 1H), 2.83 (dd, *J* = 16.9, 2.6 Hz, 1H), 3.69 (d, *J* = 5.6 Hz, 2H), 4.70 (d, *J* = 5.8 Hz, 1H), 5.77 (s, 1H); ^{13}C (101 MHz, $CDCl_3$) δ 22.7, 24.0, 26.7, 57.6, 69.1, 71.5, 80.0, 171.3; HRMS (ESI): *m/z* calcd for $C_8H_{14}NO_2$ $[M+H]^+$: 156.1019, found 156.1019.

Synthesis of N-(4,9-dimethyl-7-phenyl-1,3,4,5-tetrahydrooxepino[3,4-c]pyridin-4-yl)acetamine (15)

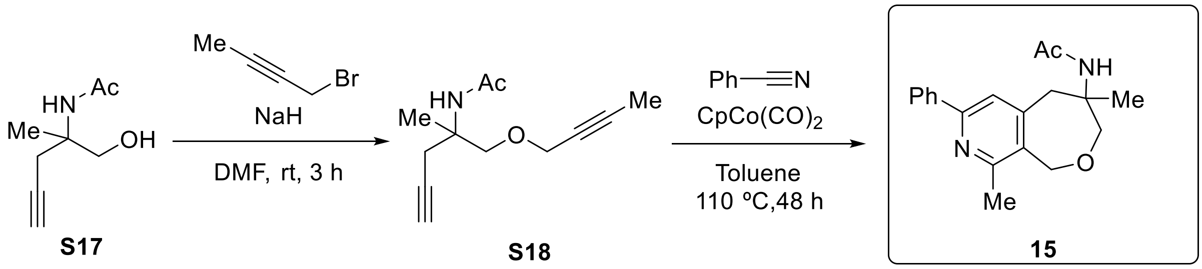

A solution of **S17** (150 mg, 0.965 mmol) in DMF (9.6 mL) was cooled to 0 ºC before sodium hydride (60% dispersion in mineral oil, 42 mg, 1.06 mmol) was added. After 20 minutes 1-bromo-2-butyne (93 uL, 1.06 mmol) was added and the reaction was stirred for 3 hours warming to room temperature. Then, NH_4Cl (sat. aq) was added and the reaction mixture was extracted with EtOAc (3x). The combined organic extracts were washed with brine, dried over Na_2SO_4, filtered and concentrated *in vacuo*. The crude product was purified by flash column chromatography (silica gel; petroleum ether/EtOAc, 3:2) to give 164 mg of *N*-(1-(but-2-yn-1-yloxy)-2-methylpent-4-yn-2-yl)acetamide (**S18**) (82%) as a colourless oil. R_f = 0.22 (petroleum ether/EtOAc, 3:2); ^{1}H (400 MHz, $CDCl_3$) δ 1.47 (s, 3H), 1.88 (t, *J* = 2.3 Hz, 3H), 1.97 (s, 3H), 2.01 (t, *J* = 2.7 Hz, 1H), 2.64 (dd, *J* = 16.7, 2.7 Hz, 1H), 2.94 (dd, *J* = 16.7, 2.7 Hz, 1H), 3.54 (d, *J* = 9.2 Hz, 1H), 3.72 (d, *J* = 9.2 Hz, 1H), 4.16 (m, *J* = 2.3 Hz, 2H), 5.70 (s, 1H); ^{13}C (101 MHz, $CDCl_3$) δ 3.6, 21.1, 24.4, 25.9, 55.4, 59.2, 70.6, 73.5, 74.9, 80.6, 82.9, 170.1. HRMS (ESI): *m/z* calcd for $C_{12}H_{17}NO_2Na$ [M+Na]$^+$: 230.1152, found 230.1146.

$CpCo(CO)_2$ (96 uL, 0.540 mmol) and benzonitrile (104 uL, 1.544 mmol) and) were added to a solution of **S18** (80 mg, 0.386 mmol) in toluene (3.8 mL) previously degassed with an argon purge, and the heated to 110 ºC for 48 hours (in a sealed tube). Them, the reaction was cooled to room temperature and the solvents removed *in vacuo.* The crude product was purified by flash column chromatography (silica gel; petroleum ether/EtOAc, 7:3) 16 mg of **15** (22%) as a white solid (based on 39% recovered unreacted starting material). Data for **15**: R_f = 0.12 (petroleum ether/EtOAc, 1:1); mp 147 –151 ºC; ^{1}H NMR (500 MHz, $CDCl_3$) δ 1.46 (s, 3H), 1.65 (s, 3H), 2.67 (s, 3H), 3.04 (d, *J* = 14.2 Hz, 1H), 3.64 (d, *J* = 12.3 Hz, 1H), 3.79 (dd, *J* = 14.3, 2.0 Hz, 1H), 3.98 (dd, *J* = 12.3, 2.0 Hz, 1H), 4.55 (d, *J* = 14.0 Hz, 1H), 5.13 (d, *J* = 14.0 Hz, 1H), 5.49 (s, 1H), 7.37 – 7.41 (m, 1H), 7.41 – 7.48 (m, 3H), 7.97 (dt, *J* = 8.3, 1.8 Hz, 2H); ^{13}C NMR (126 MHz, $CDCl_3$) δ 22.6, 23.9, 29.7, 43.5, 54.0, 69.8, 83.0, 121.0, 126.9, 128.7, 128.9, 130.4, 139.1, 148.1, 154.8, 156.2, 170.2; HRMS (ESI): *m/z* calcd for $C_{19}H_{23}N_2O_2$ [M+H]$^+$: 311.1760, found 311.1764.

Synthesis of *cis-N*-(4,9-dimethyl-8,10-dioxo-3,4,5,7,7a,8,9,10,10a,10b-decahydro-1*H*-oxepino[3,4-*e*]isoindol-4-yl)acetamide (16)

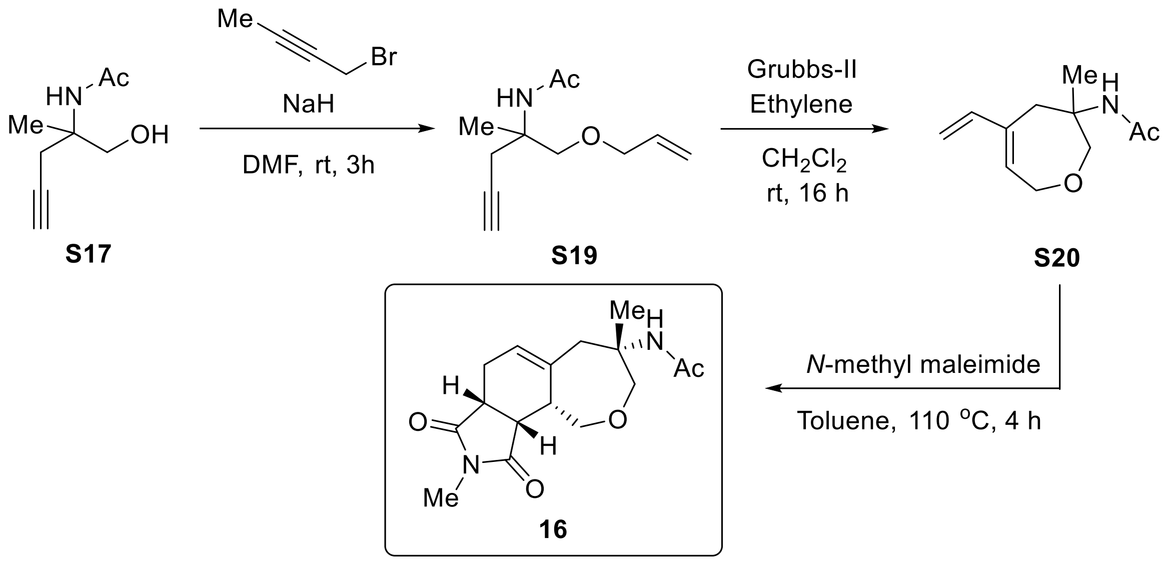

To a solution of **S17** (250 mg, 1.61 mmol) in DMF (17.8 mL) at 0 ºC was added sodium hydride (60% dispersion in mineral oil, 70 mg, 1.77 mmol). After 15 minutes allyl bromide (153 uL, 1.77 mmol) was added and the reaction was stirred for 3 hours. Then, NH_4Cl (sat. aq.) was added and the reaction mixture was extracted with EtOAc (3x). The combined organic extracts were washed with brine, dried over Na_2SO_4, filtered and concentrated *in vacuo*. The crude product was purified by flash column chromatography (silica gel; petroleum ether/EtOAc, 3:2) to give 276 mg of *N*-(1-(allyloxy)-2-methylpent-4-yn-2-yl)acetamide (**S19**) (88%) as a colourless oil. R_f = 0.25 (petroleum ether/EtOAc, 3:2); ^{1}H NMR (400 MHz, $CDCl_3$) δ 1.46 (s, 3H), 1.96 (s, 3H), 2.01 (t, *J* = 2.7 Hz, 1H), 2.64 (dd, *J* = 16.7, 2.7 Hz, 1H), 2.92 (dd, *J* = 16.7, 2.7 Hz, 1H), 3.47 (d, *J* = 9.2 Hz, 1H), 3.66 (d, *J* = 9.2 Hz, 1H), 4.03 (d, *J* = 5.6 Hz, 2H), 5.21 (dd, *J* = 10.4, 1.3 Hz, 1H), 5.29 (dt, *J* = 17.3, 1.5 Hz, 1H), 5.69 (s, 1H), 5.91 (ddt, *J* = 16.0, 10.6, 5.6 Hz, 1H); ^{13}C NMR (101 MHz, $CDCl_3$) δ 21.2, 24.4, 25.9, 55.6, 70.6, 72.3, 73.8, 80.6, 117.2, 134.4, 170.1; HRMS (ESI): *m/z* calcd for $C_{11}H_{18}NO_2$ $[M+H]^+$: 196.1329, found 196.1332.

A solution of **S19** (240 mg, 1.18 mmol) in CH_2Cl_2 (22.8 mL) was degassed with an argon purge for 15 minutes. Grubbs-II (100 mg, 0.118 mmol) was added and the reaction was

degassed with ethylene and stirred under this atmosphere for 18 hours. Then, the reaction was concentrated *in vacuo* and purified by flash column chromatography (silica gel; petroleum ether/EtOAc, 1:1) to give 120 mg of *N*-(3-methyl-5-vinyl-2,3,4,7-tetrahydrooxepin-3-yl)acetamide (**S20**) (52%). R_f = 0.22 (petroleum ether/EtOAc, 2:3); ^{1}H NMR (400 MHz, $CDCl_3$) δ 1.46 (s, 3H), 1.89 (s, 3H), 2.43 (d, *J* = 15.0 Hz, 1H), 3.30 (d, *J* = 15.0 Hz, 1H), 3.52 (d, *J* = 12.4 Hz, 1H), 3.99 (d, *J* = 12.3 Hz, 1H), 4.09 – 4.23 (m, 1H), 4.32 (dd, *J* = 15.2, 6.2 Hz, 1H), 5.09 (d, *J* = 10.8 Hz, 1H), 5.3 – 5.39 (m, 1H), 5.69 (br s, 1H), 5.88 (t, *J* = 5.2 Hz, 1H), 6.33 (dd, *J* = 17.4, 10.8 Hz, 1H); ^{13}C NMR (101 MHz, $CDCl_3$) δ 23.2, 24.3, 35.6, 53.8, 69.1, 81.4, 114.1, 129.7, 139.5, 140.0, 170.2; HRMS (ESI): *m/z* calcd for $C_{11}H_{17}NO_2Na$ $[M+Na]^+$: 218.1152, found 218.1146.

To a solution of **S20** (45 mg, 0.230 mmol) in toluene (2.3 mL) was added *N*-methylmaleimide (51 mg, 4.62 mmol) and the reaction heated to 110 ºC for 5 hours. Then, the reaction was cooled to room temperature and concentrated *in vacuo* to give a mixture of diastereomers (dr = *ca.* 85:15 determined by ^{1}H NMR). The major diastereomer was isolated by flash column chromatography (silica gel, EtOAc) to give 49 mg of **16** (70%) as a white solid. R_f = 0.12 (EtOAc); mp 134 –137 ºC; ^{1}H NMR (400 MHz, $CDCl_3$) δ 1.33 (s, 3H), 1.86 (s, 3H), 2.1 – 2.19 (m, 1H), 2.37 (d, *J* = 14.4 Hz, 1H), 2.57 – 2.64 (m, 1H), 2.68 – 2.75 (m, 1H), 2.92 (s, 3H), 3.02 – 3.08 (m, 2H), 3.16 (t, *J* = 7.7 Hz, 1H), 3.30 (d, *J* = 12.5 Hz, 1H), 3.87 (dd, *J* = 12.5, 2.2 Hz, 1H), 4.18 – 4.3 (m, 2H), 5.53 (s, 1H), 5.71 – 5.78 (m, 1H); ^{13}C NMR (101 MHz, $CDCl_3$) δ 22.0, 24.0, 24.9, 25.0, 40.6, 41.5, 42.7, 43.8, 56.7, 72.6, 81.4, 125.4, 139.6, 169.8, 178.0, 179.5; HRMS (ESI): *m/z* calcd for $C_{16}H_{23}N_2O_4$ $[M+H]^+$: 307.1658, found 307.1663.

Synthesis of *tert*-butyl (3-methyl-5-methylene-2-oxotetrahydrofuran-3-yl)carbamate (17)

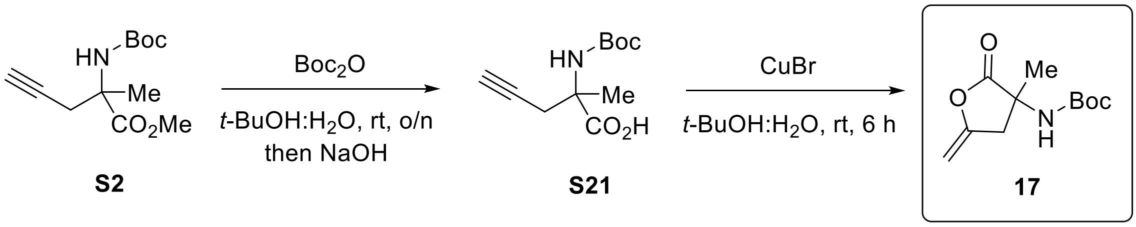

To a stirred solution of **S2** (150 mg, 1.06 mmol) in a mixture 2:1 of THF:H_2O (9 mL), Boc_2O (695 mg, 3.18 mmol) and a solution of 10% aqueous solution of NaOH (1 mL) were added. The mixture was stirred at room temperature overnight and then the organic solvent was removed under reduced pressure. The aqueous layer was washed with EtOAc (3x), acidified with 1N HCl (pH = 2-3) and extracted with EtOAc (3x). The combined organic layers were dried over Na_2SO_4, filtered and concentrated *in vacuo* to yield 186 mg of 2-((*tert*-butoxycarbonyl)amino)-2-methylpent-4-ynoic acid (**S21**) (77% yield) as a white solid without further purification. mp 75 – 77 °C; ^{1}H NMR (500 MHz, $CDCl_3$) δ 1.44 (s, 9H), 1.59 (s, 3H), 2.05 (t, J = 2.3 Hz, 1H), 2.85 (d, J = 11.0 Hz, 1H), 2.99 (d, J = 16.6 Hz, 1H), 5.26 (s, 1H); ^{13}C NMR (126 MHz, $CDCl_3$) δ 23.3, 27.0, 28.4, 58.3, 71.7, 79.2, 80.8, 155.3, 177.2; HRMS (ESI): *m/z* calcd for $C_{11}H_{18}NO_4$ $[M+H]^+$: 228.1230; found: 228.1226.

To a stirred solution of **S21** (24 mg, 0.105 mmol) in a mixture 1:1 of *t*-BuOH:H_2O (2 mL), CuBr (4.5 mg, 10 mol%) was added. The resulting mixture was stirred for 6 hours, diluted with water, and extracted with EtOAc (3x). The organic layers were washed twice with brine, dried over Na_2SO_4, and evaporated under reduced pressure. The crude product was purified by flash column chromatography (silica gel; petroleum ether/EtOAc, 4:1) to afford 22 mg of **17** (92% yield) as a white solid. R_f = 0.32 (petroleum ether/EtOAc, 4:1); mp 119 – 121 °C; ^{1}H NMR (400 MHz, $CDCl_3$) δ 1.43 (s, 9H), 1.46 (s, 3H), 2.77 (dt, J = 16.0, 1.0 Hz, 1H), 3.20 – 3.55 (m, 1H), 4.35 (td, J = 2.5, 1.1 Hz, 1H), 4.77 – 4.80 (m, 1H), 5.04 (s, 1H); ^{13}C NMR (101 MHz, $CDCl_3$) δ 23.6, 28.3, 38.9, 56.8, 81.0, 90.1, 152.4, 154.5, 175.8; HRMS (EI): *m/z* calcd for $C_{11}H_{17}KNO_4$ $[M+K]^+$: 266.0795; found: 266.0791.

Synthesis of *tert*-butyl (3-methyl-3,4-dihydro-2*H*-pyran-3-yl)carbamate (18)

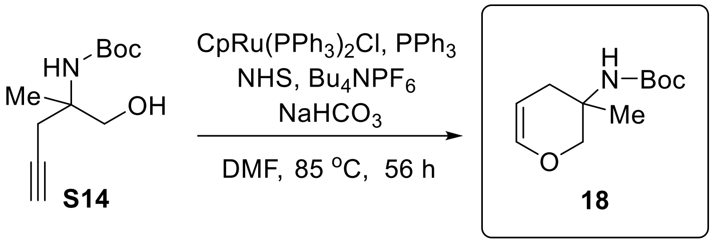

A solution of **S14** (19 mg, 0.0890 mmol), *N*-hydroxysuccinimide (5 mg, 0.0445 mmol), tetrabutylammonium hexafluorophosphonate (17.3 mg, 0.0445 mmol) and $NaHCO_3$ (3.80 mg, 0.00270 mmol) in DMF (1 mL) was degassed with an argon purge for 15 minutes. $CpRu(PPh_3)_2Cl$ (6 mg, 0.024 mmol) and PPh_3 (2.10 mg, 0.008 mmol) were added and the reaction heated to 80 °C for 56 hours (in a sealed tube). Then, the reaction was cooled to room temperature and filtered through celite, washing with EtOAc. The filtrate was washed with brine, dried over Na_2SO_4, filtered and concentrated *in vacuo*. The crude product was purified by flash column chromatography (silica gel; petroleum ether/EtOAc 3:2) to give 9.1 mg of **18** (47%) as a colourless oil. R_f = 0.39 (petroleum ether/EtOAc 19:1); ^{1}H NMR (400 MHz, $CDCl_3$) 1.37 (s, 3H), 1.43 (s, 9H), 1.97 (d, *J* = 17.2 Hz, 1H), 2.28 (d, J = 16.9 Hz, 1H), 3.56 (d, *J* = 11.0 Hz, 1H), 4.04 (d, *J* = 10.16 Hz, 1H), 4.66 – 4.63 (m, 2H), 6.35 (d, *J* = 5.9 Hz, 1H); ^{13}C NMR (101 MHz, $CDCl_3$) 22.8, 28.4, 32.5, 48.6, 70.8, 79.2, 98.6, 143.2, 154.8; HRMS (EI): *m/z* calcd for $C_{11}H_{19}NO_3Na$ $[M+Na]^+$: 236.1257, found 236.1250.

Synthesis of methyl 2-methyl-2-(1*H*-pyrrol-1-yl)pent-4-ynoate (19)

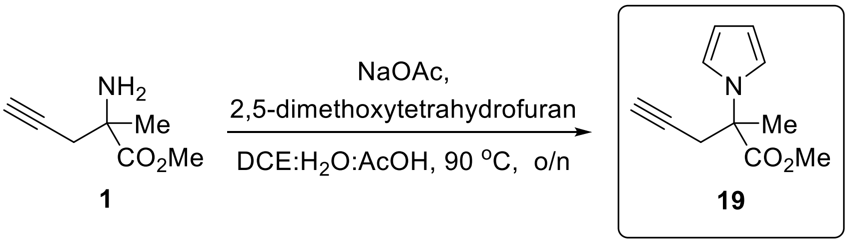

NaOAc (97 mg, 1.19 mmol) was added to a stirred solution of **1** (140 mg, 0.99 mmol) in a mixture 5:3:1 of DCE:H_2O:AcOH (2.24 mL) and the reaction was stirred at 90 °C. After 5 minutes, 2,5-dimethoxytetrahydrofuran (0.13 mL, 0.99 mmol) was added and the mixture was stirred at the same temperature overnight. Then, the reaction was cooled to room temperature, diluted with EtOAc and washed with saturated aqueous solution of NaCl. The

organic layer was dried over Na_2SO_4, filtered and the solvents were concentrated *in vacuo*. The crude product was purified by flash column chromatography (silica gel; petroleum ether/EtOAc, 9:1) to yield 115 mg of **19** (61% yield) as a white solid. R_f = 0.28 (petroleum ether/EtOAc, 9:1); mp 55 – 57 ºC; ^{1}H NMR (400 MHz, CDCl3) δ 1.94 (s, 3H), 2.05 (t, *J* = 2.6 Hz, 1H), 3.00 (dd, *J* = 16.8, 2.6 Hz, 1H), 3.07 (dd, *J* = 16.8, 2.6 Hz, 1H), 3.74 (s, 3H), 6.21 (t, *J* = 2.2 Hz, 2H), 6.83 (t, *J* = 2.2 Hz, 2H); ^{13}C NMR (101 MHz, $CDCl_3$) δ 22.8, 30.0, 53.1, 63.1, 72.3, 78.5, 108.8, 118.7, 172.0; HRMS (ESI): *m/z* calcd for $C_{11}H_{14}NO_2$ [M+H]$^+$: 192.1019; found: 192.1013.

Synthesis of methyl 2-(2-cyano-1*H*-pyrrol-1-yl)-2-methylpent-4-ynoate (20)

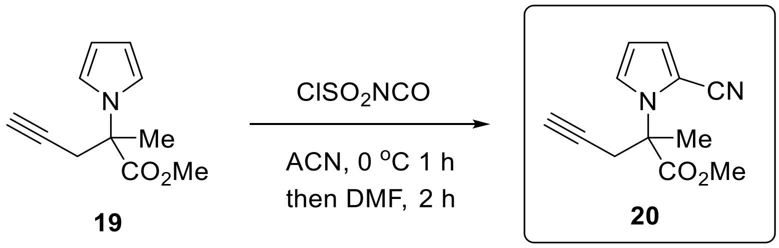

Chlorosulfonyl isocyanate (0.047 mL, 0.55 mmol) was added drop-wise to a solution of **19** (70 mg, 0.37 mmol) in MeCN (3.6 mL) at 0 ºC. After 1 hour stirring, DMF (0.14 mL, 1.85 mmol) was added and the mixture was stirred for 2 hours. Then, saturated aqueous solution of $NaHCO_3$ was added and the reaction mixture was extracted with EtOAc (3x). The combined organic layers were dried over Na_2SO_4, filtered and the solvents were concentrated *in vacuo*. The crude product was purified by flash column chromatography (silica gel; petroleum ether/EtOAc, 4:1) to yield 57 mg of **20** (71% yield) as colourless oil. R_f = 0.29 (petroleum ether/EtOAc; 5:1); mp 64 – 66 ºC; ^{1}H NMR (400 MHz, $CDCl_3$) δ 1.88–2.08 (m, 4H), 3.09 (dd, *J* = 17.5, 2.7 Hz, 1H), 3.31 (dd, *J* = 17.5, 2.1 Hz, 1H), 3.84 (s, 3H), 6.19 (dd, *J* = 3.8, 3.0 Hz, 1H), 6.93 (dd, *J* = 3.9, 1.5 Hz, 1H), 7.07 (dd, *J* = 2.8, 1.6 Hz, 1H); ^{13}C NMR (101 MHz, $CDCl_3$) δ 23.8, 29.5, 53.7, 64.5, 72.8, 77.2, 102.9, 108.5, 114.1, 123.2, 125.5, 171.5; HRMS (ESI): *m/z* calcd for $C_{12}H_{12}N_2NaO_2$ [M+Na]$^+$: 239.0791; found: 239.0783.

Synthesis of 3,4-dimethyl-3-(prop-2-yn-1-yl)-3,4-dihydro-1*H*-benzo[*e*][1,4]diazepine-2,5-dione (21)

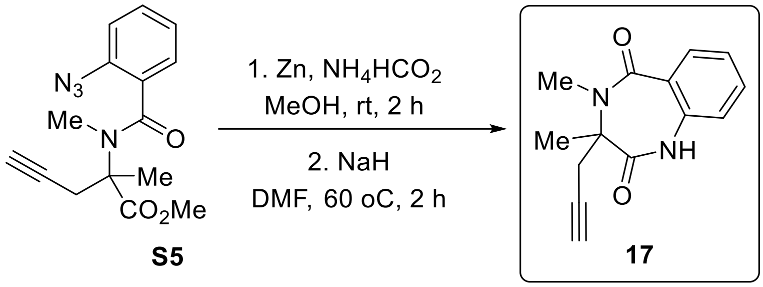

To a stirred solution of **S5** (50 mg, 0.166 mmol) in methanol (1.7 mL) was added zinc (16.3 mg, 0.25 mmol), followed by ammonium formate (32 mg, 0.5 mmol). The resulting mixture was stirred for 2 hours. The reaction mixture was then filtered through a celite pad, washing with CH_2Cl_2. The solvents were removed under reduced pressure and the resulting intermediate was dissolved in DMF (1.7 mL). Sodium hydride (60% in mineral oil, 13 mg, 0.33 mmol) was added to the solution and the mixture was heated at 60 °C for 2 hours. Then, the mixture was diluted with water and the aqueous layer was extracted with EtOAc (3x). The combined organic layers were washed with brine, dried with Na_2SO_4, filtered, and the solvents were evaporated. The crude product was purified by flash column chromatography (silica gel; petroleum ether/EtOAc, gradient from 1:1 to 0:1) to yield 25 mg of **21** (62% yield) as a white solid. R_f = 0.18 (petroleum ether/EtOAc, 1:1); mp 213 – 215 °C; ^{1}H NMR (400 MHz, $CDCl_3$) δ 1.78 (s, 3H), 2.06 (s, 1H), 2.54 (s, 1H), 2.68 (s, 1H), 3.31 (s, 3H), 6.97 (d, *J* = 8.0 Hz, 1H), 7.20 – 7.27 (m, 1H), 7.46 (td, *J* = 8.0, 1.5 Hz, 1H), 7.99 (dd, *J* = 7.9, 1.2 Hz, 1H), 8.79 (br s, 1H); ^{13}C NMR (101 MHz, $CDCl_3$) δ 23.3, 27.7, 32.7, 61.6, 72.8, 119.4, 125.1, 127.6, 131.8, 132.8, 134.6, 168.0, 171.3 (one quaternary carbon coincides with solvent signal); HRMS (EI): *m/z* calcd for $C_{14}H_{15}N_2O_2$ [M+H]$^+$: 243.1128; found: 243.1133.

Synthesis of methyl 2-(3-ethoxy-3-oxopropanamido)-2-methylpent-4-ynoate (22)

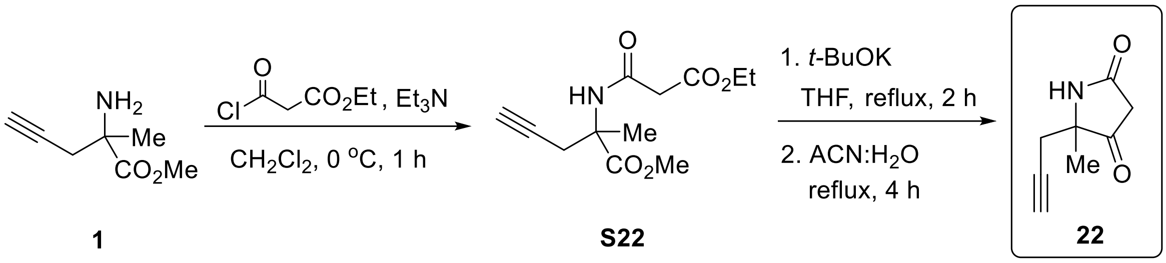

To a solution of **1** (220 mg, 1.56 mmol) in CH_2Cl_2 (15 mL) cooled to 0 ºC, was added ethyl malonyl chloride (0.40 mL, 3.12 mmol) and Et_3N (0.43 mL, 3.12 mmol) and the mixture was stirred for 1 hour. Then, saturated aqueous solution of NH_4Cl was added and the organic layer was separated. The aqueous layer was extracted with CH_2Cl_2 (2x) and the combined organic layers were dried over Na_2SO_4, filtered and concentrated *in vacuo*. The crude product was purified by flash column chromatography (silica gel; petroleum ether/EtOAc; 1:1) to afford 379 mg of methyl 2-(3-ethoxy-3-oxopropanamido)-2-methylpent-4-ynoate (**S22**) (95% yield) as a colourless oil. R_f = 0.34 (petroleum ether/EtOAc, 1:1); ^{1}H NMR (400 MHz, $CDCl_3$) δ 1.25 (t, *J* = 7.1 Hz, 3H), 1.57 (s, 3H), 1.98 (t, *J* = 2.6 Hz, 1H), 2.93 (d, *J* = 2.6 Hz, 2H), 3.27 (s, 2H), 3.72 (s, 3H), 4.18 (q, *J* = 7.1 Hz, 2H), 7.78 (br s, 1H); ^{13}C NMR (101 MHz, $CDCl_3$) δ 14.1, 22.7, 26.4, 41.4, 53.0, 58.7, 61.7, 71.3, 79.2, 164.5, 169.1, 173.1; HRMS (EI): *m/z* calcd for $C_{12}H_{17}NNaO_5$ $[M+Na]^+$: 278.0999; found: 278.0987.

t-BuOK (250 mg, 2.22 mmol) was added to a solution of **S22** (379 mg, 1.48 mmol) in THF (15 mL) and the resulting mixture was heated at reflux for 2 hours. The mixture warmed to room temperature and then diluted with EtOAc (7 mL) and 1N HCl (7 mL). The aqueous layer was extracted with EtOAc (3x) and the combined organic layers were dried over Na_2SO_4, filtered and the solvents concentrated *in vacuo*. The resulting residue was dissolved in a mixture MeCN:H_2O (10:1, 15 mL) heated at reflux for 4 hours. The solvents were evaporated *in vacuo* and the crude product was purified by flash column chromatography (silica gel; petroleum ether/EtOAc, 1:2) to afford 166 mg of **22** (74% yield) as a white solid. R_f = 0.22 (petroleum ether/EtOAc; 1:2); mp 121 – 123 ºC; ^{1}H NMR (400 MHz, $CDCl_3$) δ 1.42 (s, 3H), 2.10 (t, *J* = 2.6 Hz, 1H), 2.54 (qd, *J* = 16.9, 2.6 Hz, 2H), 3.08 (d,

J = 2.3 Hz, 2H), 7.26 (br s, 1H); ^{13}C NMR (101 MHz, $CDCl_3$) δ 23.1, 28.9, 40.6, 67.1, 72.5, 78.0, 170.5, 208.3; HRMS (EI): *m/z* calcd for $C_8H_{10}NO_2$ $[M+H]^+$: 152.0706; found: 152.0704.

Synthesis of *tert*-butyl 2-((1-(2-ethoxy-2-oxoethyl)-1*H*-1,2,3-triazol-5-yl)methyl)-2-methylaziridine-1-carboxylate (23)

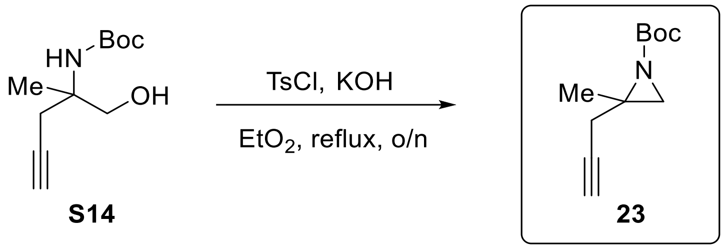

To a solution of **S14** (500 mg, 2.34 mmol) in Et_2O (40 mL), was added TsCl (536 mg, 2.81 mmol) and powdered KOH (1052 mg, 18.4 mmol) and the reaction refluxed overnight. The reaction was cooled to room temperature and diluted with water. The aqueous solution was extracted with Et_2O (2x) and the combined organic layers dried over Na_2SO_4, filtered and concentrated *in vacuo*. The crude product was purified by flash column chromatography (silica gel; petroleum ether/EtOAc, 4:1) to give 357 mg of **23** (78%) as a colourless oil. R_f = 0.55 (petroleum ether/EtOAc 9:1); ^{1}H NMR (400 MHz, $CDCl_3$) δ 1.39 (s, 3H), 1.48 (s, 9H), 2.08 (t, *J* = 2.7 Hz, 1H), 2.12 (s, 1H), 2.25 (s, 1H), 2.37 (dd, *J* = 17.1, 2.7 Hz, 1H), 2.56 (dd, *J* = 17.1, 2.6 Hz, 1H); ^{13}C NMR (101 MHz, $CDCl_3$) δ 19.7, 27.1, 28.0, 36.4, 41.1, 71.0, 79.6, 81.1, 160.6; HRMS (EI): *m/z* calcd for $C_{11}H_{18}NO_2$ $[M+H]^+$: 196.1332, found 196.1333.

Synthesis of 5-methyl-5-(prop-2-yn-1-yl)morpholin-2-one (24)

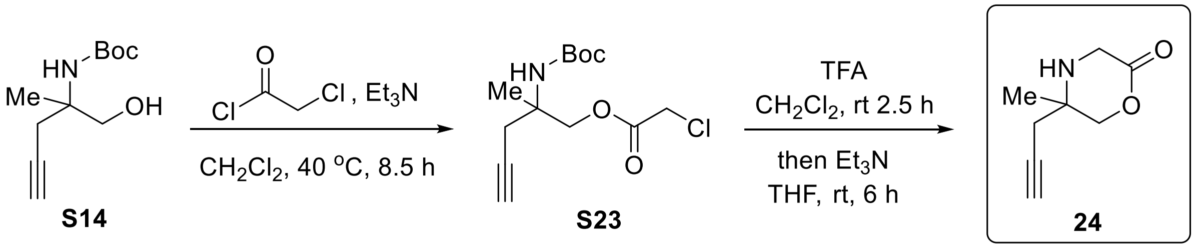

To a solution of **S14** (84.0 mg, 0.394 mmol) and trimethylamine (65 uL, 0.473 mmol) in CH_2Cl_2 (3 mL) at 0 ºC was added chloroacetyl chloride (37.6 uL, 0.473 mmol) and the reaction warmed to room temperature before heating to 40 ºC for 7 hours. Further chloroacetyl chloride (9.40 uL, 0.118 mmol) was added and the reaction heated for a further 1.5 hours. Then, the reaction was cooled to room temperature, diluted with H_2O and extracted with CH_2Cl_2 (3x). The combined organic layers were dried over Na_2SO_4, filtered and concentrated *in vacuo.* The crude product was purified by flash column chromatography (silica gel; petroleum ether/EtOAc 3:2) to give 80 mg of 2-((*tert*-butoxycarbonyl)amino)-2-methylpent-4-yn-1-yl 2-chloroacetate (**S23**) (70%) as a colourless oil. R_f = 0.14 (petroleum ether/EtOAc, 17:3); ^{1}H NMR (400 MHz, $CDCl_3$) δ 1.40 (s, 3H), 1.44 (s, 9H), 2.06 (t, *J* = 2.6 Hz, 1H), 2.57 (dd, *J* = 16.8, 2.6 Hz, 1H), 2.72 – 2.81 (m, 1H), 4.10 (s, 2H), 4.35 (d, *J* = 11.0 Hz, 1H), 4.43 (d, *J* = 11.0 Hz, 1H), 4.70 (s, 1H); ^{13}C NMR (101 MHz, $CDCl_3$) δ 21.9, 26.8, 28.3, 40.7, 53.9, 68.6, 71.6, 79.3, 79.9, 154.3, 166.9; HRMS (EI): *m/z* calcd for $C_{13}H_{21}NO_4Cl$ $[M+H]^+$: 290.1159, found 290.1144.

To a solution of **S23** (24.0 mg, 0.0830 mmol) in CH_2Cl_2 (0.9 mL) was added TFA (0.85 mL) and the reaction stirred for 2.5 hours. Then, the reaction was concentrated *in vacuo* to afford the intermediate as a colourless gum. To a solution of this intermediate (40 mg, 0.138 mmol) in THF (1.3 mL) was added triethylamine (77 uL, 0.553 mmol) and the reaction stirred for 6 hours. The, the reaction was diluted with water and extracted with CH_2Cl_2 (3x). The combined organic layers were washed with NH_4Cl, dried over Na_2SO_4, filtered and concentrated *in vacuo* to give **24** (16 mg, 0.105 mmol, 76%) as a colourless oil. R_f = 0.41 (petroleum ether/EtOAc, 1:1); 1H NMR (400 MHz, $CDCl_3$) δ 1.42 (s, 3H), 2.13 (t, *J* = 2.6 Hz, 1H), 2.51 (dd, *J* = 16.9, 2.6 Hz, 1H), 2.81 (dd, *J* = 16.9, 2.6 Hz, 1H), 3.75 (s, 2H), 4.05 (s, 2H), 6.93 (s, 1H); ^{13}C NMR (101 MHz, $CDCl_3$) δ 22.1, 26.7, 42.8, 57.8, 68.2, 71.9, 79.3, 166.6; HRMS (EI): *m/z* calcd for $C_8H_{12}NO_2$ $[M+H]^+$: 154.0863, found 154.0862.

Synthesis of 5-methyl-5-(prop-2-yn-1-yl)morpholin-3-one (24)

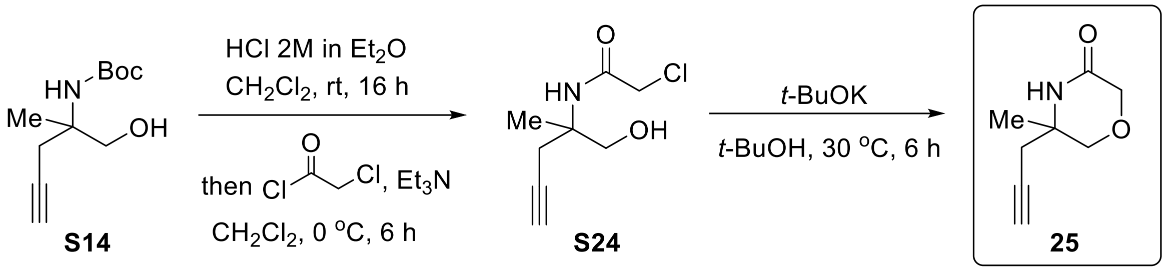

To a solution of **S14** (140 mg, 0.656 mmol) in CH_2Cl_2 (1 mL) was added HCl (2M in Et_2O) (4.28 mL, 0.856 mmol) and the reaction stirred for 22 hours. Then, the reaction was concentrated *in vacuo* to give the intermediate salt. A solution of the intermediate (93 mg) in CH_2Cl_2 (6.5 mL) was cooled to 0 °C before triethylamine (0.260 mL, 1.968 mmol) was added. Chloroacetyl chloride (59 uL, 0.744 mmol) was added drop-wise and the reaction was warmed to room temperature and stirred for 5 hours. Then, a saturated aqueous solution of NH_4Cl (30 mL) was added and extracted with EtOAc (3x). The combined organics were dried over Na_2SO_4, filtered and concentrated *in vacuo*. The crude product was purified by flash column chromatography (silica gel; petroleum ether/EtOAc 7:3) to give 102 mg of 2-chloro-*N*-(1-hydroxy-2-methylpent-4-yn-2-yl)acetamide (**S24**) (88%) as a colourless oil. R_f = 0.21 (petroleum ether/EtOAc 3:2); ^{1}H NMR (400 MHz, $CDCl_3$) 1.39 (s, 3H), 2.10 (t, *J* = 2.7 Hz, 1H), 2.47 (dd, *J* = 16.9, 2.6 Hz, 1H), 2.78 (dd, *J* = 16.9, 2.7 Hz, 1H), 3.72 (s, 2H), 3.94 (s, 1H), 4.03 (s, 2H), 6.90 (br s, 1H); ^{13}C NMR (101 MHz, $CDCl_3$) δ 22.1, 26.8, 42.8, 57.8, 68.2, 72.0, 79.3, 166.6; HRMS (EI): *m/z* calcd for $C_8H_{13}NO_2Cl$ $[M+H]^+$: 190.0629, found 190.0629.

To a solution of **S24** (102 mg, 0.540 mmol) in *t*-BuOH (5.4 mL) was added *t*-BuOK (65 mg, 0.579 mmol) and the reaction heated to 30 °C for 6 hours. Then, NH_4Cl (sat. aq.) was added and the solution extracted with EtOAc (3x). The combined organics were dried over Mg_2SO_4 and concentrated *in vacuo*. The crude product was purified by flash column chromatography (silica gel; petroleum ether/EtOAc 7:3) to give 60 mg of **25** (73%) as a white solid. R_f = 0.09 (petroleum ether/EtOAc 3:2) ; mp 77 – 79°C; ^{1}H NMR (400 MHz, $CDCl_3$) δ 1.36 (s, 3H), 2.13 (t, *J* = 2.6 Hz, 1H), 2.46 (dd, *J* = 16.5, 2.6 Hz, 1H), 2.59 (dd, *J* = 16.5, 2.6 Hz, 1H), 3.55 (d, *J*

= 11.8 Hz, 1H), 3.77 (d, J = 11.8 Hz, 1H), 4.10 – 4.23 (m, 2H), 6.34 (s, 1H); ^{13}C NMR (101 MHz, $CDCl_3$) δ 23.5, 29.8, 53.7, 67.6, 71.8, 72.1, 78.8, 125.0, 168.2; HRMS (EI): *m/z* calcd for $C_8H_{12}NO_2$ [M+H]$^+$: 154.0863, found 154.0863.

Synthesis of 4-methyl-4-(prop-2-yn-1-yl)oxazolidin-2-one (26)

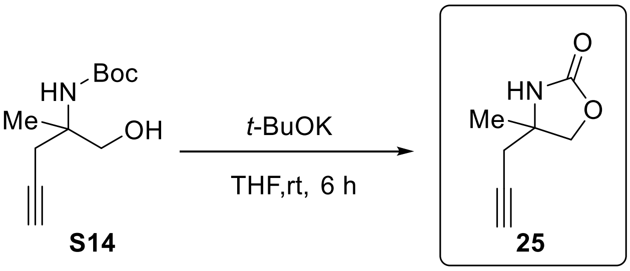

To a of **S14** (33 mg, 0.155 mmol) in THF (1.60 mL) at 0 ºC was added *t*-BuOK (35 mg, 0.31 mmol) and the reaction warmed room temperature and stirred for 6 hours. Then, the reaction was diluted with NH_4Cl saturated aqueous solution and extracted with CH_2Cl_2 (3x). The combined organic extracts were dried over Na_2SO_4, filtered and concentrated *in vacuo* to give 14.7 mg of **26** (67 %) as a white solid without further purification. R_f = 0.26 (petroleum ether/EtOAc, 1:1); m 83 – 85 ºC; ^{1}H NMR (400 MHz, $CDCl_3$) δ 1.48 (s, 3H), 2.12 (t, J = 2.6 Hz, 1H), 2.45 – 2.56 (m, 2H), 4.12 (d, J = 8.7 Hz, 1H), 4.30 (d, J = 8.7 Hz, 1H), 5.95 (s, 1H); ^{13}C NMR (101 MHz, $CDCl_3$); 25.0, 30.9, 57.1, 71.8, 74.9, 78.5, 158.5; HRMS (EI): *m/z* calcd for $C_7H_{10}NO_2$ [M+H]$^+$: 140.0706, found 140.0708.

Synthesis of 3-methyl-5-methylene-3-(1*H*-pyrrol-1-yl)dihydrofuran-2(3*H*)-one (27)

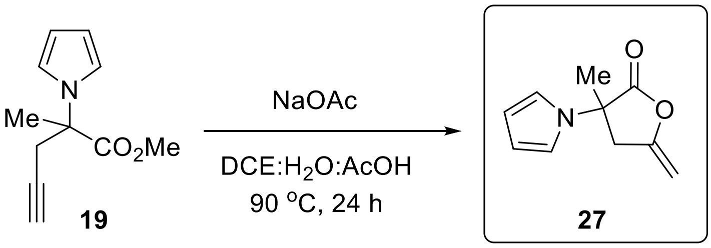

NaOAc (26 mg, 0.32 mmol) was added to a stirred solution of **19** (30 mg, 0.16 mmol) in a mixture 5:3: of DCE:H_2O:AcOH (1.6 mL) and the reaction was stirred at 90 ºC for 24 hours. Then, the reaction was cooled to room temperature, diluted with EtOAc and washed with

saturated aqueous solution of NaCl. The organic layer was dried over Na_2SO_4, filtered and the solvents were concentrated *in vacuo*. The crude product was purified by flash column chromatography to yield 12 mg of **27** (42% yield) as a colourless oil. R_f = 0.22 (petroleum ether/EtOAc, 9:1); ^{1}H NMR (400 MHz, $CDCl_3$) δ1.85 (s, 3H), 3.11 (dt, J = 16.2, 1.5 Hz, 1H), 3.39 (dt, J = 16.2, 1.8 Hz, 1H), 4.51 (dt, J = 3.2, 1.7 Hz, 1H), 4.91 (dt, J = 2.8, 2.1 Hz, 1H), 6.24 (t, J = 2.2 Hz, 2H), 6.86 (t, J = 2.1 Hz, 2H); ^{13}C NMR (101 MHz, $CDCl_3$) δ 24.2, 41.5, 61.6, 91.2, 109.7, 118.4, 151.3, 173.5; HRMS (ESI): *m/z* calcd for $C_{10}H_{12}NO_2$ $[M+H]^+$: 178.0861; found: 178.0863.

Synthesis of methyl 5-methyl-5,6-dihydroindolizine-5-carboxylate (28)

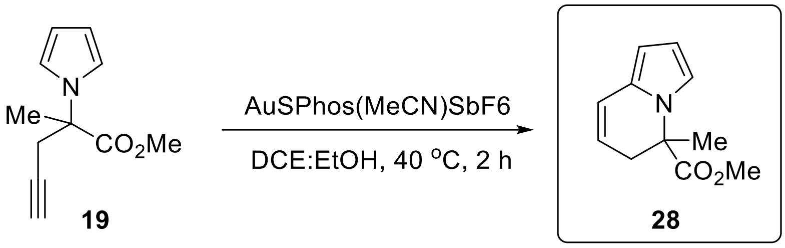

A solution of $AuSPhos(MeCN)SbF_6$ (4.8 mg, 5 mol%) in DCE (0.1 mL) was added drop-wise to a solution of **19** (21 mg, 0.11 mmol) and ethanol (0.032 mL, 0.55 mmol) in DCE (1.0 mL). The resulting solution was heated for 2 hours at 40 °C and then the solvent was removed under reduced pressure. The crude product was purified by flash column chromatography (silica gel; petroleum ether /EtOAc, 4:1) to yield 17 mg of **28** (81% yield) as a colourless oil. R_f = 0.24 (petroleum ether/EtOAc, 5:1); ^{1}H NMR (400 MHz, $CDCl_3$) δ 1.80 (s, 3H), 2.49 (dt, J = 16.9, 2.7 Hz, 1H), 2.95 (dd, J = 16.9, 6.1 Hz, 1H), 3.64 (s, 3H), 5.58 – 5.69 (m, 1H), 6.12 (d, J = 2.5 Hz, 1H), 6.22 (t, J = 3.2 Hz, 1H), 6.46 (dd, J = 9.7, 2.8 Hz, 1H), 6.85 (s, 1H); ^{13}C NMR (101 MHz, $CDCl_3$) δ 24.0, 35.8, 52.9, 61.3, 107.4, 108.9, 117.1, 118.9, 120.8, 130.3, 174.0; HRMS (ESI): *m/z* calcd for $C_{11}H_{14}NO_2$ $[M+H]^+$: 192.1019; found: 192.1012.

Synthesis of methyl 2-cyclopropyl-6-methyl-5,6-dihydropyrrolo[1,2-*h*][1,7]naphthyridine-6-carboxylate (29) and methyl 3-cyclopropyl-6-methyl-5,6-dihydropyrrolo[1,2-*h*][1,7]naphthyridine-6-carboxylate (30)

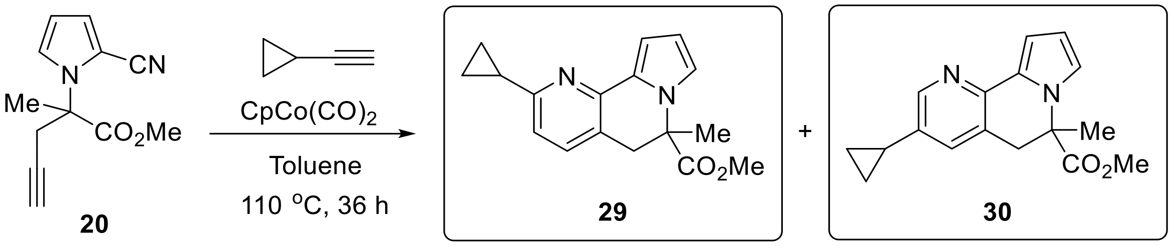

$CpCo(CO)_2$ (13.3 mg, 0.038 mmol) and cyclopropylacetylene (0.048 mL, 0.57 mmol) were added to a solution of **20** (40 mg, 0.19 mmol) in toluene (3.5 mL), previously degassed with argon for 15 minutes, and the mixture was heated at 110 ºC for 36 hours. Then, the solvent was removed under reduced pressure and the crude product was purified by column chromatography (silica gel; gradient from petroleum/EtOAc, gradient from 4:1 to 2:1) to afford 14 mg of **29** (27% yield) and 5 mg of **30** (10% yield) both as a white solids.

Data of major regioisomer (29):

R_f = 0.34 (petroleum ether/EtOAc; 4:1); mp 108 – 110 ºC; ^{1}H NMR (400 MHz, $CDCl_3$) δ 0.94 (dd, *J* = 8.2, 3.3 Hz, 2H), 0.99 – 1.12 (m, 2H), 1.88 (s, 3H), 1.95 – 2.07 (m, 1H), 3.06 (d, *J* = 15.4 Hz, 1H), 3.43 (d, *J* = 15.5 Hz, 1H), 3.54 (s, 3H), 6.33 (t, *J* = 3.2 Hz, 1H), 6.79 (d, *J* = 7.8 Hz, 1H), 6.90 – 6.99 (m, 2H), 7.28 (d, *J* = 7.8 Hz, 1H); ^{13}C NMR (101 MHz, $CDCl_3$) δ 9.7, 17.2, 24.3, 39.4, 53.0, 61.2, 107.9, 110.1, 117.8, 120.0, 120.2, 131.5, 135.1, 146.9, 161.7, 173.1; HRMS (ESI): *m/z* calcd for $C_{17}H_{19}N_2O_2$ $[M+H]^+$: 283.1441; found: 283.1435.

Data of minor regioisomer (30):

R_f = 0.23 (petroleum ether/EtOAc; 2:1); ^{1}H NMR (400 MHz, $CDCl_3$) δ 0.67 – 0.75 (m, 2H), 0.97 – 1.03 (m, 2H), 1.82 – 1.92 (m, 4H), 3.08 (d, *J* = 15.5 Hz, 1H), 3.43 (d, *J* = 15.5 Hz, 1H), 3.54 (s, 3H), 6.35 (t, *J* = 3.0 Hz, 1H), 6.94 (s, 2H), 7.07 (s, 1H), 8.25 (s, 1H); ^{13}C NMR (101 MHz, $CDCl_3$) δ 8.9, 9.0, 13.1, 24.3, 39.8, 53.1, 61.0, 107.6, 110.3, 120.0, 123.5, 131.0,

132.2, 136.4, 145.3, 147.2, 173.0; HRMS (ESI): *m/z* calcd for $C_{17}H_{19}N_2O_2$ $[M+H]^+$: 283.1441; found: 283.1440.

Synthesis of 4-methyl-4-propyl-1,2-dihydropyrrolo[1,2-*a*]pyrazin-3(4*H*)-one (31)

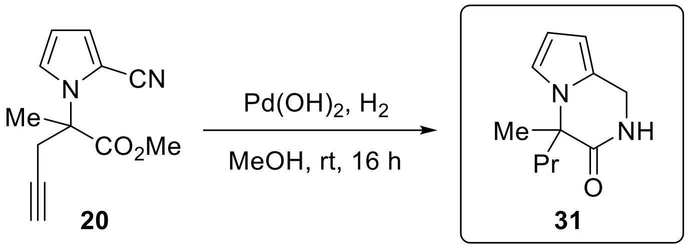

PtO_2 (34 mg, 0.15 mmol) was added to a solution of **20** (35 mg, 0.15 mmol) in MeOH (3 mL) and the reaction mixture was stirred under H_2 atmosphere (1 atm) for 16 h. Then, the resulting suspension was filtered and the filtrate was concentrated under reduced pressure. The crude product was purified by flash column chromatography (silica gel; petroleum ether/EtOAc, 1:1) to yield 10 mg of **31** (35% yield) as colourless oil. R_f = 0.24 (petroleum ether/EtOAc; 1:1); ^1H NMR (400 MHz, $CDCl_3$) δ 0.82 (t, J = 7.2 Hz, 3H), 0.88 – 1.01 (m, 1H), 1.11 – 1.23 (m, 1H), 1.72 (d, J = 4.5 Hz, 3H), 1.82 (ddd, J = 13.6, 10.3, 4.3 Hz, 1H), 2.16 (ddd, J = 13.7, 12.1, 4.6 Hz, 1H), 4.52 – 4.63 (m, 2H), 5.93 (d, J = 2.0 Hz, 1H), 6.25 (t, J = 3.2 Hz, 1H), 6.35 (s, 1H), 6.69 (dd, J = 2.4, 1.8 Hz, 1H); ^{13}C NMR (126 MHz, $CDCl_3$) δ 14.0, 17.4, 26.4, 39.8, 43.7, 62.9, 102.8, 109.8, 115.9, 122.1, 172.2; HRMS (ESI): *m/z* calcd for $C_{11}H_{17}N_2O$ $[M+H]^+$: 193.1335; found: 193.1328.

Synthesis of 8a-methyl-6,8a-dihydro-1*H*-pyrrolo[2,1-*c*][1,4]oxazin-4(3*H*)-one (32)

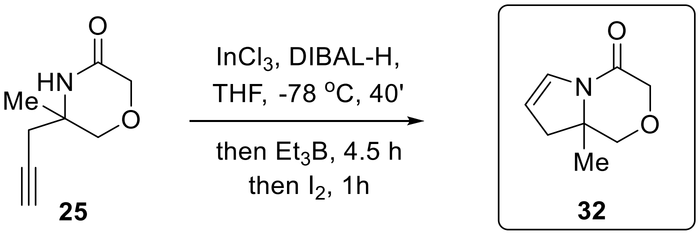

$InCl_3$ (188 mg, 0.852 mmol) was introduced into 10 mL flask and heated with a heat gun (150 °C) under vacuum for 2 min. After being allowed to cool to room temperature, THF (4

mL) was added under Argon. The mixture was stirred at room temperature for 10 min and then cooled to –78 ºC. DIBAL-H (1.0 M in hexane, 0.852 mL, 0.852 mmol) was added drop-wise and the mixture was stirred at –78 ºC for 40 min. Then, **25** (87 mg, 0.570 mmol) in THF (1 mL) was then added, followed by Et_3B (1.0 M in THF, 0.284 mL, 0.284 mmol) and the mixture was stirred at –78 ºC for 4.5 hours. A solution of iodine (865 mg, 3.41 mmol) in THF (1 mL) was then added. After 1 hour, the mixture was poured onto a saturated solution of $NaHCO_3$ (5 mL). $Na_2S_2O_3$ was added under stirring until complete decoloration and the aqueous layer was extracted with EtOAc (5x). The combined organic layers were washed with brine, dried over Na_2SO_4, filtered and concentrated *in vacuo*. The crude sample was purified over a short column (silica gel; petroleum ether/EtOAc, 7:3). The crude product (90 mg), Cs_2CO_3 (121 mg, 0.373 mmol) CuI (24 mg, 0.124 mmol) and *N*,*N*-dimethylethyl-1,2-diamine (27 uL, 0.249 mmol) in toluene (2 mL) were heated to 80 ºC for 3 hours (in a sealed tube). H_2O (10 mL) was added and the reaction extracted with CH_2Cl_2 (5x). The combined organic extracts were washed with brine (10 mL), dried over Na_2SO_4, filtered and concentrated *in vacuo* to give a mixture of desired product and unreacted **25** (*ca.* 50:50 determined by ^{1}H NMR). The crude product was purified by flash column chromatography (silica gel; petroleum ether/EtOAc, 7:3) to give 12 mg of **32** (24%) as a white solid (based on 43% recovered unreacted starting material). R_f = 0.17 (petroleum ether/EtOAc, 7:3); mp 77 – 79 ºC; ^{1}H NMR (400 MHz, $CDCl_3$) δ 1.40 (s, 3H), 2.34 (ddd, *J* = 15.8, 3.1, 1.3 Hz, 1H), 2.58 (dt, *J* = 15.8, 2.5 Hz, 1H), 3.57 (d, *J* = 11.2 Hz, 1H), 3.92 (d, *J* = 11.2 Hz, 1H), 4.08 (d, *J* = 17.1 Hz, 1H), 4.28 (d, *J* = 17.1 Hz, 1H), 5.33 (ddd, *J* = 4.4, 3.1, 2.2 Hz, 1H), 6.83 – 6.88 (m, 1H); ^{13}C NMR (101 MHz, $CDCl_3$) δ 23.9, 40.4, 61.4, 66.0, 72.2, 111.2, 127.2, 163.8; HRMS (ESI): *m/z* calcd for $C_8H_{12}N_2O$ $[M+H]^+$: 154.0868, found 154.0870.

Synthesis of 7a-methyl-5,7a-dihydro-1*H*,3*H*-pyrrolo[1,2-*c*]oxazol-3-one (33)

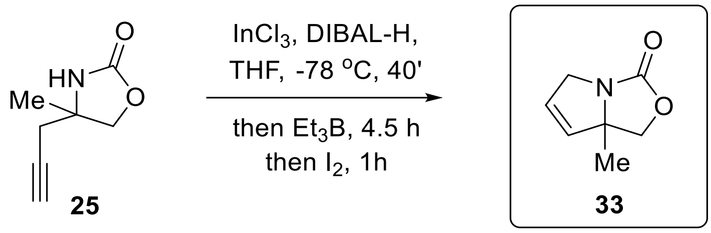

$InCl_3$ (62 mg, 0.28 mmol) was introduced into 5 mL flask and heated with a heat gun (150 ºC) under vacuum for 2 min. After being allowed to cool to room temperature, THF (0.8 mL) was added under Argon. The mixture was stirred at room temperature for 10 min and cooled to –78 ºC. DIBAL-H (1.0 M in hexane, 0.27 mL, 0.27 mmol) was added drop-wise and the mixture was stirred at –78 ºC for 40 min. Then, **26** (25 mg, 0.18 mmol) was then added, followed by Et_3B (1.0 M in THF, 0.09 mL, 0.09 mmol) and the mixture was stirred at –78 ºC for 3 hours. A solution of iodine (274 mg, 1.08 mmol) in THF (0.4 mL) was then added. After 40 min, the mixture was poured onto a saturated solution of $NaHCO_3$ (5 mL). $Na_2S_2O_3$ was added under stirring until complete decoloration and the aqueous layer was extracted with EtOAc (5x). The combined organic layers were dried over Na_2SO_4, filtered and concentrated *in vacuo*. The crude product was dissolved in toluene (1.8 mL) and CuI (15 mg, 0.08 mmol), Cs_2CO_3 (70 mg, 0.22 mmol) and *N,N'*-dimethylethane-1,2-diamine (17 µL, 0.16 mmol) were added. The mixture was stirred at 85 ºC for 2 hours (in a sealed tube). Then, the reaction was cooled to room temperature, diluted with CH_2Cl_2 (5 mL) and washed with water (5 mL). The aqueous layer was extracted with CH_2Cl_2 (3x) and the combined organic layers were dried over Na_2SO_4, filtered and concentrated *in vacuo*. The crude product was purified by flash column chromatography (silica gel; EtOAc/petroleum ether 2:1) to give 7 mg of **33** (28%) as a colourless oil. R_f = 0.14 (petroleum ether/EtOAc, 17:3); ^{1}H NMR (400 MHz, $CDCl_3$) 1.39 (s, 3H), 2.37 (qd, J = 11.5, 2.8 Hz, 1H), 2.71 (dt, J = 16.6, 2.6 Hz, 1H), 4.20 (d, J = 8.6 Hz, 1H), 4.28 (d, J = 8.6 Hz, 1H), 5.23 – 5.21 (m, 1H), 6.38 – 6.35 (m, 1H); ^{13}C NMR (101 MHz, $CDCl_3$) 26.7, 42.8, 64.9, 77.3, 112.6, 127.1, 157.1; HRMS (ESI) *m/z* calcd for $[C_7H_{10}NO_2]^+$: 140.0706, found 140.0712.

Synthesis of methyl 2-methyl-3-(1-phenyl-1*H*-1,2,3-triazol-4-yl)-2-(1*H*-pyrrol-1-yl)propanoate (19a)

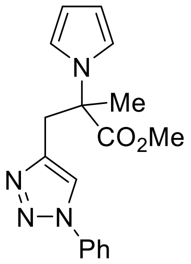

$CuSO_4·5H_2O$ (1.2 mg, 0.041 mmol) and sodium ascorbate (6.2 mg, 0.03 mmol) were added to a stirred solution of **19** (20 mg, 0.105 mmol) and azidobenzene (0.5 M in *tert*-butyl methyl ether, 0.3 mL, 0.156 mmol) in a mixture 1:1 of *t*-BuOH:H_2O (0.5 mL). The mixture was stirred at room temperature overnight. The reaction mixture was concentrated *in vacuo* and the aqueous layer was extracted with EtOAc (3x). The combined organic layer was washed with 5% aqueous solution of NH_4OH, dried over Na_2SO_4, filtered and concentrated *in vacuo*. The crude product was purified by flash column chromatography (silica gel; petroleum ether/EtOAc, 2:1) to yield 30 mg of **19a** (92% yield) as white solid. R_f = 0.34 (petroleum ether/EtOAc, 2:1); mp 100 – 102 ºC; ^1H NMR (400 MHz, $CDCl_3$) δ 1.73 (s, 3H), 3.61 (s, 2H), 3.79 (s, 3H), 6.23 (t, *J* = 2.2 Hz, 2H), 6.60 (s, 1H), 6.78 (t, *J* = 2.2 Hz, 2H), 7.34 – 7.43 (m, 1H), 7.43 – 7.49 (m, 2H), 7.52 – 7.59 (m, 2H); ^{13}C NMR (101 MHz, $CDCl_3$) δ 21.8, 36.3, 53.2, 63.8, 108.9, 119.1, 120.4, 120.8, 128.6, 129.8, 137.1, 142.3, 173.0; HRMS (ESI): *m/z* calcd for $C_{17}H_{18}N_4NaO_2$ $[M+Na]^+$: 333.1327; found: 333.1331.

Synthesis of 3,4-dimethyl-3-((1-phenyl-1*H*-1,2,3-triazol-4-yl)methyl)-3,4-dihydro-1*H*-benzo[*e*][1,4]diazepine-2,5-dione (21a)

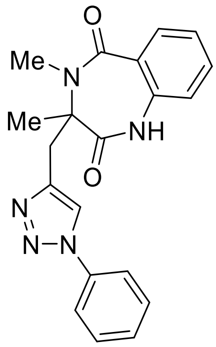

$CuSO_4{\cdot}5H_2O$ (3.4 mg, 0.0135 mmol, 8 mol%) and sodium ascorbate (16 mg, 0.08 mmol) were added to a solution of **21** (41 mg, 0.17 mmol) and azidobenzene (0.5 M in *tert*-butyl methyl ether, 0.54 mL, 0.27 mmol) in a mixture 1:1 of *t*-BuOH:H_2O (0.2 mL). The mixture was stirred at room temperature for 24 hours and then the solvents were removed *in vacuo*. The residue was diluted with brine and then extracted with CH_2Cl_2 (3x). The combined organic layers were washed with 5% aqueous solution of NH_4OH, dried over Na_2SO_4, filtered and solvent was removed *in vacuo*. The crude product was purified by flash column chromatography (silica gel; petroleum ether/EtOAc, gradient from 1:2 to 0:1) to afford 47 mg of **21a** (77% yield) as a white solid. R_f = 0.22 (petroleum ether/EtOAc, 1:2); mp 190 – 192 ºC; ^{1}H NMR (400 MHz, $CDCl_3$) δ 1.79 (br s, 3H), 3.12 (br s, 1H), 3.16 (s, 3H), 3.20 (d, *J* = 14.7 Hz, 1H), 7.02 (d, *J* = 7.9 Hz, 1H), 7.23 – 7.31 (m, 2H), 7.41 – 7.46 (m, 1H), 7.47 – 7.53 (m, 3H), 7.61 (s, 1H), 7.64 – 7.69 (m, 2H), 8.10 (dd, *J* = 7.9, 1.2 Hz, 1H), 8.55 (s, 1H); ^{13}C NMR (101 MHz, $CDCl_3$) δ 23.3, 32.5, 32.8, 62.6, 119.6, 120.6, 120.7, 125.1, 127.6, 129.0, 129.9, 131.8, 133.1, 135.1, 137.0, 142.3, 168.2, 171.9; HRMS (ESI): *m/z* calcd for $C_{20}H_{20}N_5O_2$ $[M+H]^+$: 362.1612; found: 362.1611.

Synthesis of 5-methyl-5-((1-phenyl-1*H*-1,2,3-triazol-4-yl)methyl)pyrrolidine-2,4-dione (22a)

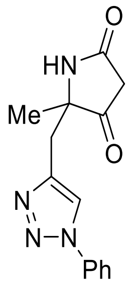

$CuSO_4{\cdot}5H_2O$ (6.7 mg, 0.027 mmol) and sodium ascorbate (16 mg, 0.08 mmol) were added to a solution of **22** (41 mg, 0.27 mmol) and azidobenzene (0.5 M in *tert*-butyl methyl ether, 0.54 mL, 0.27 mmol) in a mixture 1:1 of *t*-BuOH:H_2O (0.2 mL). The mixture was stirred for 24 hours and then solvents were removed *in vacuo*. The residue was dissolved in brine and then extracted with CH_2Cl_2 (3x). The combined organic layers were washed with 5% aqueous solution of NH_4OH, dried over Na_2SO_4, filtered and solvent was removed *in vacuo*. The crude product was purified by flash column chromatography (silica gel; EtOAc/MeOH, gradient from 0:1 to 25:1) to afford 56 mg of **22a** (77% yield) as a white solid. R_f = 0.31 (EtOAc/MeOH, 25:1); mp 188 – 190 ºC; ^1H NMR (400 MHz, $CDCl_3$) δ 1.46 (s, 3H), 2.87 (d, J = 22.2 Hz, 1H), 3.04 (d, J = 22.2 Hz, 1H), 3.13 (d, J = 14.9 Hz, 1H), 3.22 (d, J = 14.9 Hz, 1H), 7.41 (t, J = 7.3 Hz, 1H), 7.48 (t, J = 7.6 Hz, 2H), 7.66 (d, J = 7.8 Hz, 2H), 7.77 (br s, 1H), 7.86 (s, 1H); ^{13}C NMR (101 MHz, $CDCl_3$) δ 23.9, 33.9, 40.4, 67.9, 120.4, 121.0, 129.0, 129.9, 136.9, 142.5, 170.1, 209.8; HRMS (EI): *m/z* calcd for $C_{14}H_{15}N_4O_2$ $[M+H]^+$: 271.1195; found: 271.1192.

Synthesis of 4-methyl-4-((1-phenyl-1*H*-1,2,3-triazol-4-yl)methyl)oxazolidin-2-one (26a)

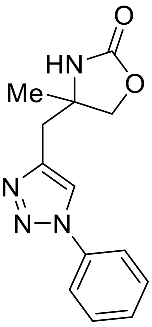

$CuSO_4{\cdot}5H_2O$ (6.7 mg, 0.027 mmol) and sodium ascorbate (16 mg, 0.08 mmol) were added to a solution of **26** (20.0 mg, 0.140 mmol) and azidobenzene (0.5 M in *tert*-butyl methyl ether, 0.431 mL, 0.215 mmol) in a mixture 1:1 of *t*-BuOH:H_2O (1 mL). The mixture was stirred for 24 hours and then solvents were removed *in vacuo*. The residue was dissolved in brine and then extracted with CH_2Cl_2 (3x). The combined organic layers were washed with 5% aqueous solution of NH_4OH, dried over Na_2SO_4, filtered and solvent was removed *in vacuo*. The crude product was purified by flash column chromatography (100% EtOAc) to give 26 mg of **26a** (72%) as a white solid. R_f = 0.12 (petroleum ether/EtOAc 1:1); mp 147.3 – 146.7 ºC; ^{1}H NMR (400 MHz, $CDCl_3$) 1.41 (s, 3H), 3.10 – 3.02 (m, 2H), 4.11 (d, *J* = 8.6 Hz, 1H), 6.97 (s, 1H), 4.40 (d, *J* = 8.6 Hz, 1H), 7.40 – 7.36 (m, 1H), 7.48 – 7.43 (m, 2H), 7.71 – 7.67 (m, 2H), 7.99 (s, 1H); ^{13}C NMR (101 MHz, $CDCl_3$) 26.0, 36.3, 57.7, 75.1, 120.4, 121.1, 128.7, 129.7, 136.9, 142.9, 159.3; HRMS (ESI) *m/z* calcd for $[C_{13}H_{15}N_4O_2]^+$: 259.1190, found 259.1196.

Synthesis of 3,4-dimethyl-3-(3-phenylprop-2-yn-1-yl)-3,4-dihydro-1*H*-benzo[*e*][1,4]diazepine-2,5-dione (21b)

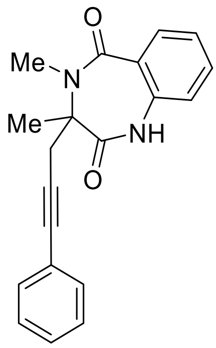

A solution of **21** (25 mg, 0.1 mmol) in DMF (1.2 mL) was added over a period of 1 hour to a mixture of $PdCl_2(PPh_3)_2$ (3.5 mg, 5 mmol%), CuI (1.9 mg, 10 mmol%), Et_3N (0.15 mL), and benzyl 2-iodobenzoate (0.016 mL, 0.15 mmol) in degassed DMF (2.5 mL). After 16 hours, the reaction mixture was diluted with EtOAc and washed with saturated aqueous solution of $NaHCO_3$ and brine. The organic layer was dried over Na_2SO_4, filtered and the solvents were removed *in vacuo*. The crude product was purified by flash column chromatography (silica gel; petroleum ether/EtOAc, 2:1) to yield 19 mg of **21b** (60 % yield) as a white solid. R_f = 0.34 (petroleum ether/EtOAc, 2:1); mp 225 – 227 ºC; ^1H NMR (400 MHz, $CDCl_3$) δ 1.86 (br s, 3H), 2.75 (br s, 1H), 2.90 (br s, 1H), 3.38 (s, 3H), 6.99 (d, J = 8.0 Hz, 1H), 7.21 – 7.34 (m, 4H), 7.34 – 7.43 (m, 2H), 7.48 (t, J = 7.5 Hz, 1H), 8.04 (d, J = 7.8 Hz, 1H), 8.67 (s, 1H); ^{13}C NMR (101 MHz, $CDCl_3$) δ 23.7, 28.8, 32.7, 62.0, 83.4, 84.7, 119.4, 122.9, 125.1, 127.6, 128.4, 128.4, 131.7, 131.9, 132.8, 134.7, 168.1, 171.6; HRMS (ESI): *m/z* calcd for $C_{20}H_{18}N_2NaO_2$ $[M+Na]^+$: 341.1260; found: 341.1253.

Synthesis of 5-methyl-5-(3-phenylprop-2-yn-1-yl)pyrrolidine-2,4-dione (22b)

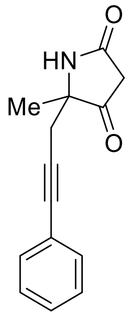

A solution of **22** (25 mg, 0.165 mmol) in DMF (1.2 mL) was added over a period of 1 hour to a mixture of $PdCl_2(PPh_3)_2$ (3.5 mg, 0.005 mmol), CuI (1.9 mg, 0.01 mmol), Et_3N (0.15 mL), and benzyl 2-iodobenzoate (0.016 mL, 0.15 mmol) in degassed DMF (2.5 mL). After 16 hours, the reaction mixture was diluted with EtOAc and washed with saturated aqueous solution of $NaHCO_3$ and brine. The organic layer was dried over Na_2SO_4, filtered and the solvents were removed *in vacuo*. The crude product was purified by flash column chromatography (silica gel; petroleum ether/EtOAc, 2:1) to yield 10 mg of **22b** (26% yield) as

a white solid. R_f = 0.29 (petroleum ether/EtOAc, 2:1); mp 93 – 95 ºC; ^{1}H NMR (400 MHz, $CDCl_3$) δ 1.48 (s, 3H), 2.73 (d, *J* = 16.9 Hz, 1H), 2.82 (d, *J* = 16.9 Hz, 1H), 3.02 – 3.19 (m, 2H), 7.24 (s, 1H), 7.26 – 7.35 (m, 3H), 7.35 – 7.43 (m, 2H); ^{13}C NMR (101 MHz, $CDCl_3$) δ 23.1, 30.0, 40.6, 67.4, 83.2, 84.5, 122.5, 128.5, 128.6, 131.8, 170.4, 208.5; HRMS (EI): *m/z* calcd for $C_{14}H_{14}NO_2$ $[M+H]^+$: 228.1025; found: 228.1019.

Synthesis of 3,4-dimethyl-3-propyl-3,4-dihydro-1*H*-benzo[*e*][1,4]diazepine-2,5-dione (21c)

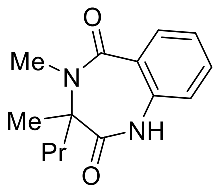

Pd/C (10% wt, 4.4 mg, 10 mol %) was added to a solution of **21** (10 mg, 0.041 mmol) in MeOH (1 mL) and the mixture was stirred under H_2 atmosphere (1 atm) for 2 hours. Then, the mixture was filtered through celite and the filtrate was concentrated *in vacuo*. The crude product was purified by flash column chromatography (silica gel; EtOAc/MeOH, 20:1) to yield 7 mg of **21c** (69% yield) as a white solid. R_f = 0.30 (EtOAc/MeOH, 20:1); mp 156 – 158 ºC; ^{1}H NMR (400 MHz, $CDCl_3$) δ 0.64 (t, *J* = 7.2 Hz, 3H). 1.17 – 1.28 (m, 2H), 1.39 – 1.49 (m, 1H), 1.55 – 1.63 (m, 1H), 1.68 (s, 3H), 3.24 (s, 3H), 6.91 (d, *J* = 8.0 Hz, 1H), 7.21 (t, *J* = 7.6 Hz, 1H), 7.44 (t, *J* = 7.6 Hz, 1H), 7.96 (d, *J* = 7.9 Hz, 1H), 8.38 (s, 1H); ^{13}C NMR (101 MHz, $CDCl_3$) δ 14.1, 17.4, 23.1, 32.6, 38.4, 62.9, 119.1, 124.8, 127.8, 131.4, 132.5, 134.9, 168.3, 173.3; HRMS (EI): *m/z* calcd for $C_{14}H_{18}KN_2O_2$ $[M+K]^+$: 285.1005; found: 285.0989.

Synthesis of 5-methyl-5-propylpyrrolidine-2,4-dione (22c)

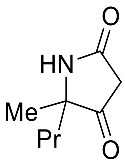

Pd/C (10% wt, 15.5 mg, 10 mol %) was added to a solution of **22** (22 mg, 0.15 mmol) in MeOH (3 mL) and the mixture was stirred under H_2 atmosphere (1 atm) for 2 hours. Then, the mixture was filtered through celite and the filtrate was concentrated *in vacuo*. The crude product was purified by flash column chromatography (silica gel; petroleum ether/EtOAc, 2:1) to afford 20 mg of **22c** (87% yield) as a white solid. R_f = 0.29 (petroleum ether/EtOAc, 1:2); mp 94 – 96 ºC; ^{1}H NMR (400 MHz, $CDCl_3$) δ 0.91 (t, J = 7.3 Hz, 3H), 1.12 – 1.22 (m, 1H), 1.34 (s, 3H), 1.39 – 1.51 (m, 1H), 1.56 (ddd, J = 13.9, 12.4, 4.4 Hz, 1H), 1.73 (ddd, J = 13.9, 12.2, 4.8 Hz, 1H), 2.97 (d, J = 22.2 Hz, 1H), 3.04 (d, J = 22.2 Hz, 1H), 7.17 (br s, 1H); ^{13}C NMR (101 MHz, $CDCl_3$) δ 14.2, 17.3, 24.0, 40.6, 40.7, 68.6, 170.6, 210.3; HRMS (EI): *m/z* calcd for $C_8H_{13}NNaO_2$ $[M+Na]^+$: 178.0844; found: 178.0845.

Synthesis of *tert*-butyl 2-((1-(2-ethoxy-2-oxoethyl)-1*H*-1,2,3-triazol-5-yl)methyl)-2-methylaziridine-1-carboxylate (23d)

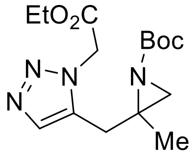

A solution of **23** (50.0 mg, 0.256 mmol) and ethyl 2-azidoacetate (49.6 mg, 0.384 mmol) in toluene (3 mL) was degassed with an argon purge. Cp*Ru(COD)Cl (3.90 mg, 0.0102 mmol) was added at room temperature for 18 hours and then heated to 60 ºC for 3 hours. Further Cp*Ru(COD)Cl (7.80 mg, 0.0204 mmol) was added and the reaction stirred at the same temperature for 24 hours. Then, the reaction was concentrated *in vacuo* and the crude product purified by flash column chromatography (silica gel; petroleum ether/EtOAc, gradient from 1:1 to 0:1) to yield 35 mg of **23d** (42%) as a brown oil. R_f = 0.12 (petroleum ether/EtOAc, 1:1); ^{1}H NMR (400 MHz, $CDCl_3$) δ 1.27 (s, 3H), 1.30 (t, J = 7.1 Hz, 3H), 1.44 (s, 9H), 2.03 (s, 1H), 2.19 (s, 1H), 2.75 (d, J = 15.5 Hz, 1H), 3.03 (d, J = 15.5 Hz, 1H), 4.25 (qd, J = 7.1, 1.5 Hz, 2H), 5.28 (d, J = 17.8 Hz, 1H), 5.43 (d, J = 17.8 Hz, 1H), 7.63 (s, 1H); ^{13}C NMR (101 MHz, $CDCl_3$) δ 14.1, 19.0, 28.0, 31.1, 35.9, 41.3, 49.4, 62.1, 81.7, 133.6,

134.0, 160.2, 166.8; HRMS (EI): *m/z* calcd for $C_{15}H_{25}N_4O_4$ $[M+H]^+$: 325.1876, found 325.1871.

SYNTHESIS OF THE PHENYL-CONTAINING DERIVATIVE 10'

Synthesis of methyl 2-amino-2-phenylpent-4-ynoate (1')

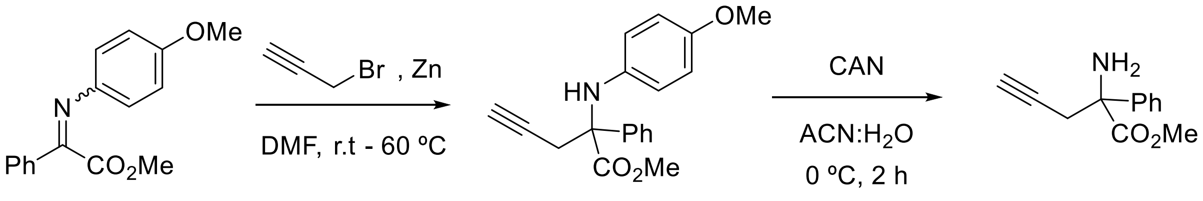

S25 **1'**

Propargyl bromide (80 wt. % in toluene, 1.16 mL, 12.25 mmol) and activated zinc powder (801 mg, 12.25 mmol) were added to a stirred solution of methyl 2-((4-methoxyphenyl)imino)-2-phenylacetate[5] (2.2 g, 8.16 mmol) in DMF (40 mL) cooled at 0 ºC. The reaction mixture was then slowly warmed to room temperature and heated at 60 ºC for 1 hour. Then, the mixture was cooled to 0 °C, quenched with saturated aqueous solution of NH_4Cl and the aqueous layer was extracted with EtOAc (3x). The combined organic layers were washed with brine, dried with Na_2SO_4, filtered, and the solvents were evaporated *in vacuo*. The crude product was purified by flash column chromatography (silica gel; petroleum ether/EtOAc, 9:1) to afford 1.6 g of methyl 2-((4-methoxyphenyl)amino)-2-phenylpent-4-ynoate (**S25**) (64% yield) as a yellow oil. R_f = 0.23 (petroleum ether/EtOAc, 9:1); ^{1}H NMR (400 MHz, $CDCl_3$) δ 2.02 (t, *J* = 2.6 Hz, 1H), 3.21 (dd, *J* = 16.4, 2.6 Hz, 1H), 3.39 (dd, *J* = 16.4, 2.6 Hz, 1H), 3.69 (s, 3H), 3.72 (s, 3H), 4.97 (s, 1H), 6.36 – 6.46 (m, 2H), 6.58 – 6.70 (m, 2H), 7.30 – 7.43 (m, 3H), 7.52 – 7.62 (m, 2H); ^{13}C NMR (101 MHz, $CDCl_3$) δ 25.0, 53.3, 55.6, 66.9, 71.9, 79.4, 114.5, 118.8, 127.0, 128.2, 128.9, 137.8, 139.7, 153.3, 173.1; HRMS (ESI): *m/z* calcd for $C_{19}H_{20}NO_3$ $[M+H]^+$: 310.1443; found: 310.1430.

[5] G. Shang, Q. Yang, X. Zhang, *Angew. Chem. Int. Ed*. **2006**, *45*, 6360.

A solution of CAN (3.78 g, 6.9 mmol) in water (22 mL) was added drop-wise over 15 minutes to a stirred solution of **S25** (1.067 g, 3.45 mmol) in MeCN (22 mL) cooled to 0 ºC. After 2 hours stirring, the resulting solution was treated with 2N HCl to achieve pH 1. The aqueous phase was washed with EtOAc (3x) and basified by the addition of K_2CO_3. The resulting suspension was extracted with CH_2Cl_2 (3x), dried over Na_2SO_4, filtered, and the solvents were evaporated under reduced pressure to yield 450 mg of **1’** (64% yield) as a colourless oil without further purification. R_f = 0.32 (petroleum ether/EtOAc, 2:1); ^{1}H NMR (400 MHz, $CDCl_3$) δ 2.06 (t, J = 2.6 Hz, 1H), 2.18 (br s, 2H), 2.77 (dd, J = 16.4, 2.6 Hz, 1H), 3.14 (dd, J = 16.4, 2.6 Hz, 1H), 3.76 (s, 3H), 7.27 – 7.33 (m, 1H), 7.33 – 7.40 (m, 2H), 7.48 – 7.55 (m, 2H); ^{13}C NMR (101 MHz, $CDCl_3$) δ 31.1, 53.0, 63.5, 71.6, 80.0, 125.4, 128.1, 128.7, 141.5, 174.9; HRMS (EI): *m/z* calcd for $[C_{12}H_{14}NO_2]^+$: 204.1021; found: 204.1025.

Synthesis of methyl 2-((*tert*-butoxycarbonyl)amino)-2-phenylpent-4-ynoate (S2’)

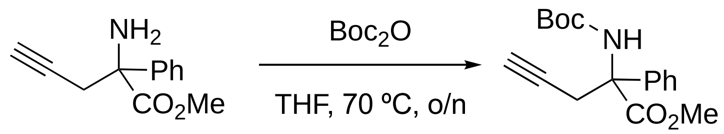

1’ **S2’**

Boc_2O (642 mg, 2.94 mmol) was added to a solution of **1’** (200 mg, 0.98 mmol) in THF (10 mL) and the resulting mixture was stirred at 70 ºC overnight (in a sealed tube). Then, the solvent was evaporated under reduced pressure and the crude product was purified by column chromatography (silica gel; petroleum ether/EtOAc, 4:1) to yield 249 mg of **S2’** (84% yield) as a white solid. R_f = 0.22 (petroleum ether/EtOAc, 8:1); mp 100 – 102 ºC; ^{1}H NMR (400 MHz, $CDCl_3$) δ 1.41 (br s, 9H), 1.99 (s, 1H), 3.45 (dd, J = 16.2, 1.7 Hz, 1H), 3.57 (br s, 1H), 3.71 (s, 3H), 6.04 (s, 1H), 7.27 – 7.40 (m, 3H), 7.41 – 7.46 (m, 2H); ^{13}C NMR (101 MHz, $CDCl_3$) δ 25.3, 28.4, 53.5, 64.5, 71.3, 79.6, 80.2, 125.9, 128.3, 128.7, 138.5, 154.1, 171.9; HRMS (EI): *m/z* calcd for $[C_{17}H_{21}NO_4Na]^+$: 326.1368; found 326.1365.

Synthesis of methyl 7-oxo-5-phenyl-5,6,7,8-tetrahydro-4*H*-[1,2,3]triazolo[1,5-*d*][1,4]diazepine-5-carboxylate (10')

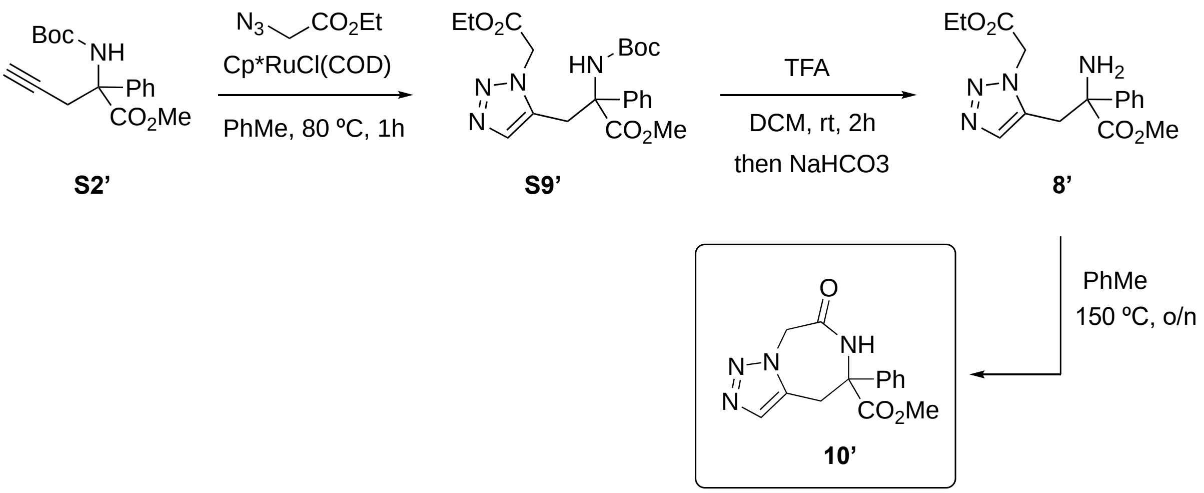

A solution of **S2'** (100 mg, 0.32 mmol) and ethyl 2-azidoacetate (62 mg, 0.48 mmol) in toluene (1.6 mL) was degassed for 15 minutes with argon. Cp*RuCl(COD) (2.3 mg, 0.006 mmol) was added and the mixture was stirred at 80 ºC for 1 hour. Then, the solvent was removed under reduced pressure and the crude product was purified by flash column chromatography (silica gel; petroleum ether//EtOAc, 1:1) to yield 120 mg of methyl 2-((*tert*-butoxycarbonyl)amino)-3-(1-(2-ethoxy-2-oxoethyl)-1*H*-1,2,3-triazol-5-yl)-2-phenylpropanoate (**S9'**) (87% yield) as a colourless oil. R_f = 0.18 (petroleum ether/EtOAc; 2:1); ^{1}H NMR (400 MHz, $CDCl_3$) δ 1.25 (t, *J* = 7.1 Hz, 3H), 1.38 (s, 9H), 3.74 (s, 3H), 3.86 (d, *J* = 15.0 Hz, 1H), 4.02 (d, *J* = 15.2 Hz, 1H), 4.11 – 4.28 (m, 2H), 4.84 (d, *J* = 17.7 Hz, 1H), 5.09 (d, *J* = 17.7 Hz, 1H), 5.85 (s, 1H), 7.19 (s, 1H), 7.28 – 7.41 (m, 5H); ^{13}C NMR (101 MHz, $CDCl_3$) δ 14.1, 28.2, 29.2, 48.7, 53.6, 62.3, 65.1, 80.9, 125.3, 128.7, 129.1, 133.5, 133.6, 138.4, 154.6, 166.6, 171.8; HRMS (EI): *m/z* calcd for $C_{21}H_{28}N_4NaO_6$ $[M+Na]^+$: 455.1907; found: 455.1901.

TFA (0.6 mL) was added to a solution of **S9'** (70 mg, 0.16 mmol) in CH_2Cl_2 (1 mL) and after 2 hours stirring the solvent was removed under reduced pressure. The crude product was dissolved in EtOAc and a saturated aqueous solution of $NaHCO_3$ was added and the mixture was stirred for 10 minutes. Then, the organic layer was separated, dried over Na_2SO_4,

filtered and concentrated *in vacuo* to afford 53 mg methyl 2-amino-3-(1-(2-ethoxy-2-oxoethyl)-1*H*-1,2,3-triazol-5-yl)-2-phenylpropanoate (**8’**) (quantitative yield) as a colourless oil without further purification. R_f = 0.28 (petroleum ether:EtOAc; 1:2); ^{1}H NMR (400 MHz, $CDCl_3$) δ 1.26 (t, *J* = 7.1 Hz, 3H), 1.95 (s, 2H), 3.19 (d, *J* = 15.0 Hz, 1H), 3.51 (d, *J* = 15.0 Hz, 1H), 3.72 (s, 3H), 4.21 (q, *J* = 7.1 Hz, 2H), 5.08 (d, *J* = 17.6 Hz, 1H), 5.16 (d, *J* = 17.6 Hz, 1H), 7.29 – 7.40 (m, 4H), 7.40 – 7.46 (m, 2H); ^{13}C NMR (101 MHz, $CDCl_3$) δ 14.2, 34.4, 49.2, 53.0, 62.3, 64.3, 125.1, 128.5, 129.0, 133.6, 134.4, 141.6, 166.8, 174.6; HRMS (EI): *m/z* calcd for $C_{16}H_{21}N_4O_4$ $[M+H]^+$: 333,1563; found: 333.1560.

A stirred solution of **8’** (38 mg, 0.11 mmol) in toluene (3.8 mL) was heated at 150 ºC overnight. Then, the solvent was removed under reduced pressure and the crude product was purified by flash column chromatography (silica gel; EtOAc) to afford 24 mg of **10’** (76% yield) as a colourless oil. R_f = 0.29 (EtOAc); ^{1}H NMR (400 MHz, $CDCl_3$) δ 3.62 (d, *J* = 16.6 Hz, 1H), 3.78 (s, 3H), 4.20 (d, *J* = 16.6 Hz, 1H), 4.61 (d, *J* = 16.7 Hz, 1H), 5.20 (d, *J* = 16.7 Hz, 1H), 7.27 – 7.37 (m, 5H), 7.40 (br s, 1H), 7.48 (s, 1H); ^{13}C NMR (101 MHz, $CDCl_3$) δ 31.0, 53.3, 54.4, 64.3, 125.6, 129.5, 129.7, 131.3, 134.1. 137.2, 165.1, 170.4; HRMS (ESI): *m/z* calcd for $C_{14}H_{15}N_4O_3$ $[M+H]^+$: 287.1139; found: 287.1135.

SYNTHESIS OF THE OPTICALLY PURE DERIVATIVE (*R*)-22

Synthesis of methyl (*R*)-2-amino-2-methylpent-4-ynoate ((*R*)-1)

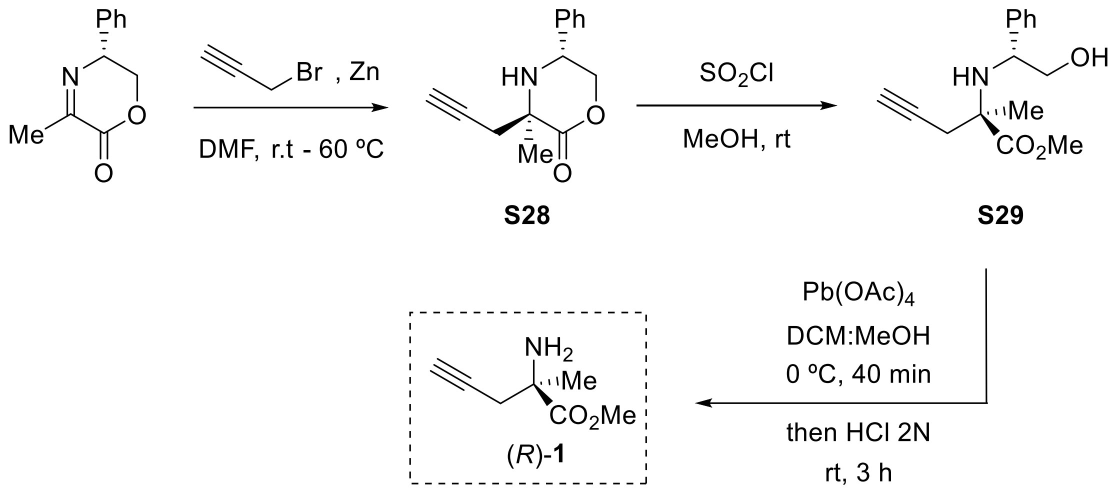

Propargyl bromide (0.62 mL, 8.11 mmol) and activated zinc powder (530 mg, 8.11 mmol) were added to a solution of (*R*)-3-methyl-5-phenyl-5,6-dihydro-2*H*-1,4-oxazin-2-one[6] (1.03 g, 5.41 mmol) in DMF (27 mL) and the mixture was stirred for 1 hour and at 60 ºC for 2 hours. Then, the mixture was cooled to 0 °C and hydrolysed with a saturated aqueous solution of NH_4Cl. The aqueous layer was extracted with EtOAc (3x), washed with brine, dried over Na_2SO_4, filtered, and the solvents were evaporated under reduced pressure. The crude product was purified by flash column chromatography (silica gel; petroleum ether/EtOAc, 8:1) to afford 930 mg of (3*R*,5*R*)-3-methyl-5-phenyl-3-(prop-2-yn-1-yl)morpholin-2-one (**S28**)[7] (75% yield) as an inseparable mixture of regioisomers (mixture of **S28** and inconsequential allene, ca. 82:18 ratio as determined by ^{1}H NMR). R_f = 0.18 (petroleum ether/EtOAc, 8:1); $[\alpha]^{25}_D$ +42.4 (c 0.6, $CHCl_3$); spectroscopic data of the major regioisomer (**S28**): ^{1}H NMR (400 MHz, $CDCl_3$) δ1.59 (s, 3H), 2.11 (t, *J* = 2.6 Hz, 1H), 2.37 (br s, 1H), 2.65 (dd, *J* = 16.9, 2.6 Hz, 1H), 3.12 (dd, *J* = 16.9, 2.5 Hz, 1H), 4.30 – 4.36 (m, 3H), 7.38 – 7.48 (m, 3H), 7.45 – 7.49 (m, 2H); ^{13}C NMR (101 MHz, CDCl3) δ 26.7, 28.7, 53.2, 59.0, 73.0, 75.4, 78.5, 127.4,

---

[6] L. M. Harwood, K. J. Vines, M. G. B. Drew, *Synlett* **1996**, 1051.
[7] Some examples on the use of chiral iminolactones derived from phenylglycinol in diastereoselective syntheses: a) L. M. Harwood, S. N. G. Tyler, G. B. Drew, A. Jahans, *Arkivoc* **2000**, 820; b) S. Fustero, N. Mateu, A. Simón-Fuentes, J. L. Aceña, *Org. Lett.* **2010**, *12*, 3014; c) S. Fustero, N. Mateu, L. Albert, J. L. Aceña, *J. Org. Chem.* **2009**, *74*, 4429.

128.8, 129.0, 137.7, 172.2; HRMS (ESI): *m/z* calcd for $C_{14}H_{16}NO_2$ $[M+H]^+$: 230.1176; found: 230.1173.

SO_2Cl (0.6 mL, 8.1 mmol) was added drop-wise to a solution of **S28** (930 mg, 4.05 mmol) in MeOH (40 mL). The mixture was stirred for 2 hours and then the solvent was removed under reduced pressure. The resulting residue was dissolved in EtOAc and a saturated aqueous solution of $NaHCO_3$. The mixture was stirred for 15 minutes and then the aqueous layer was separated and extracted with EtOAc (2x). The combined organic layers were dried over Na_2SO_4, filtered, and the solvents were evaporated under reduced pressure. The crude product was purified by flash column chromatography (silica gel; petroleum ether/EtOAc, 2:1) to afford 887 mg of methyl (*R*)-2-(((*R*)-2-hydroxy-1-phenylethyl)amino)-2-methylpent-4-ynoate (**S29**) (84% yield) as a colourless oil. $[\alpha]^{25}_D$ –72.8 (c 0.9, $CHCl_3$); ^1H NMR (400 MHz, $CDCl_3$) δ 1.40 (s, 3H), 1.99 (t, *J* = 2.6 Hz, 1H), 2.29 (s, 1H), 2.49 (qd, *J* = 16.6, 2.6 Hz, 2H), 3.02 (s, 1H), 3.37 (s, 3H), 3.38 – 3.48 (m, 1H), 3.56 (dd, *J* = 10.9, 4.4 Hz, 1H), 3.82 (dd, *J* = 9.3, 4.7 Hz, 1H), 7.20 – 7.27 (m, 3H), 7.27 – 7.34(m, 2H); ^{13}C NMR (101 MHz, $CDCl_3$) δ 22.6, 30.0, 52.1, 59.8, 60.9, 67.0, 71.4, 79.9, 127.2, 127.7, 128.6, 141.1, 174.9; HRMS (ESI): *m/z* calcd for $C_{15}H_{19}NNaO_3$ $[M+Na]^+$: 284.1257; found: 284.1246.

$Pb(OAc)_4$ (1.184 g, 2.67 mmol) was added to a solution of **S29** (500 mg, 1.91 mmol) in a mixture 2:1 of CH_2Cl_2:MeOH (18 mL) at 0 ºC. The reaction mixture was vigorously stirred for 1 hour at 0 ºC, and then 20 mL of 2M HCl were added. The reaction was warmed to room temperature and after 2 hours of additional stirring the reaction mixture was filtered through a pad of SiO_2, eluting with MeOH. The organic solvents were removed under reduced pressure and the aqueous residue was washed with EtOAc (3x) and basified by the addition of Na_2CO_3. The resulting suspension was extracted with EtOAc (3x), dried over Na_2SO_4, filtered and the solvents were evaporated under reduced pressure to yield 232 mg of (*R*)-**1** (85% yield) as a colourless oil without further purification. $[\alpha]^{25}_D$ +16.1 (*c* 0.2, $CHCl_3$) (lit. data: $[\alpha]^{25}_D$ +2.08 (*c* 0.8, ethanol).[7] The spectroscopic data are in agreement with those

previously reported in the literature.[2] HRMS (ESI): *m/z* calcd for $C_7H_{12}NO_2$ [M+H]$^+$: 142.0863; found: 142.0866.

Synthesis of methyl (*R*)-2-((*tert*-butoxycarbonyl)amino)-2-methylpent-4-ynoate ((*R*)-S2)

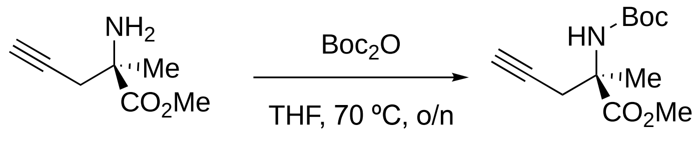

(*R*)-**1** (*R*)-**S2**

Boc_2O (478 mg, 2.19 mmol) was added to a solution of (*R*)-**1** (155 mg, 1.1 mmol) in THF (5.5 mL) and the resulting mixture was stirred at 70 ºC overnight (in a sealed tube). Then, the solvent was evaporated under reduced pressure and the crude product was purified by column chromatography (silica gel; petroleum ether/EtOAc, 4:1) to yield 238 mg of (*R*)-**S2** (90% yield) as a white solid. $[\alpha]^{25}_D$ +34.1 (*c* 0.7, $CHCl_3$) (lit. data for (*S*)-*N*-Boc-α-propargylalanine methyl ester: $[\alpha]^{25}_D$ –63 (*c* 0.10, THF). The spectroscopic data are in agreement with those previously reported in the literature for the (*S*)-*N*-Boc-α-propargylalanine methyl ester.[3] HRMS (ESI): *m/z* calcd for $C_{12}H_{20}NO_4$ [M+H]$^+$: 242.1392; found: 242.1382.

Synthesis of methyl (*R*)-5-methyl-7-oxo-5,6,7,8-tetrahydro-4*H*-[1,2,3]triazolo[1,5-*d*][1,4]diazepine-5-carboxylate ((*R*)-10)

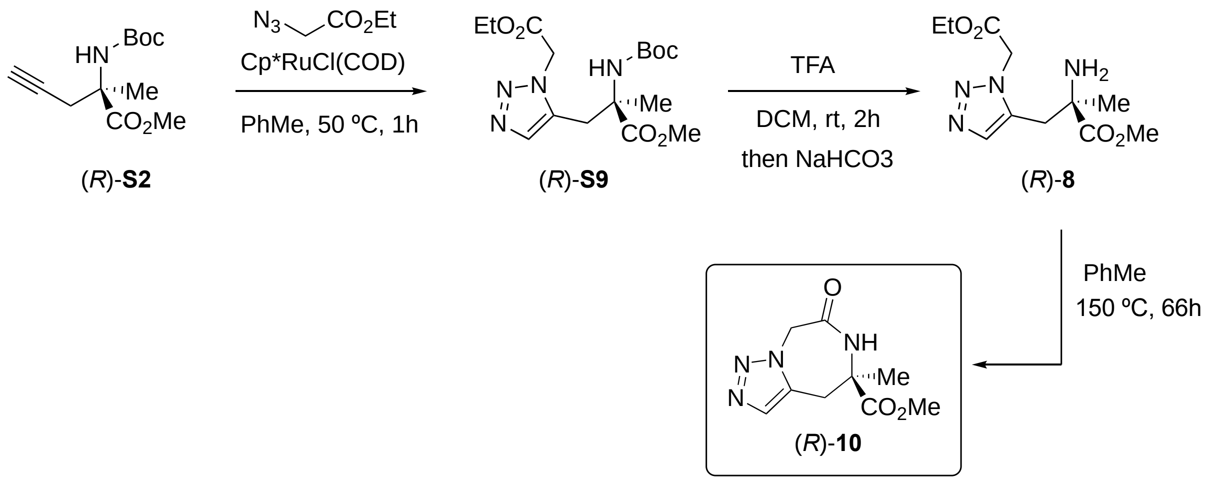

A solution of (*R*)-**S2** (100 g, 0.41 mmol) and ethyl 2-azidoacetate (80 mg, 0.62 mmol) in toluene (2.4 mL) was degassed for 15 minutes with argon. Cp*RuCl(COD) (3.8 mg, 0.01 mmol) was added and the mixture was stirred at 50 ºC for 1 hour. Then, the solvent was removed under reduced pressure and the crude product was purified by flash column chromatography (silica gel; petroleum ether/EtOAc, gradient from 1:1 to 1:2) to yield 116 mg of ((*R*)-2-((*tert*-butoxycarbonyl)amino)-3-(1-(2-ethoxy-2-oxoethyl)-1*H*-1,2,3-triazol-5-yl)-2-methylpropanoate ((*R*)-**S9**) (76% yield) as a colourless oil. R_f = 0.25 (petroleum ether/EtOAc, 1:1); $[\alpha]^{25}_{D}$ +7.3 (*c* 0.5, $CHCl_3$); ^{1}H NMR (400 MHz, $CDCl_3$) δ 1.25 (t, *J* = 7.1 Hz, 3H), 1.40 (s, 9H), 3.38 (d, *J* = 15.5 Hz, 1H), 3.51 (d, *J* = 15.4 Hz, 1H), 3.76 (s, 3H), 4.20 (q, *J* = 7.1 Hz, 2H), 5.12 (s, 2H), 5.30 (s, 1H), 7.40 (s, 1H); ^{13}C NMR (101 MHz, $CDCl_3$) δ 14.1, 24.2, 28.3, 29.3, 48.8, 53.2, 59.2, 62.3, 80.6, 133.5, 133.8, 154.7, 166.6, 173.7; HRMS (ESI): *m/z* calcd for $C_{16}H_{27}N_4O_6$ $[M+H]^+$: 371.1925; found: 371.1921.

TFA (1 mL) was added to a solution of (*R*)-**S9** (136 mg, 0.37 mmol) in CH_2Cl_2 (2 mL) and after 2 h stirring the solvent was removed under reduced pressure. The crude product was dissolved in EtOAc (2 mL) and a saturated aqueous solution of $NaHCO_3$ (2 mL) was added and the mixture was stirred for 10 minutes. Then, the organic layer was separated and the aqueous layer extracted with EtOAc (2x). The combined organic layers were dried over Na_2SO_4, filtered and concentrated *in vacuo* to afford 92 mg methyl (*R*)-2-amino-3-(1-(2-ethoxy-2-oxoethyl)-1*H*-1,2,3-triazol-5-yl)-2-methylpropanoate ((*R*)-**8**) (93% yield) as a colourless without further purification. R_f = 0.22 (EtOAc); $[\alpha]^{25}_{D}$ +9.2 (*c* 1.0, $CHCl_3$); ^{1}H NMR (400 MHz, $CDCl_3$) δ 1.27 (t, *J* = 7.1 Hz, 3H), 1.38 (s, 3H), 1.69 (s, 2H), 2.83 (d, *J* = 14.9 Hz, 1H), 3.16 (d, *J* = 14.9 Hz, 1H), 3.72 (s, 3H), 4.22 (q, *J* = 7.1 Hz, 2H), 5.29 (d, *J* = 17.6 Hz, 1H), 5.35 (d, *J* = 17.6 Hz, 1H), 7.49 (s, 1H); ^{13}C NMR (101 MHz, $CDCl_3$) δ 14.2, 27.0, 34.1, 49.5, 52.8, 58.3, 62.3, 133.9, 133.9, 166.9, 176.7; HRMS (EI): *m/z* calcd for $C_{11}H_{19}N_4O_4$ $[M+H]^+$: 271.1401; found: 271.1396.

A stirred solution of (*R*)-**8** (48 mg, 0.17 mmol) in toluene (3 mL) was heated at 150º C for 66 hours. Then, the solvent was removed under reduced pressure and the crude product was purified by flash column chromatography (silica gel; EtOAc 100%) to afford 30 mg of (*R*)-**10** (79% yield) as a white solid. R_f = 0.18 (EtOAc 100%); $[\alpha]^{25}_{D}$ –12.9 (*c* 0.6, $CHCl_3$); mp 147 – 149 ºC; ^1H NMR (400 MHz, $CDCl_3$) δ 1.54 (s, 3H), 3.34 (d, *J* = 15.8 Hz, 1H), 3.56 (d, *J* = 15.8 Hz, 1H), 3.70 (s, 3H), 5.28 (d, *J* = 0.6 Hz, 2H), 7.54 (s, 1H), 6.83 (br s, 1H); ^{13}C NMR (101 MHz, $CDCl_3$) δ 27.4, 31.0, 52.7, 53.7, 60.7, 131.9, 133.7, 165.0, 172.1; HRMS (EI): *m/z* calcd for $C_9H_{13}N_4O_3$ $[M+H]^+$: 225.0982; found: 225.0982.

STEREOCHEMICAL AND STRUCTURAL ASSIGNMENTS

Compounds 2 and 3

The position of the phenyl substituent present in **2** and **3** was established by means of nOe studies. Thereby, in the nOe spectrum of compound **2** the aromatic proton (H_a) at the α -position to the nitrogen showed an interaction with the methylene group ($2H_c$) adjacent to the NH (Figure S3). As expected, the nOe spectrum of **3** showed an interaction between the aromatic proton (H_a) at the α-position to the nitrogen and the methylene group ($2H_b$) adjacent to the quaternary stereocenter (Figure S4).

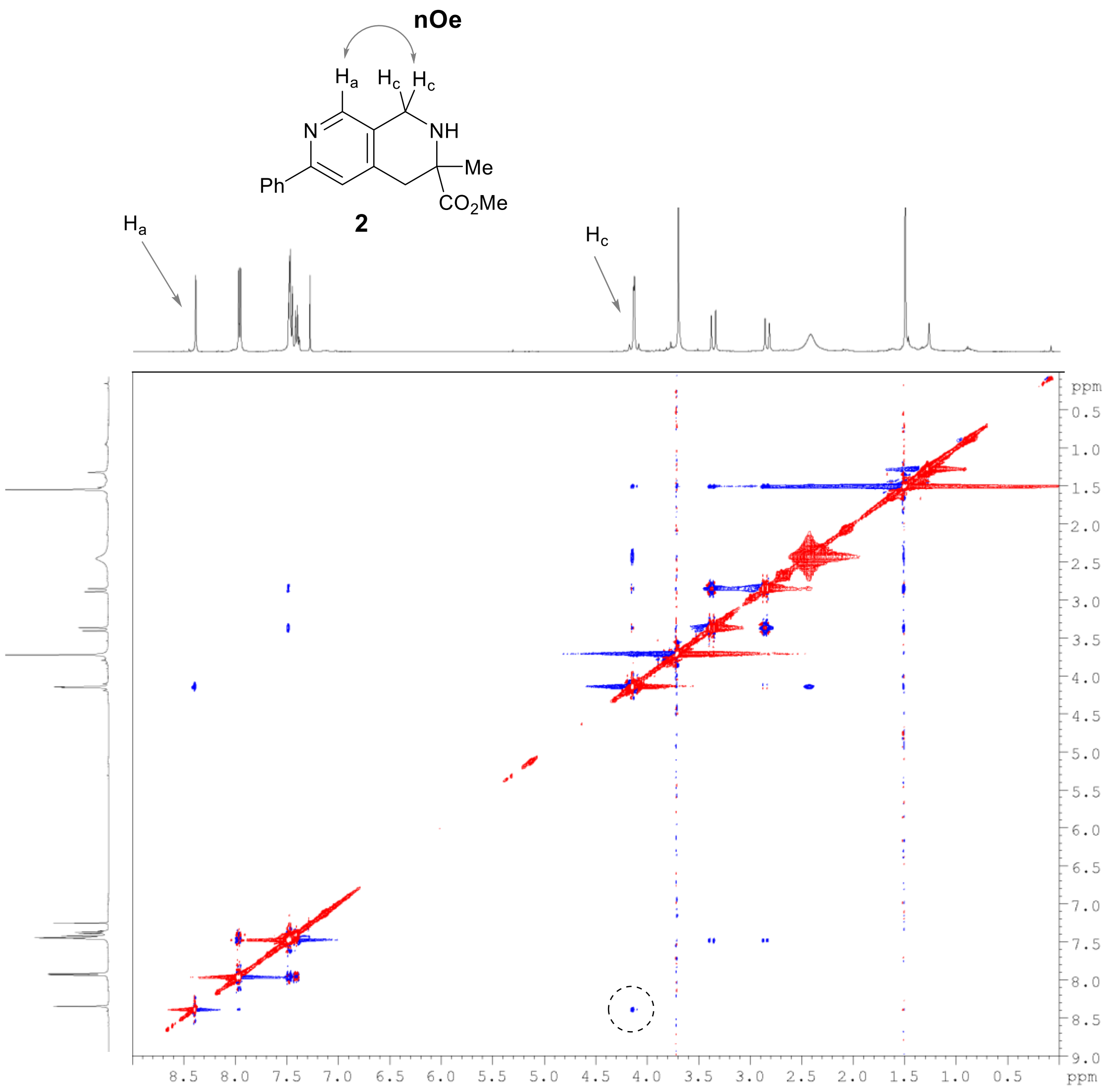

Figure S3.

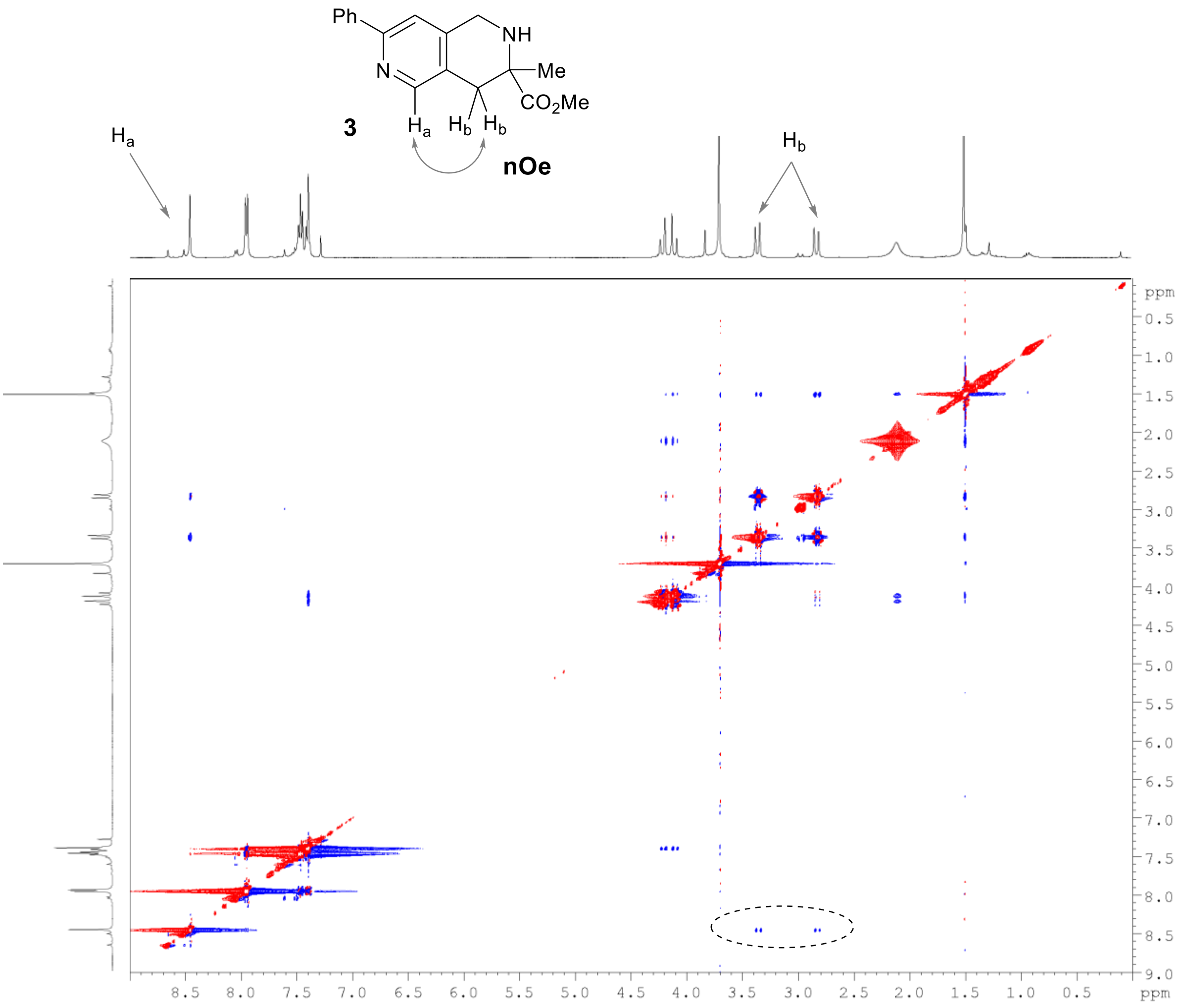

Figure S4.

Compound 14

Attempts to determine the relative position of the substituents present within **14** were carried out. Unfortunately, nOe experiments were not conclusive however, further analysis is being carried out to determine the relative configuration of **14**.

Compound 16

The relative position of the substituents present in **16** was established by means of nOe studies. Thereby, in the nOe spectrum of compound **16**, the alkene proton (H_a) showed an interaction with the NH proton (H_b) (Figure S6). Furthermore, an interaction could also be seen between the alkene proton (H_a) and the *t*-butyl group methyl groups H_c).

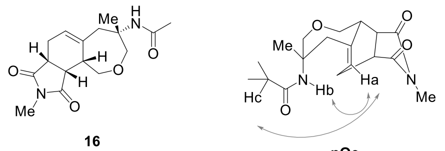
Me
H
N
O
H
H
O
H
O
N
Me
O
16
O
O
Me
N
Hc
Hb
O
Ha
O
N
Me
nOe

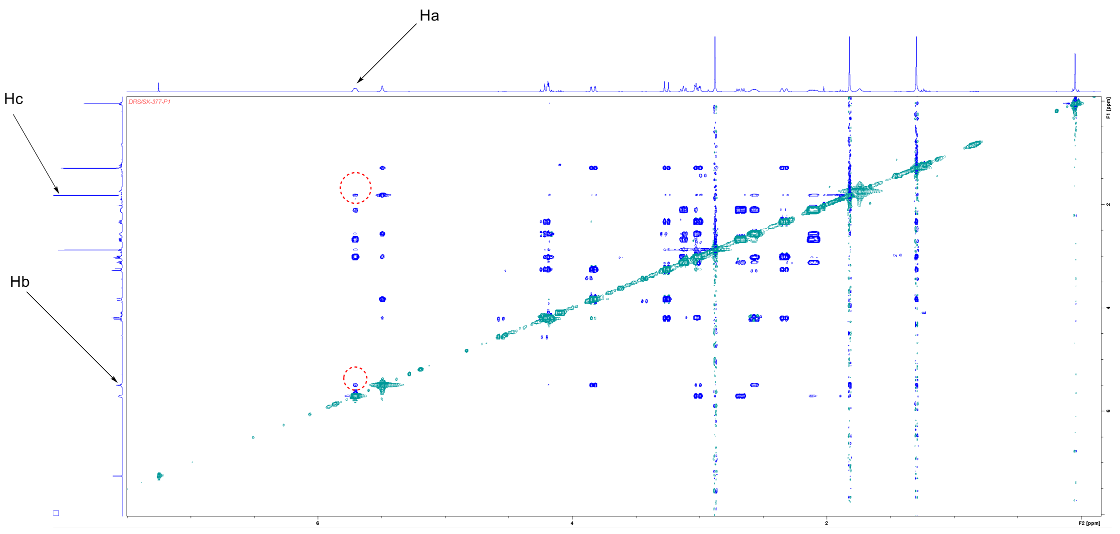
Ha
Hc
Hb
F2 [ppm]
F1 [ppm]

Figure S6.

COMPUTATIONAL ANALYSIS

A) Principal Moment of Inertia (PMI)

General details

Principal Moment of Inertia (PMI) was performed using Molecular Operating Environment (MOE) software package version 2012.10 from the Chemical Computing Group. Merck molecular force field 94X (MMFF94x), an all-atom force field parameterised for small organic molecules with the Generalised Born solvation model, was used to minimise the energy potential of the library members. A LowModeMD search was employed for the conformation generation. Detailed settings for conformational search are listed below.

Rejection Limit	100
RMS Gradient	0.005
Iteration Limit	10000
MM Iteration Limit	500
RMSD Limit	0.15
Energy window	3
Conformation Limit	100

Only the conformer with the lowest energy was retained for principal moment of inertia (PMI) calculations. Normalized PMI ratios (I1/I3 and I2/I3) of these conformers were obtained from MOE and then plotted on a triangular graph, with the canonical coordinates (0,1), (0.5,0.5) and (1,1) representing a perfect rod, disc and sphere respectively (Figure 3).

Compound collections analysed and PMI plots

Collection 1: DOS Library based on reported 40 small molecules.

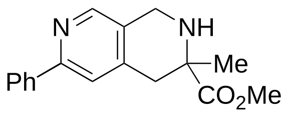

2

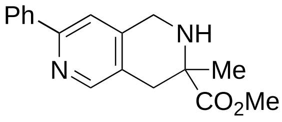

3

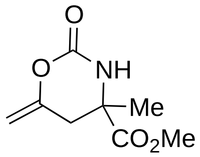

4

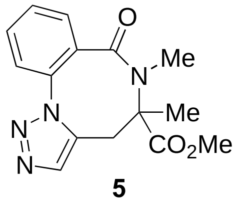

5

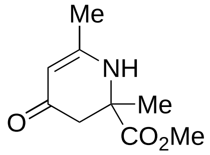

6

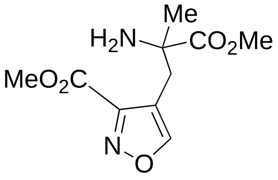

7

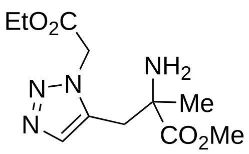

8

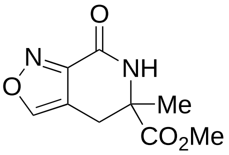

9

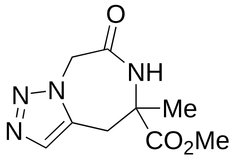

10

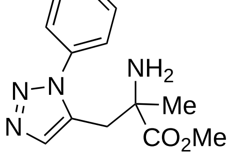

11

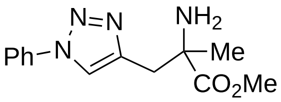

12

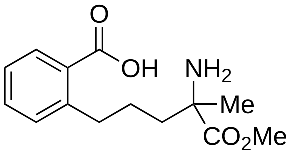

13

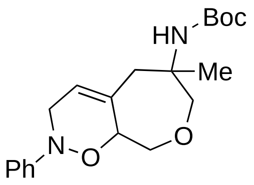

14

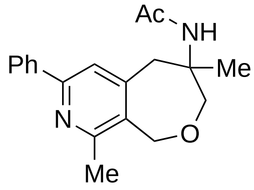

15

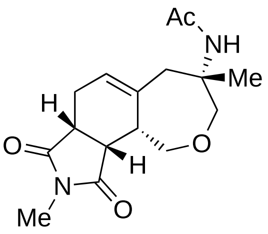

16

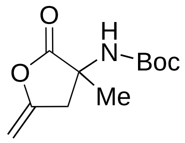

17

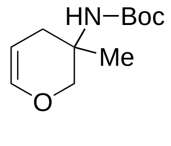

18

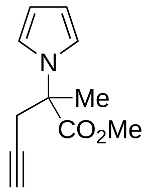

19

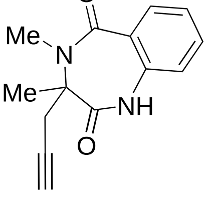

21

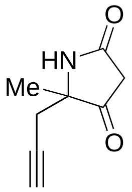

22

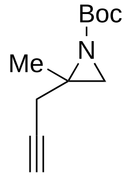

23

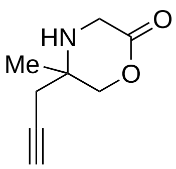

24

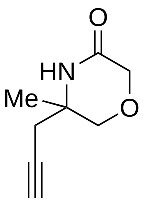

25

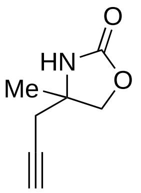

26

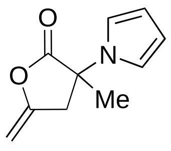

27

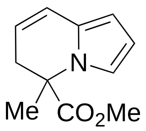

28

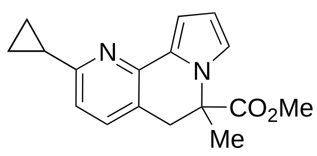

29

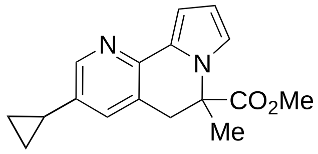

30

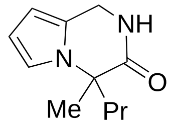

31

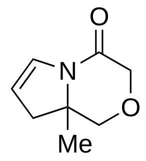

32

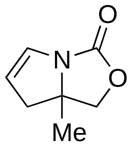

33

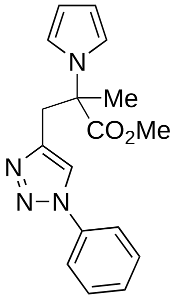

19a

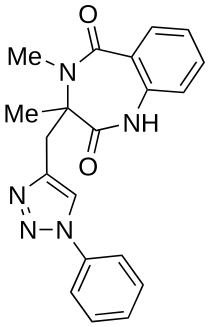

21a

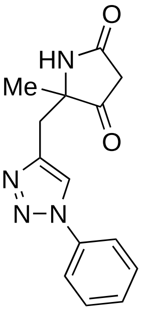

22a

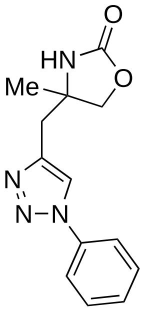

26a

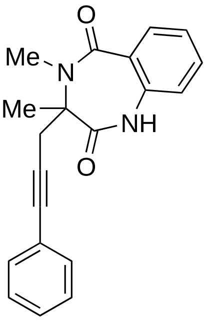

21b

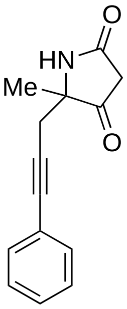

22b

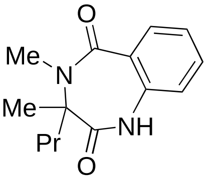

21c

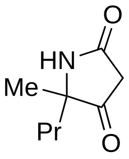

22c

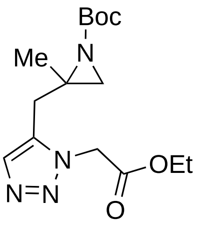

23d

Table S1. Chemical structures of DOS Library in SMILES format.

Compound	SMILES
**2**	O=C(C1(C)CC2=C(C=NC(C3=CC=CC=C3)=C2)CN1)OC
**3**	O=C(C1(C)CC2=C(C=C(C3=CC=CC=C3)N=C2)CN1)OC
**4**	C=C(CC(C(OC)=O)(C)N1)OC1=O
**5**	O=C1N(C)C(C(OC)=O)(C)CC2=CN=NN2C3=C1C=CC=C3
**6**	O=C1C=C(C)NC(C)(C(OC)=O)C1
**7**	NC(C)(C(OC)=O)CC1=CON=C1C(OC)=O
**8**	NC(CC1=CN=NN1CC(OCC)=O)(C)C(OC)=O
**9**	O=C1NC(C)(C(OC)=O)CC2=CON=C21
**10**	O=C(CN1C(C2)=CN=N1)NC2(C(OC)=O)C
**11**	NC(CC1=CN=NN1C2=CC=CC=C2)(C)C(OC)=O
**12**	NC(CC1=CN(C2=CC=CC=C2)N=N1)(C(OC)=O)C

**13**	NC(CCCC1=CC=CC=C1C(O)=O)(C)C(OC)=O
**14**	CC(C1)(NC(OC(C)(C)C)=O)COCC2C1=CCN(C3=CC=CC=C3)O2
**15**	CC1(NC(C)=O)COCC2=C(C)N=C(C3=CC=CC=C3)C=C2C1
**16**	O=C(N1C)[C@]([C@@]2([H])C1=O)([H])CC=C3[C@@H]2COC[C@](N C(C)=O)(C)C3
**17**	O=C1C(NC(OC(C)(C)C)=O)(C)CC(O1)=C
**18**	CC1(NC(OC(C)(C)C)=O)COC=CC1
**19**	C#CCC(C)(C(OC)=O)N1C=CC=C1
**21**	O=C(N(C)C1(CC#C)C)C2=C(C=CC=C2)NC1=O
**22**	O=C(NC1(CC#C)C)CC1=O
**23**	C#CCC1(C)N(C(OC(C)(C)C)=O)C1
**24**	C#CCC(CO1)(C)NCC1=O
**25**	O=C1COCC(CC#C)(C)N1
**26**	C#CCC(CO1)(C)NC1=O
**27**	O=C1C(N2C=CC=C2)(C)CC(O1)=C
**28**	CC1(C(OC)=O)CC=CC2=CC=CN12
**29**	O=C(C1(C)CC2=CC=C(C3CC3)N=C2C4=CC=CN14)OC
**30**	O=C(C1(C)CC2=CC(C3CC3)=CN=C2C4=CC=CN14)OC
**31**	O=C(NC1)C(C)(CCC)N2C1=CC=C2
**32**	O=C1COCC2(C)N1C=CC2
**33**	O=C1OCC2(C)N1C=CC2
**19a**	CC(CC1=CN(C2=CC=CC=C2)N=N1)(C(OC)=O)N3C=CC=C3
**21a**	O=C(N(C)C1(CC2=CN(C3=CC=CC=C3)N=N2)C)C4=C(C=CC=C4)NC1 =O
**22a**	O=C(NC1(CC2=CN(C3=CC=CC=C3)N=N2)C)CC1=O
**26a**	O=C1OCC(CC2=CN(C3=CC=CC=C3)N=N2)(C)N1

**21b**	O=C(N(C)C1(CC#CC2=CC=CC=C2)C)C3=C(C=CC=C3)NC1=O
**22b**	O=C(NC1(CC#CC2=CC=CC=C2)C)CC1=O
**21c**	O=C(N(C)C1(CCC)C)C2=C(C=CC=C2)NC1=O
**22c**	O=C(NC1(C)CCC)CC1=O
**23d**	O=C(OCC)CN1C(CC2(C)N(C(OC(C)(C)C)=O)C2)=CN=N1

Table S2. Normalised PMI ratio (npr) values of conformers of the DOS Library with the lowest energy.

Compound	npr1	npr2
**2**	0.2007	0.9149
**3**	0.1761	0.9628
**4**	0.6608	0.9315
**5**	0.4033	0.7284
**6**	0.348	0.7927
**7**	0.2989	0.8118
**8**	0.3747	0.7195
**9**	0.2735	0.8451
**10**	0.3122	0.813
**11**	0.3506	0.7691
**12**	0.1877	0.9268
**13**	0.4495	0.9293
**14**	0.3226	0.8555
**15**	0.2727	0.8894
**16**	0.2417	0.9216
**17**	0.2034	0.9381

Compound	npr1	npr2
**23**	0.4231	0.7895
**24**	0.2465	0.9311
**25**	0.4579	0.8303
**26**	0.2325	0.9648
**27**	0.3332	0.9538
**28**	0.4224	0.8973
**29**	0.3633	0.8393
**30**	0.3696	0.7741
**31**	0.313	0.8822
**32**	0.6285	0.7595
**33**	0.4928	0.7482
**19a**	0.2723	0.9423
**21a**	0.3108	0.8538
**22a**	0.221	0.9554
**26a**	0.0846	0.9619
**21b**	0.3231	0.8831

**18**	0.2626	0.9435
**19**	0.646	0.695
**21**	0.5321	0.8555
**22**	0.4319	0.7592

**22b**	0.1535	0.9378
**21c**	0.6047	0.9251
**22c**	0.538	0.8653
**23d**	0.3812	0.8038

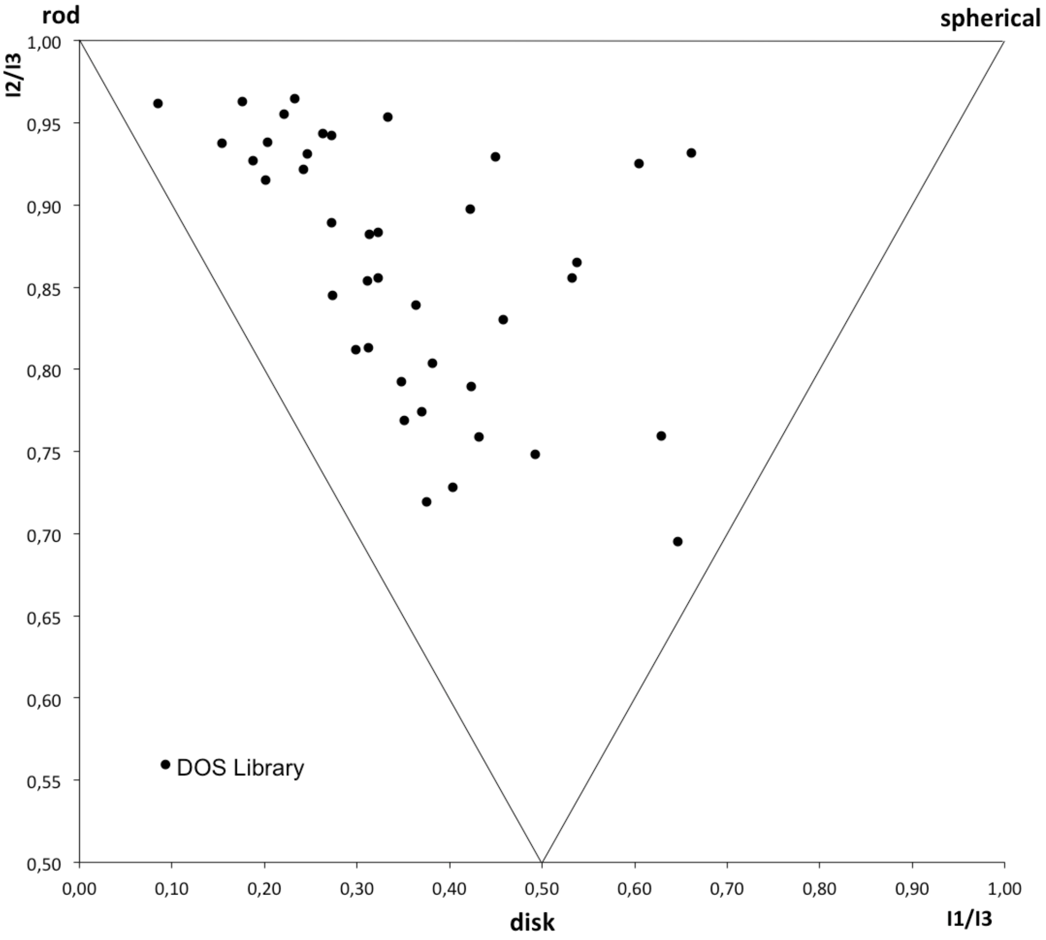

Figure S7. PMI plot of DOS Library.

Collection 2: DOS Library Ph. Virtual collection of 40 small molecules based on DOS Library featuring a Ph substituent at the quaternary stereocenter.

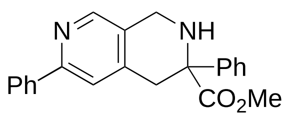

2’

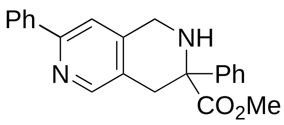

3’

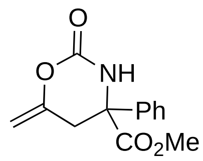

4’

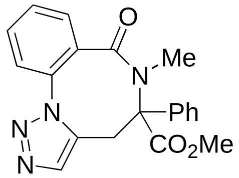

5’

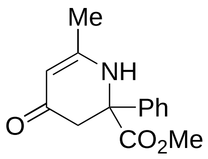

6’

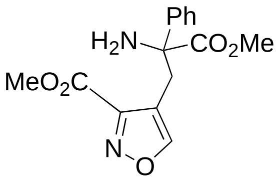

7’

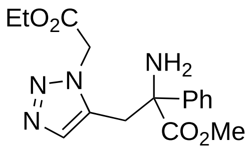

8’

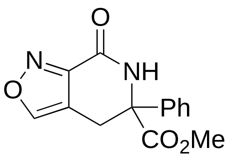

9’

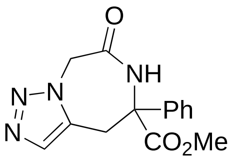

10’

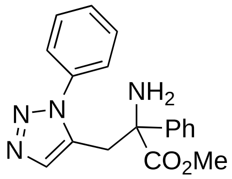

11’

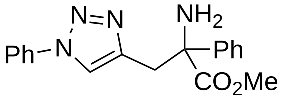

12’

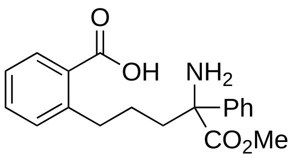

13’

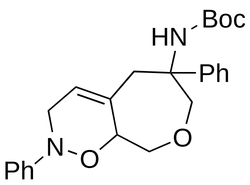

14’

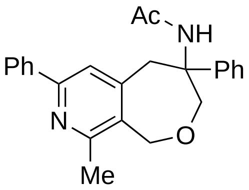

15’

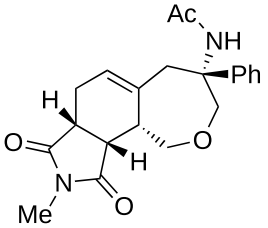

16’

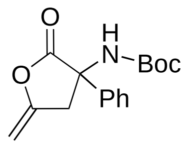

17’

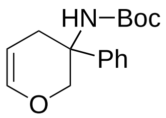

18’

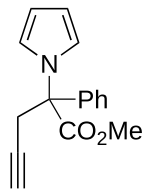

19’

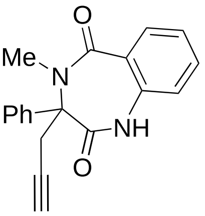

21’

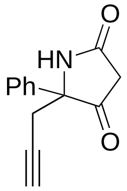

22’

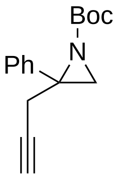

23’

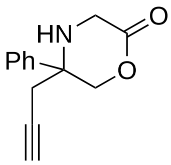

24’

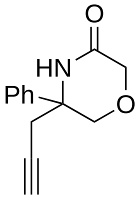

25’

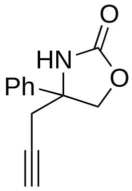

26’

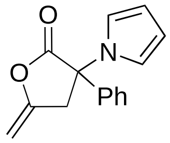

27’

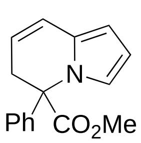

28’

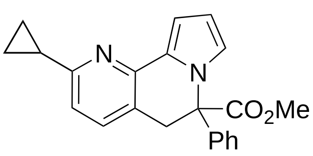

29’

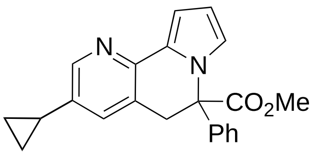

30’

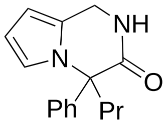

31’

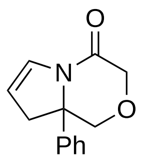

32’

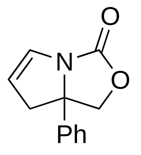

33’

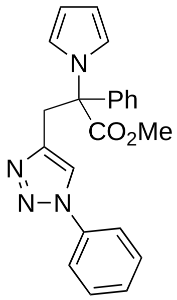

19a’

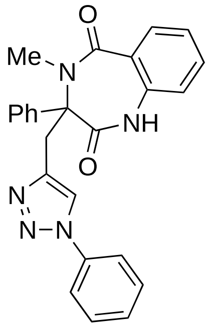

21a’

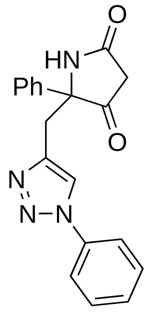

22a’

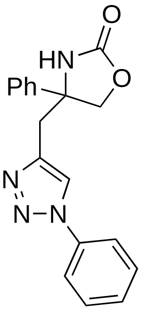

26a’

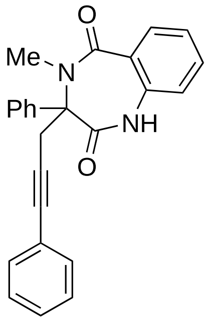

21b’

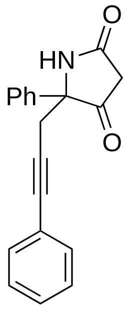

22b’

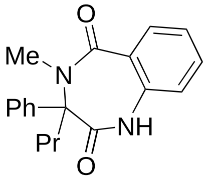

21c’

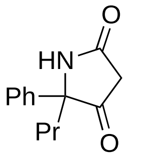

22c’

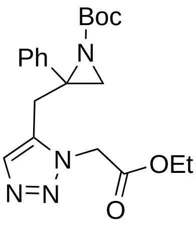

23d’

Table S3. Chemical structures of DOS Library Ph in SMILES format.

Compound	SMILES
**2’**	O=C(C1(C2=CC=CC=C2)CC3=C(C=NC(C4=CC=CC=C4)=C3)CN1)OC
**3’**	O=C(C1(C2=CC=CC=C2)CC3=C(C=C(C4=CC=CC=C4)N=C3)CN1)OC
**4’**	C=C(CC(C(OC)=O)(C1=CC=CC=C1)N2)OC2=O
**5’**	O=C1N(C)C(C(OC)=O)(C2=CC=CC=C2)CC3=CN=NN3C4=C1C=CC=C4
**6’**	O=C1C=C(C)NC(C2=CC=CC=C2)(C(OC)=O)C1
**7’**	NC(C1=CC=CC=C1)(C(OC)=O)CC2=CON=C2C(OC)=O
**8’**	NC(CC1=CN=NN1CC(OCC)=O)(C2=CC=CC=C2)C(OC)=O
**9’**	O=C1NC(C2=CC=CC=C2)(C(OC)=O)CC3=CON=C31
**10’**	O=C(CN1C(C2)=CN=N1)NC2(C(OC)=O)C3=CC=CC=C3

**11’**	NC(CC1=CN=NN1C2=CC=CC=C2)(C3=CC=CC=C3)C(OC)=O
**12’**	NC(CC1=CN(C2=CC=CC=C2)N=N1)(C(OC)=O)C3=CC=CC=C3
**13’**	NC(CCCC1=CC=CC=C1C(O)=O)(C2=CC=CC=C2)C(OC)=O
**14’**	CC(OC(NC(C1)(C2=CC=CC=C2)COCC3C1=CCN(C4=CC=CC=C4)O3)=O)(C)C
**15’**	CC1=C(COCC(NC(C)=O)(C2=CC=CC=C2)C3)C3=CC(C4=CC=CC=C4)=N1
**16’**	O=C(N1C)[C@]([C@@]2([H])C1=O)([H])CC=C3[C@@H]2COC[C@](NC(C)=O)(C4=CC=CC=C4)C3
**17’**	O=C1C(NC(OC(C)(C)C)=O)(C2=CC=CC=C2)CC(O1)=C
**18’**	CC(OC(NC1(C2=CC=CC=C2)COC=CC1)=O)(C)C
**19’**	C#CCC(C1=CC=CC=C1)(C(OC)=O)N2C=CC=C2
**21’**	O=C(N(C)C1(CC#C)C2=CC=CC=C2)C3=C(C=CC=C3)NC1=O
**22’**	O=C(NC1(CC#C)C2=CC=CC=C2)CC1=O
**23’**	C#CCC1(C2=CC=CC=C2)N(C(OC(C)(C)C)=O)C1
**24’**	C#CCC(CO1)(C2=CC=CC=C2)NCC1=O
**25’**	O=C1COCC(CC#C)(C2=CC=CC=C2)N1
**26’**	C#CCC(CO1)(C2=CC=CC=C2)NC1=O
**27’**	O=C1C(N2C=CC=C2)(C3=CC=CC=C3)CC(O1)=C
**28’**	O=C(C1(C2=CC=CC=C2)CC=CC3=CC=CN13)OC
**29’**	O=C(C1(C2=CC=CC=C2)CC3=CC=C(C4CC4)N=C3C5=CC=CN15)OC
**30’**	O=C(C1(C2=CC=CC=C2)CC3=CC(C4CC4)=CN=C3C5=CC=CN15)OC
**31’**	O=C(NC1)C(C2=CC=CC=C2)(CCC)N3C1=CC=C3
**32’**	O=C1COCC2(C3=CC=CC=C3)N1C=CC2
**33’**	O=C1OCC2(C3=CC=CC=C3)N1C=CC2
**19a’**	O=C(C(CC1=CN(C2=CC=CC=C2)N=N1)(C3=CC=CC=C3)N4C=CC=C4)OC

**21a'**	O=C(N(C)C1(CC2=CN(C3=CC=CC=C3)N=N2)C4=CC=CC=C4)C5=C(C=CC=C5)NC1=O
**22a'**	O=C(NC1(CC2=CN(C3=CC=CC=C3)N=N2)C4=CC=CC=C4)CC1=O
**26a'**	O=C1OCC(CC2=CN(C3=CC=CC=C3)N=N2)(C4=CC=CC=C4)N1
**21b'**	O=C(N(C)C1(CC#CC2=CC=CC=C2)C3=CC=CC=C3)C4=C(C=CC=C4)NC1=O
**22b'**	O=C(NC1(CC#CC2=CC=CC=C2)C3=CC=CC=C3)CC1=O
**21c'**	O=C(N(C)C1(CCC)C2=CC=CC=C2)C3=C(C=CC=C3)NC1=O
**22c'**	O=C(NC1(C2=CC=CC=C2)CCC)CC1=O
**23d'**	O=C(OCC)CN1C(CC2(C3=CC=CC=C3)N(C(OC(C)(C)C)=O)C2)=CN=N1

Table S4. Normalised PMI ratio (npr) values of conformers of the DOS Library Ph with the lowest energy.

Compound	npr1	npr2
**2'**	0.2509	0.2509
**3'**	0.2509	0.932
**4'**	0.4634	0.9343
**5'**	0.6252	0.7811
**6'**	0.6827	0.7967
**7'**	0.4118	0.4118
**8'**	0.4118	0.8226
**9'**	0.5677	0.773
**10'**	0.606	0.8234
**11'**	0.496	0.8478
**12'**	0.3317	0.8011
**13'**	0.3531	0.8914

Compound	npr1	npr2
**23**	0.3872	0.7793
**24'**	0.5471	0.754
**25'**	0.3858	0.7454
**26'**	0.4475	0.7587
**27'**	0.5158	0.8271
**28'**	0.643	0.784
**29'**	0.3867	0.9416
**30'**	0.4424	0.9206
**31'**	0.5641	0.6922
**32'**	0.5096	0.9404
**33'**	0.3555	0.8412
**19a'**	0.2371	0.9147

**14’**	0.222	0.9232
**15’**	0.2845	0.8513
**16’**	0.3576	0.8375
**17’**	0.4091	0.8306
**18’**	0.5367	0.7998
**19’**	0.6347	0.7591
**21’**	0.6175	0.8648
**22’**	0.5456	0.8896

**21a’**	0.2698	0.943
**22a’**	0.2834	0.8679
**26a’**	0.2682	0.8633
**21b’**	0.3082	0.9453
**22b’**	0.3405	0.8466
**21c’**	0.5731	0.8761
**22c’**	0.5695	0.7983
**23d’**	0.5009	0.7579

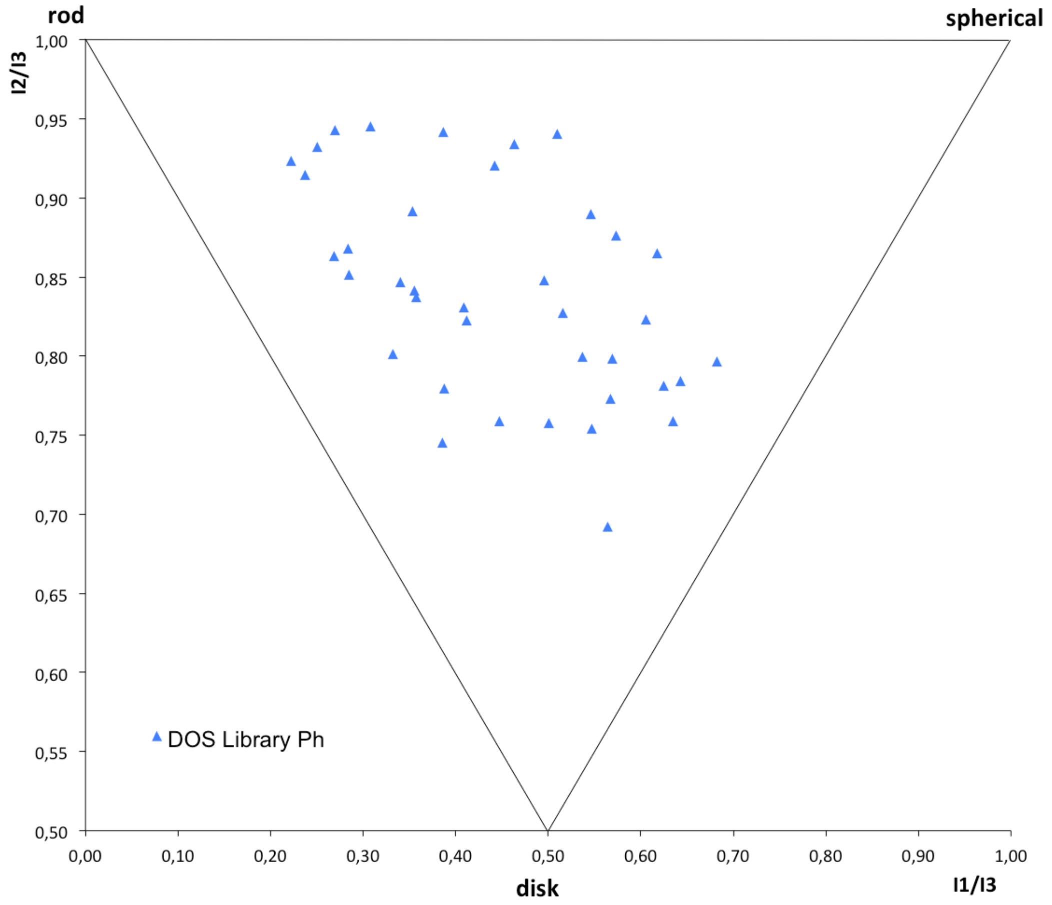

Figure S8. PMI plot of DOS Library Ph.

Collection 3: Maybridge 'Ro3' core fragment collection

This library is based on the core 1000-member collection within the Maybridge Fragment library. Details of the library (including SMILES and SDF) are available from 'http://www.maybridge.com/' under the 'Ro3 Fragment library section. More details can be found at:

'http://www.maybridge.com/images/pdfs/MB_Ro3_fragment_flyer_2011_EUR_v7.pdf'

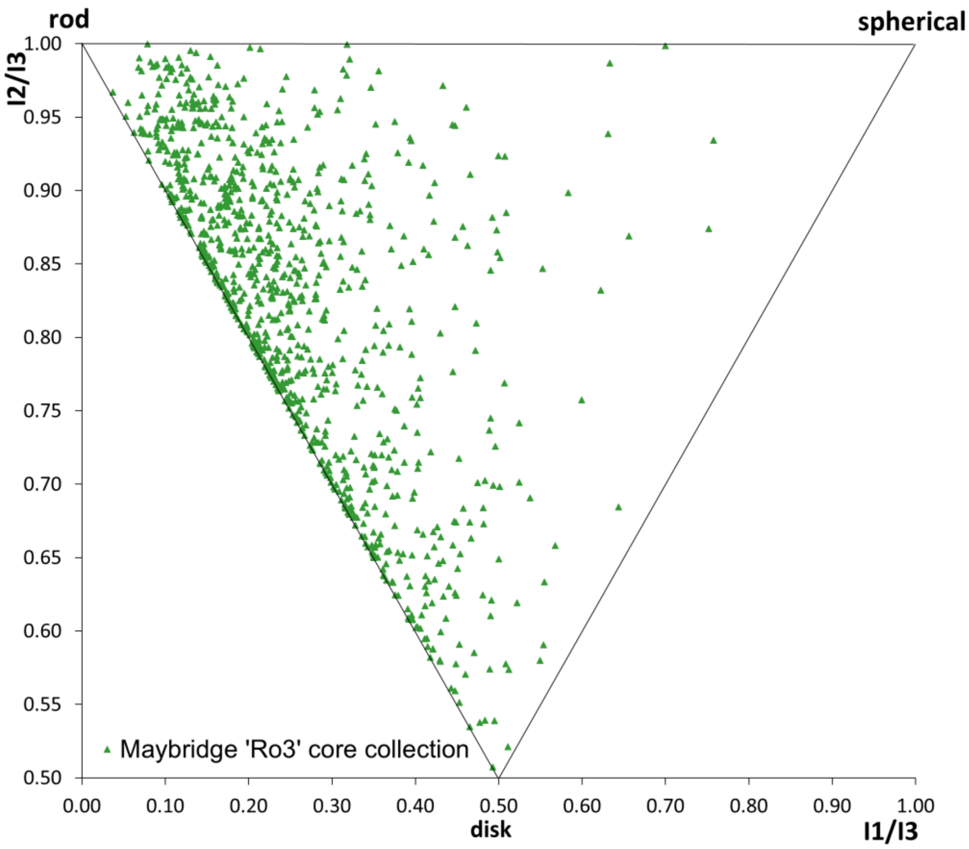

Figure S9. PMI plot of Maybridge 'Ro3' core collection.

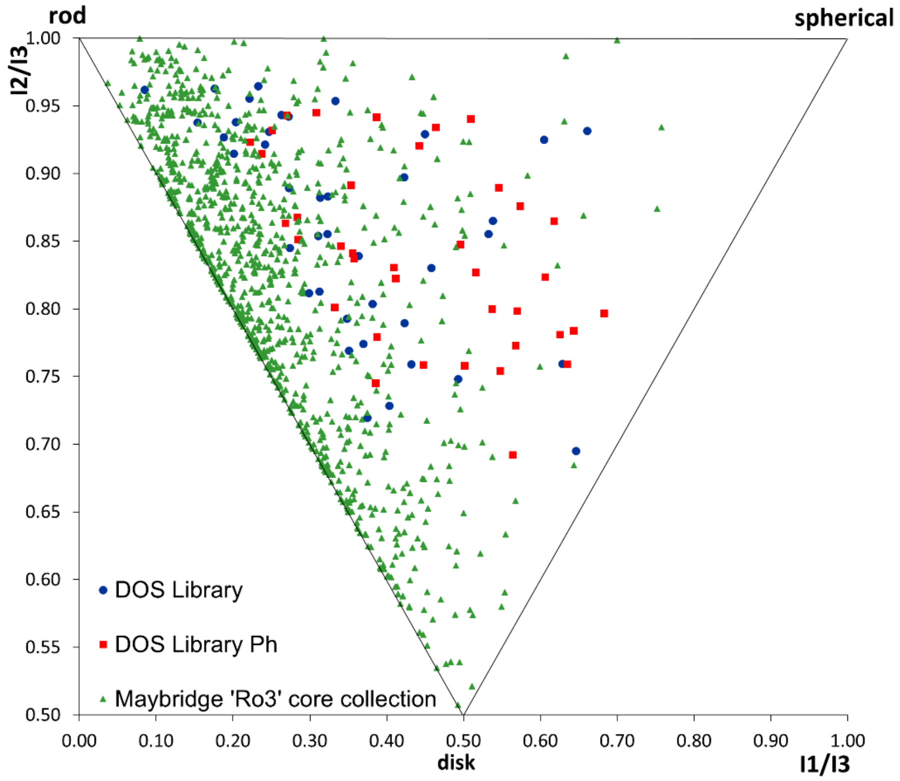

Figure S10. Comparative PMI plot of the presented DOS library, the virtual DOS Library Ph and the Maybridge 'Ro3' core collection.

Analysis of comparative PMI plot

Figure S9 shows that the synthesized DOS Library displays a high level of molecular shape diversity, covering a broad area of molecular shape space. Notable differences in the molecular shape space distribution can be observed between the DOS library and the DOS Library Ph, with a shift towards the right-hand side of the plot. In comparison to the Maybridge 'Ro3' core fragment collection, the DOS library presented in this work shows a broader coverage of the 3D molecular space shape, whilst the Maybridge library is densely populated around the 'rod-' and 'disk-' like areas of the plot. Furthermore, a higher percentage of the DOS Library and DOS Library Ph occupy the right-hand side of the plot suggesting more 3D character, when compared to the Maybridge library.

B) Computational evaluation of physicochemical properties

General details

Computational analysis was carried out using the same MOE software package and settings described above in section **A**. The DOS library compounds were analysed for the for the following properties: SlogP, molecular weight (MW), number of hydrogen-bond acceptors (HBA), number of hydrogen-bond donors (HBD), number of chiral centres and fraction aromatic (the number of aromatic atoms expressed as a fraction of the total number of heavy atoms).

The distribution of these data and the mean values are displayed in a series of histograms in Figure S11. By means of comparison with existing libraries, the mean values are shown alongside those of two popular commercially available fragment libraries, Chembridge (consisting of 7,547 fragments) and Maybridge ‘Ro3’ core collection (consisting of 1,000 fragments), in Table 1.

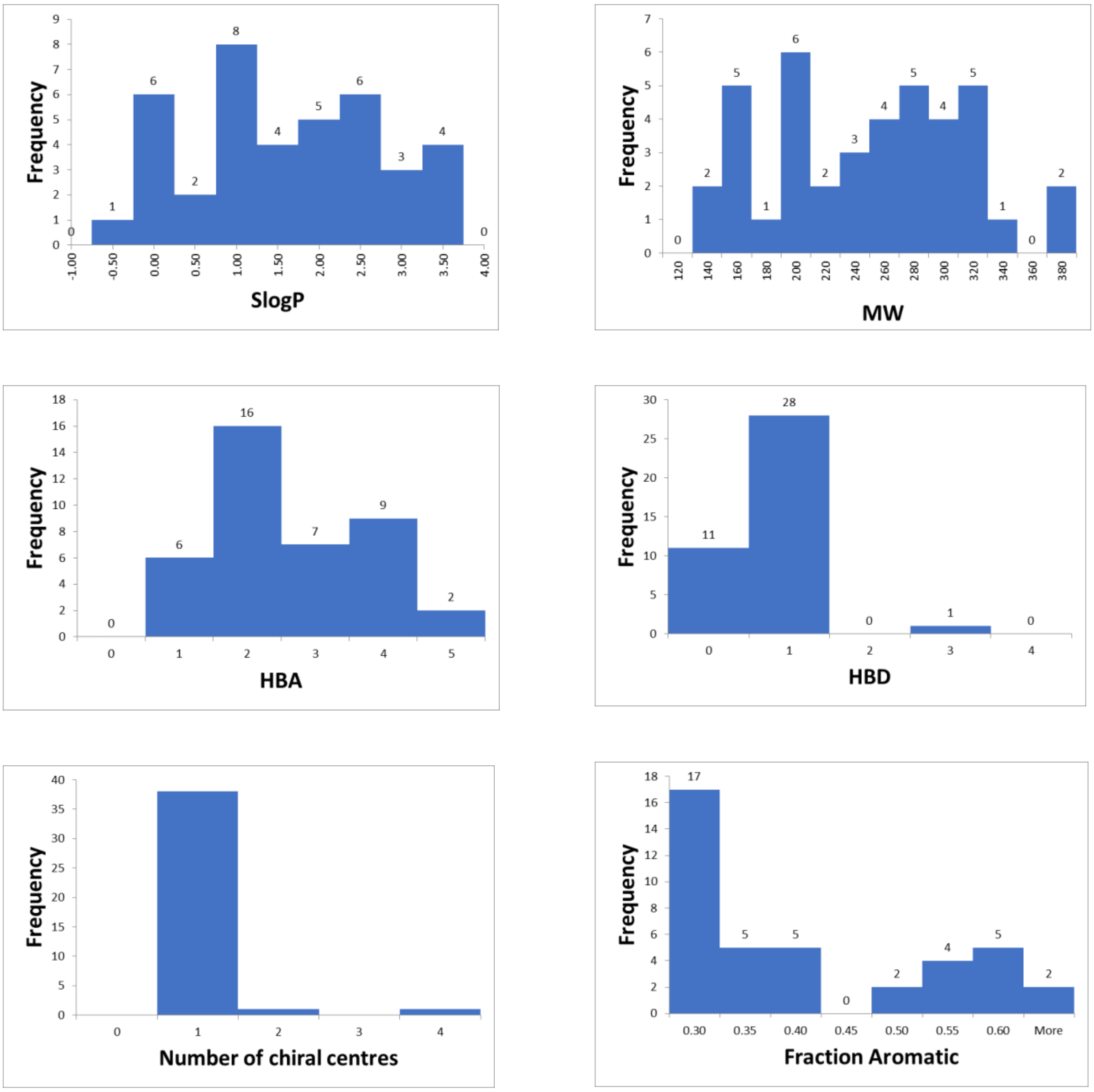

Figure S11. Histograms showing the distribution of predicted physicochemical properties for compounds in the DOS library.

C) Multi-Dimensional Scaling (MDS) Plot Generation

Selection of ChEMBL 20 compounds

All compounds reported as ligands of kinases (80,999), proteases (106,953), hydrolases (35,606), ion channels (51,182), membrane receptors (299,374), transporters (39,669), and transcription factors (136,557) were extracted from the ChEMBL 20 database,[8] followed by molecular weight filtering (200-700) and removal of salts using KNIME 2.11.3.[9] The KNIME workflow filtered the compounds in the following sequence: It (a) read the chemical data from the SDF file (b) calculated the RDKit[10] descriptor (M.W.) (c) applied the molecular weight filters *via* the Java snippet node (d) returned the data within the accepted range of molecular weights *via* the numeric row splitter node and finally (e) removed salts from the filtered compounds using the RDKit salt stripper node. Compounds of each bioactivity class passing all the applied filters, namely ligands of kinases (78,465 structures), proteases (75,504), hydrolases (33,877), ion channels (47,912), membrane receptors (170,461), transporters (37,730), and transcription factors (133,863), were written into separate SDF files.

Multi-Dimensional Scaling (MDS) analysis

2,000 ChEMBL compounds with IC_{50}, EC_{50} and K_i values ≤ 10 μM were randomly selected from each bioactivity class for MDS analysis. In addition, compounds synthesized *via* two different targeted approaches were used as a benchmark, namely those previously presented by Hergenrother (88 compounds),[11] and Kiessling (9 compounds).[12] For this purpose, the SDF files of all compounds belonging to the different bioactivity classes as well as the synthetic libraries and the DOS library presented were standardized using the

[8] A. Gaulton, L. J. Bellis, A. P. Bento, J. Chambers, M. Davies, A. Hersey, Y. Light, S. McGlinchey, D. Michalovich, B. Al-Lazikani, et al., *Nucleic Acids Res.* **2011**, *40*, 1.
[9] M. Berthold, N. Cebron, F. Dill, *SIGKDD Explor.* **2008**, *11*, 26.
[10] http://www.rdkit.org
[11] V. Nesterenko, K. S. Putt, P. J. Hergenrother, *J. Am. Chem. Soc.* **2003**, *125*, 14672.
[12] M. C. Schuster, D. A. Mann, T. J. Buchholz, K. M. Johnson, W. D. Thomas, L. L. Kiessling, *Org. Lett.* **2003**, *5*, 1407.

ChemAxon Command-Line Standardizer where the following options were selected: “Remove Fragment” (keep largest), “Neutralize”, “RemoveExplicitH”, “Clean2D”, “Mesomerize” and “Tautomerize”.[13]

Subsequently, Morgan fingerprints (radius 2, 1024 bits) were generated for all the compounds using KNIME 2.11.3. The workflow generated Morgan fingerprints in the following sequence: It (a) read chemical data from an SDF file, (b) generated RDKit molecules from a molecule string representation and appended them to a table, (c) generated hashed bit-based fingerprints for an input RDKit Mol column and appended them to a table, (d) converted RDKit molecules into string based molecule representations (SMILES), (e) excluded columns from the input table, (f) renamed columns and finally (g) saved the resulting data table into a CSV file.

Using the generated Morgan fingerprints, a 2D-similarity matrix based on Euclidean distance was calculated using R.[14] Then, a metric multidimensional scaling of the similarity matrix was computed by embedding it into two dimensions (k=2). Subsequently, a two-dimensional plot was obtained labelling the x-axis and the y-axis with Dimension 1 and Dimension 2 respectively, which are two unit less and relative dimensions that recapitulate the pairwise similarity between all points observed in an underlying distribution of Euclidean distances in a higher dimensional space. For each data set in the obtained plot (corresponding to each of the synthetic libraries and bioactivity classes), 90% confidence ellipses were computed using the ellipse package.[15] Finally, an MDS plot with 90% ellipse-like confidence regions was obtained using R ggplot2 package.[16]

NMR SPECTRA

---

[13] L. H. Mervin, A. M. Afzal, G. Drakakis, R. Lewis, O. Engkvist, A. Bender, *J. Cheminform.* **2015**, *7*, 1.
[14] R Core Team. R: A Language and Environment for Statistical Computing (version 3.2.4), **2016**.
[15] D. J. Murdoch, E. D. Chow, *Am. Stat.* **1996**, *50*, 178.
[16] Hadley Wickham, *ggplot2: Elegant Graphics for Data Analysis*, **2009**.

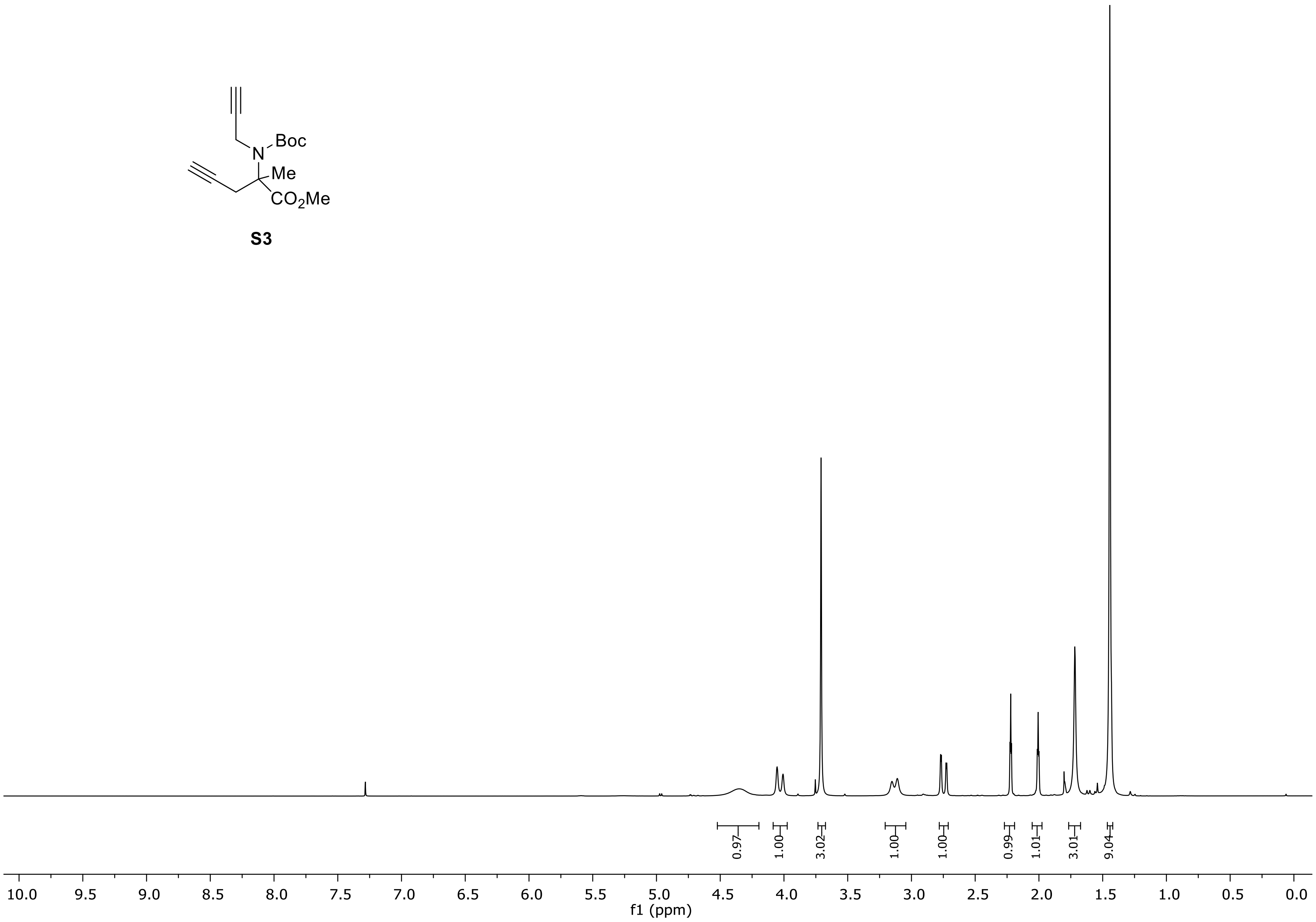
Boc
N
Me
CO_2Me
0.97
1.00
3.02
1.00
1.00
0.99
1.01
3.01
9.04
10.0 9.5 9.0 8.5 8.0 7.5 7.0 6.5 6.0 5.5 5.0 4.5 4.0 3.5 3.0 2.5 2.0 1.5 1.0 0.5 0.0
f1 (ppm)

S3

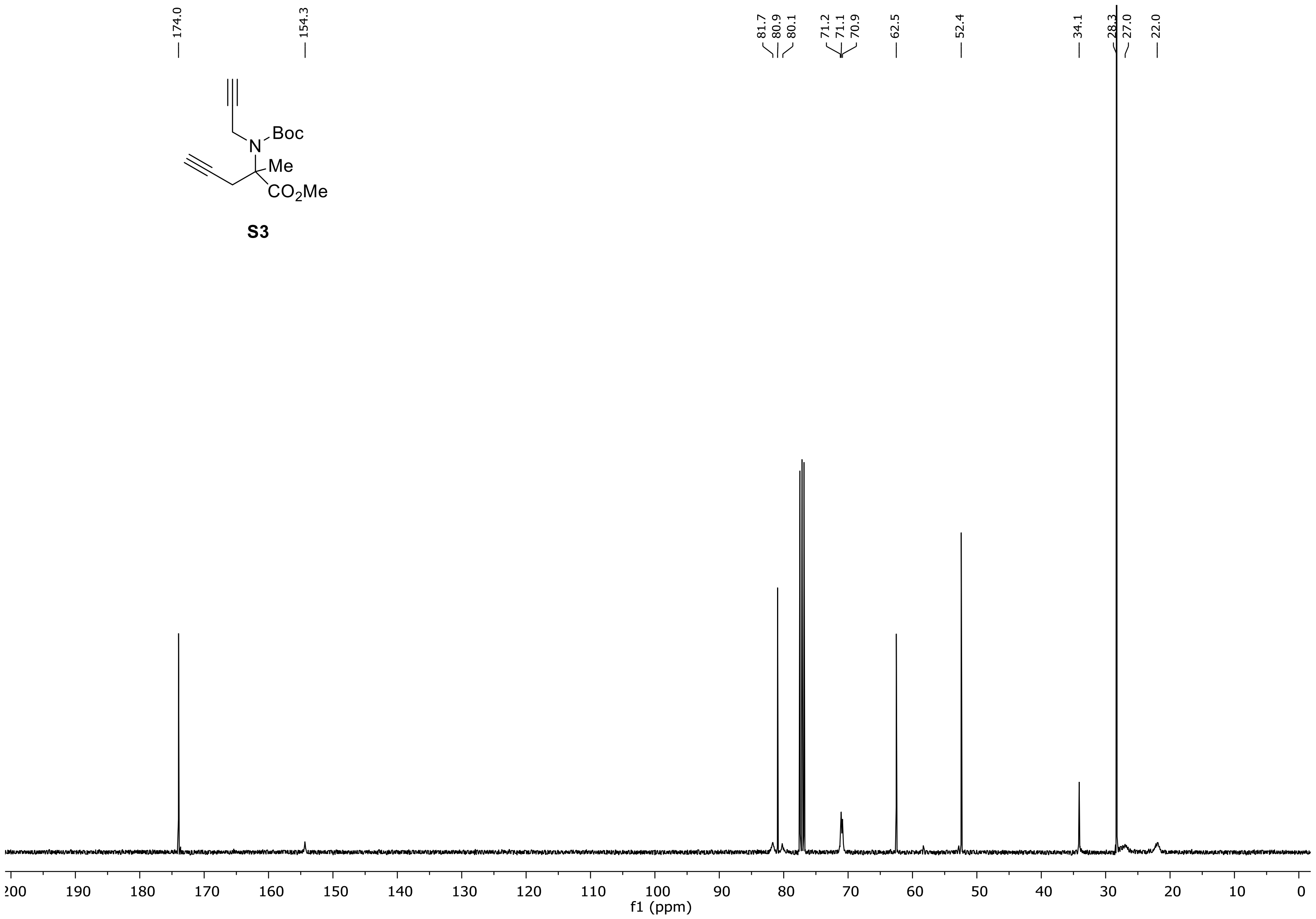

174.0
154.3
81.7
80.9
80.1
71.2
71.1
70.9
62.5
52.4
34.1
28.3
27.0
22.0
Boc
N
Me
CO2Me
S3
200 190 180 170 160 150 140 130 120 110 100 90 80 70 60 50 40 30 20 10 0
f1 (ppm)

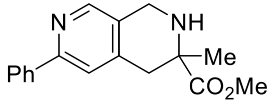
N
NH
Me
Ph
CO2Me

2

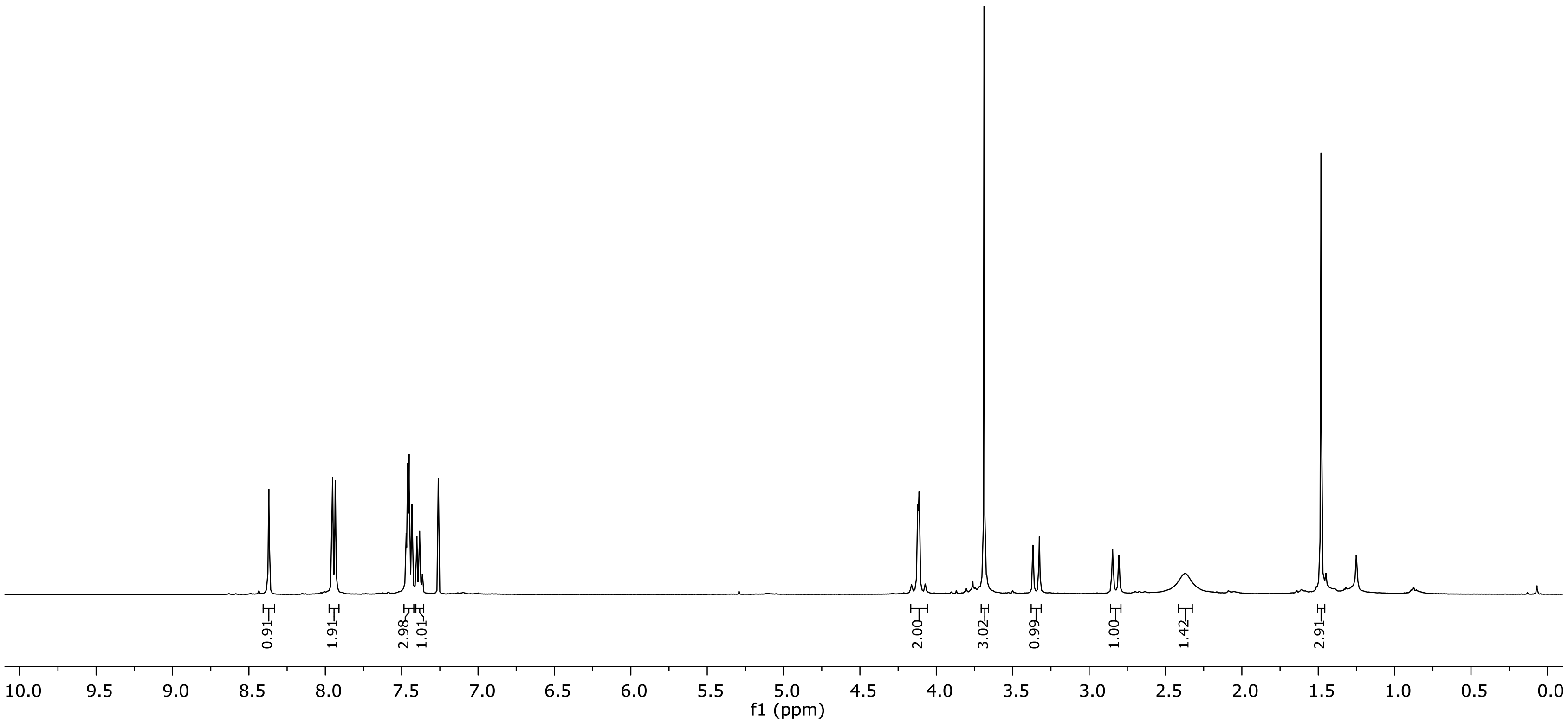
0.91
1.91
2.98
1.01
2.00
3.02
0.99
1.00
1.42
2.91
10.0 9.5 9.0 8.5 8.0 7.5 7.0 6.5 6.0 5.5 5.0 4.5 4.0 3.5 3.0 2.5 2.0 1.5 1.0 0.5 0.0
f1 (ppm)

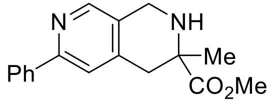
N
NH
Me
Ph
CO2Me

2

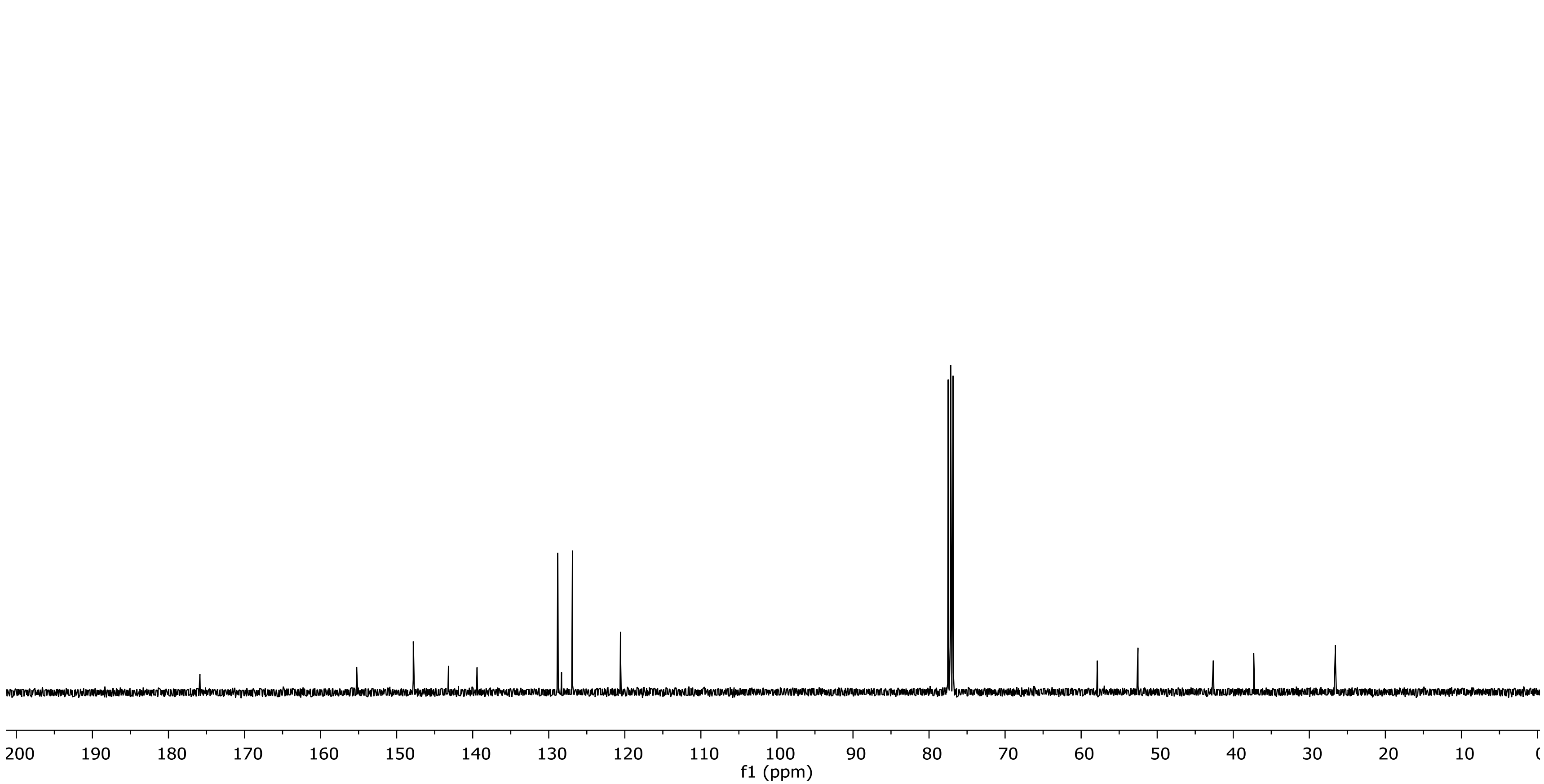
175.9
155.3
147.8
143.2
139.4
128.8
128.8
128.3
126.9
120.6
57.9
52.5
42.6
37.3
26.6
200 190 180 170 160 150 140 130 120 110 100 90 80 70 60 50 40 30 20 10
f1 (ppm)

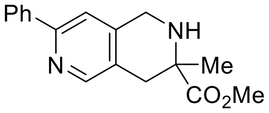

3

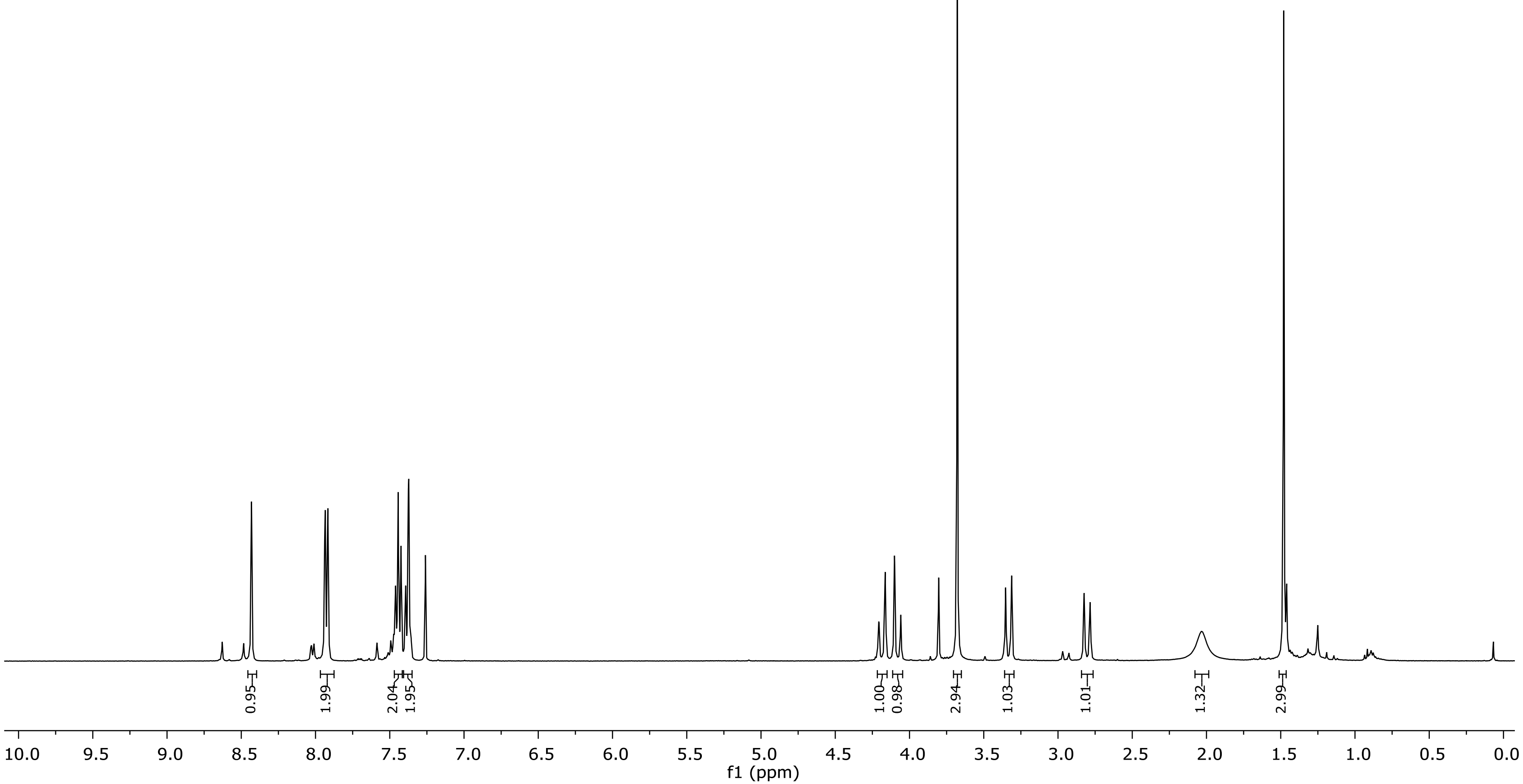
0.95
1.99
2.04
1.95
1.00
0.98
2.94
1.03
1.01
1.32
2.99
10.0
9.5
9.0
8.5
8.0
7.5
7.0
6.5
6.0
5.5
5.0
4.5
4.0
3.5
3.0
2.5
2.0
1.5
1.0
0.5
0.0
f1 (ppm)

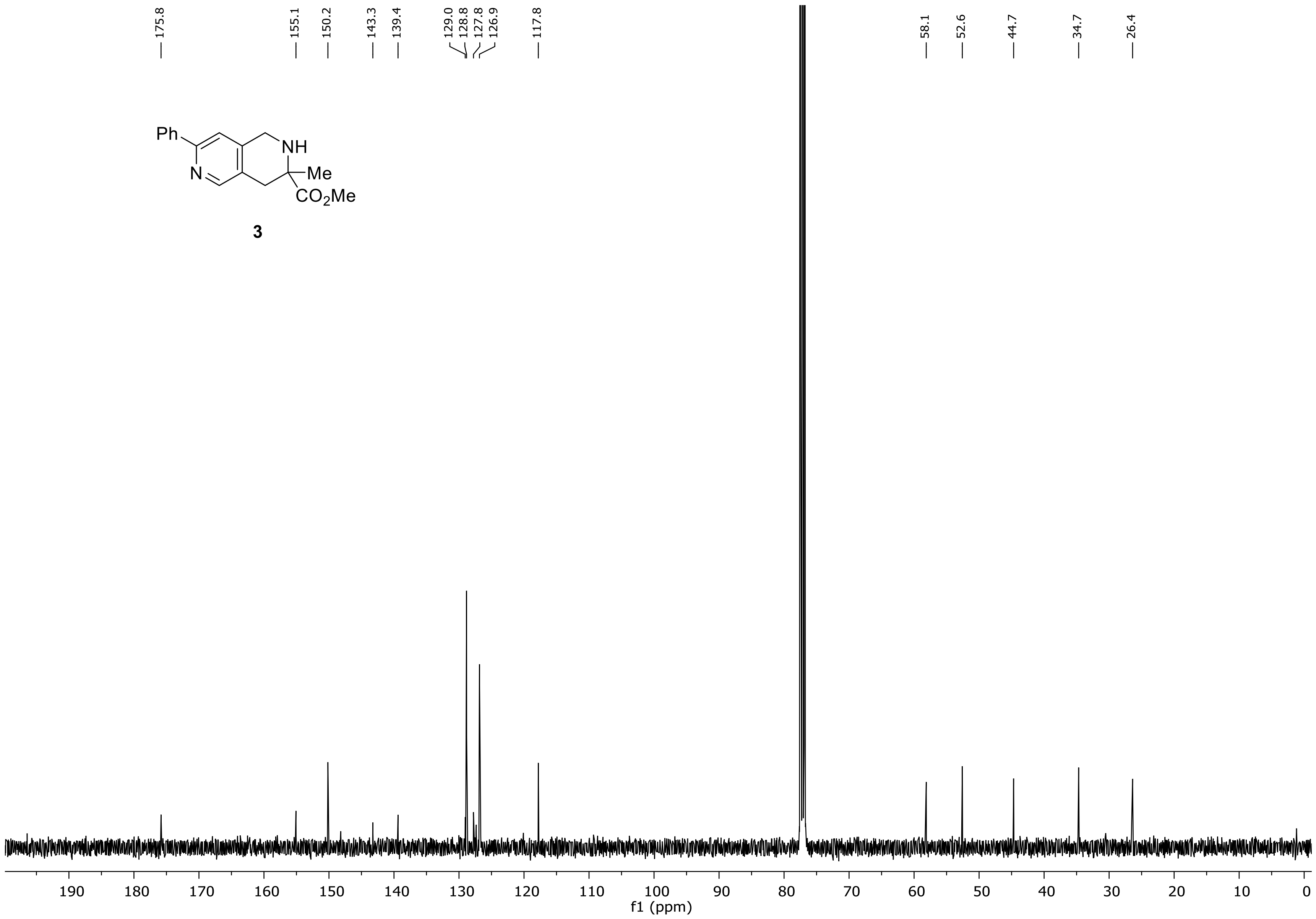
175.8
155.1
150.2
143.3
139.4
129.0
128.8
127.8
126.9
117.8
58.1
52.6
44.7
34.7
26.4
Ph
NH
N
Me
CO_2Me
3
190 180 170 160 150 140 130 120 110 100 90 80 70 60 50 40 30 20 10 0
f1 (ppm)

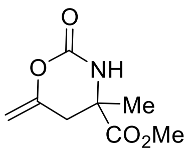
O
O
NH
Me
CO_2Me

4

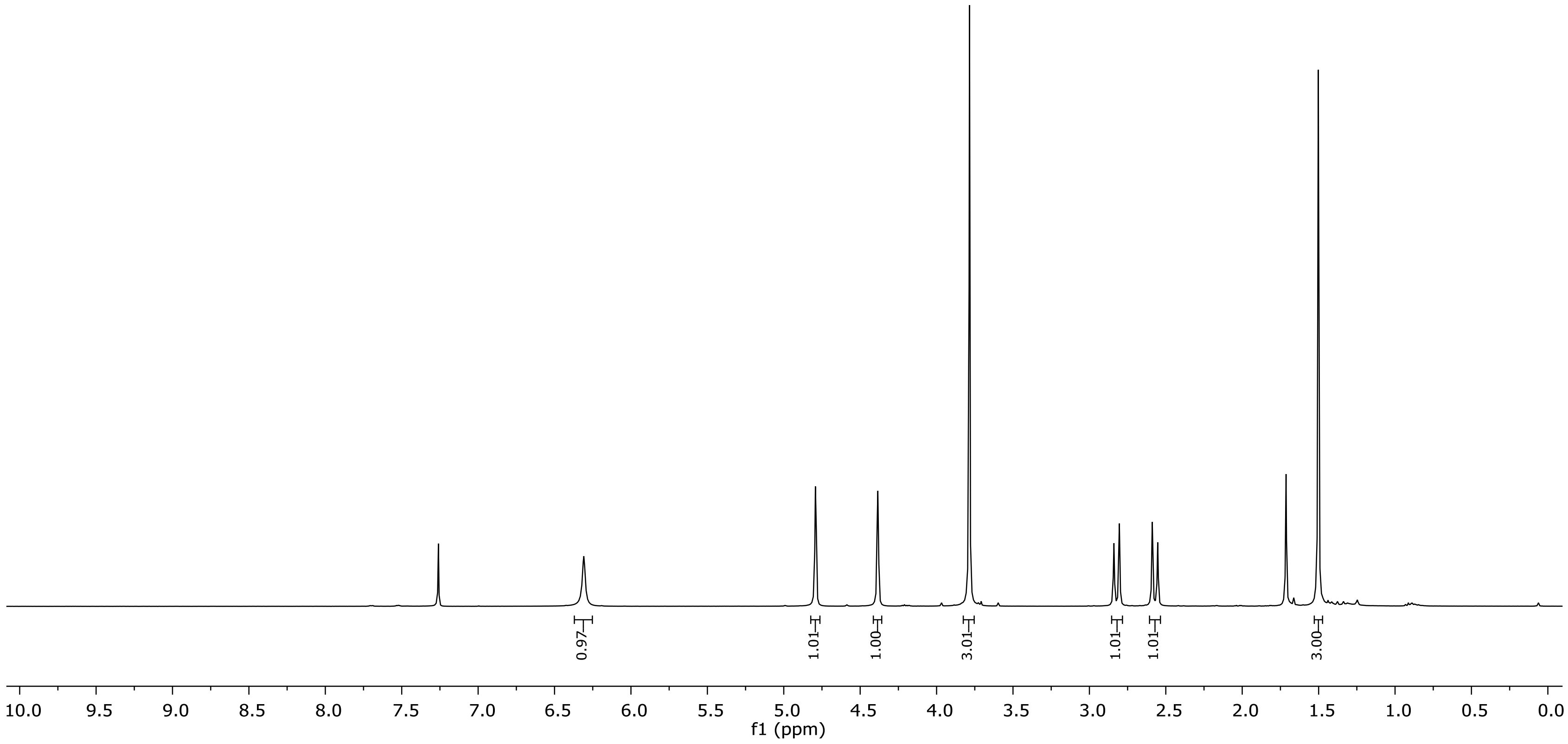
0.97
1.01
1.00
3.01
1.01
1.01
3.00
10.0
9.5
9.0
8.5
8.0
7.5
7.0
6.5
6.0
5.5
5.0
4.5
4.0
3.5
3.0
2.5
2.0
1.5
1.0
0.5
0.0
f1 (ppm)

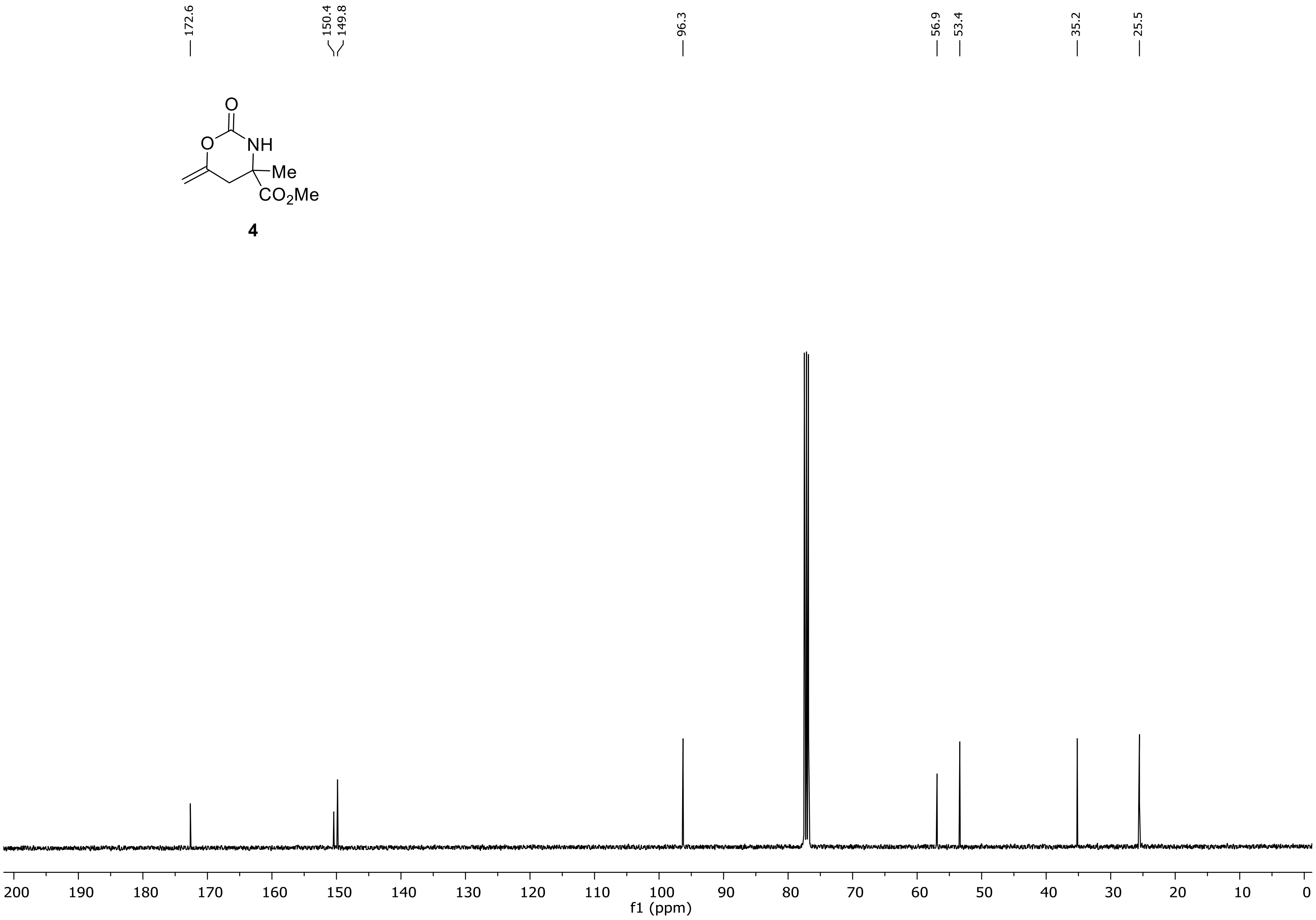
172.6
150.4
149.8
96.3
56.9
53.4
35.2
25.5
O
O
NH
Me
CO_2Me
4
200 190 180 170 160 150 140 130 120 110 100 90 80 70 60 50 40 30 20 10 0
f1 (ppm)

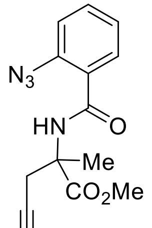

S4

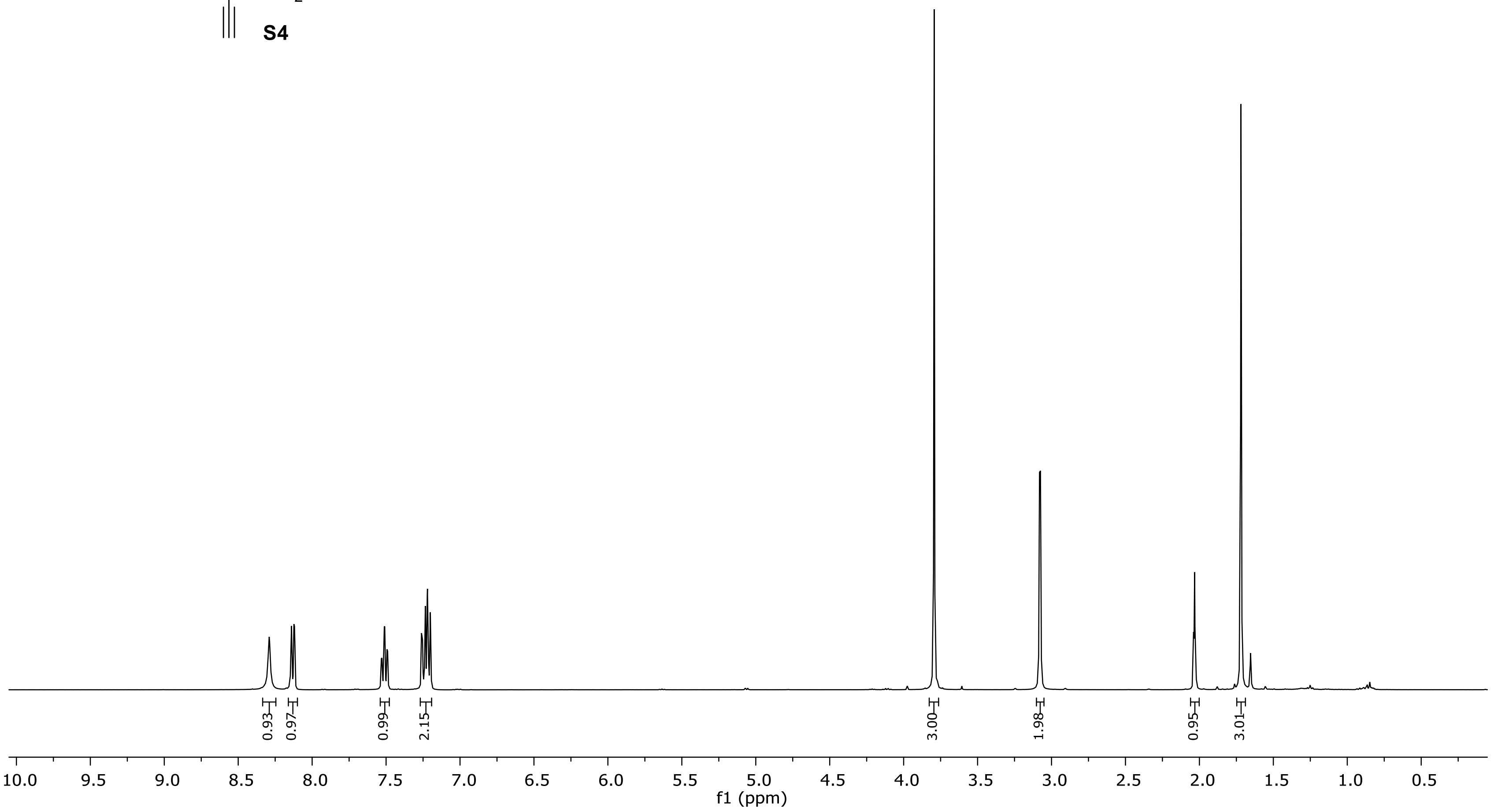

0.93
0.97
0.99
2.15
3.00
1.98
0.95
3.01
10.0 9.5 9.0 8.5 8.0 7.5 7.0 6.5 6.0 5.5 5.0 4.5 4.0 3.5 3.0 2.5 2.0 1.5 1.0 0.5
f1 (ppm)

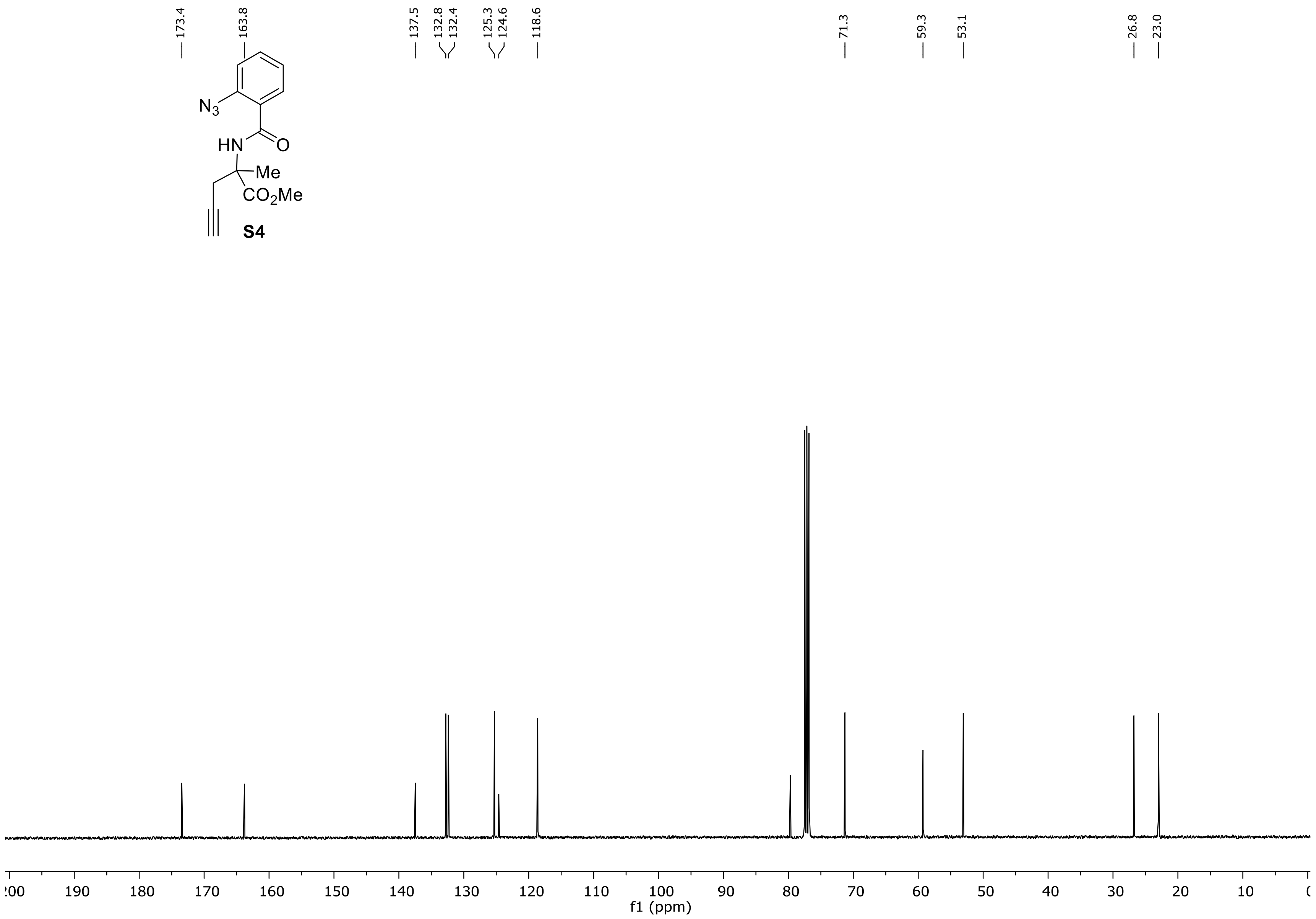
173.4
163.8
137.5
132.8
132.4
125.3
124.6
118.6
71.3
59.3
53.1
26.8
23.0
N3
HN
O
Me
CO2Me
S4
f1 (ppm)

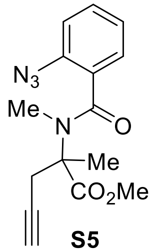
N3
Me
N
O
Me
CO2Me

S5

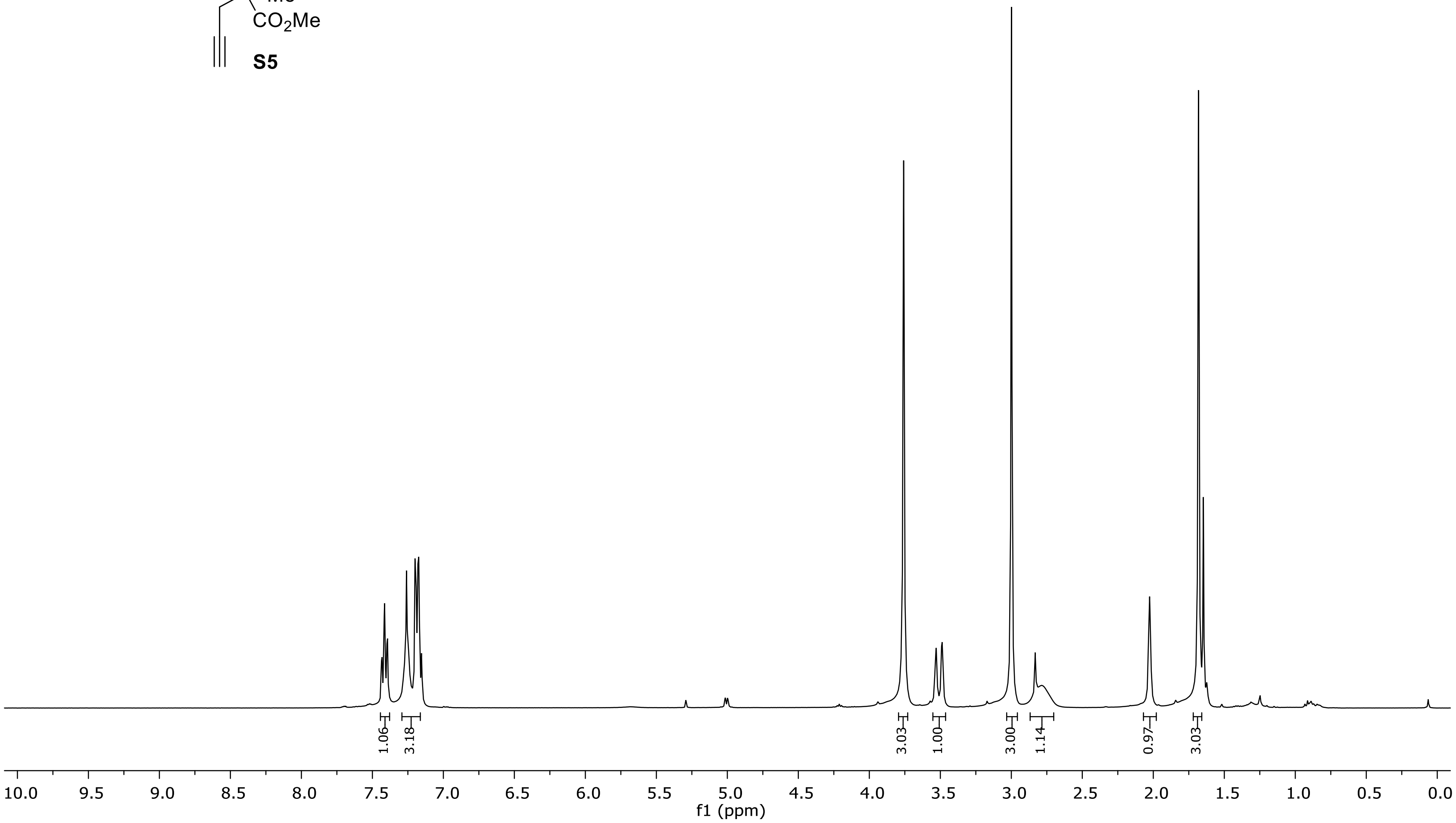
1.06
3.18
3.03
1.00
3.00
1.14
0.97
3.03
10.0 9.5 9.0 8.5 8.0 7.5 7.0 6.5 6.0 5.5 5.0 4.5 4.0 3.5 3.0 2.5 2.0 1.5 1.0 0.5 0.0
f1 (ppm)

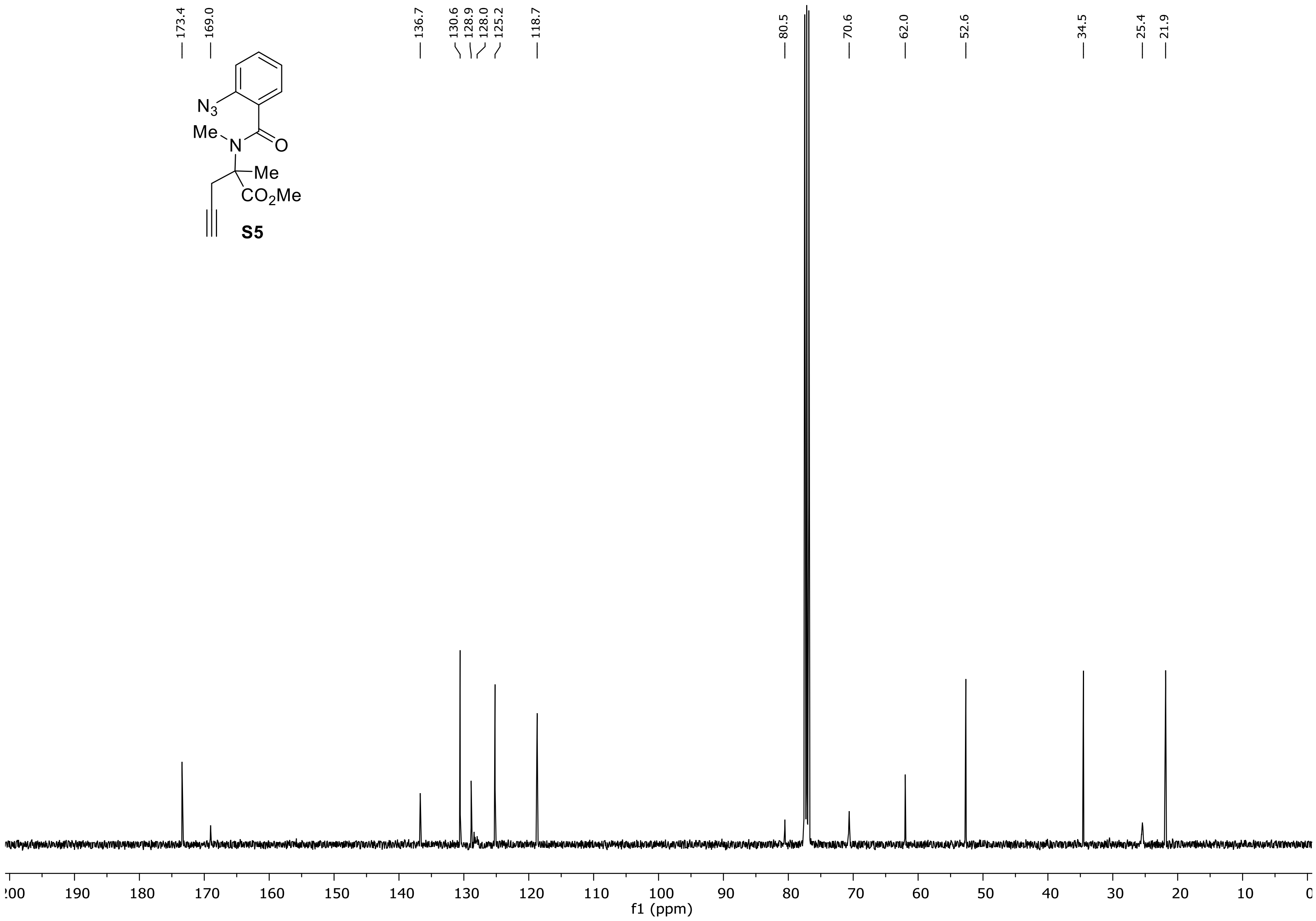
173.4
169.0
136.7
130.6
128.9
128.0
125.2
118.7
80.5
70.6
62.0
52.6
34.5
25.4
21.9
N3
Me
N
O
Me
CO2Me
S5
200
190
180
170
160
150
140
130
120
110
100
90
80
70
60
50
40
30
20
10
0
f1 (ppm)

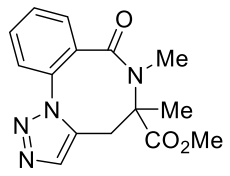
O
N
Me
Me
N
N
N
N
CO2Me

5

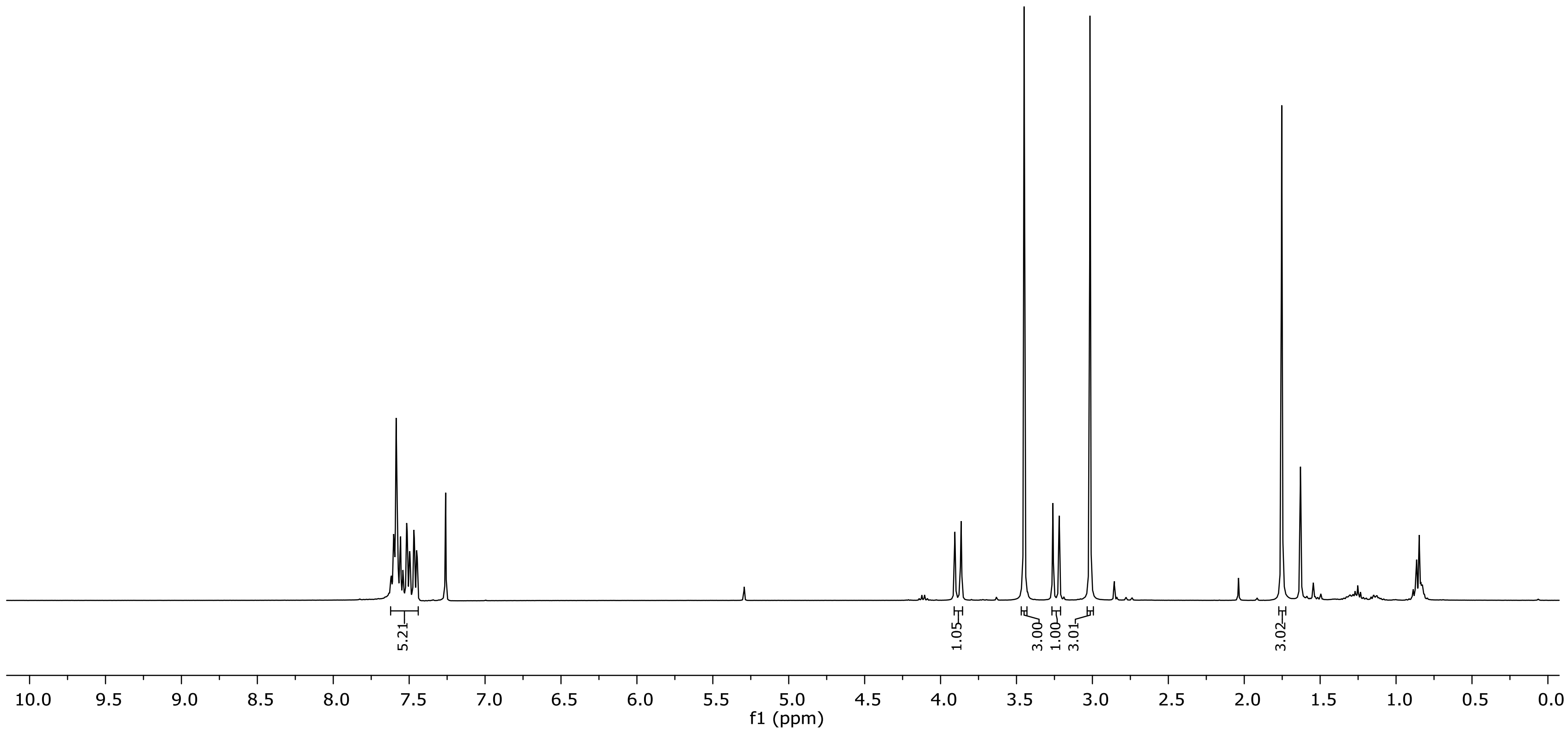
5.21
1.05
3.00
1.00
3.01
3.02
10.0 9.5 9.0 8.5 8.0 7.5 7.0 6.5 6.0 5.5 5.0 4.5 4.0 3.5 3.0 2.5 2.0 1.5 1.0 0.5 0.0
f1 (ppm)

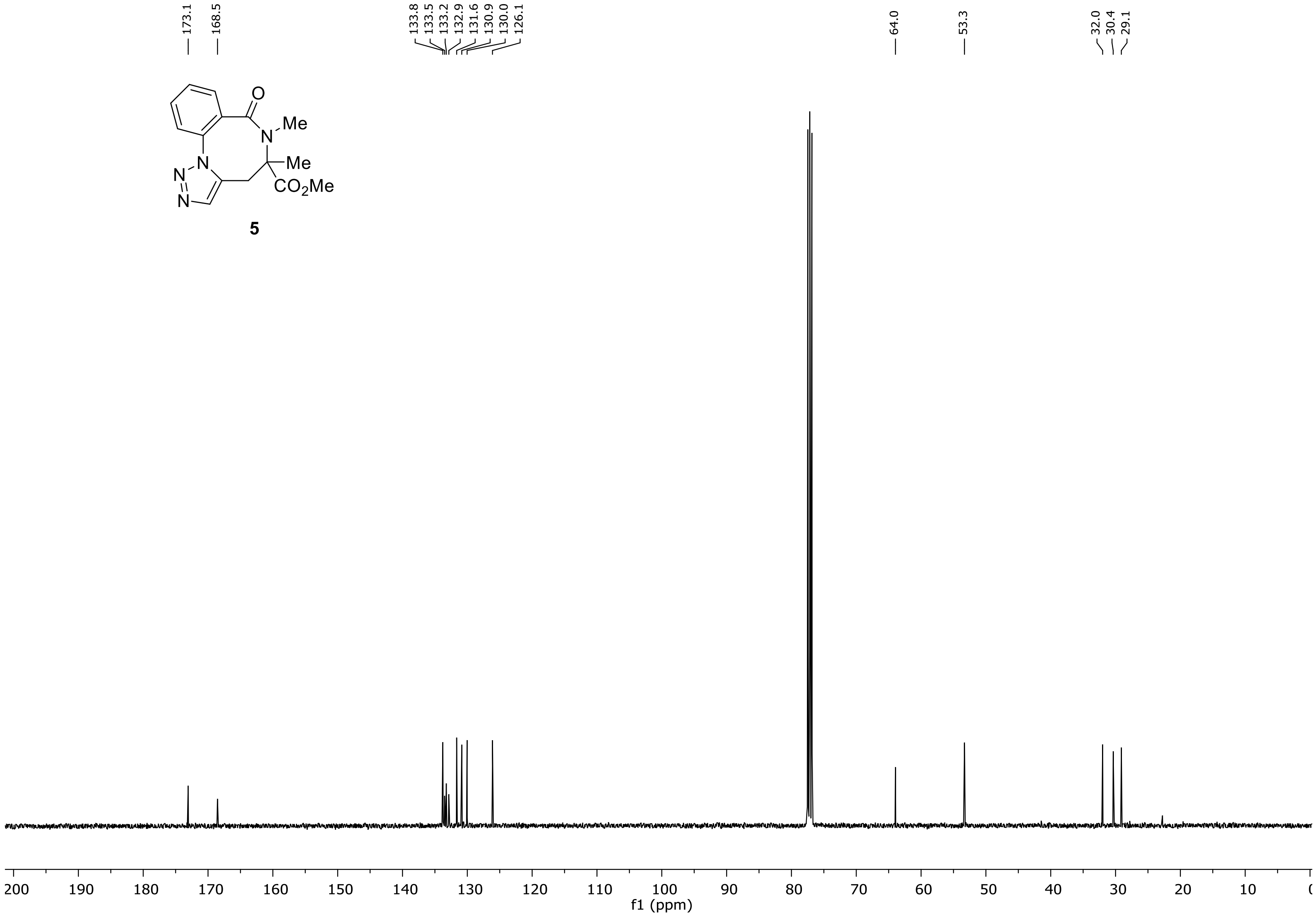
173.1
168.5
133.8
133.5
133.2
132.9
131.6
130.9
130.0
126.1
64.0
53.3
32.0
30.4
29.1
O
N
Me
Me
N
N
N
CO$_2$Me
5
200 190 180 170 160 150 140 130 120 110 100 90 80 70 60 50 40 30 20 10
f1 (ppm)

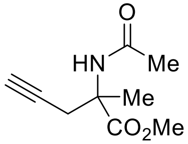
O
HN
Me
Me
CO_2Me

S6

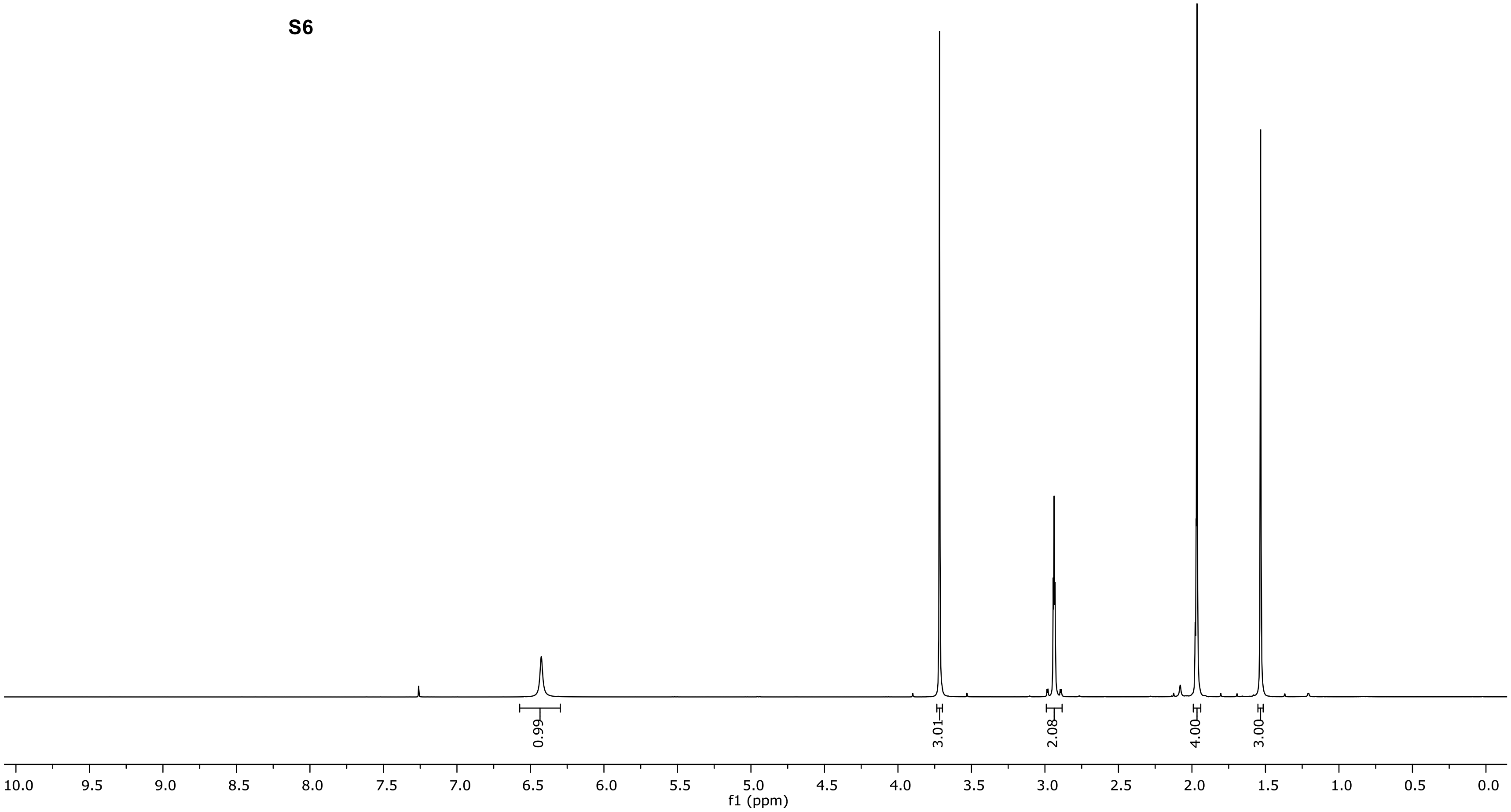
0.99
3.01
2.08
4.00
3.00
10.0 9.5 9.0 8.5 8.0 7.5 7.0 6.5 6.0 5.5 5.0 4.5 4.0 3.5 3.0 2.5 2.0 1.5 1.0 0.5 0.0
f1 (ppm)

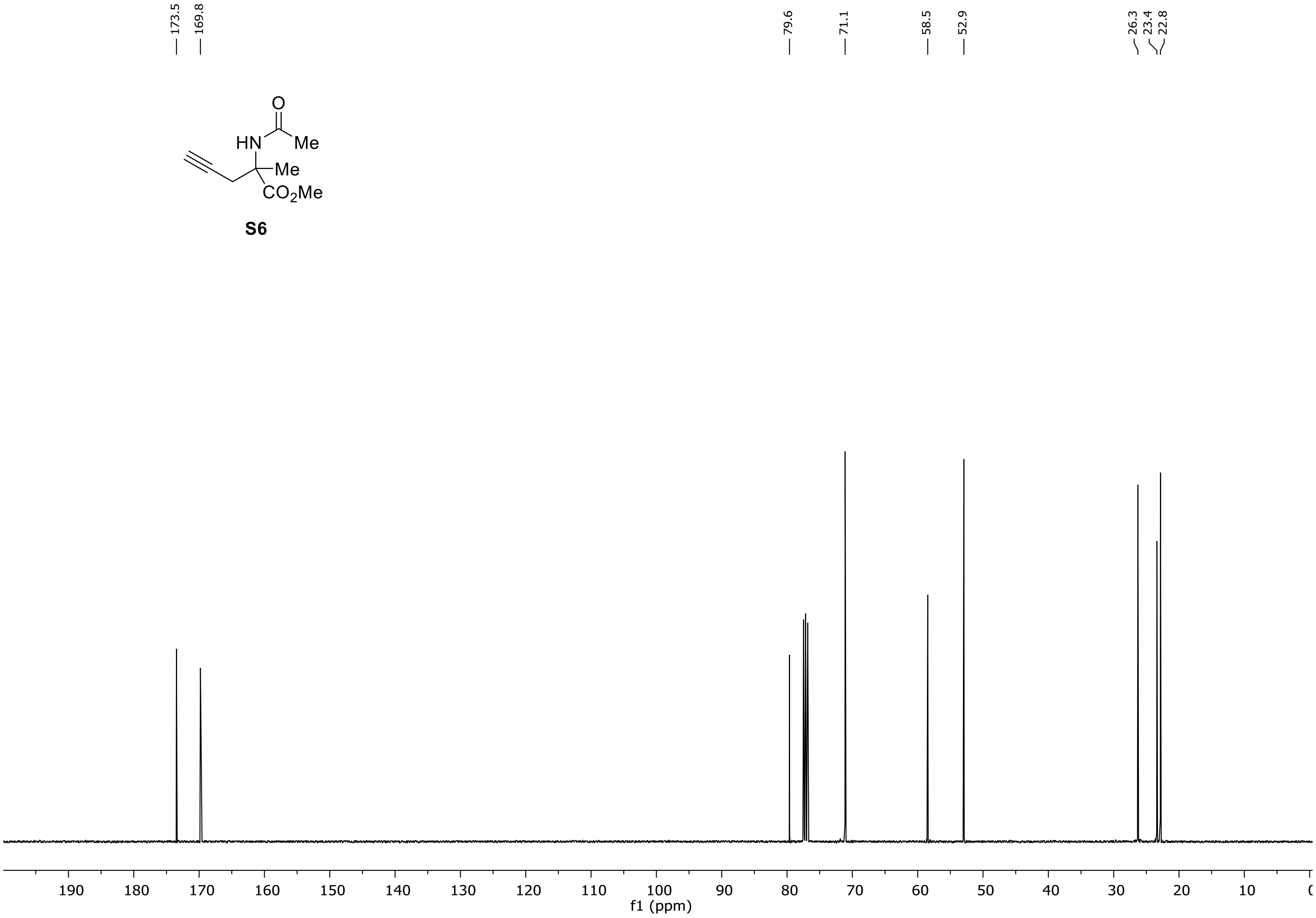

173.5
169.8
79.6
71.1
58.5
52.9
26.3
23.4
22.8
O
HN
Me
Me
CO2Me
S6
190 180 170 160 150 140 130 120 110 100 90 80 70 60 50 40 30 20 10
f1 (ppm)

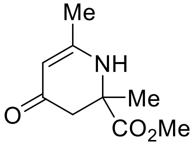
Me
NH
Me
O
CO_2Me

6

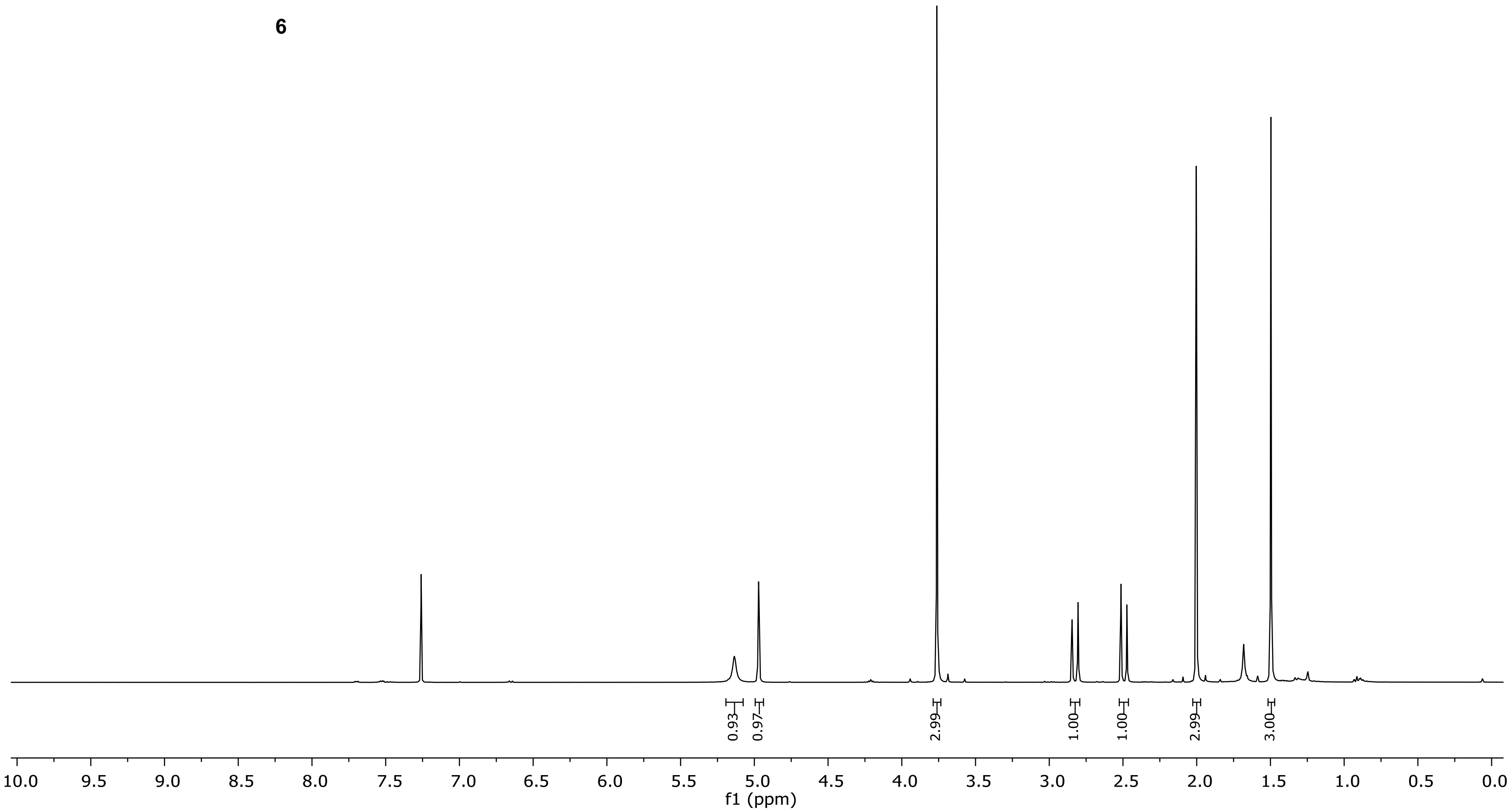
0.93
0.97
2.99
1.00
1.00
2.99
3.00
10.0
9.5
9.0
8.5
8.0
7.5
7.0
6.5
6.0
5.5
5.0
4.5
4.0
3.5
3.0
2.5
2.0
1.5
1.0
0.5
0.0
f1 (ppm)

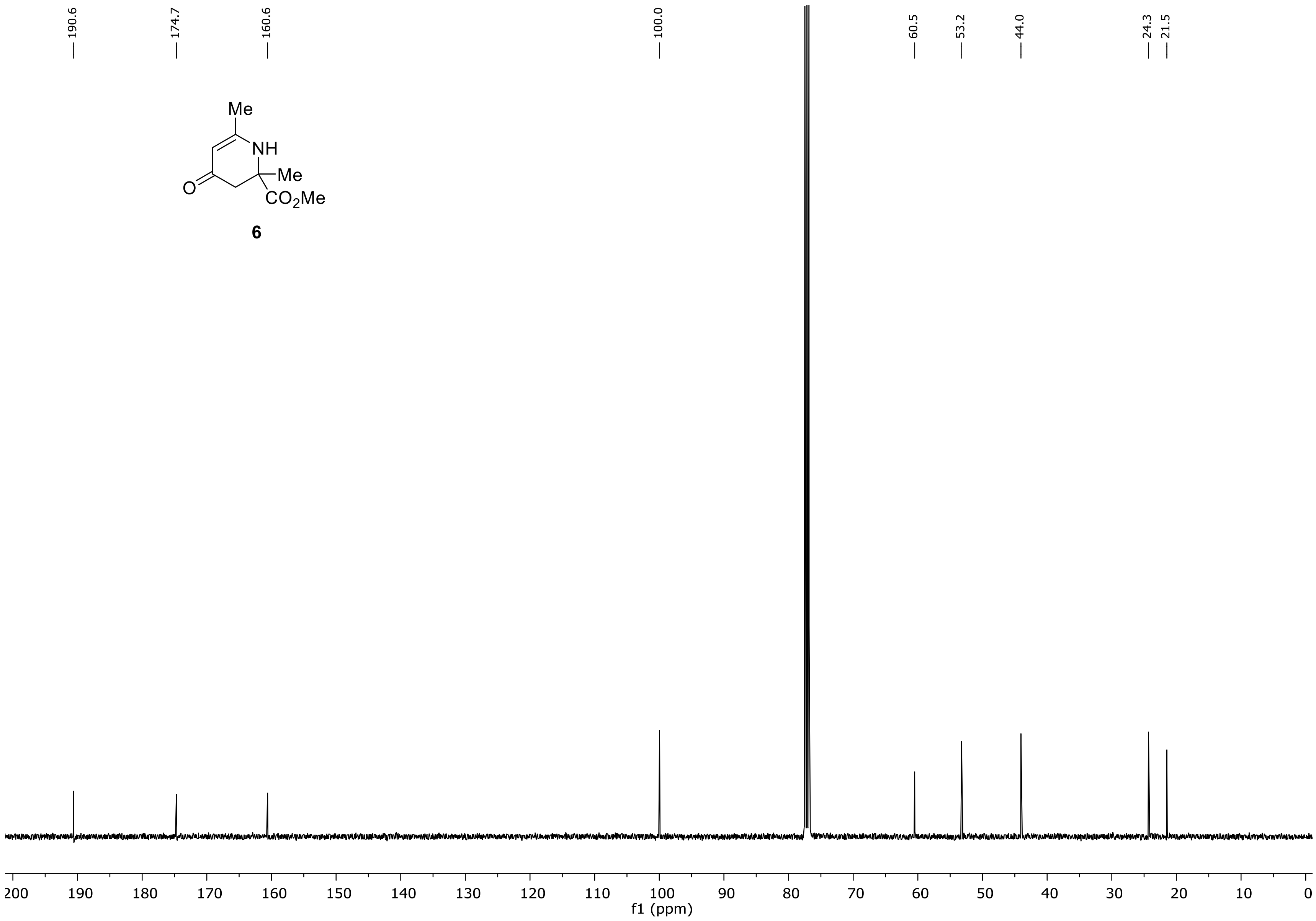
190.6
174.7
160.6
100.0
60.5
53.2
44.0
24.3
21.5
Me
NH
Me
O
CO2Me
6
200 190 180 170 160 150 140 130 120 110 100 90 80 70 60 50 40 30 20 10 0
f1 (ppm)

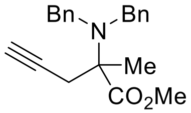
Bn
N
Bn
Me
CO2Me

S7

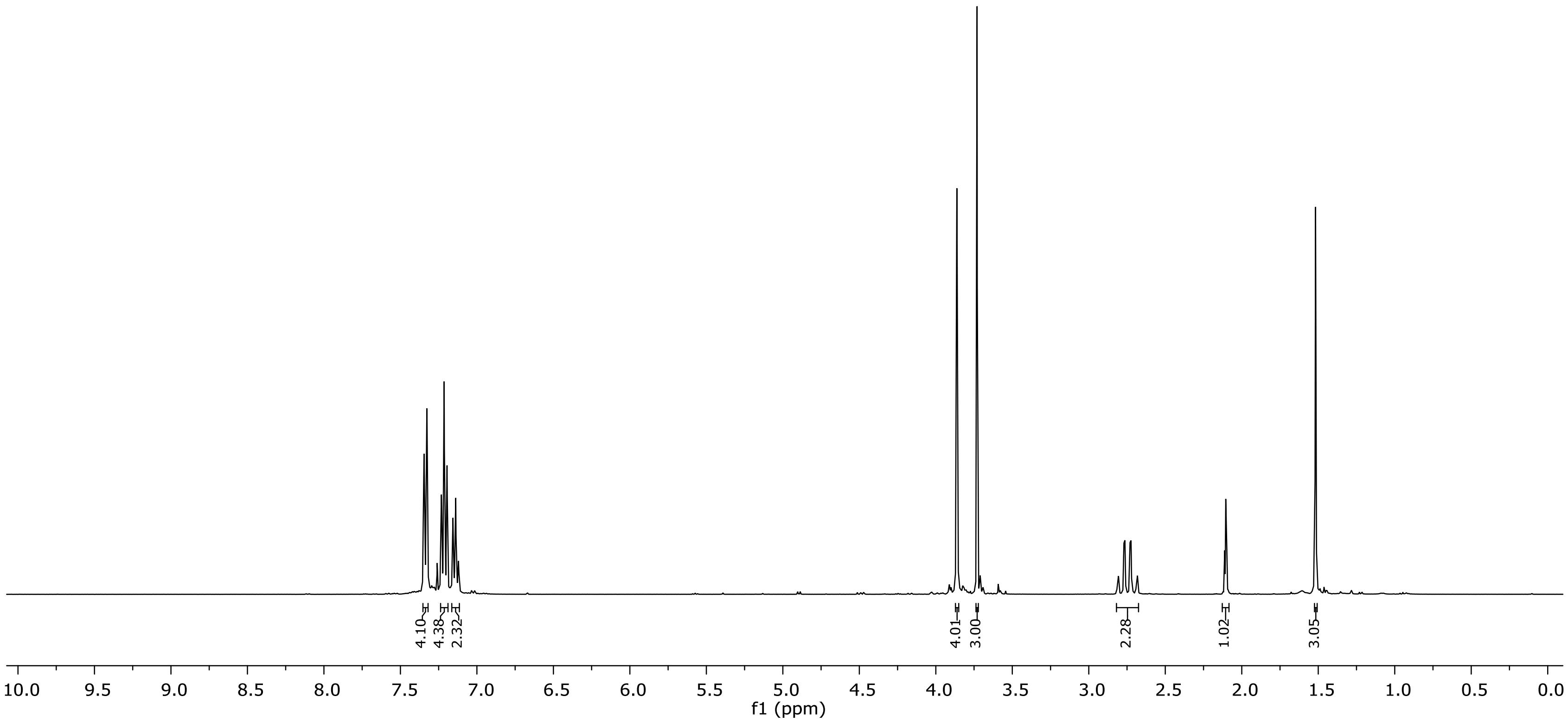
4.10
4.38
2.32
4.01
3.00
2.28
1.02
3.05
10.0 9.5 9.0 8.5 8.0 7.5 7.0 6.5 6.0 5.5 5.0 4.5 4.0 3.5 3.0 2.5 2.0 1.5 1.0 0.5 0.0
f1 (ppm)

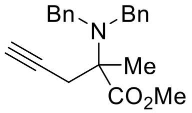
Bn
N
Bn
Me
CO2Me

S7

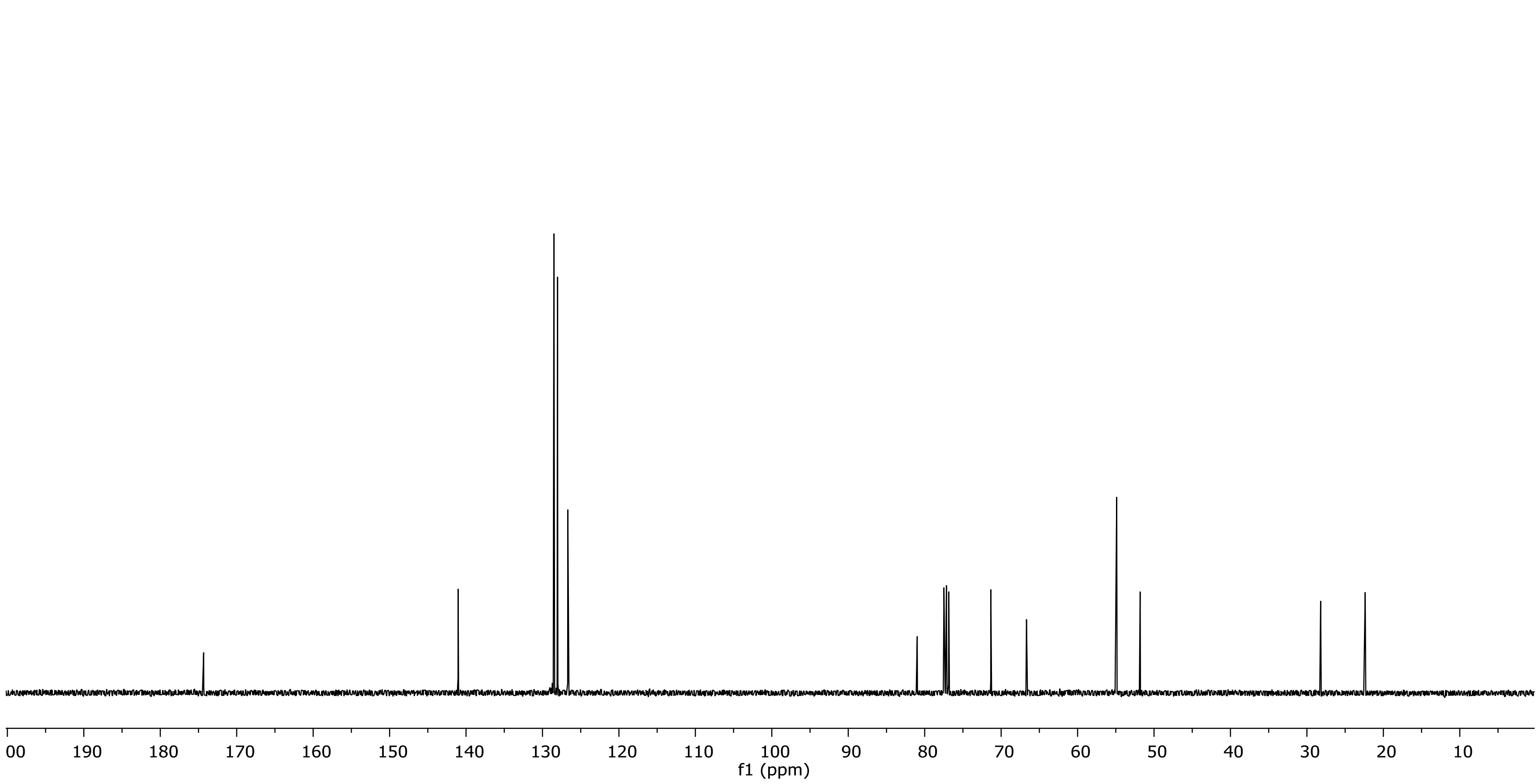
174.3
141.0
128.5
128.0
126.7
81.0
71.3
66.7
54.9
51.8
28.2
22.4
00
190
180
170
160
150
140
130
120
110
100
90
80
70
60
50
40
30
20
10
f1 (ppm)

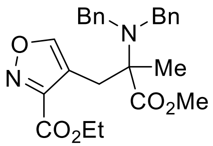
Bn
N
Bn
O
N
Me
CO2Me
CO2Et

S8

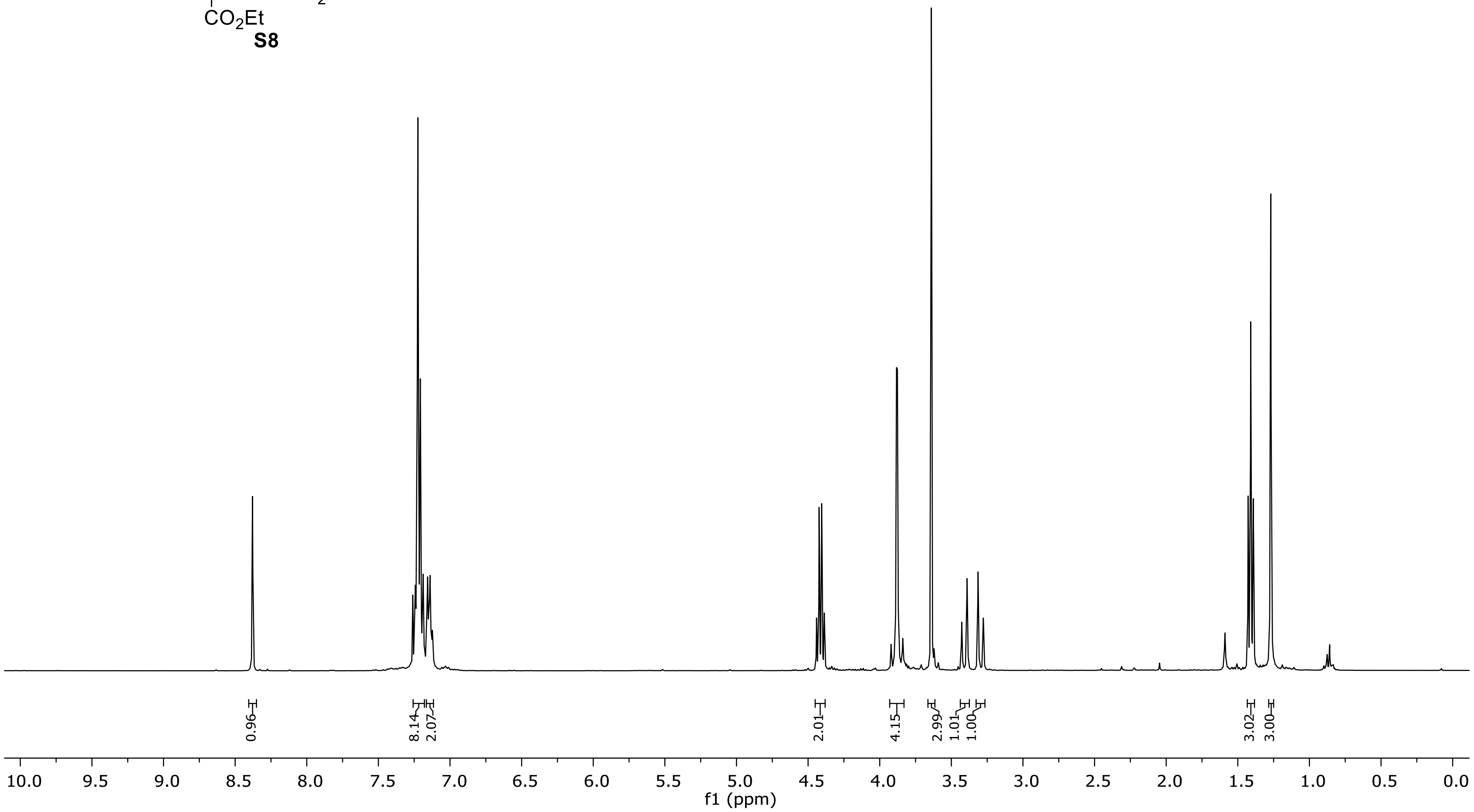
0.96
8.14
2.07
2.01
4.15
2.99
1.01
1.00
3.02
3.00
10.0 9.5 9.0 8.5 8.0 7.5 7.0 6.5 6.0 5.5 5.0 4.5 4.0 3.5 3.0 2.5 2.0 1.5 1.0 0.5 0.0
f1 (ppm)

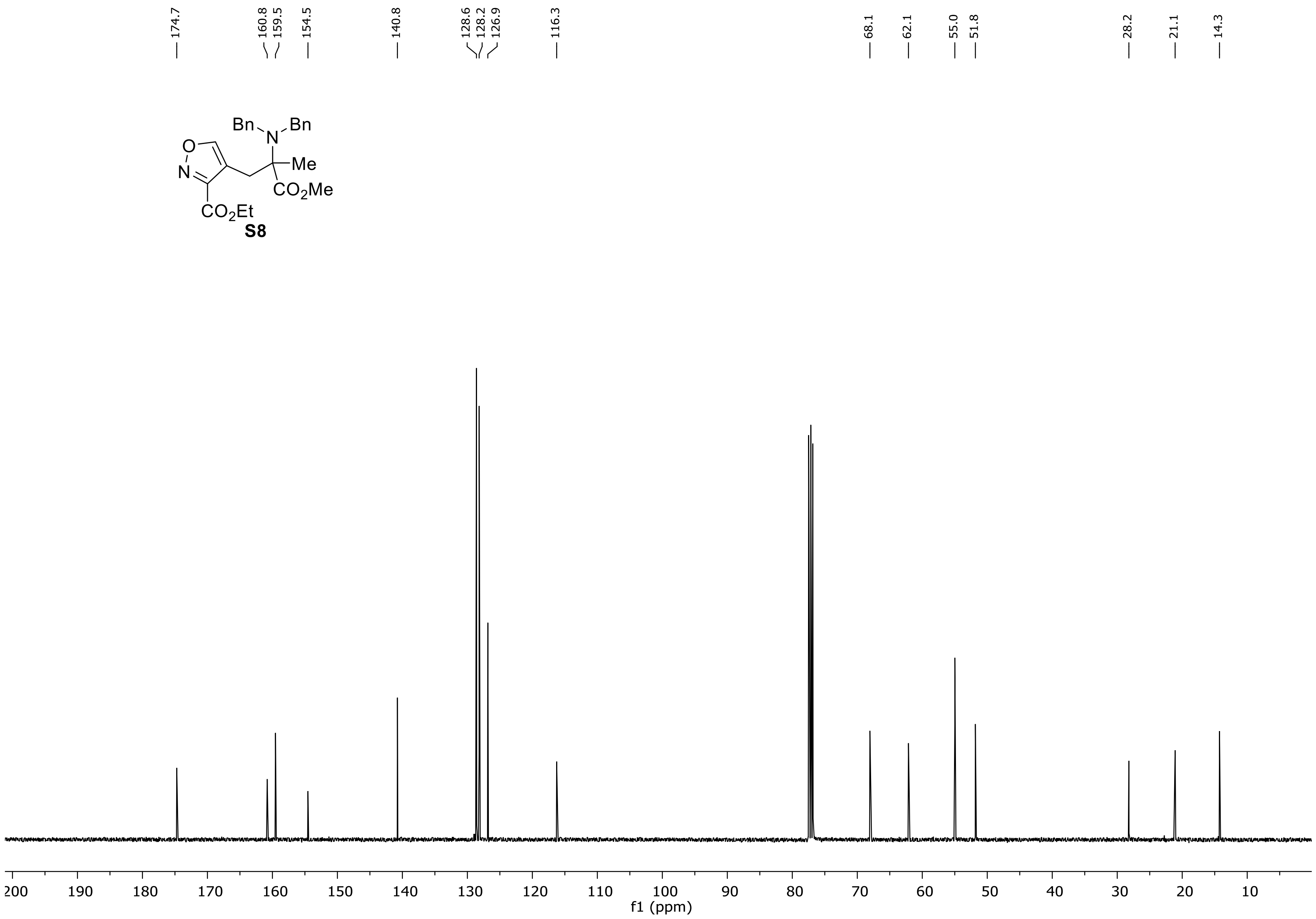
174.7
160.8
159.5
154.5
140.8
128.6
128.2
126.9
116.3
68.1
62.1
55.0
51.8
28.2
21.1
14.3
Bn
N
Bn
O
N
Me
CO2Me
CO2Et
S8
200 190 180 170 160 150 140 130 120 110 100 90 80 70 60 50 40 30 20 10
f1 (ppm)

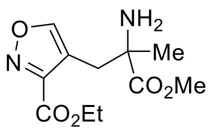

7

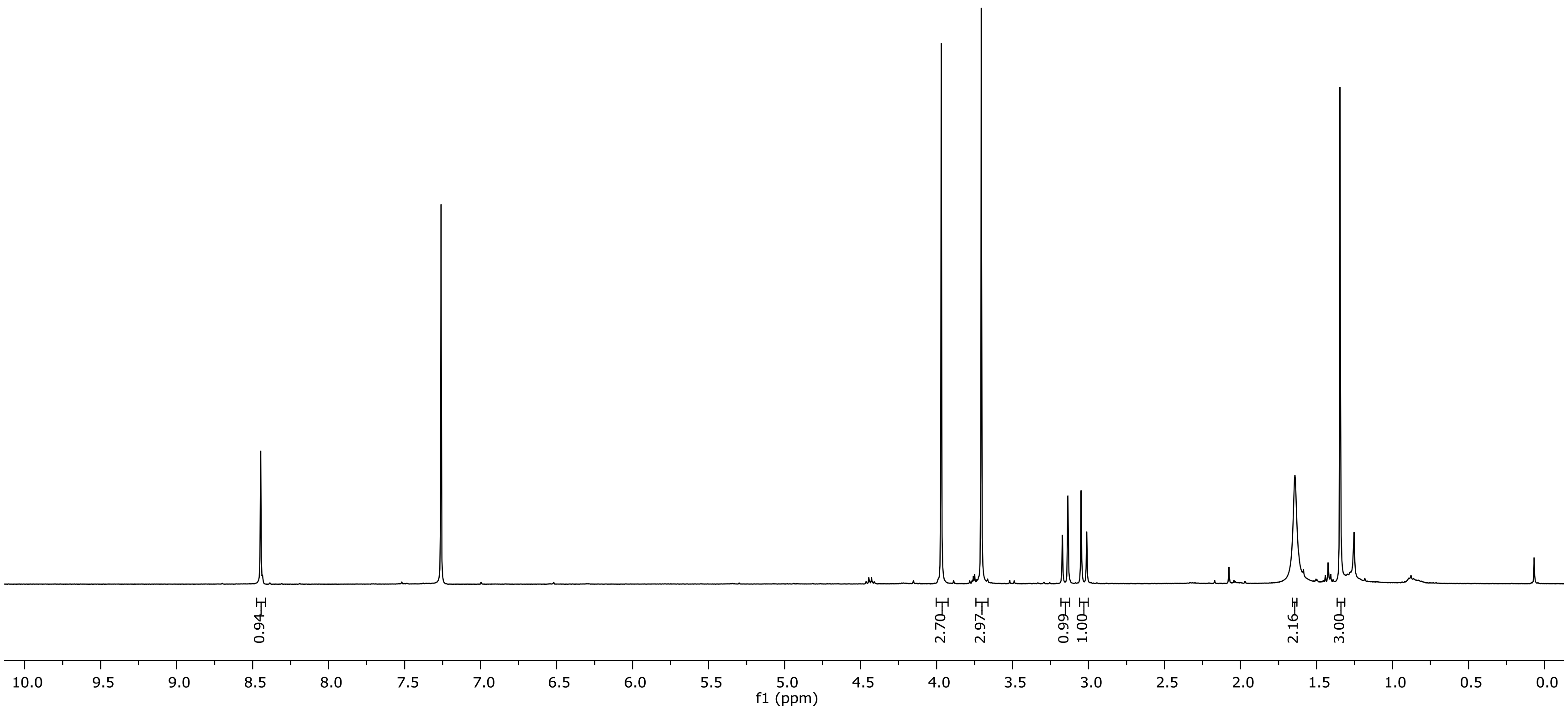
0.94
2.70
2.97
0.99
1.00
2.16
3.00
10.0 9.5 9.0 8.5 8.0 7.5 7.0 6.5 6.0 5.5 5.0 4.5 4.0 3.5 3.0 2.5 2.0 1.5 1.0 0.5 0.0
f1 (ppm)

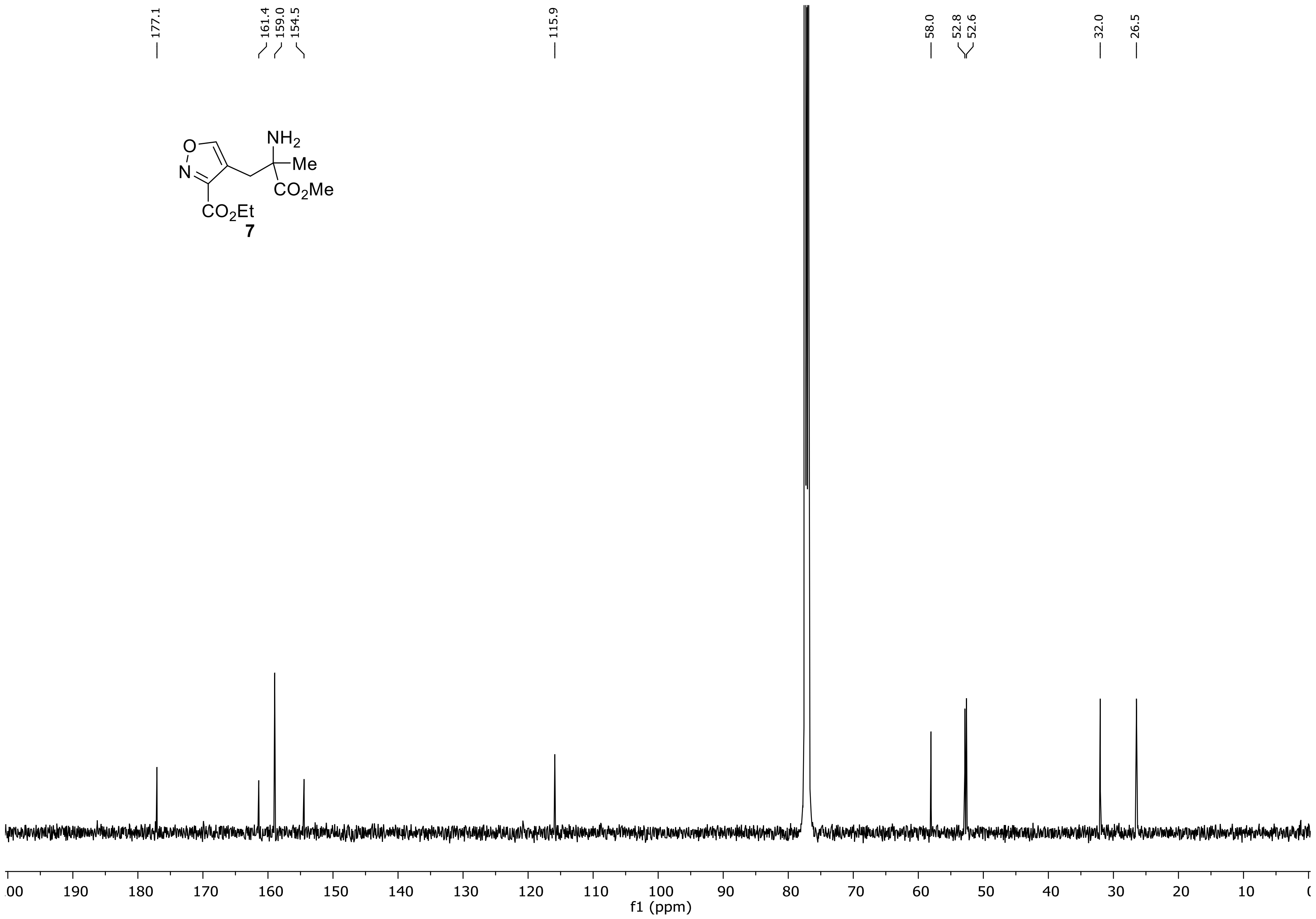

177.1
161.4
159.0
154.5
115.9
58.0
52.8
52.6
32.0
26.5
O
N
NH_2
Me
CO_2Me
CO_2Et
7
190 180 170 160 150 140 130 120 110 100 90 80 70 60 50 40 30 20 10
f1 (ppm)

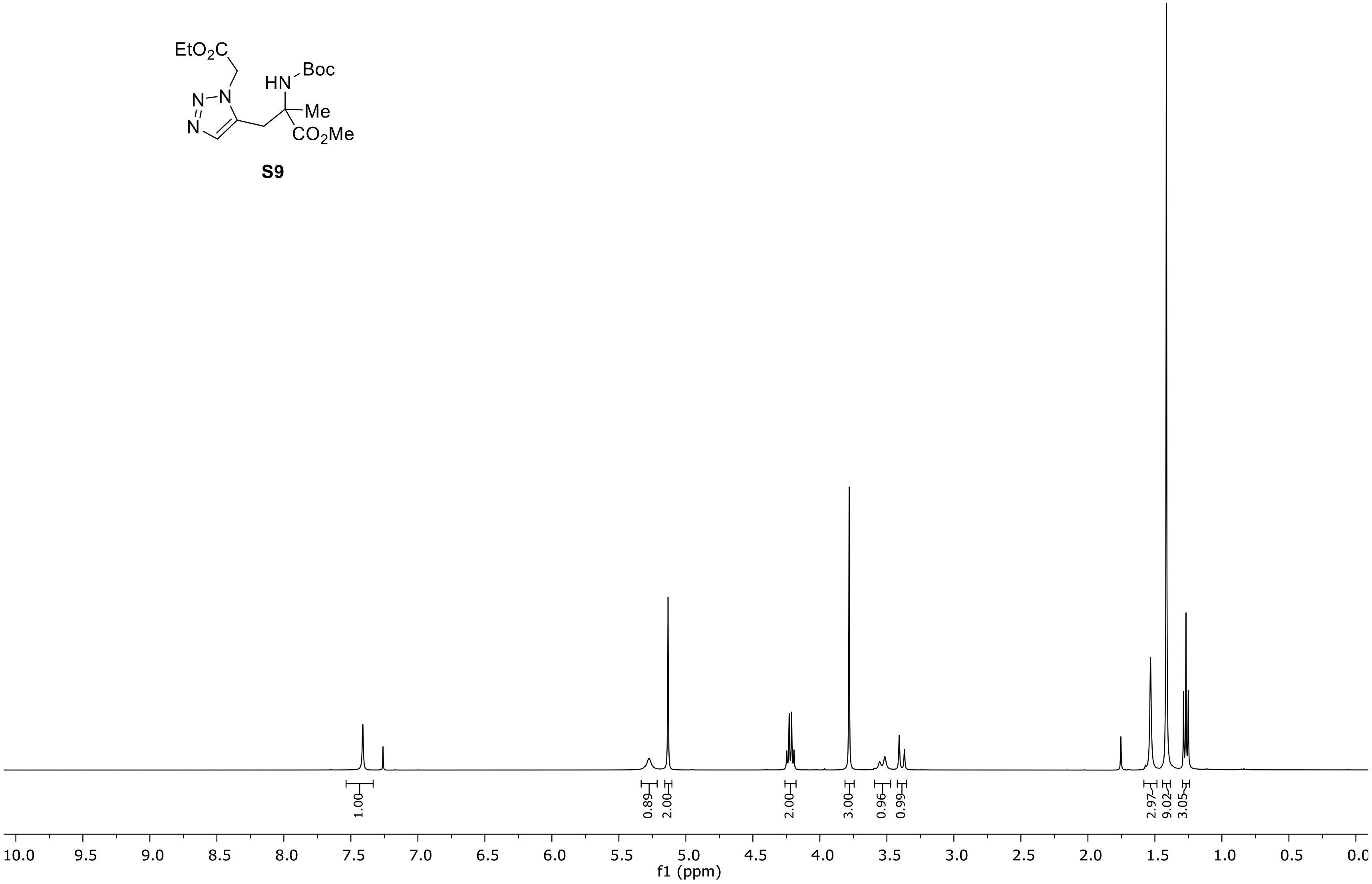
EtO2C
HN
Boc
N
N
N
Me
CO2Me
S9
1.00
0.89
2.00
2.00
3.00
0.96
0.99
2.97
9.02
3.05
10.0
9.5
9.0
8.5
8.0
7.5
7.0
6.5
6.0
5.5
5.0
4.5
4.0
3.5
3.0
2.5
2.0
1.5
1.0
0.5
0.0
f1 (ppm)

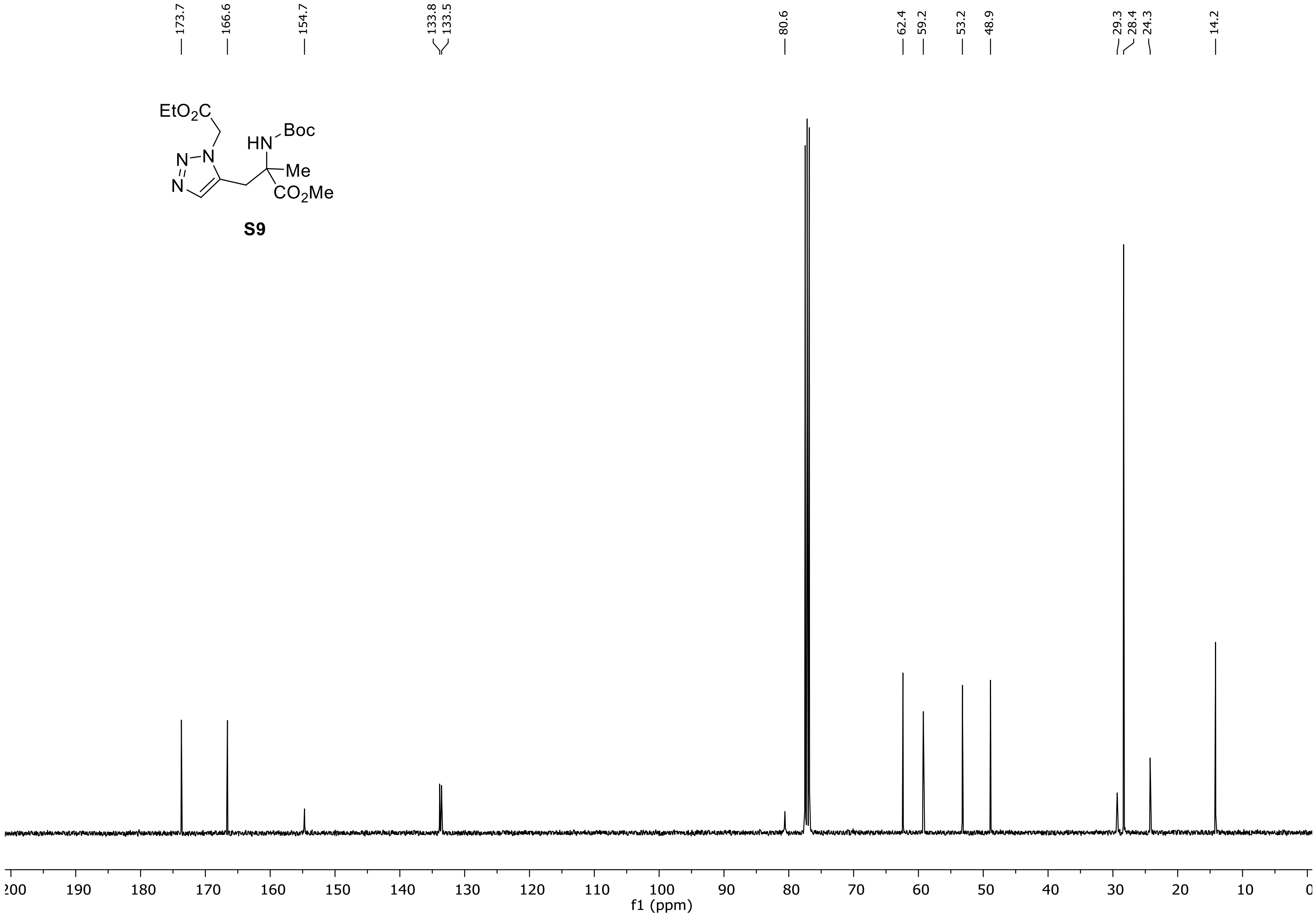
173.7
166.6
154.7
133.8
133.5
80.6
62.4
59.2
53.2
48.9
29.3
28.4
24.3
14.2
EtO2C
HN
Boc
N
Me
CO2Me
S9
200 190 180 170 160 150 140 130 120 110 100 90 80 70 60 50 40 30 20 10 0
f1 (ppm)

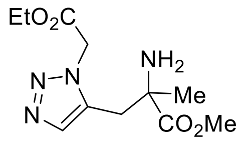
EtO2C
NH2
N
N
N
Me
CO2Me

8

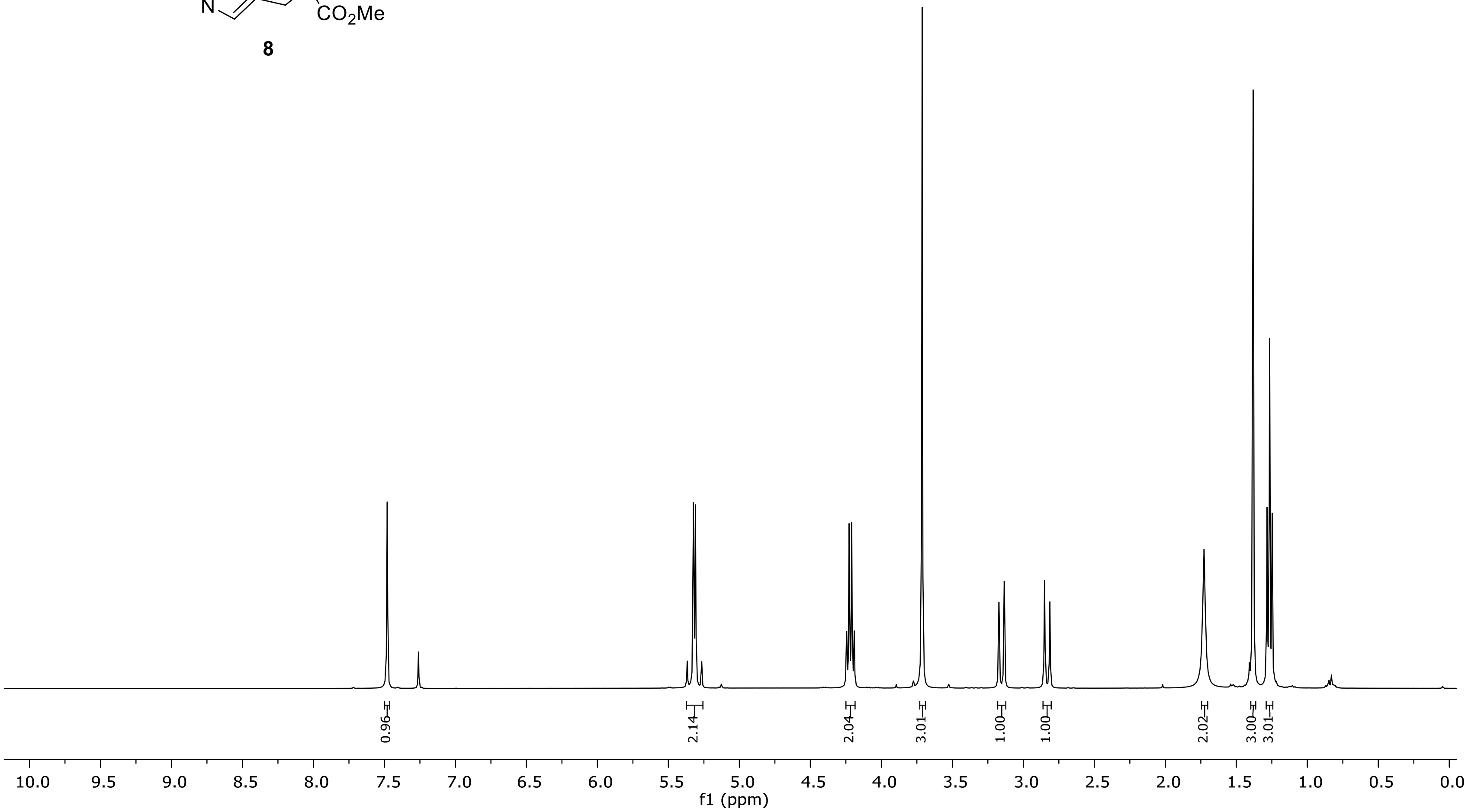
0.96
2.14
2.04
3.01
1.00
1.00
2.02
3.00
3.01
10.0 9.5 9.0 8.5 8.0 7.5 7.0 6.5 6.0 5.5 5.0 4.5 4.0 3.5 3.0 2.5 2.0 1.5 1.0 0.5 0.0
f1 (ppm)

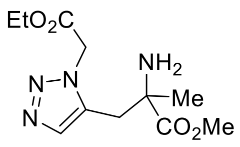

8

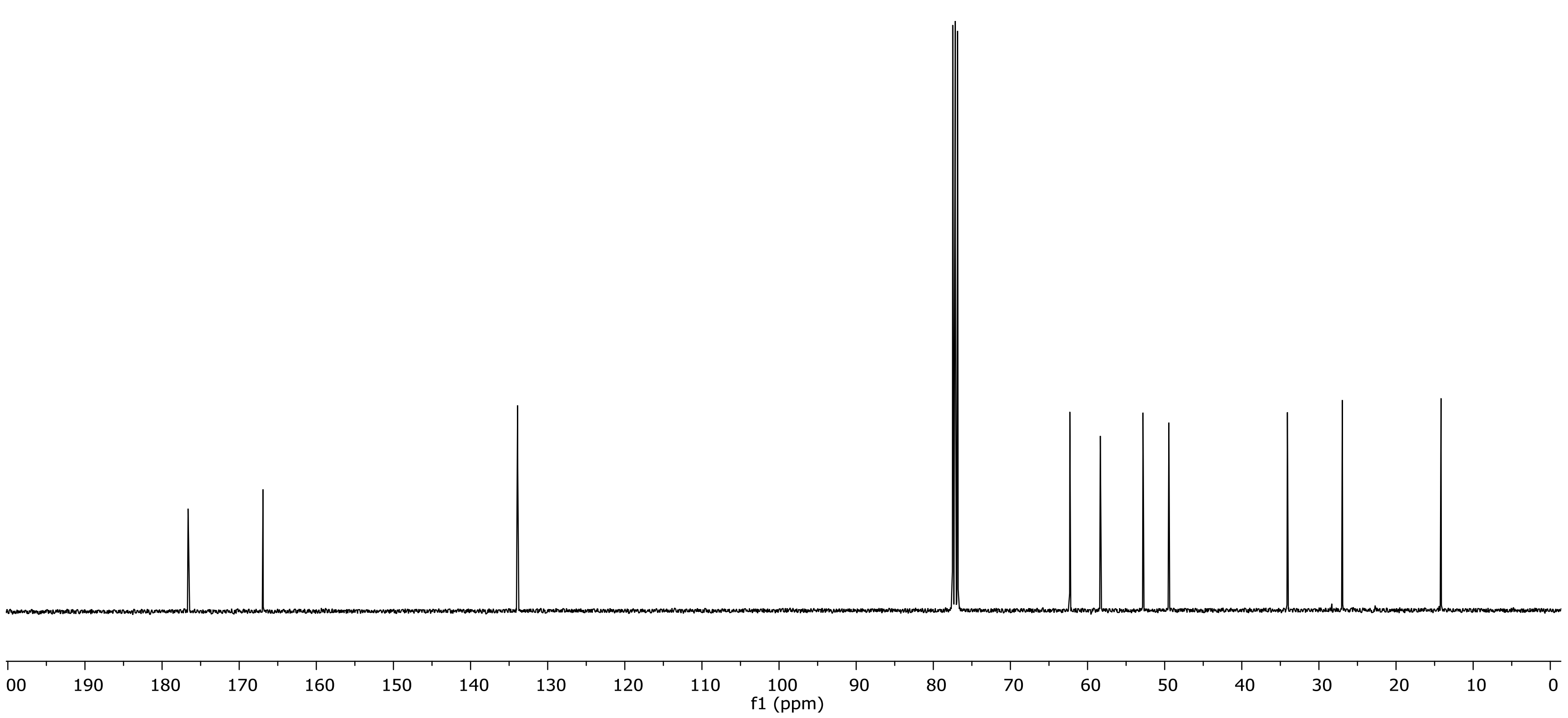
176.6
166.9
133.9
133.9
62.3
58.3
52.8
49.5
34.1
27.0
14.2
00
190
180
170
160
150
140
130
120
110
100
90
80
70
60
50
40
30
20
10
0
f1 (ppm)

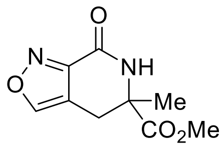
O
N
NH
O
Me
CO_2Me

9

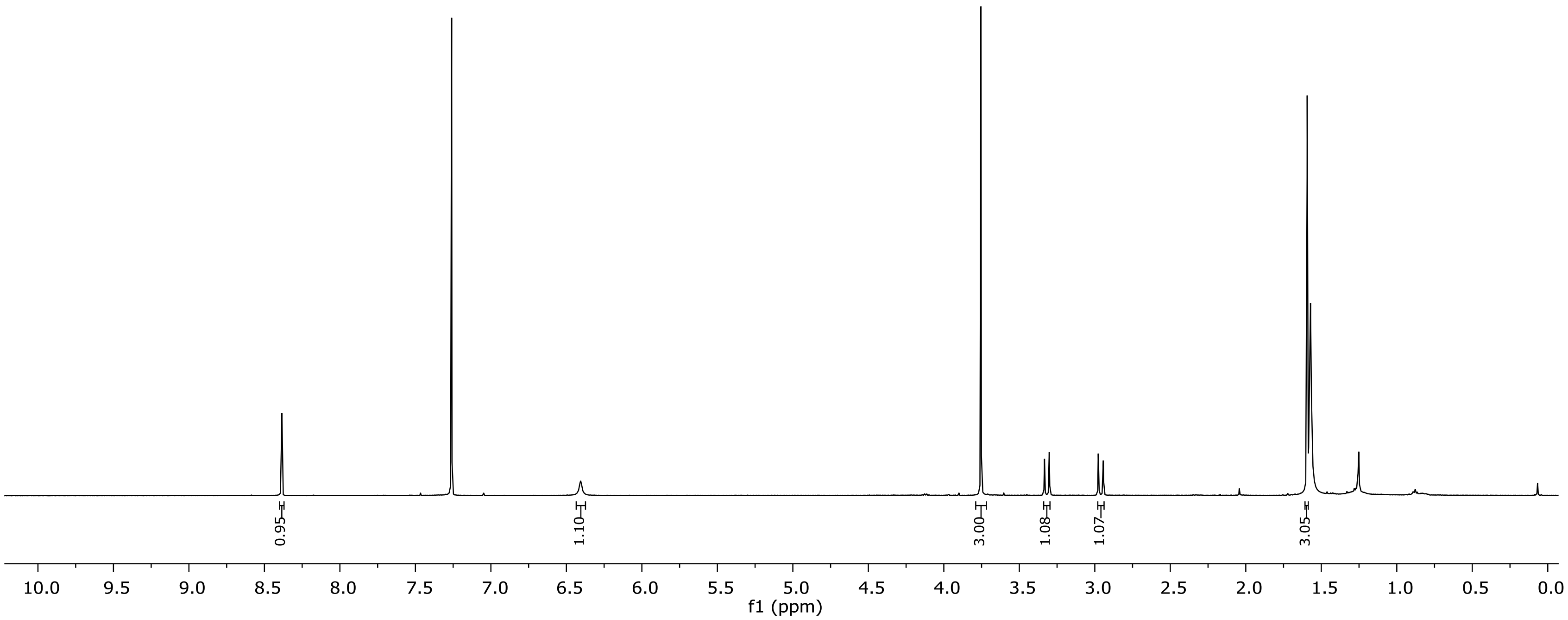
0.95
1.10
3.00
1.08
1.07
3.05
10.0 9.5 9.0 8.5 8.0 7.5 7.0 6.5 6.0 5.5 5.0 4.5 4.0 3.5 3.0 2.5 2.0 1.5 1.0 0.5 0.0
f1 (ppm)

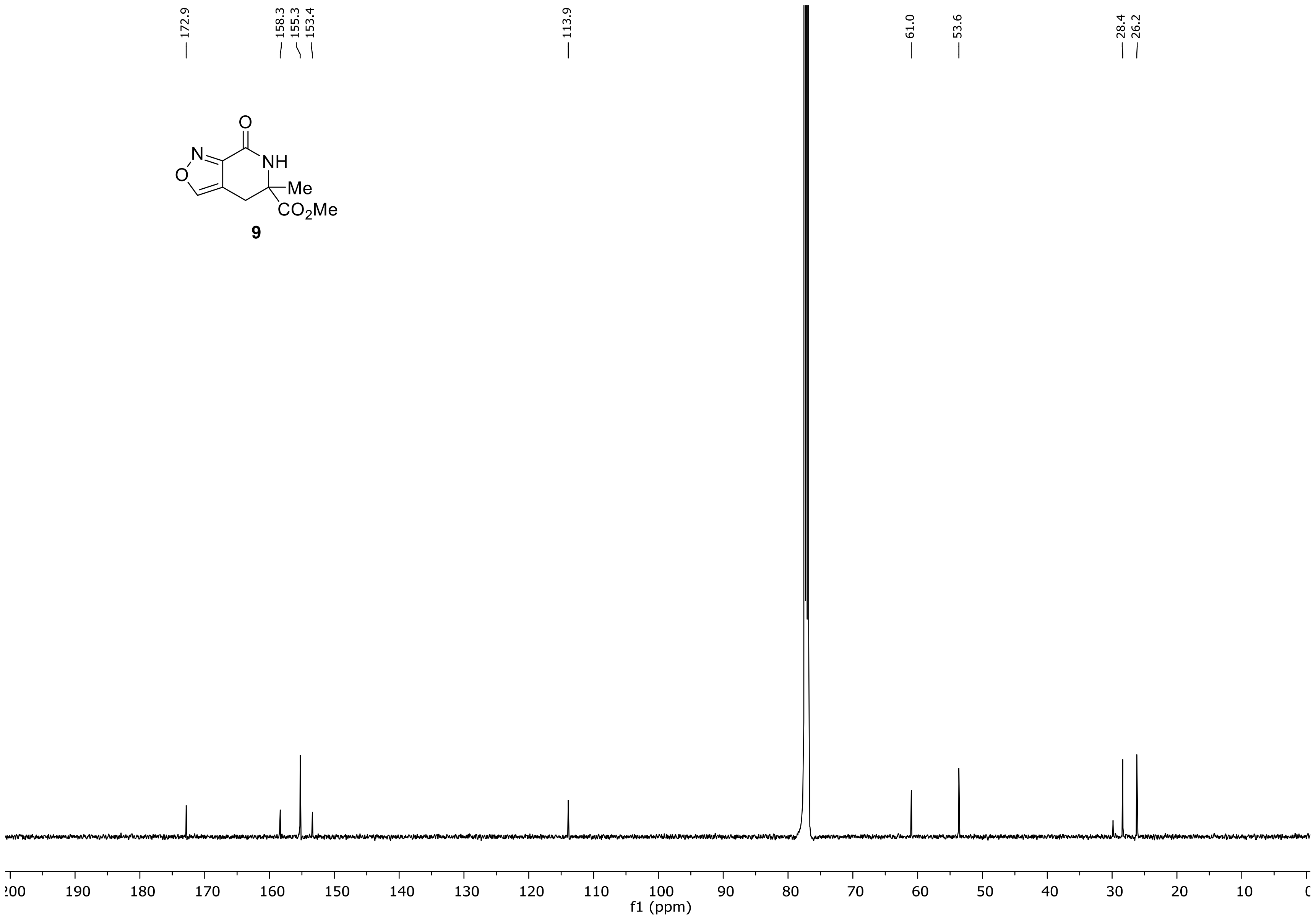

172.9
158.3
155.3
153.4
113.9
61.0
53.6
28.4
26.2
O
N
NH
O
Me
CO2Me
9
200
190
180
170
160
150
140
130
120
110
100
90
80
70
60
50
40
30
20
10
0
f1 (ppm)

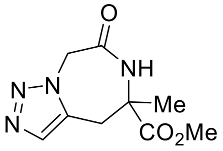

10

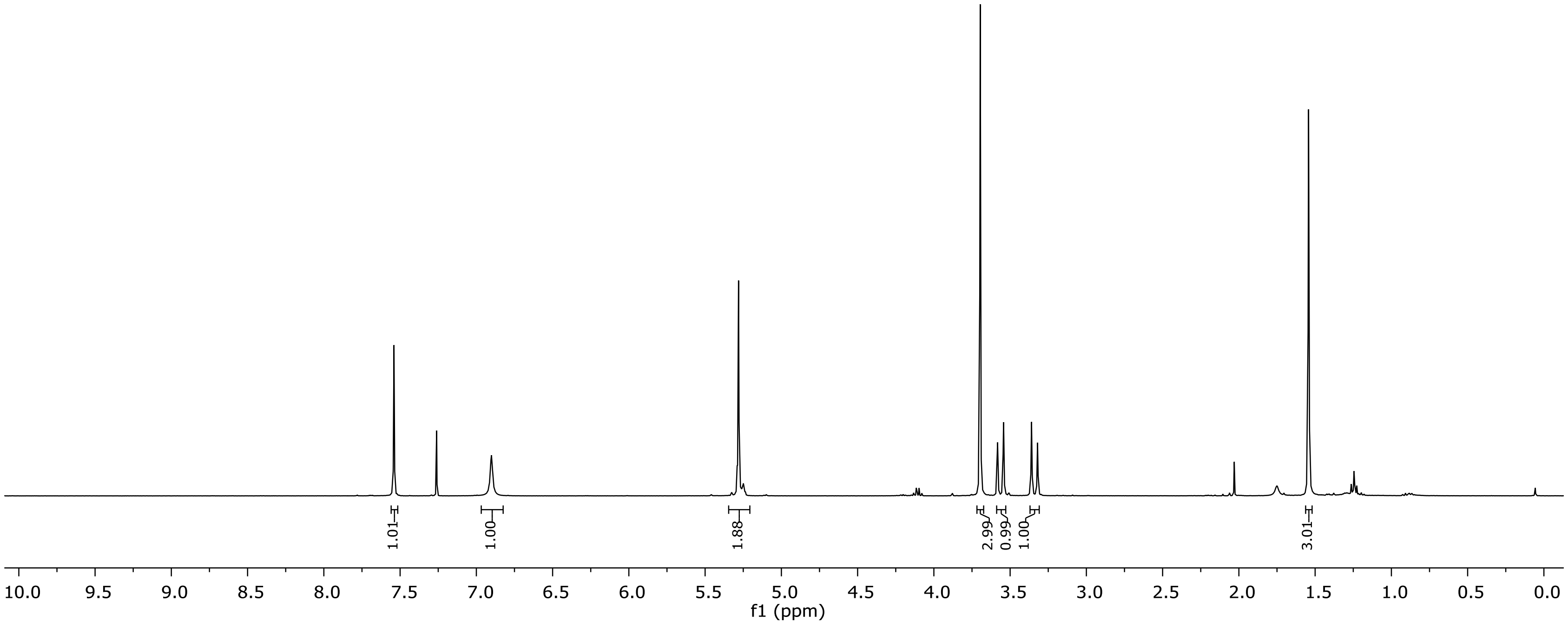
1.01
1.00
1.88
2.99
0.99
1.00
3.01
10.0 9.5 9.0 8.5 8.0 7.5 7.0 6.5 6.0 5.5 5.0 4.5 4.0 3.5 3.0 2.5 2.0 1.5 1.0 0.5 0.0
f1 (ppm)

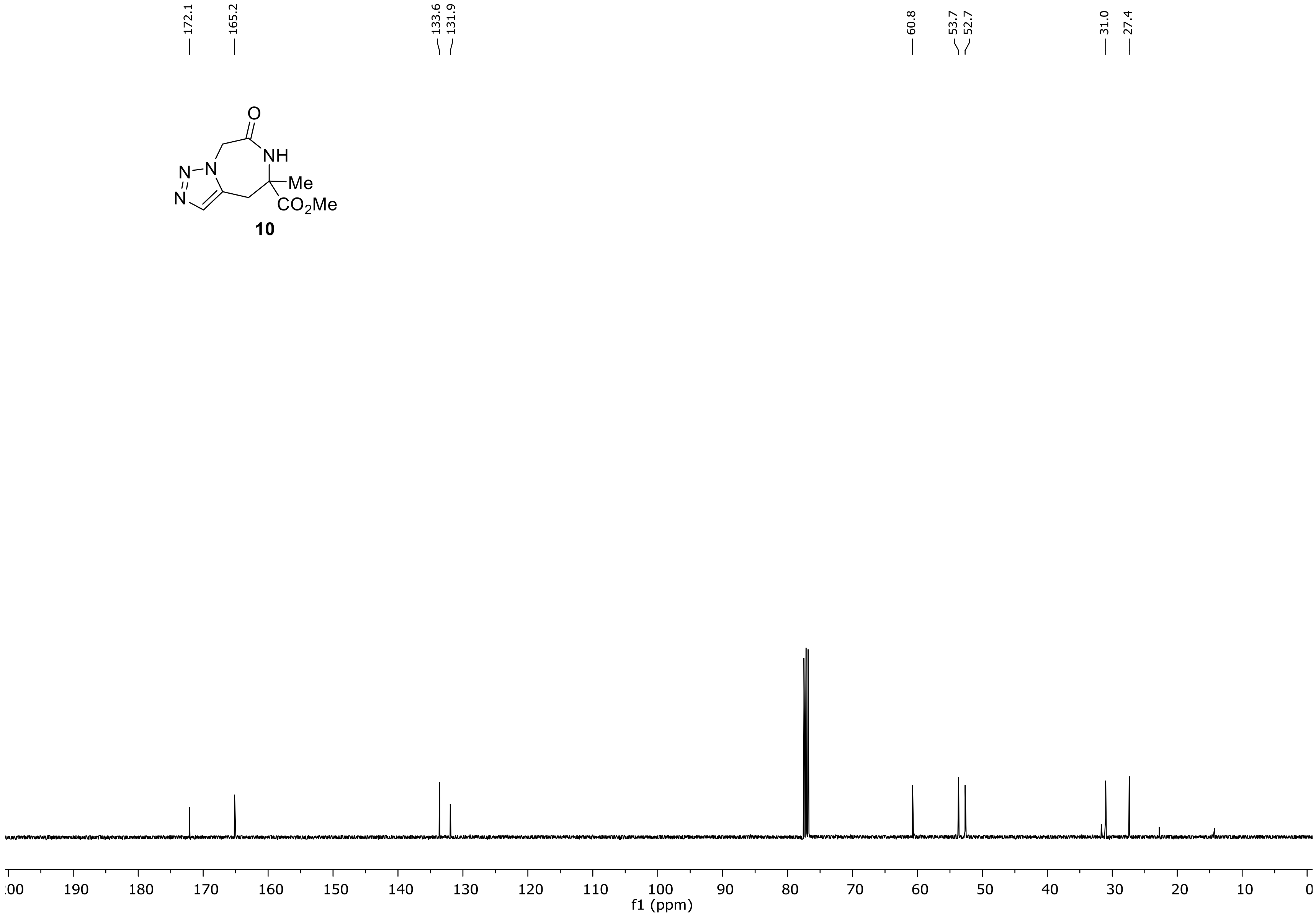
172.1
165.2
133.6
131.9
60.8
53.7
52.7
31.0
27.4
O
NH
N
N
N
Me
CO_2Me
10
200
190
180
170
160
150
140
130
120
110
100
90
80
70
60
50
40
30
20
10
0
f1 (ppm)

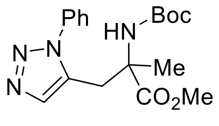
Ph
HN
Boc
N
N
N
Me
CO_2Me

S10

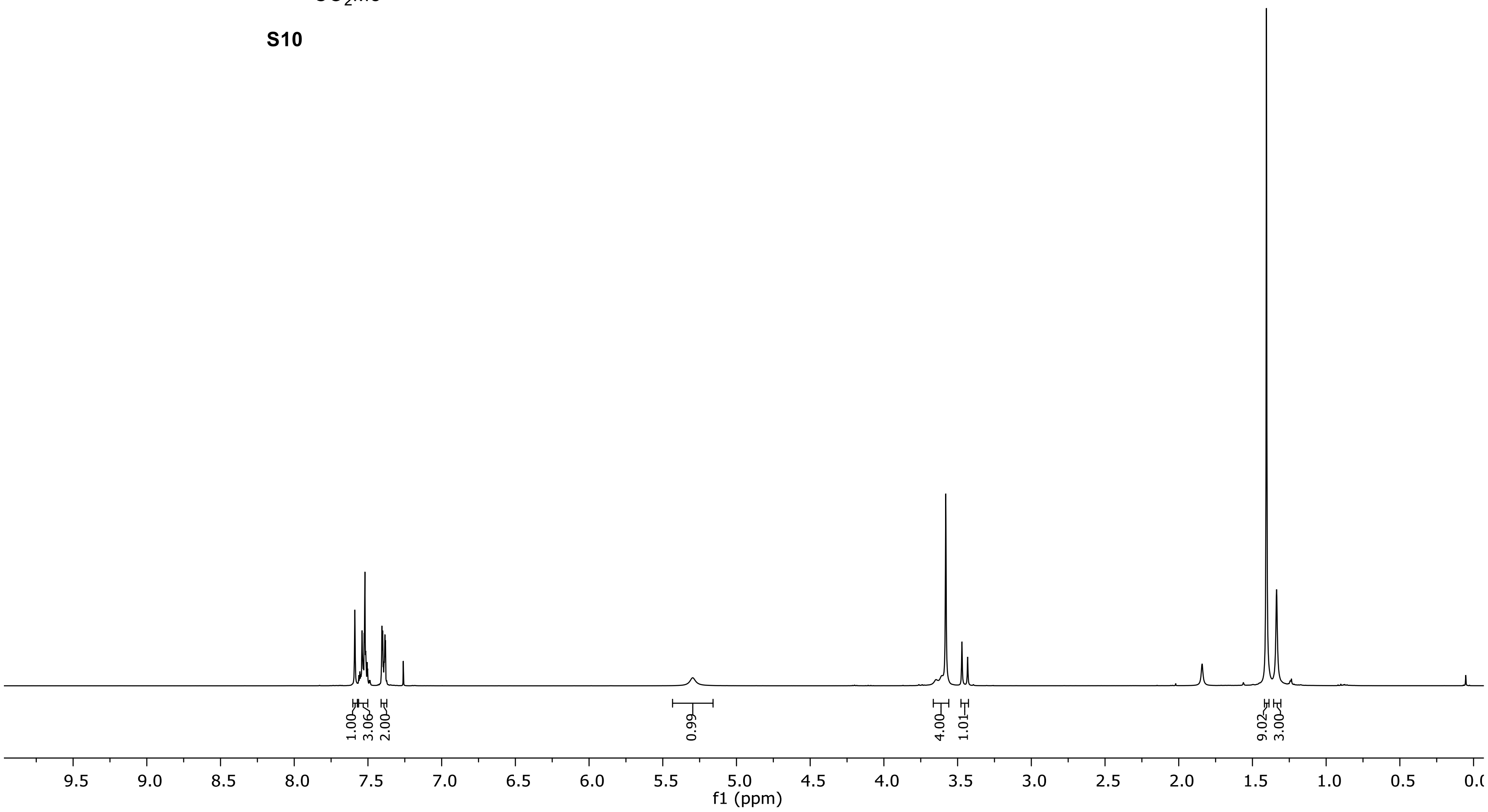
1.00
3.06
2.00
0.99
4.00
1.01
9.02
3.00
9.5
9.0
8.5
8.0
7.5
7.0
6.5
6.0
5.5
5.0
4.5
4.0
3.5
3.0
2.5
2.0
1.5
1.0
0.5
f1 (ppm)

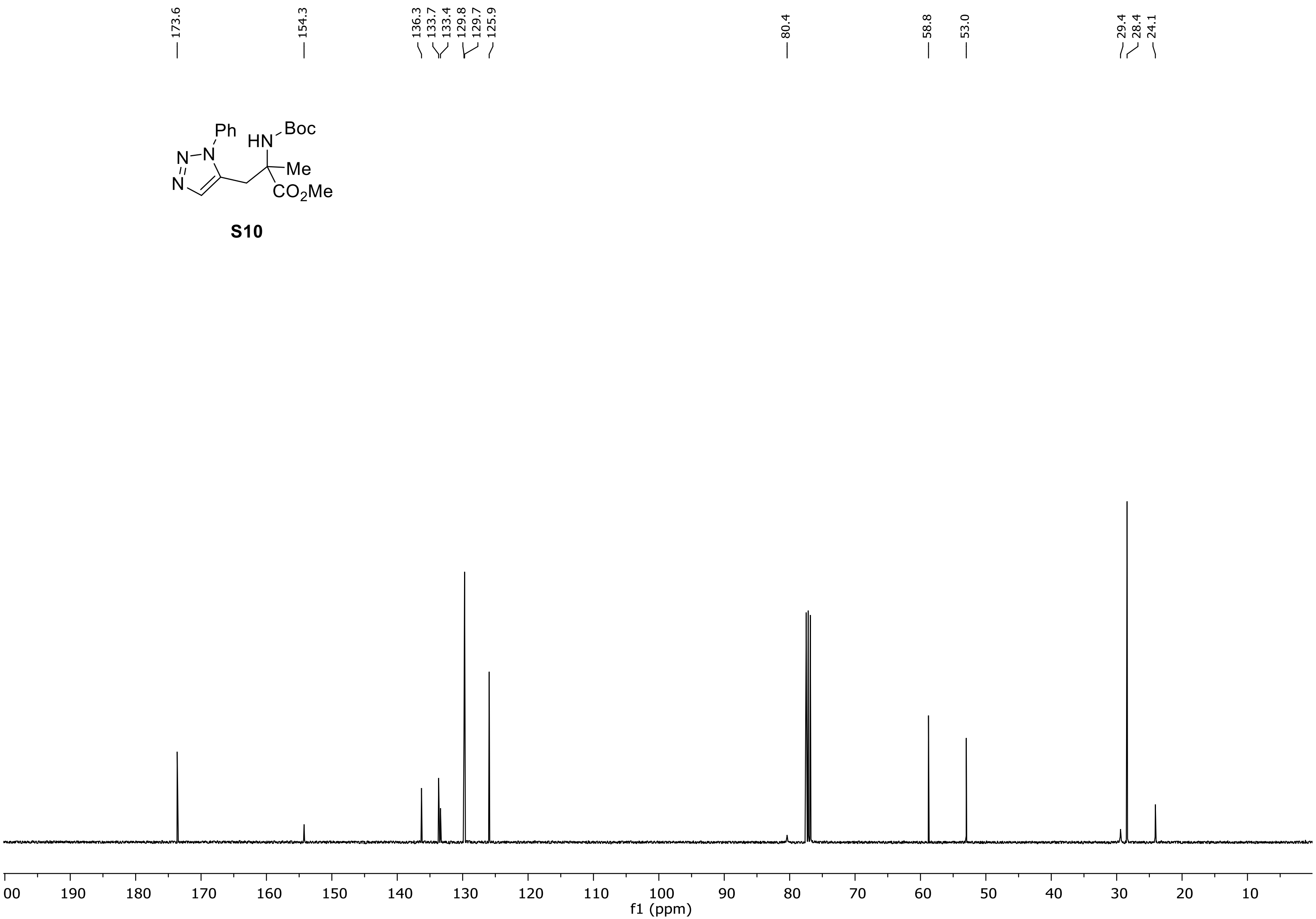
173.6
154.3
136.3
133.7
133.4
129.8
129.7
125.9
80.4
58.8
53.0
29.4
28.4
24.1
00
190
180
170
160
150
140
130
120
110
100
90
80
70
60
50
40
30
20
10
f1 (ppm)
Ph
HN
Boc
N
N
N
Me
CO2Me

S10

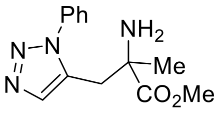
Ph
NH2
N
N
N
Me
CO2Me

11

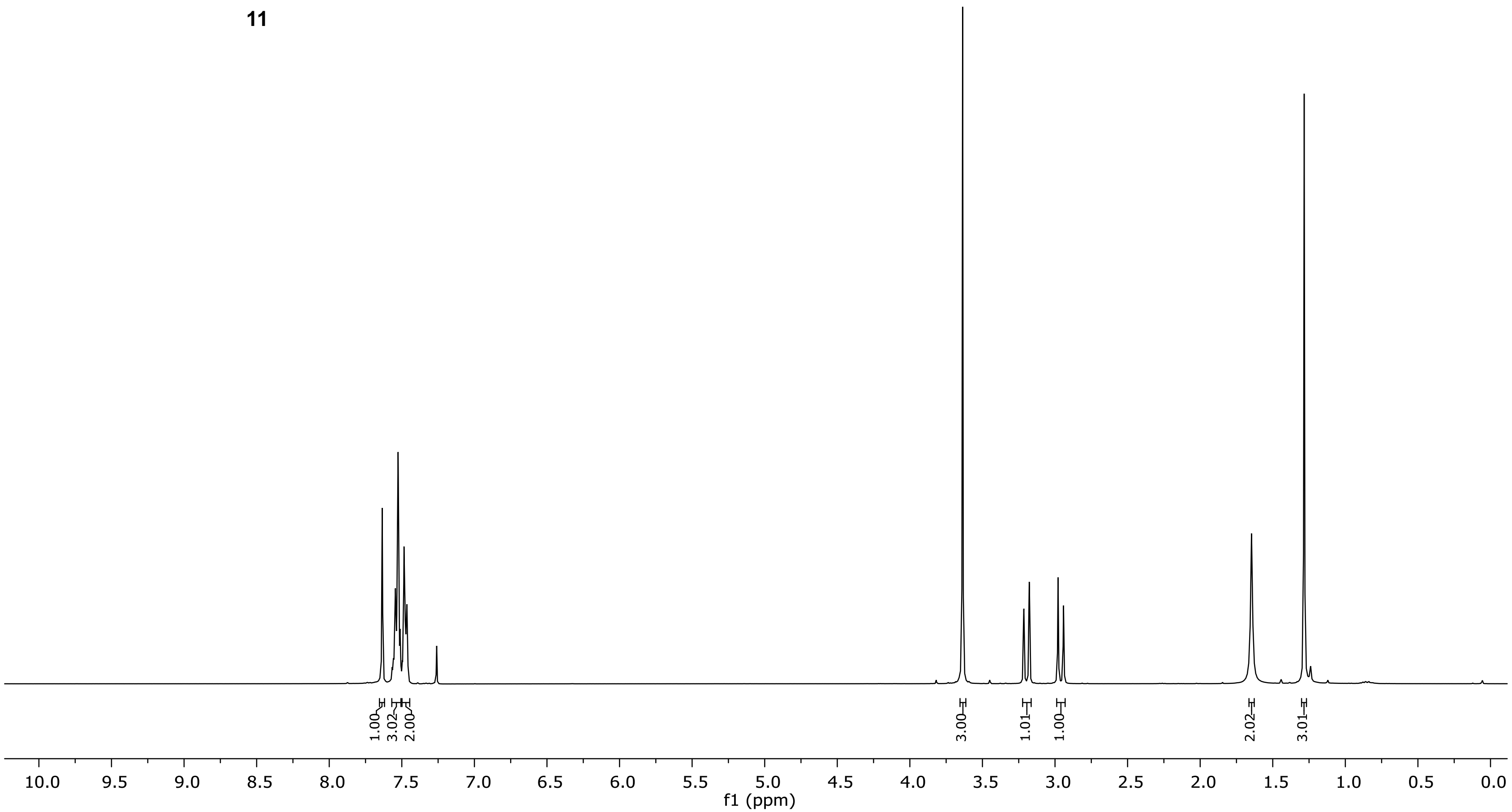
1.00
3.02
2.00
3.00
1.01
1.00
2.02
3.01
10.0
9.5
9.0
8.5
8.0
7.5
7.0
6.5
6.0
5.5
5.0
4.5
4.0
3.5
3.0
2.5
2.0
1.5
1.0
0.5
0.0
f1 (ppm)

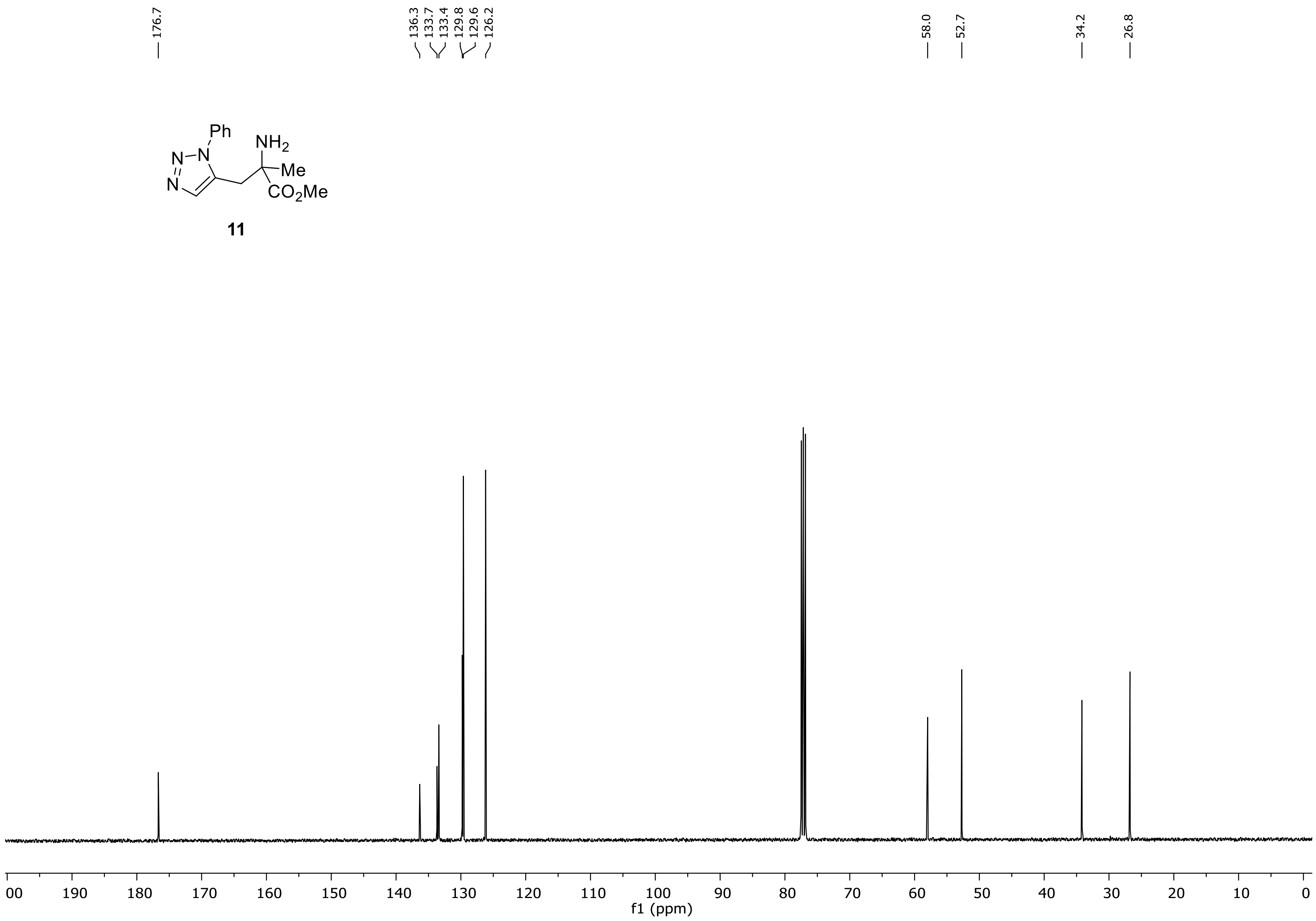

176.7
136.3
133.7
133.4
129.8
129.6
126.2
58.0
52.7
34.2
26.8
Ph
NH2
N
N
N
Me
CO2Me
11
00
190
180
170
160
150
140
130
120
110
100
90
80
70
60
50
40
30
20
10
0
f1 (ppm)

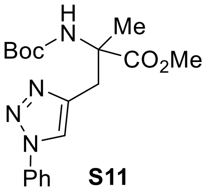
H
N
Me
Boc
CO2Me
N
N
N
Ph

S11

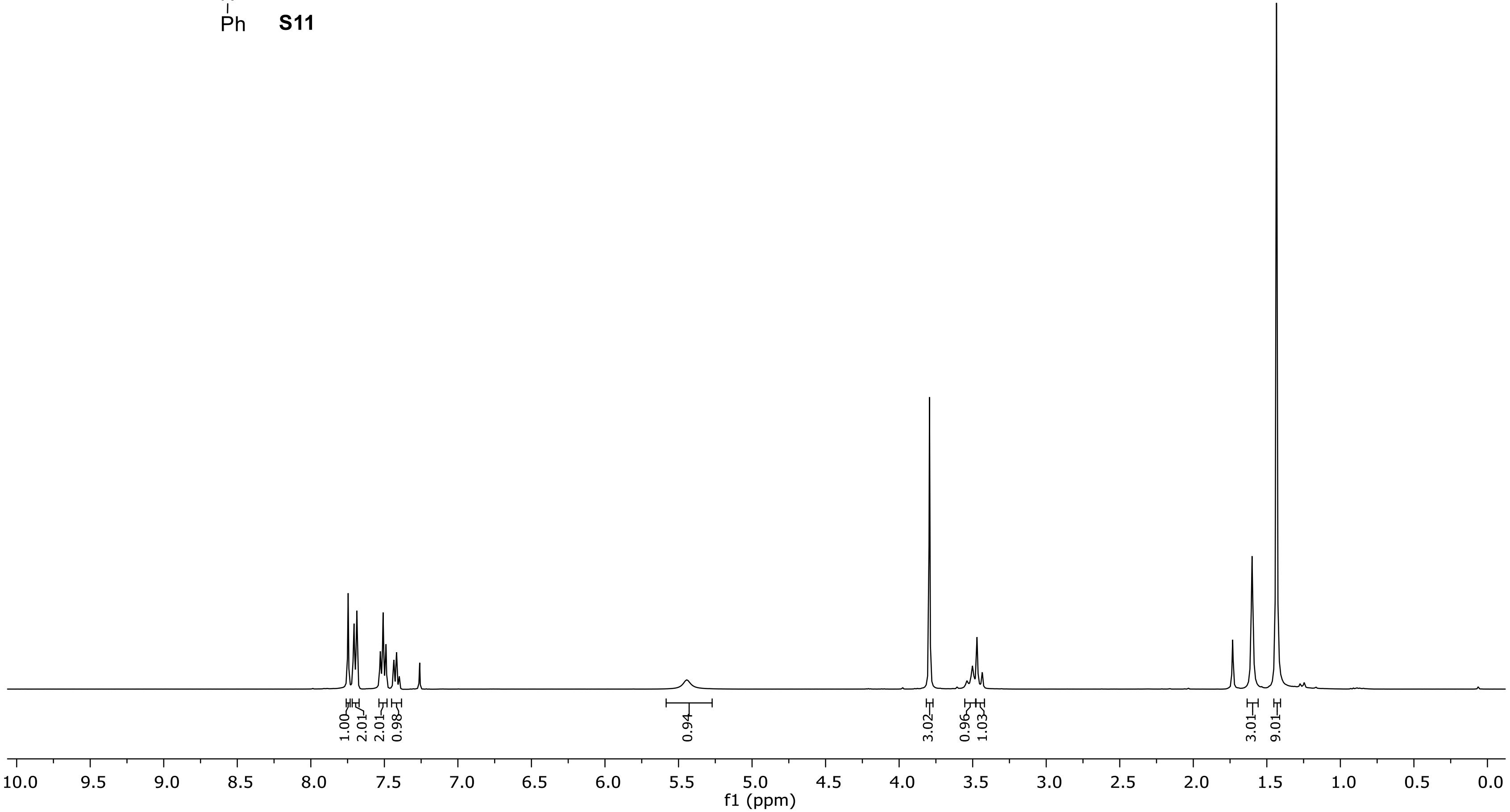
1.00
2.01
2.01
0.98
0.94
3.02
0.96
1.03
3.01
9.01
10.0
9.5
9.0
8.5
8.0
7.5
7.0
6.5
6.0
5.5
5.0
4.5
4.0
3.5
3.0
2.5
2.0
1.5
1.0
0.5
0.0
f1 (ppm)

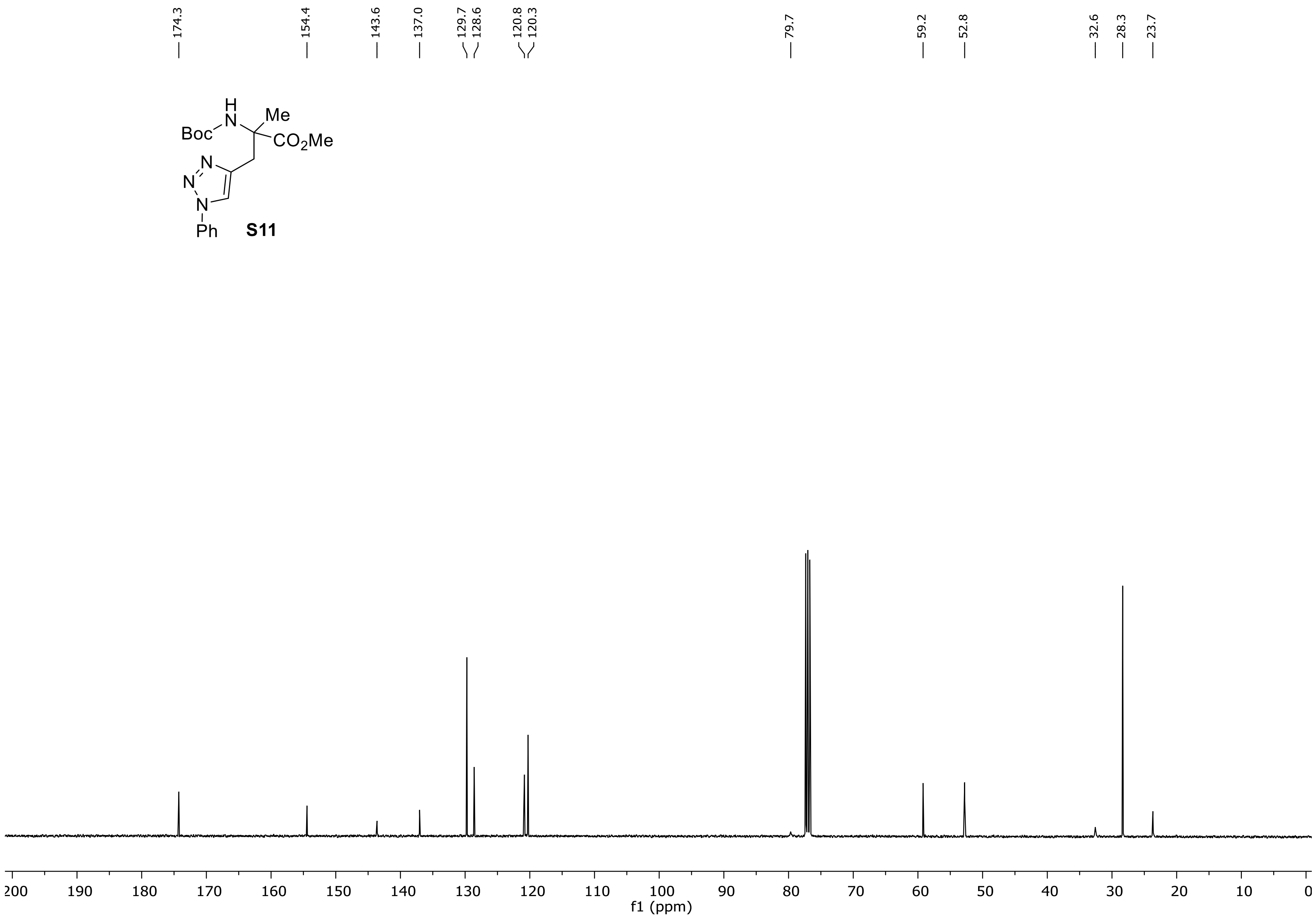
174.3
154.4
143.6
137.0
129.7
128.6
120.8
120.3
79.7
59.2
52.8
32.6
28.3
23.7
Boc
H
N
Me
CO_2Me
N
N
N
Ph
S11
200 190 180 170 160 150 140 130 120 110 100 90 80 70 60 50 40 30 20 10 0
f1 (ppm)

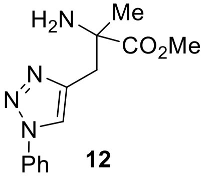
H2N
Me
CO2Me
N
N
N
Ph

12

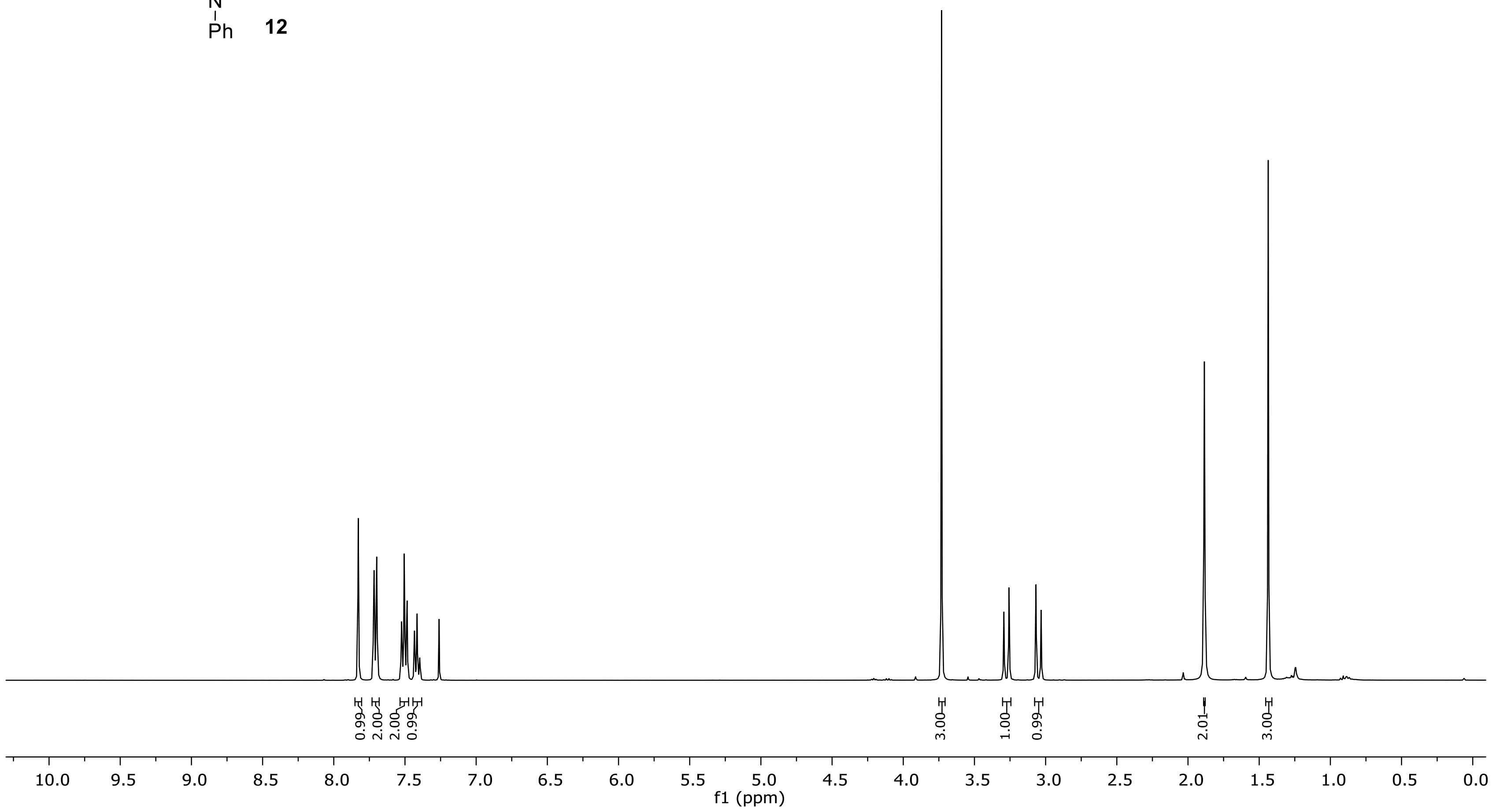
0.99
2.00
2.00
0.99
3.00
1.00
0.99
2.01
3.00
10.0 9.5 9.0 8.5 8.0 7.5 7.0 6.5 6.0 5.5 5.0 4.5 4.0 3.5 3.0 2.5 2.0 1.5 1.0 0.5 0.0
f1 (ppm)

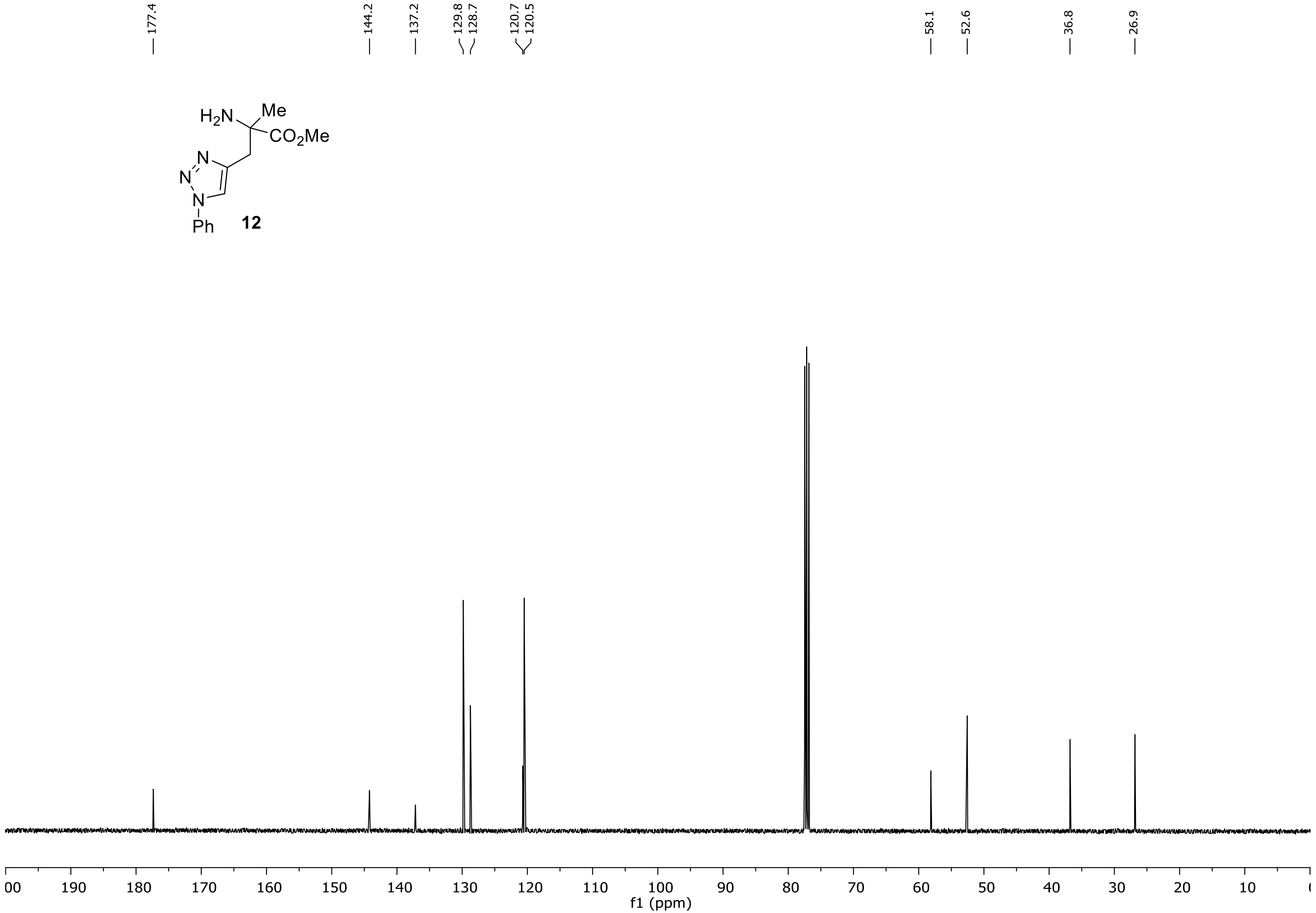

177.4
144.2
137.2
129.8
128.7
120.7
120.5
58.1
52.6
36.8
26.9
H_2N
Me
CO_2Me
N
N
N
Ph
12
00
190
180
170
160
150
140
130
120
110
100
90
80
70
60
50
40
30
20
10
f1 (ppm)

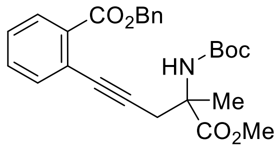
CO2Bn
HN
Boc
Me
CO2Me

S12

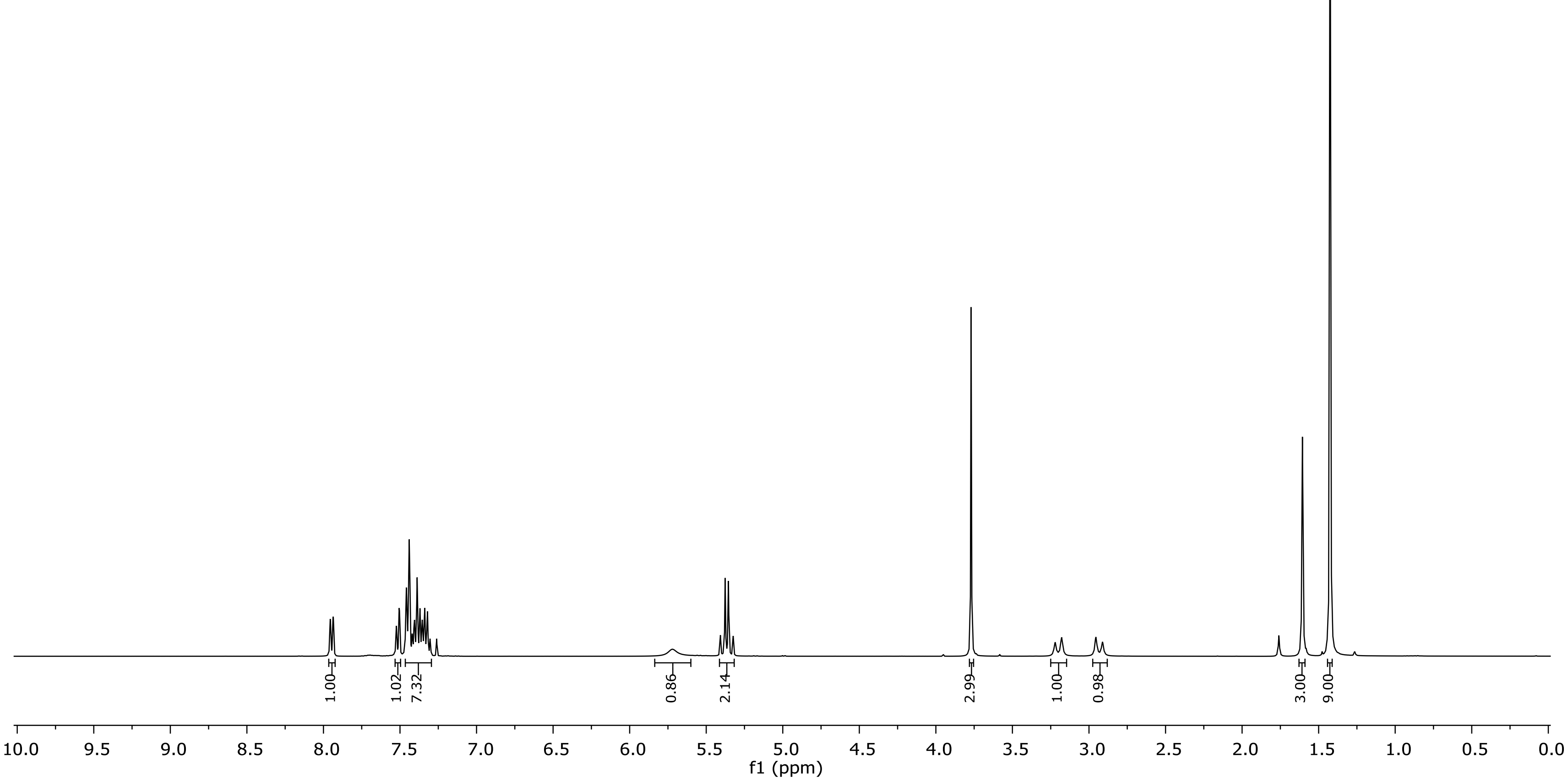
1.00
1.02
7.32
0.86
2.14
2.99
1.00
0.98
3.00
9.00
10.0 9.5 9.0 8.5 8.0 7.5 7.0 6.5 6.0 5.5 5.0 4.5 4.0 3.5 3.0 2.5 2.0 1.5 1.0 0.5 0.0
f1 (ppm)

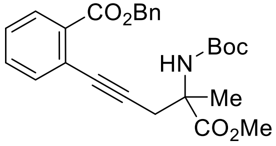
CO2Bn
HN
Boc
Me
CO2Me

S12

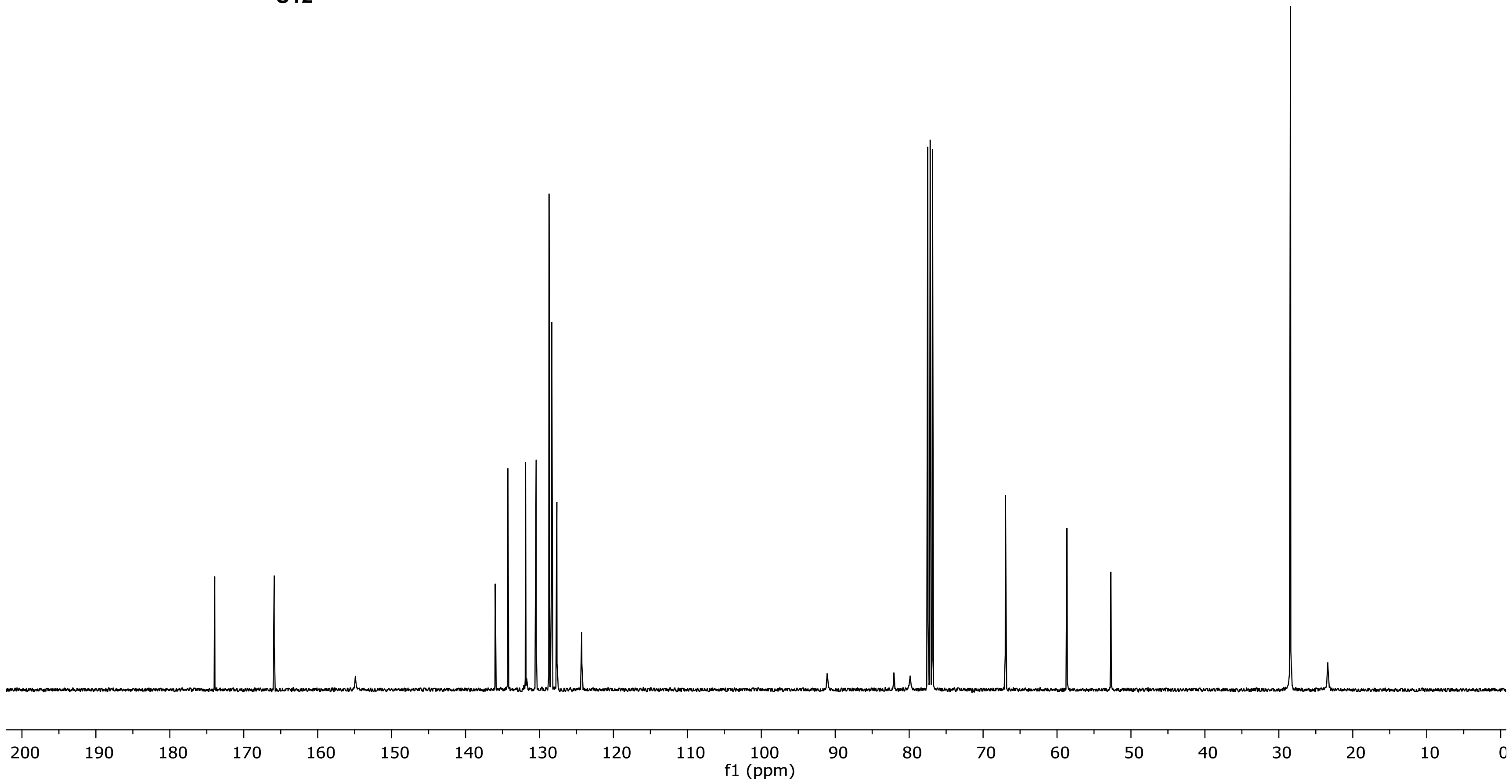
173.9
165.9
154.9
136.0
134.3
131.9
131.8
130.4
128.7
128.4
128.3
127.7
124.3
91.1
82.1
79.9
67.0
58.7
52.7
28.4
23.4
200 190 180 170 160 150 140 130 120 110 100 90 80 70 60 50 40 30 20 10 0
f1 (ppm)

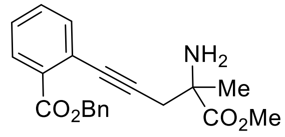
NH2
Me
CO2Bn
CO2Me

S13

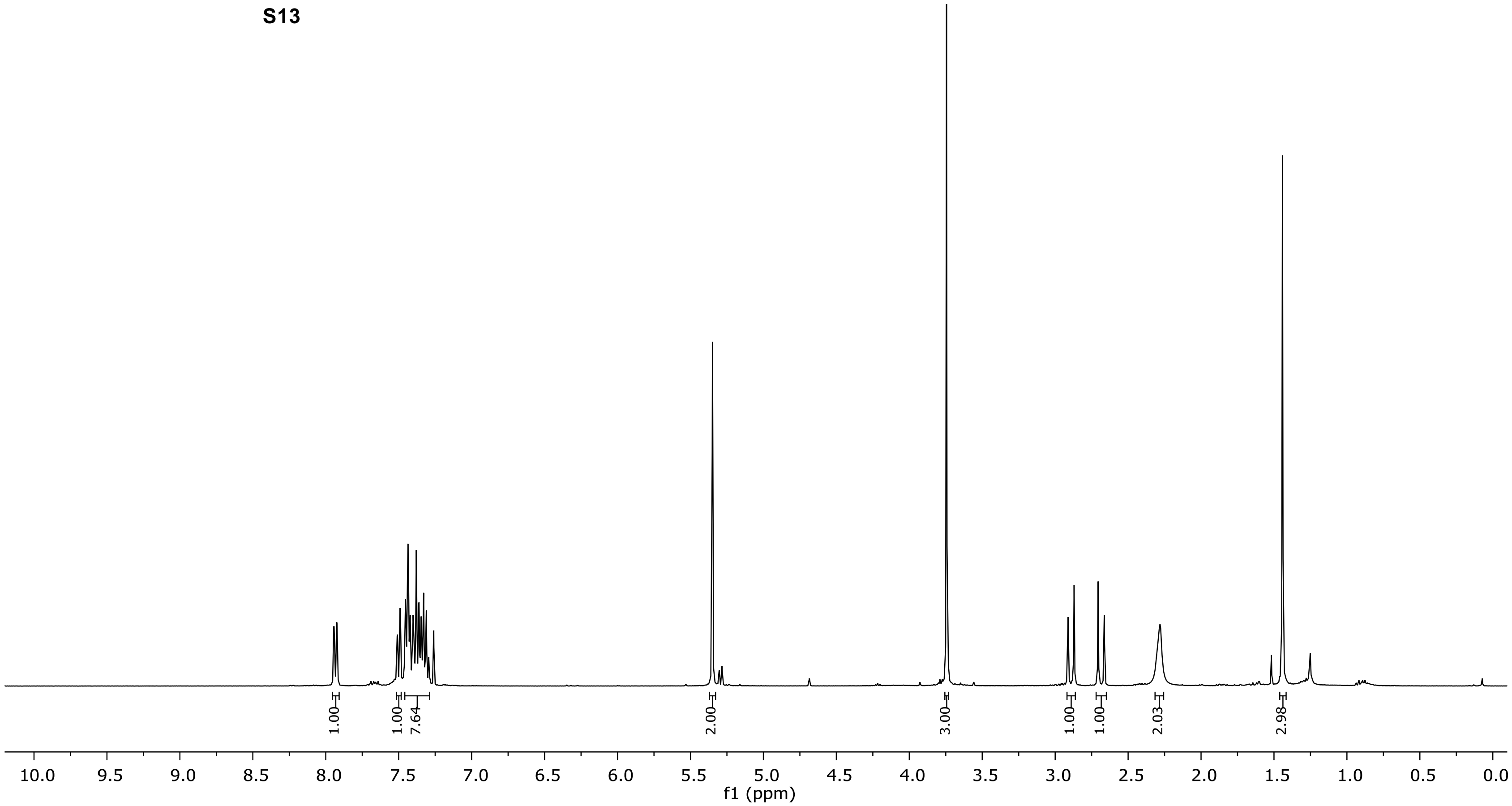
1.00
1.00
7.64
2.00
3.00
1.00
1.00
2.03
2.98
10.0 9.5 9.0 8.5 8.0 7.5 7.0 6.5 6.0 5.5 5.0 4.5 4.0 3.5 3.0 2.5 2.0 1.5 1.0 0.5 0.0
f1 (ppm)

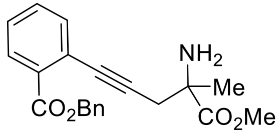
NH2
Me
CO2Bn
CO2Me

S13

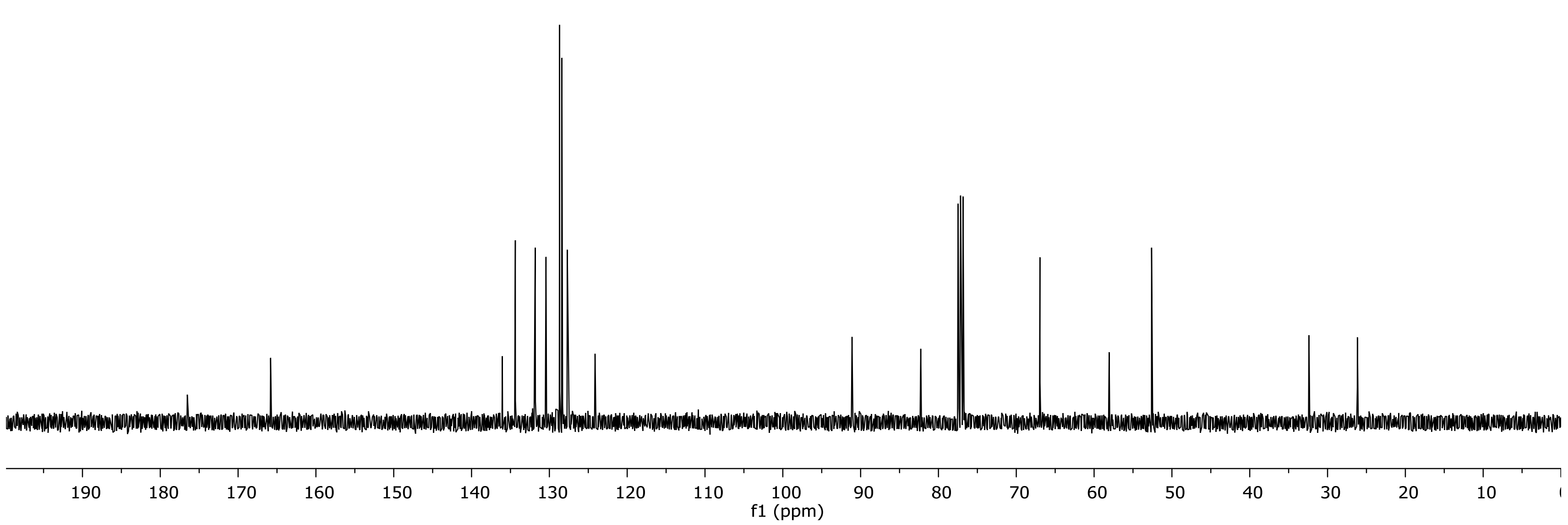
176.5
165.8
136.0
134.4
131.8
131.8
130.4
128.7
128.4
128.3
127.7
124.1
91.1
82.3
67.0
58.1
52.6
32.4
26.2
190 180 170 160 150 140 130 120 110 100 90 80 70 60 50 40 30 20 10
f1 (ppm)

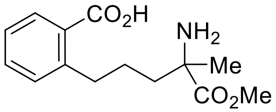

13

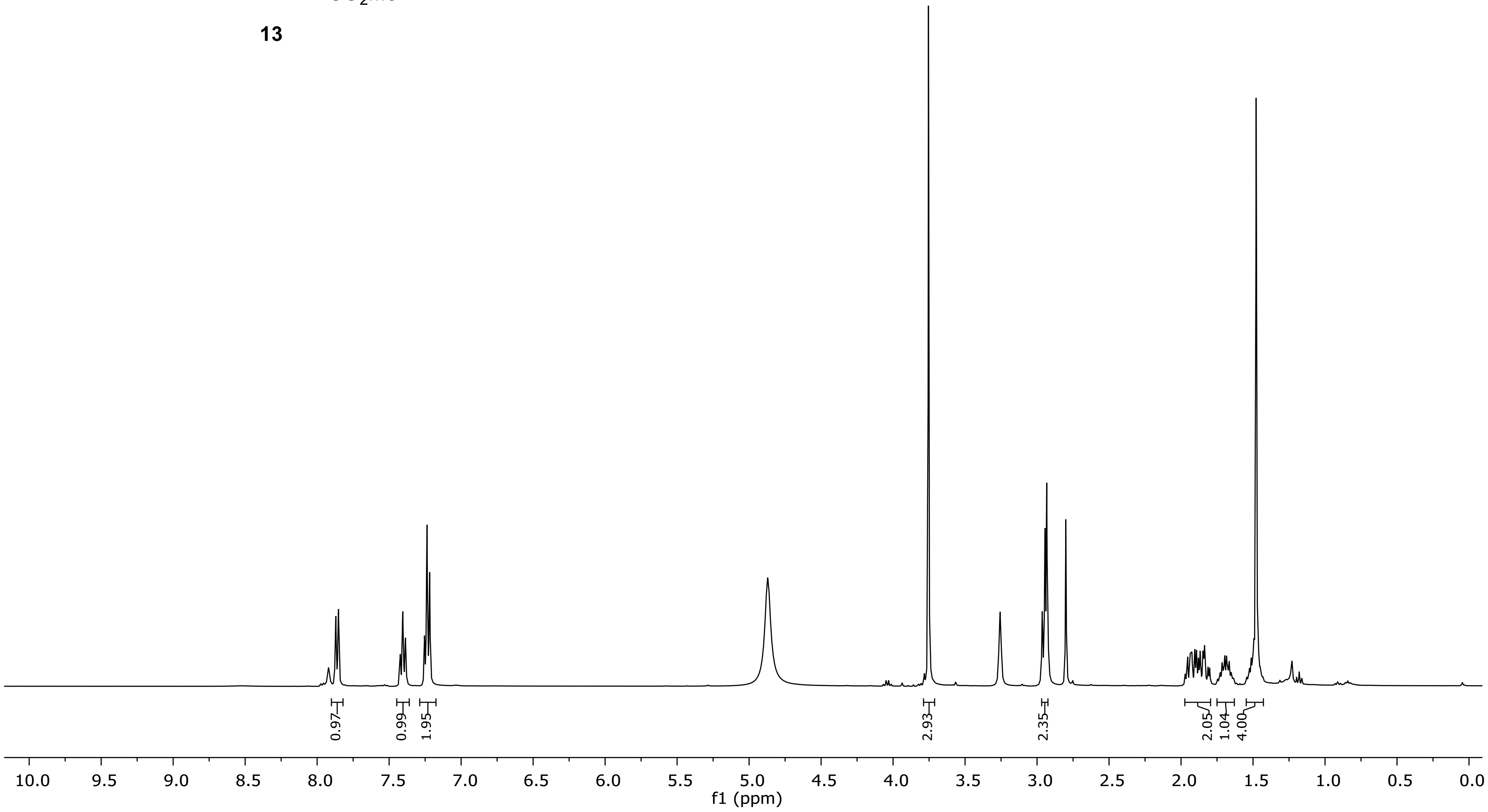

0.97
0.99
1.95
2.93
2.35
2.05
1.04
4.00
10.0
9.5
9.0
8.5
8.0
7.5
7.0
6.5
6.0
5.5
5.0
4.5
4.0
3.5
3.0
2.5
2.0
1.5
1.0
0.5
0.0
f1 (ppm)

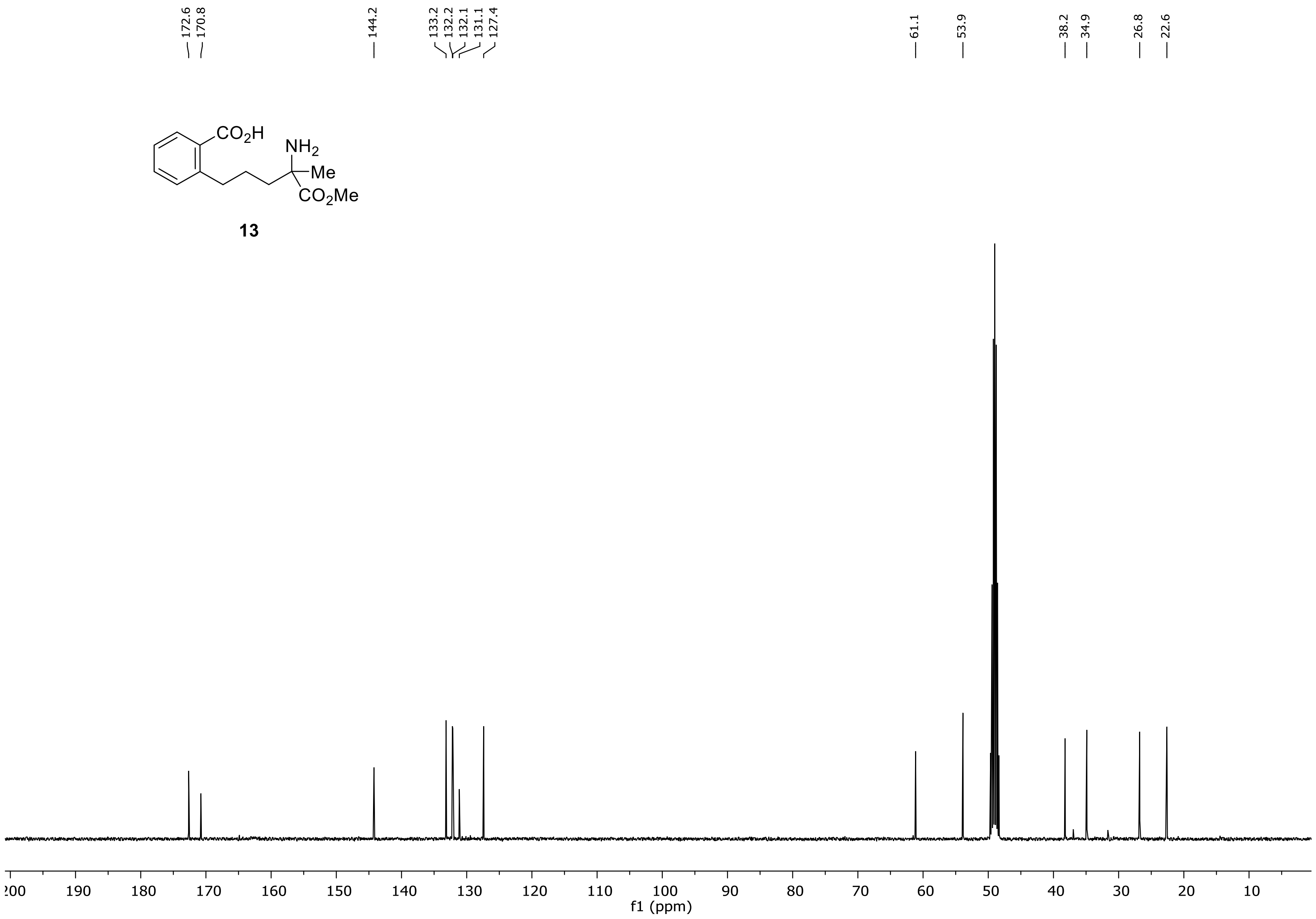
172.6
170.8
144.2
133.2
132.2
132.1
131.1
127.4
61.1
53.9
38.2
34.9
26.8
22.6
CO2H
NH2
Me
CO2Me
200 190 180 170 160 150 140 130 120 110 100 90 80 70 60 50 40 30 20 10
f1 (ppm)

13

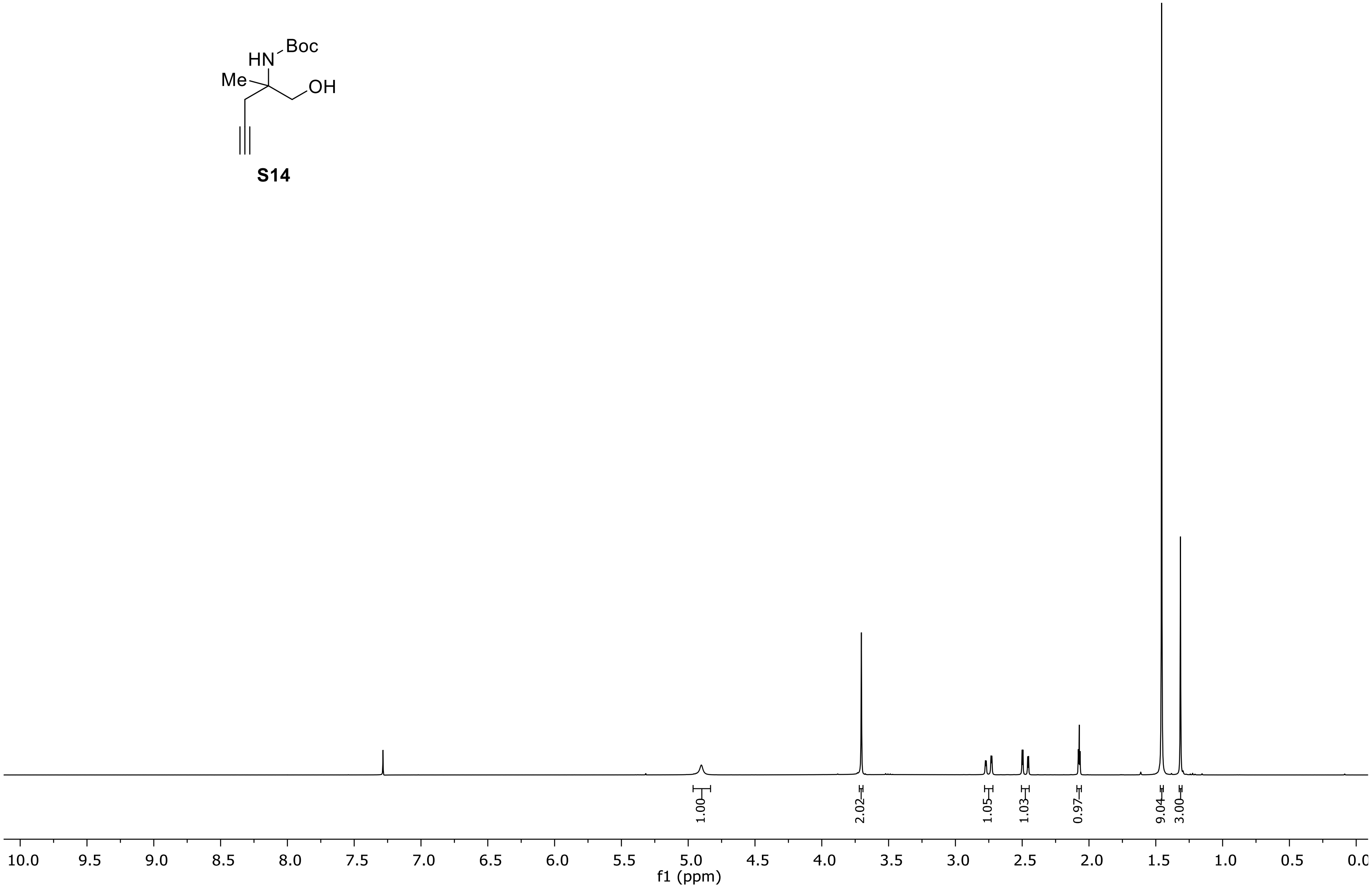
HN
Boc
Me
OH
S14
1.00
2.02
1.05
1.03
0.97
9.04
3.00
10.0
9.5
9.0
8.5
8.0
7.5
7.0
6.5
6.0
5.5
5.0
4.5
4.0
3.5
3.0
2.5
2.0
1.5
1.0
0.5
0.0
f1 (ppm)

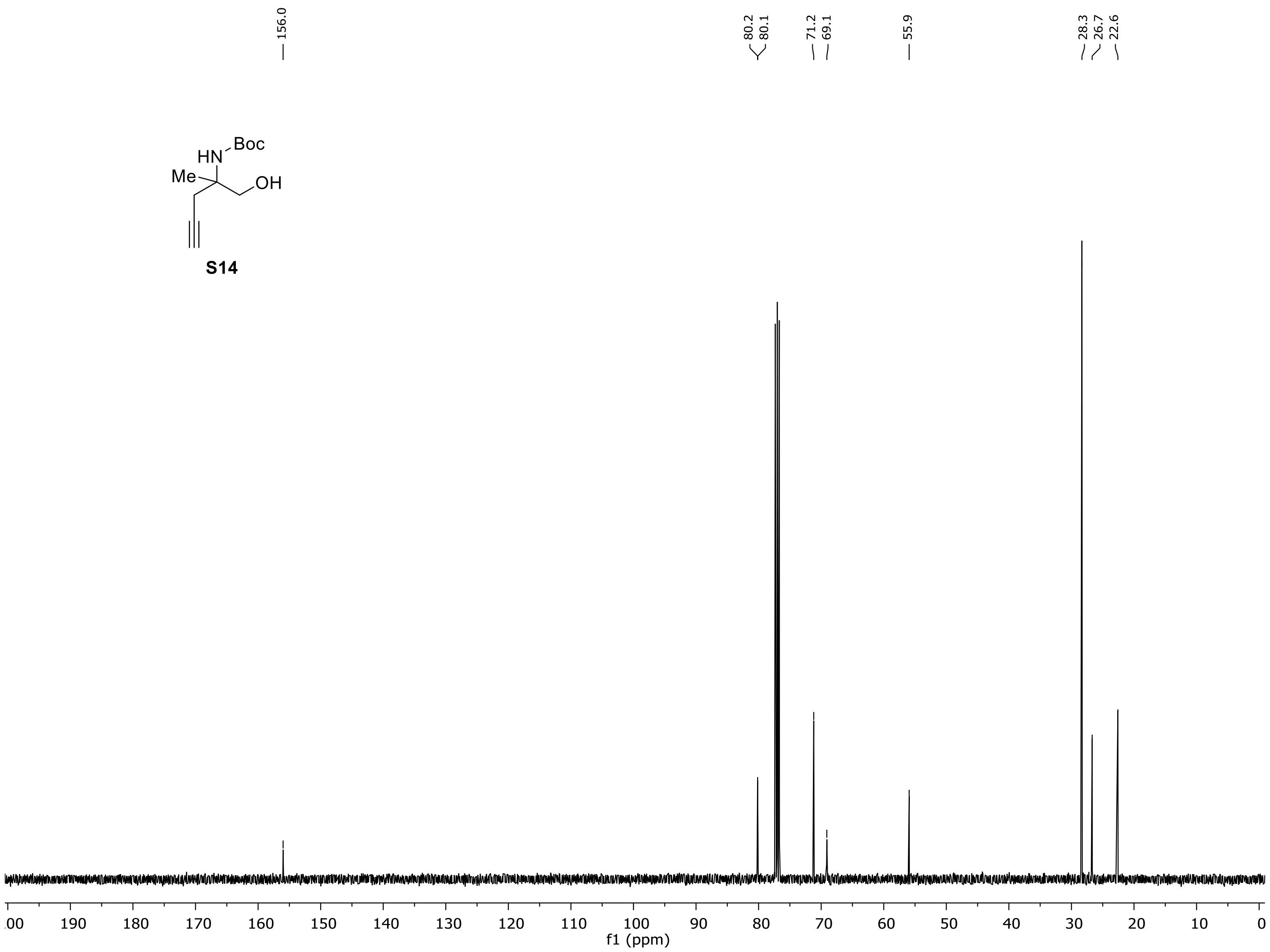

156.0
80.2
80.1
71.2
69.1
55.9
28.3
26.7
22.6
HN
Boc
Me
OH
S14
.00 190 180 170 160 150 140 130 120 110 100 90 80 70 60 50 40 30 20 10 0
f1 (ppm)

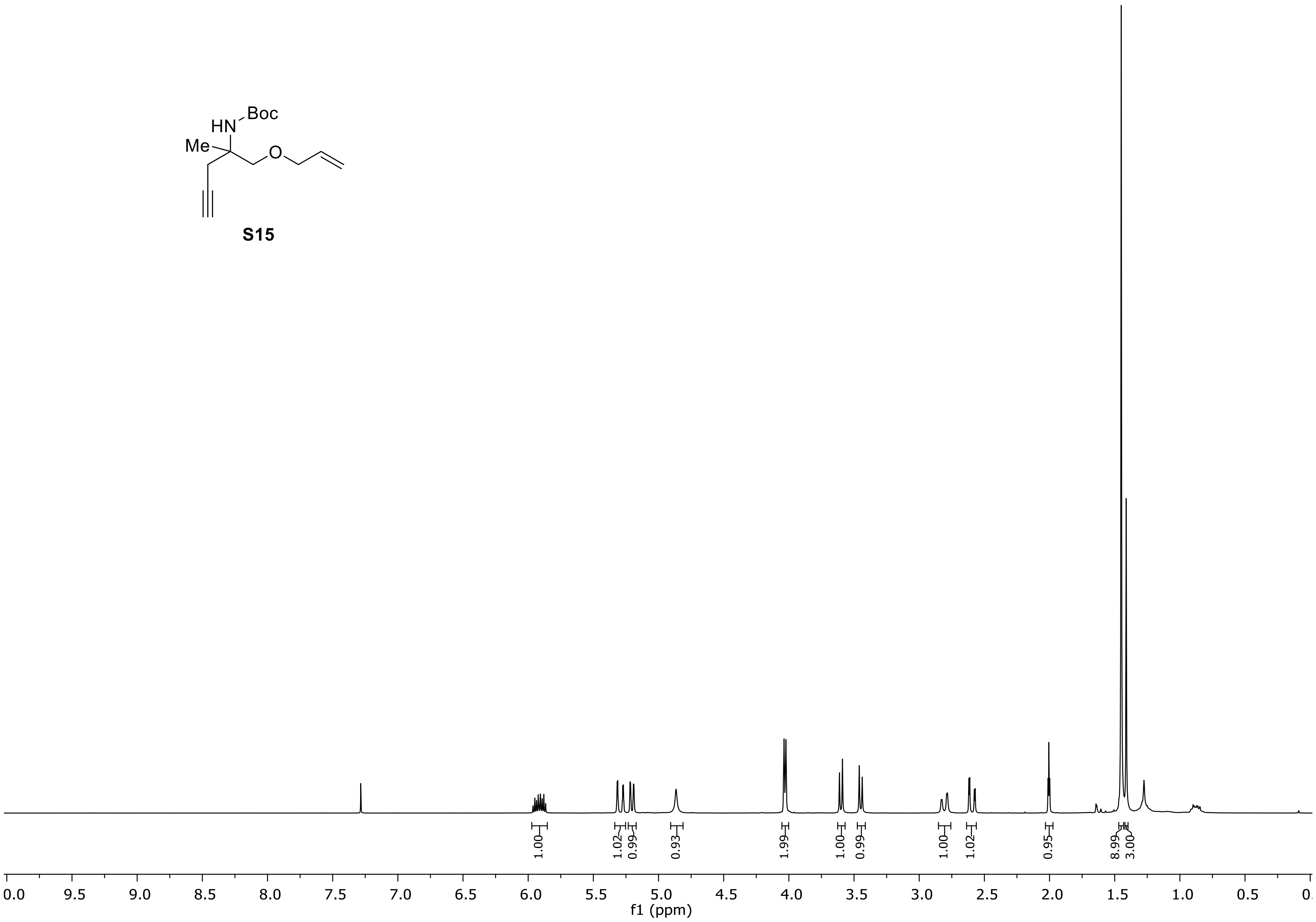
HN
Boc
Me
O
S15
1.00
1.02
0.99
0.93
1.99
1.00
0.99
1.00
1.02
0.95
8.99
3.00
9.5
9.0
8.5
8.0
7.5
7.0
6.5
6.0
5.5
5.0
4.5
4.0
3.5
3.0
2.5
2.0
1.5
1.0
0.5
f1 (ppm)

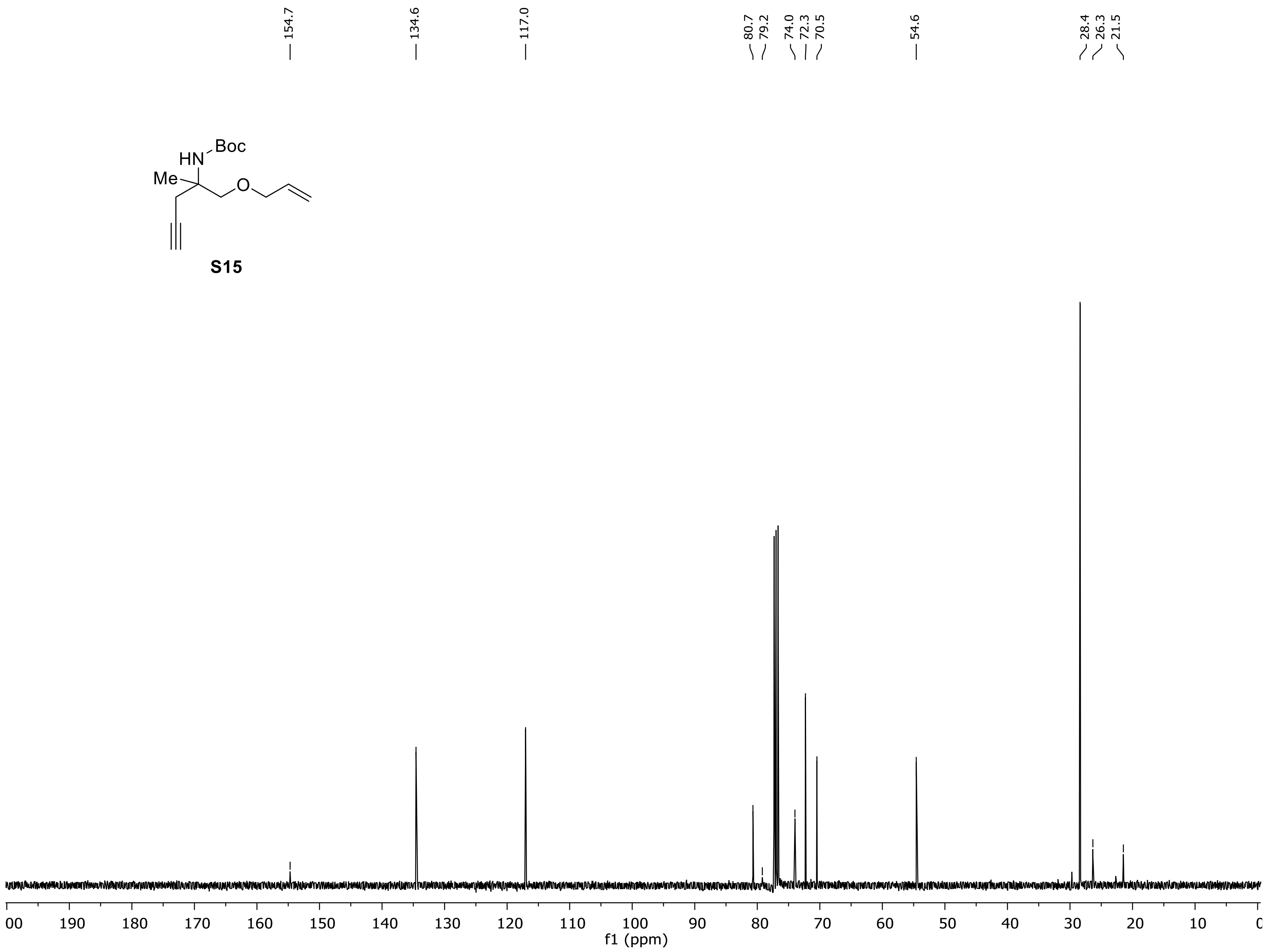
154.7
134.6
117.0
80.7
79.2
74.0
72.3
70.5
54.6
28.4
26.3
21.5
HN
Boc
Me
O
S15
00 190 180 170 160 150 140 130 120 110 100 90 80 70 60 50 40 30 20 10 0
f1 (ppm)

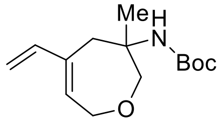
Me
H
N
Boc
O

S16

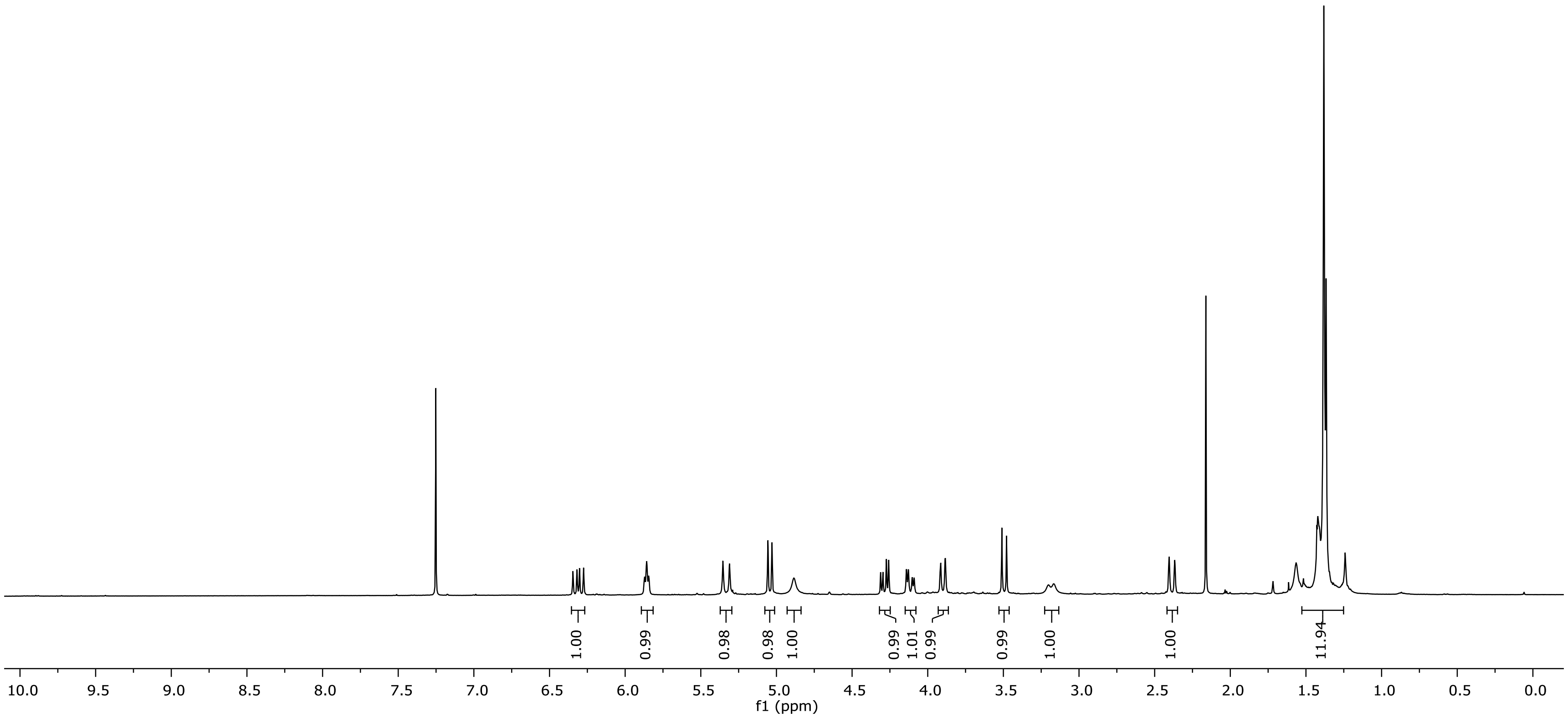
1.00
0.99
0.98
0.98
1.00
0.99
1.01
0.99
0.99
1.00
1.00
11.94
10.0
9.5
9.0
8.5
8.0
7.5
7.0
6.5
6.0
5.5
5.0
4.5
4.0
3.5
3.0
2.5
2.0
1.5
1.0
0.5
0.0
f1 (ppm)

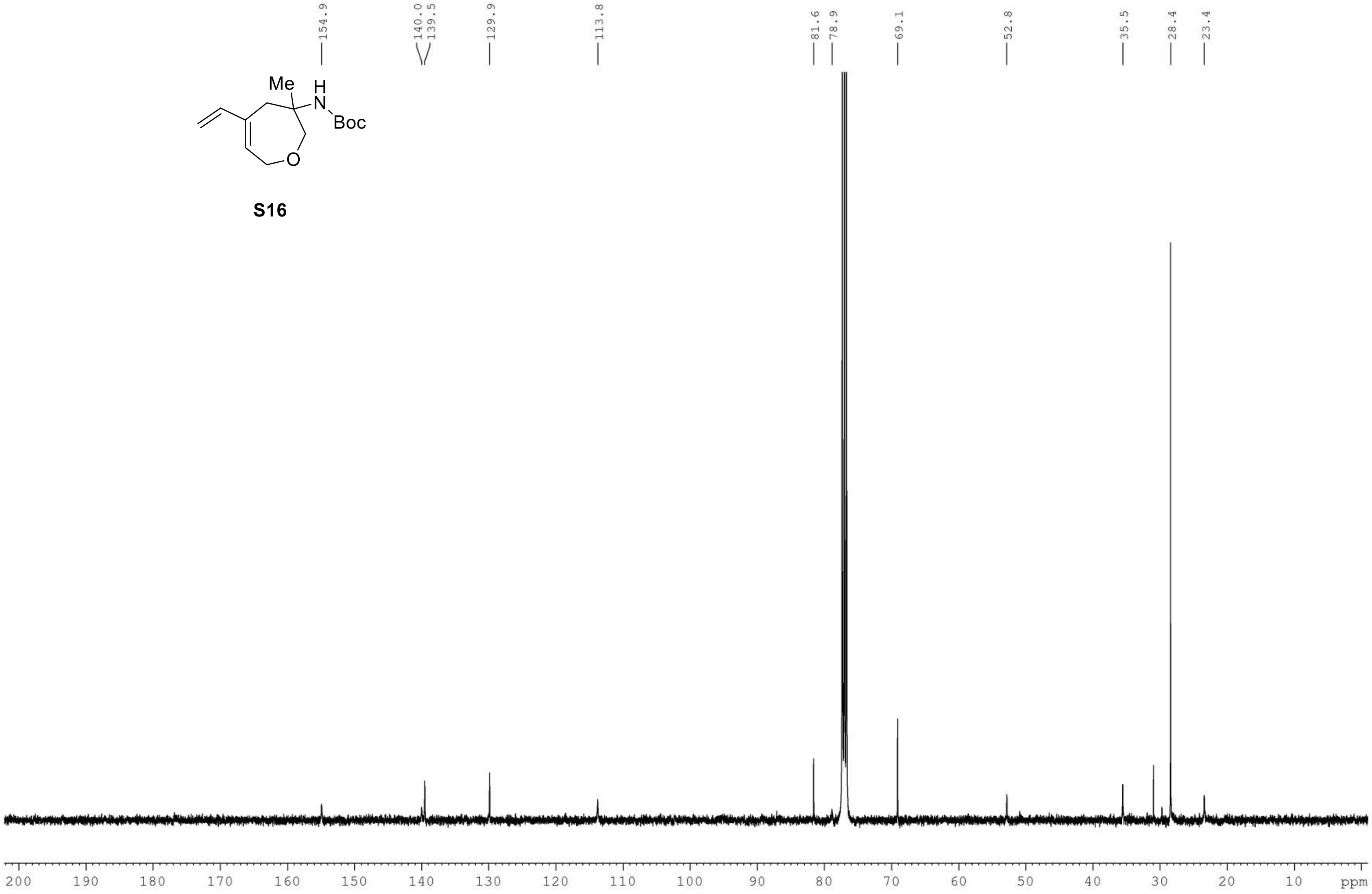

154.9
140.0
139.5
129.9
113.8
81.6
78.9
69.1
52.8
35.5
28.4
23.4
200
190
180
170
160
150
140
130
120
110
100
90
80
70
60
50
40
30
20
10
ppm

S16

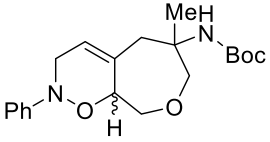
MeH
N
Boc
Ph
N
O
H
O

14

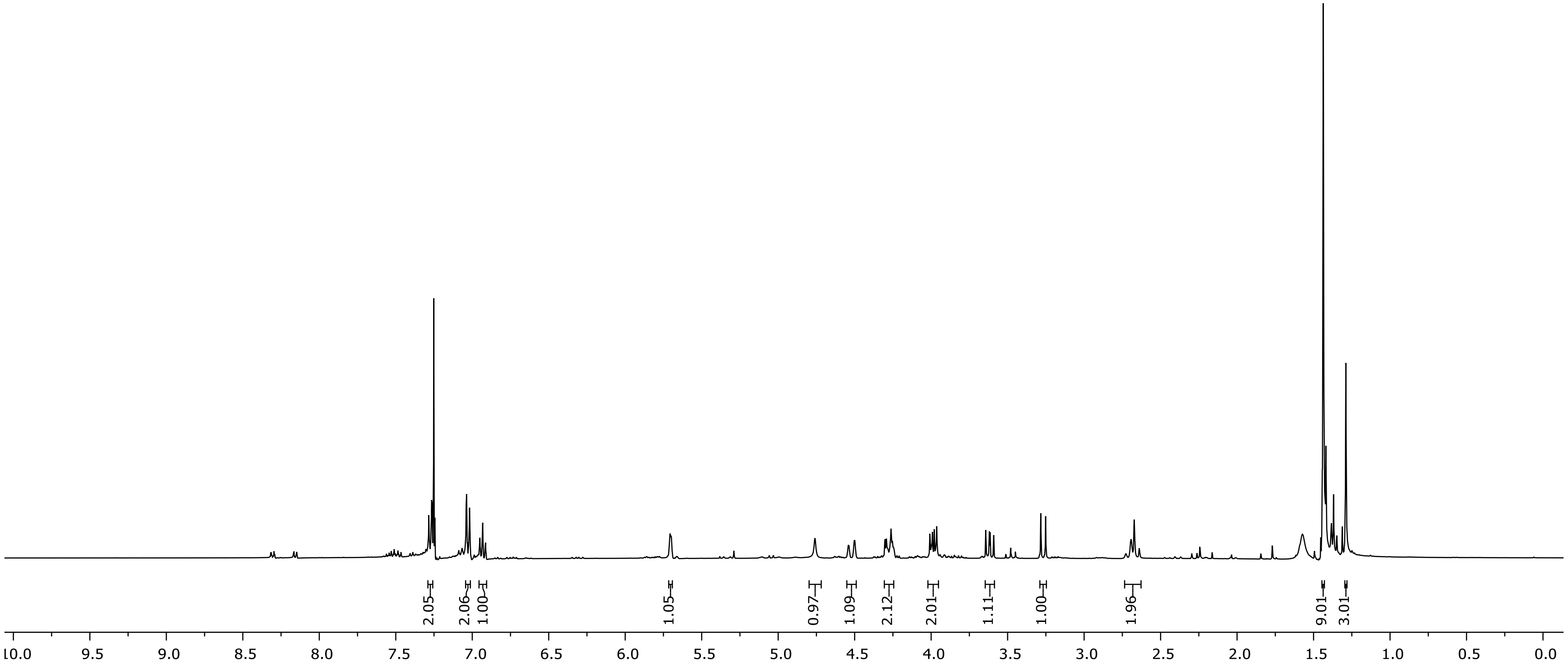
2.05
2.06
1.00
1.05
0.97
1.09
2.12
2.01
1.11
1.00
1.96
9.01
3.01
10.0 9.5 9.0 8.5 8.0 7.5 7.0 6.5 6.0 5.5 5.0 4.5 4.0 3.5 3.0 2.5 2.0 1.5 1.0 0.5 0.0
f1 (ppm)

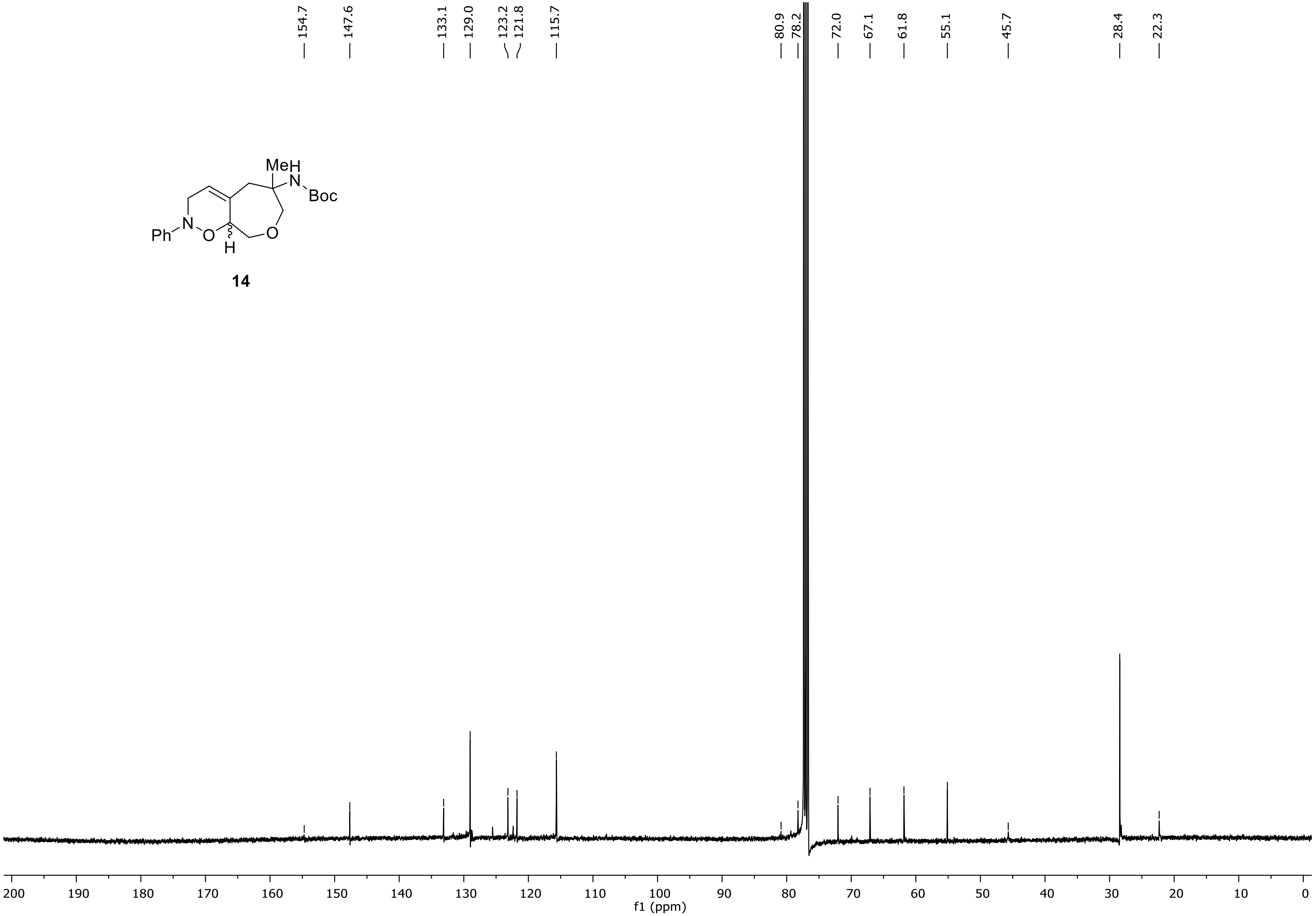
154.7
147.6
133.1
129.0
123.2
121.8
115.7
80.9
78.2
72.0
67.1
61.8
55.1
45.7
28.4
22.3
Me
H
N
Boc
N
Ph
O
H
O
14
200 190 180 170 160 150 140 130 120 110 100 90 80 70 60 50 40 30 20 10 0
f1 (ppm)

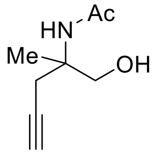
HN
Ac
Me
OH

S17

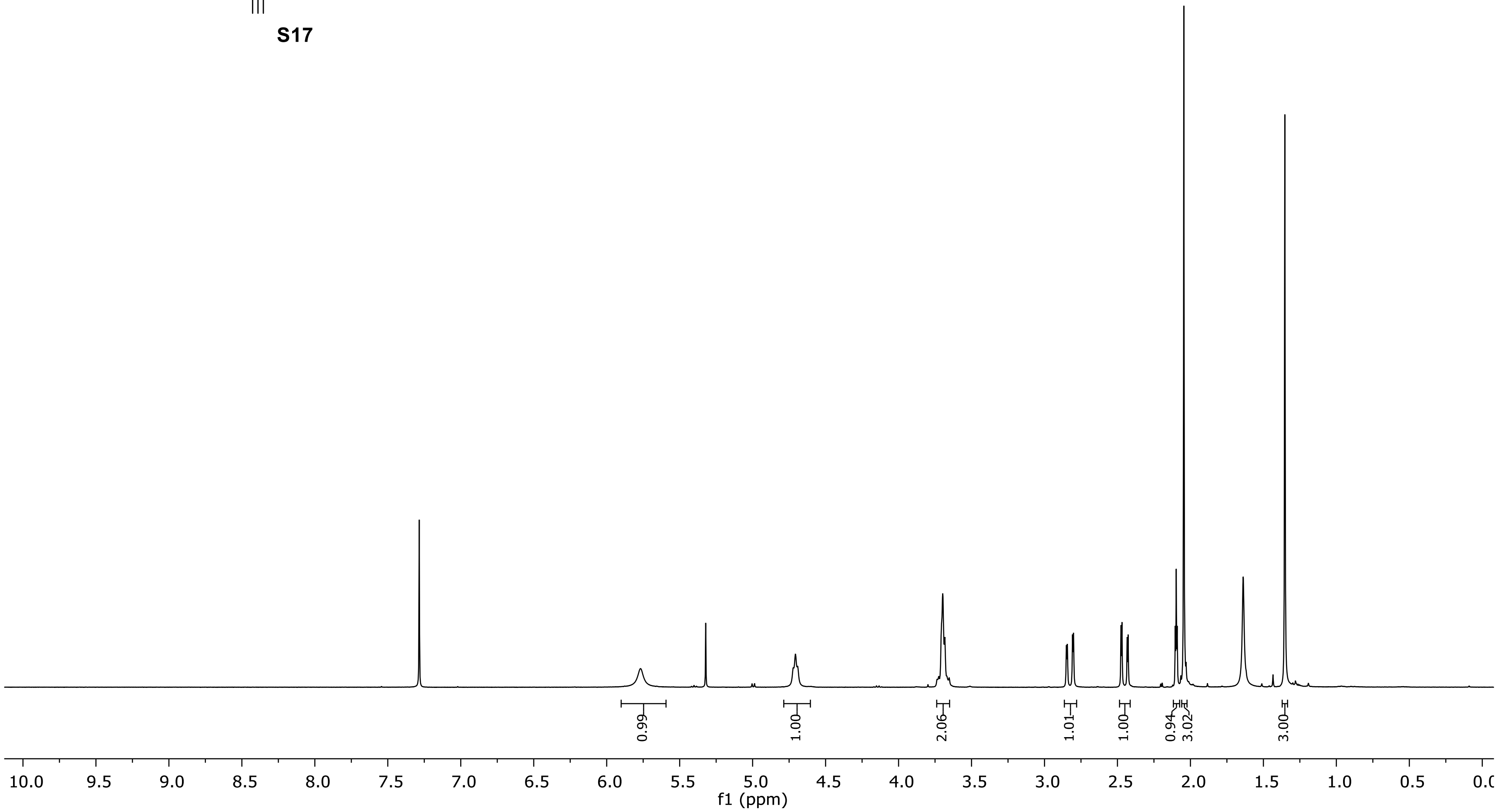
0.99
1.00
2.06
1.01
1.00
0.94
3.02
3.00
10.0 9.5 9.0 8.5 8.0 7.5 7.0 6.5 6.0 5.5 5.0 4.5 4.0 3.5 3.0 2.5 2.0 1.5 1.0 0.5 0.0
f1 (ppm)

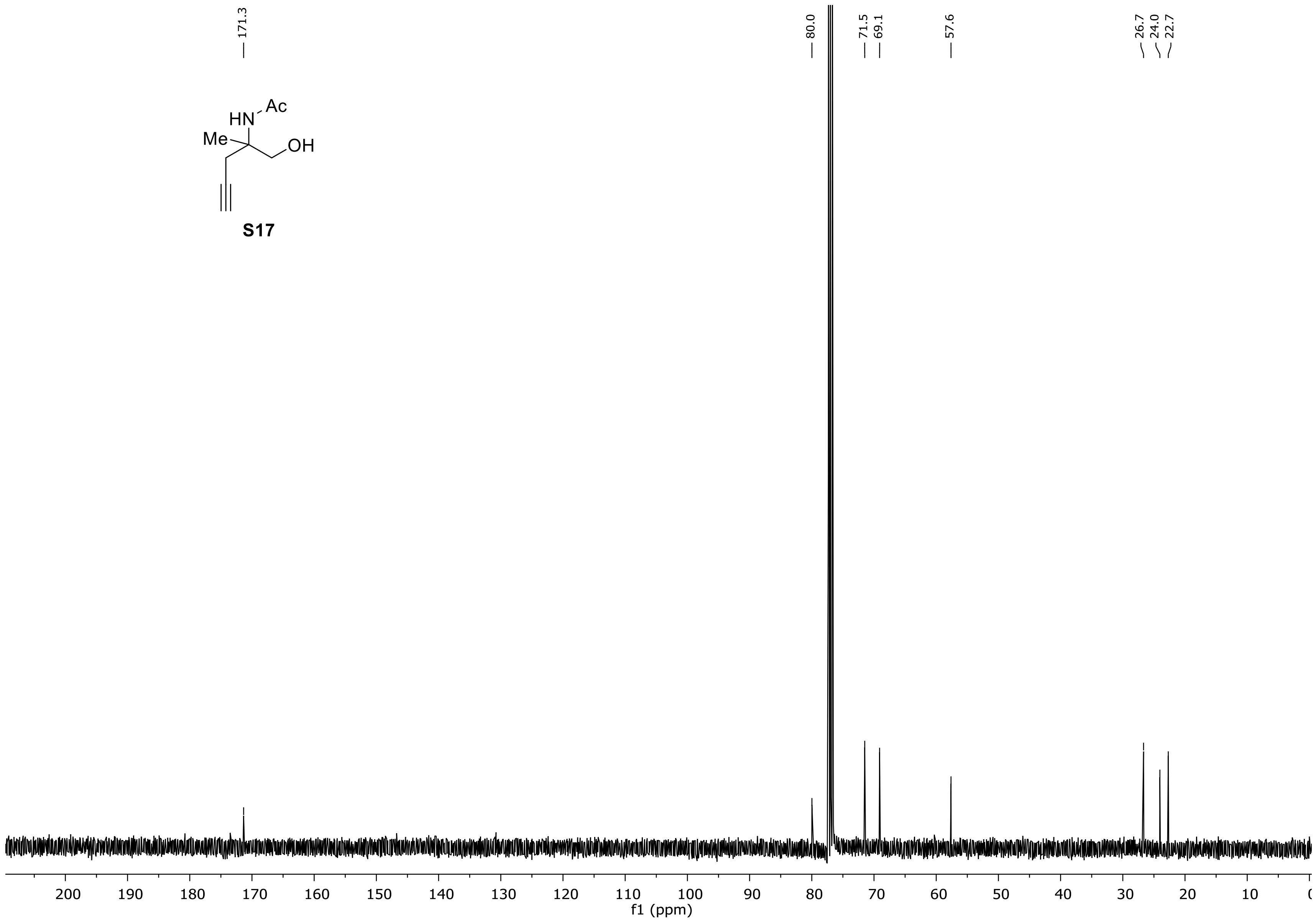
171.3
80.0
71.5
69.1
57.6
26.7
24.0
22.7
HN
Ac
Me
OH
S17
200
190
180
170
160
150
140
130
120
110
100
90
80
70
60
50
40
30
20
10
f1 (ppm)

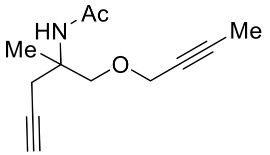
HN
Ac
Me
O
Me

S18

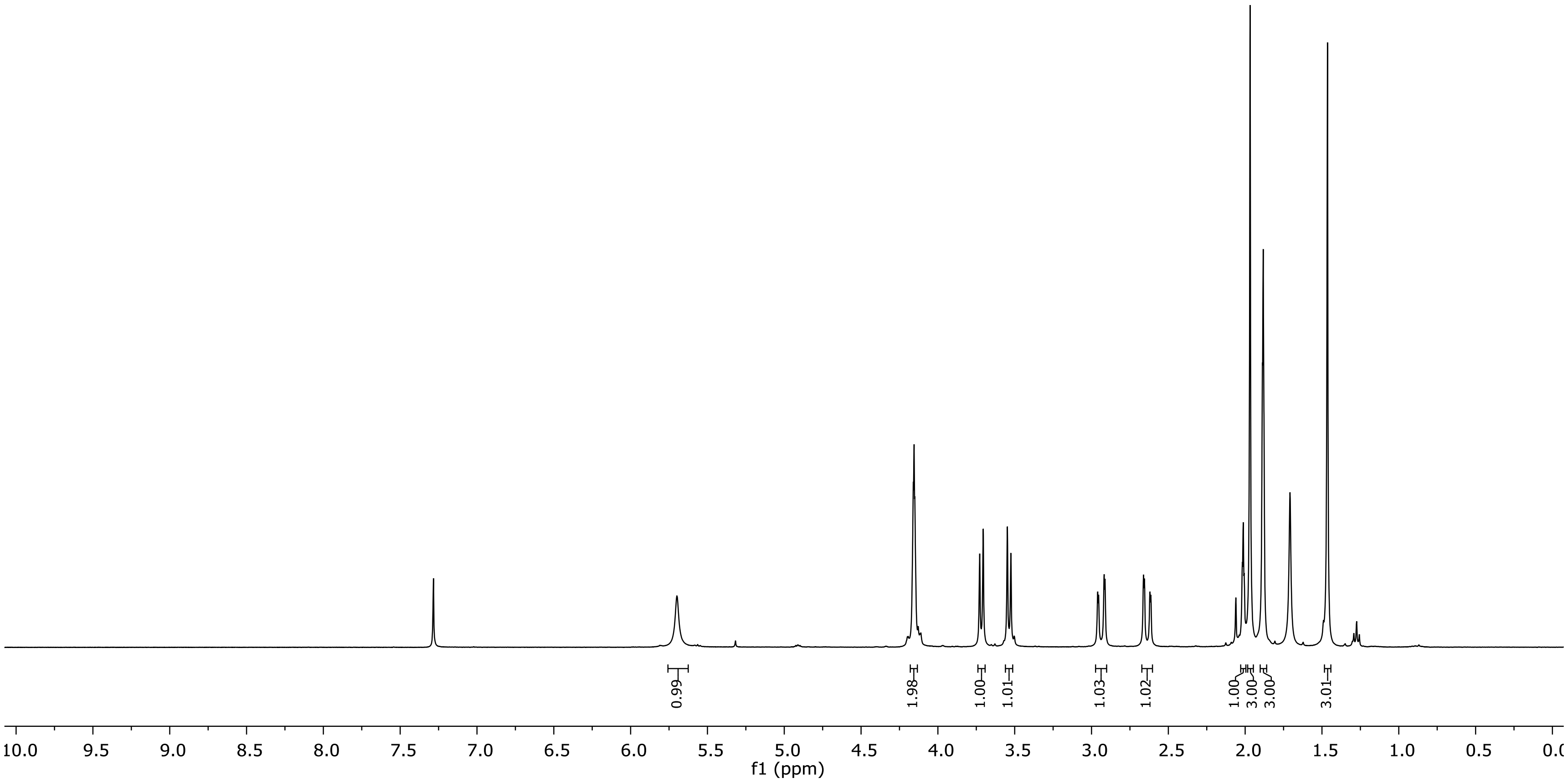
0.99
1.98
1.00
1.01
1.03
1.02
1.00
3.00
3.00
3.01
10.0
9.5
9.0
8.5
8.0
7.5
7.0
6.5
6.0
5.5
5.0
4.5
4.0
3.5
3.0
2.5
2.0
1.5
1.0
0.5
f1 (ppm)

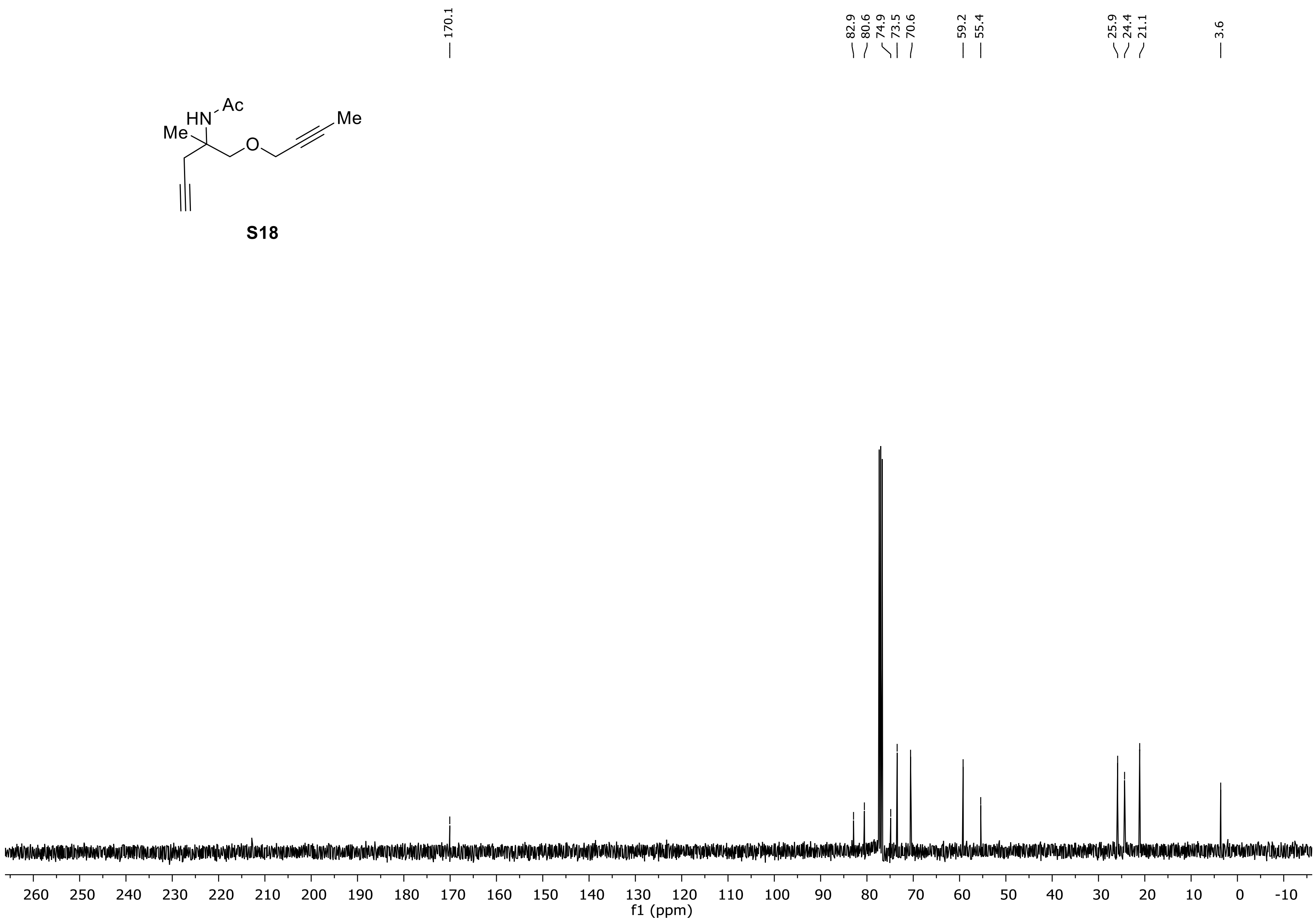
170.1
82.9
80.6
74.9
73.5
70.6
59.2
55.4
25.9
24.4
21.1
3.6
HN
Ac
Me
O
Me
S18
260 250 240 230 220 210 200 190 180 170 160 150 140 130 120 110 100 90 80 70 60 50 40 30 20 10 0 -10
f1 (ppm)

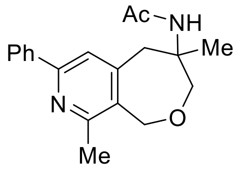
Ac
NH
Me
Ph
N
O
Me

15

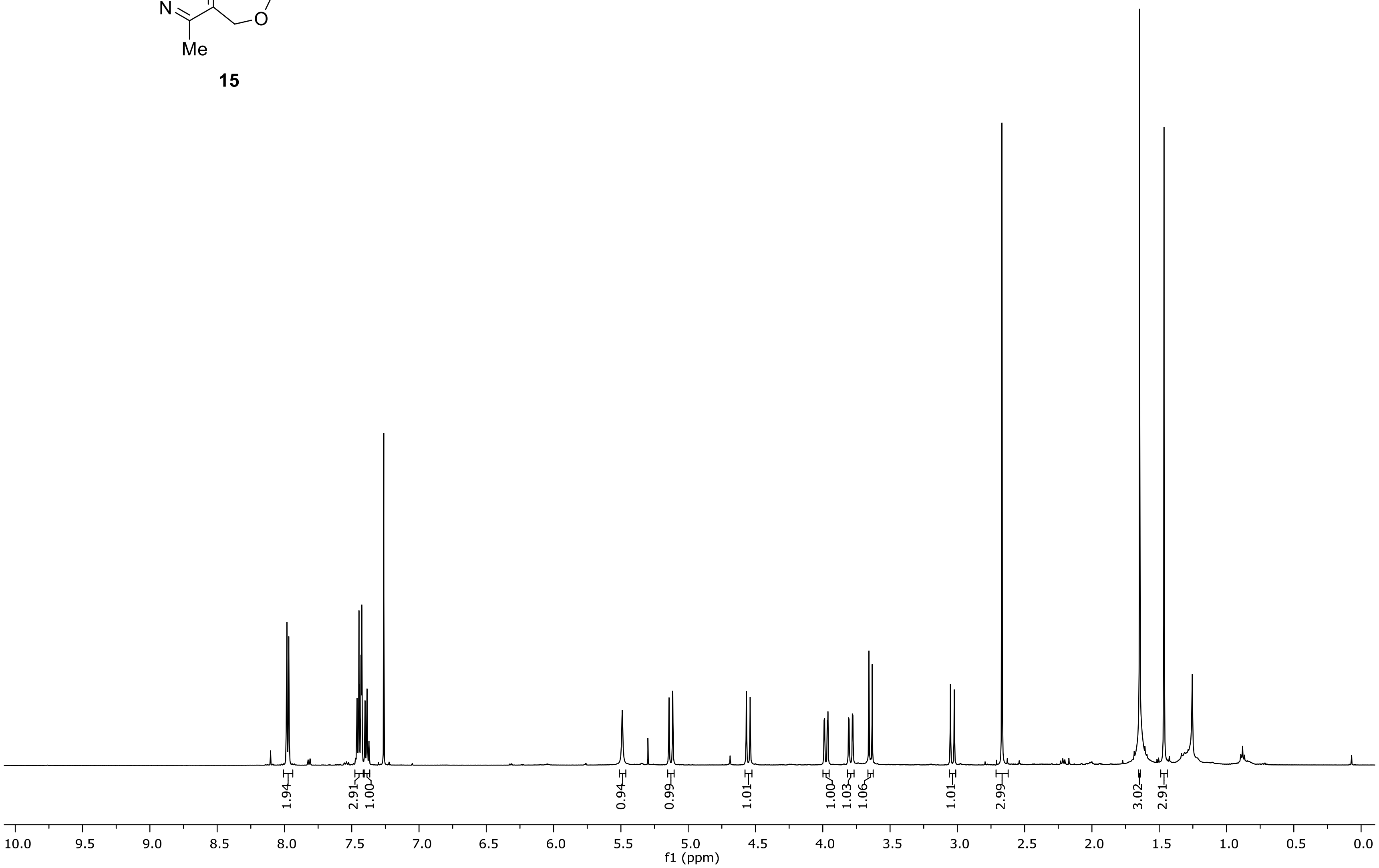
1.94
2.91
1.00
0.94
0.99
1.01
1.00
1.03
1.06
1.01
2.99
3.02
2.91
10.0 9.5 9.0 8.5 8.0 7.5 7.0 6.5 6.0 5.5 5.0 4.5 4.0 3.5 3.0 2.5 2.0 1.5 1.0 0.5 0.0
f1 (ppm)

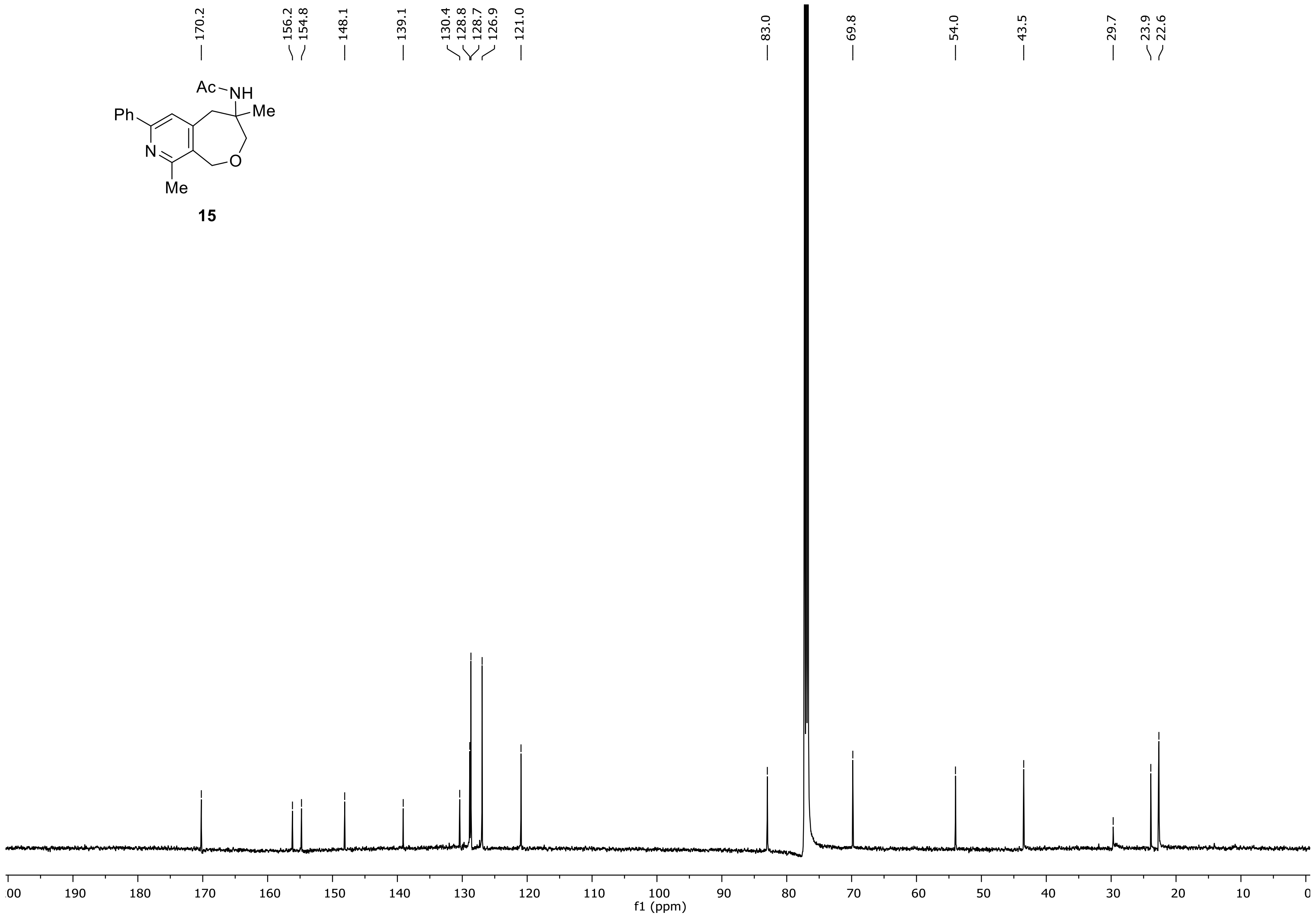
170.2
156.2
154.8
148.1
139.1
130.4
128.8
128.7
126.9
121.0
83.0
69.8
54.0
43.5
29.7
23.9
22.6
Ac
NH
Me
Ph
N
O
Me
15
00
190
180
170
160
150
140
130
120
110
100
90
80
70
60
50
40
30
20
10
0
f1 (ppm)

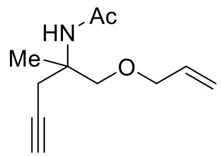

HN
Ac
Me
O

S19

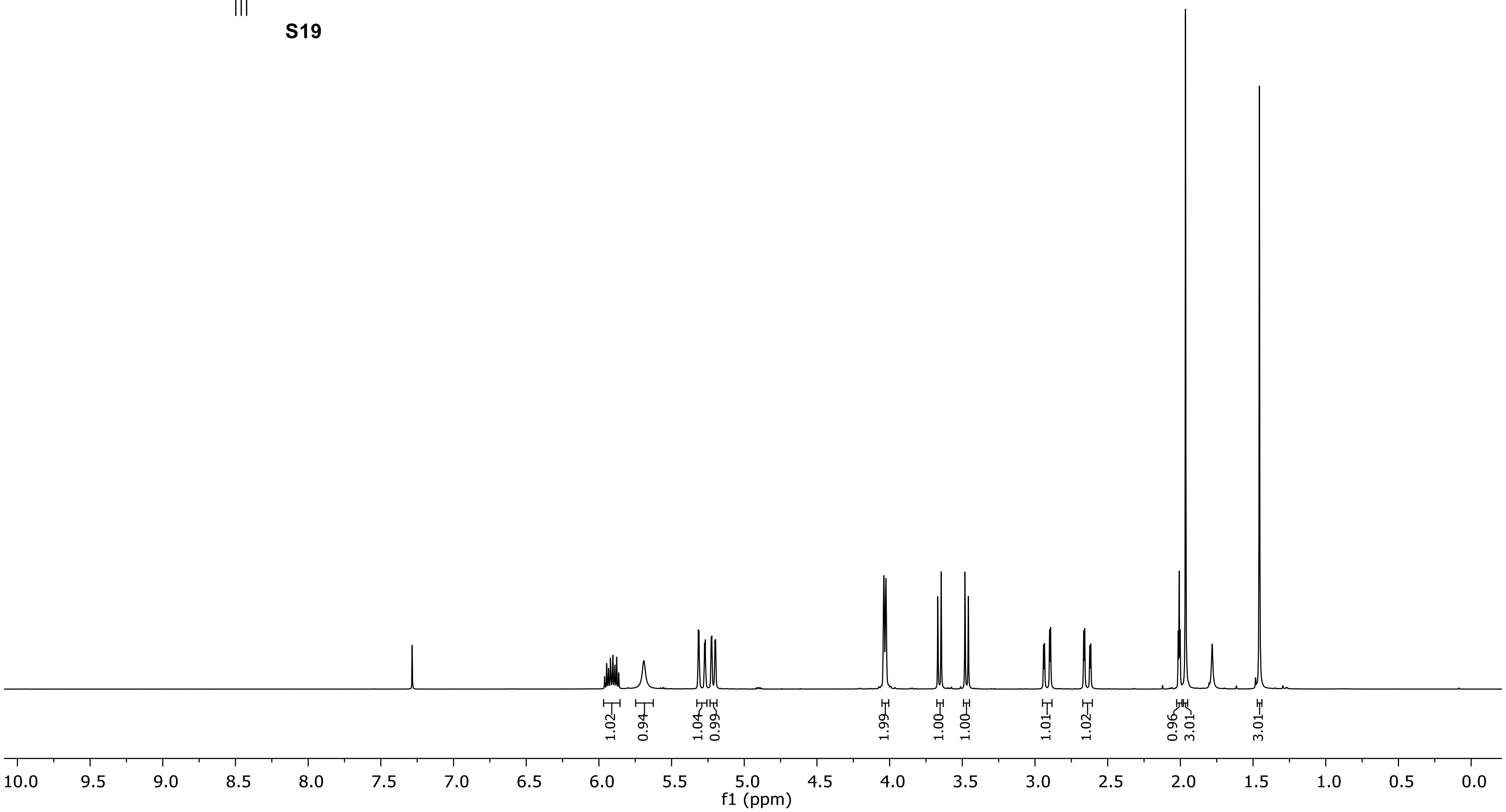

1.02
0.94
1.04
0.99
1.99
1.00
1.00
1.01
1.02
0.96
3.01
3.01
10.0 9.5 9.0 8.5 8.0 7.5 7.0 6.5 6.0 5.5 5.0 4.5 4.0 3.5 3.0 2.5 2.0 1.5 1.0 0.5 0.0
f1 (ppm)

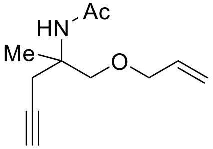

S19

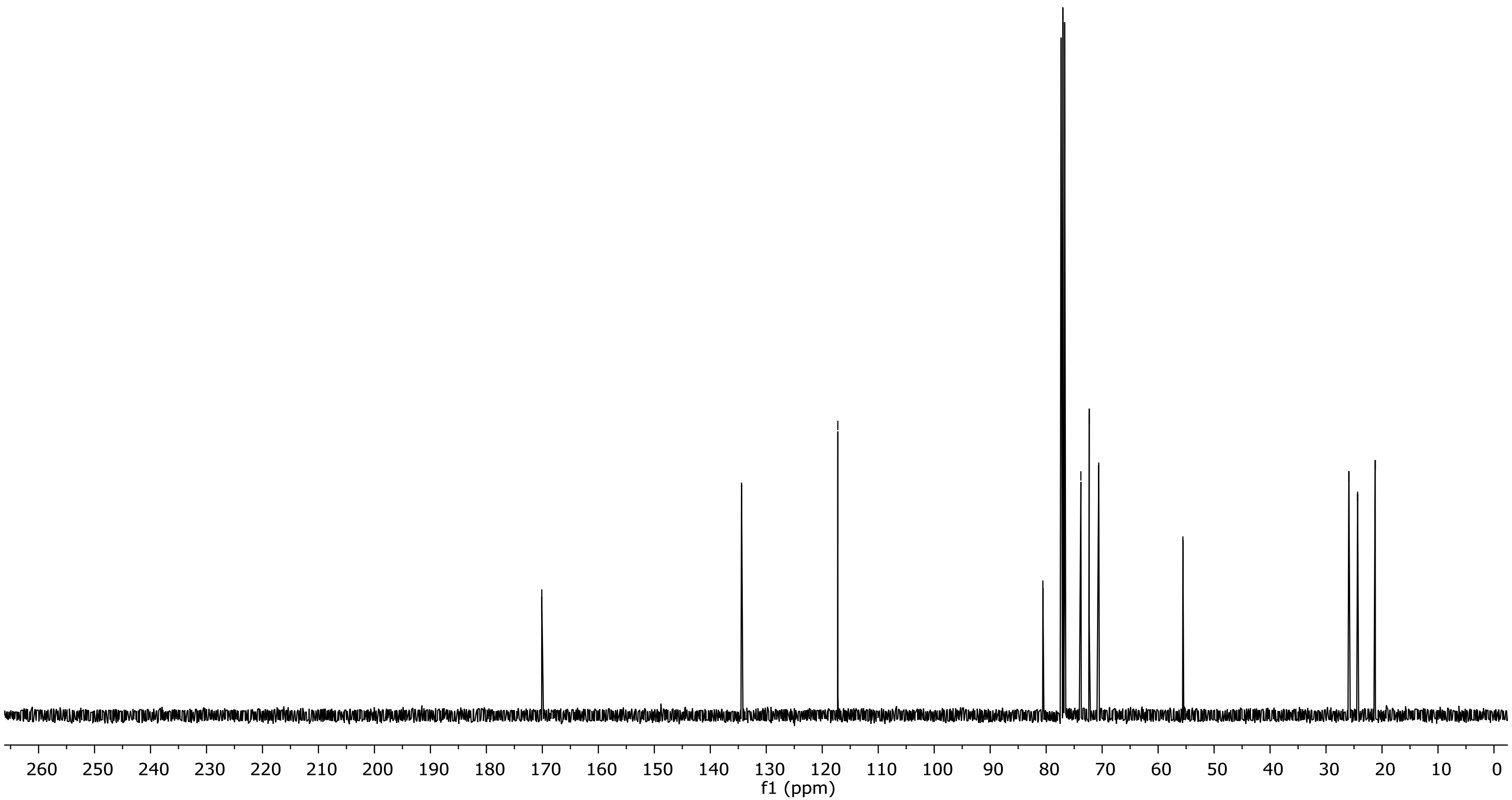

170.1
134.4
117.2
80.6
73.8
72.3
70.6
55.6
25.9
24.4
21.2
260 250 240 230 220 210 200 190 180 170 160 150 140 130 120 110 100 90 80 70 60 50 40 30 20 10 0
f1 (ppm)

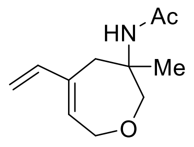
HN
Ac
Me
O

S20

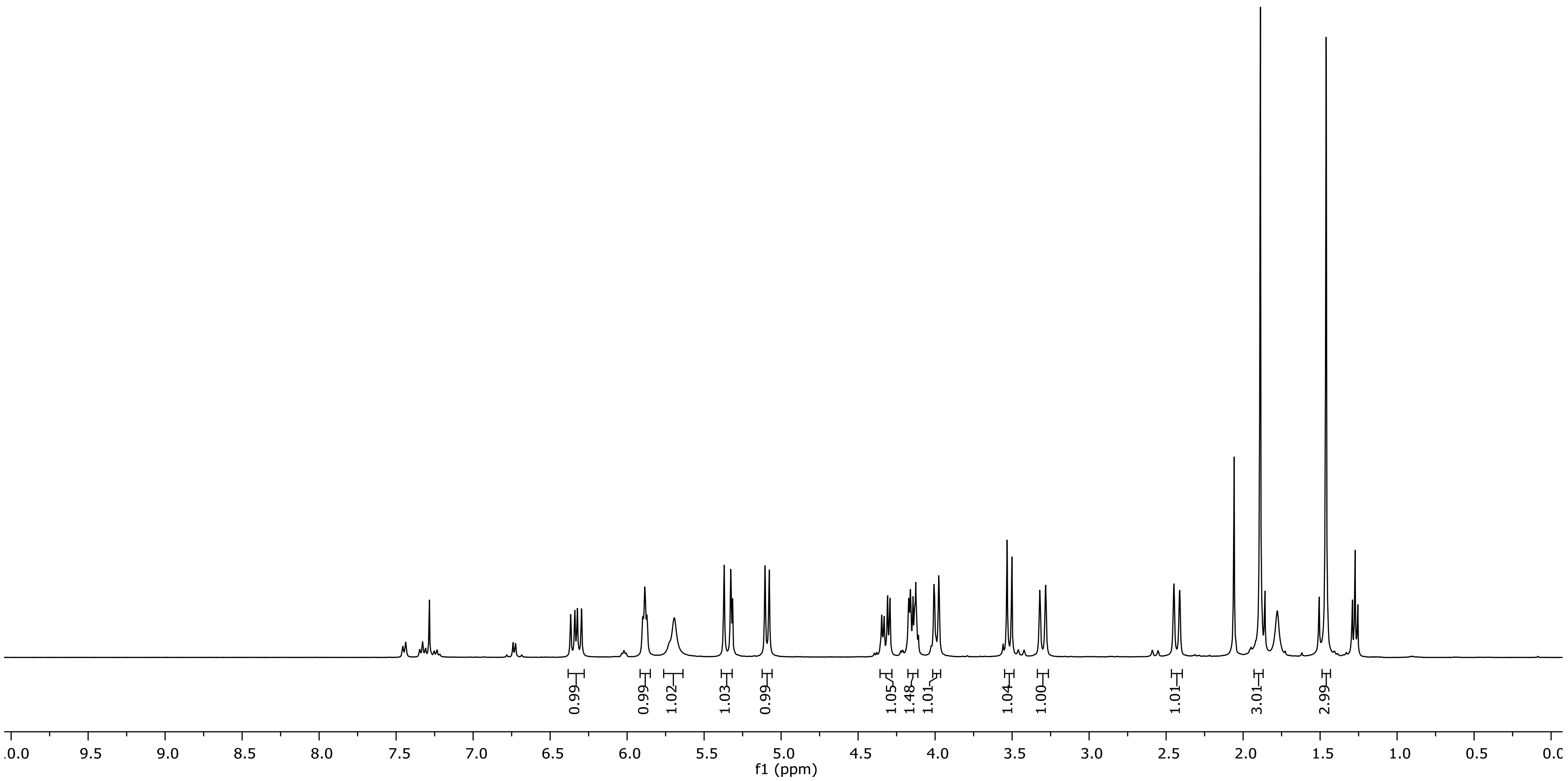
0.99
0.99
1.02
1.03
0.99
1.05
1.48
1.01
1.04
1.00
1.01
3.01
2.99
.0.0 9.5 9.0 8.5 8.0 7.5 7.0 6.5 6.0 5.5 5.0 4.5 4.0 3.5 3.0 2.5 2.0 1.5 1.0 0.5 0.C
f1 (ppm)

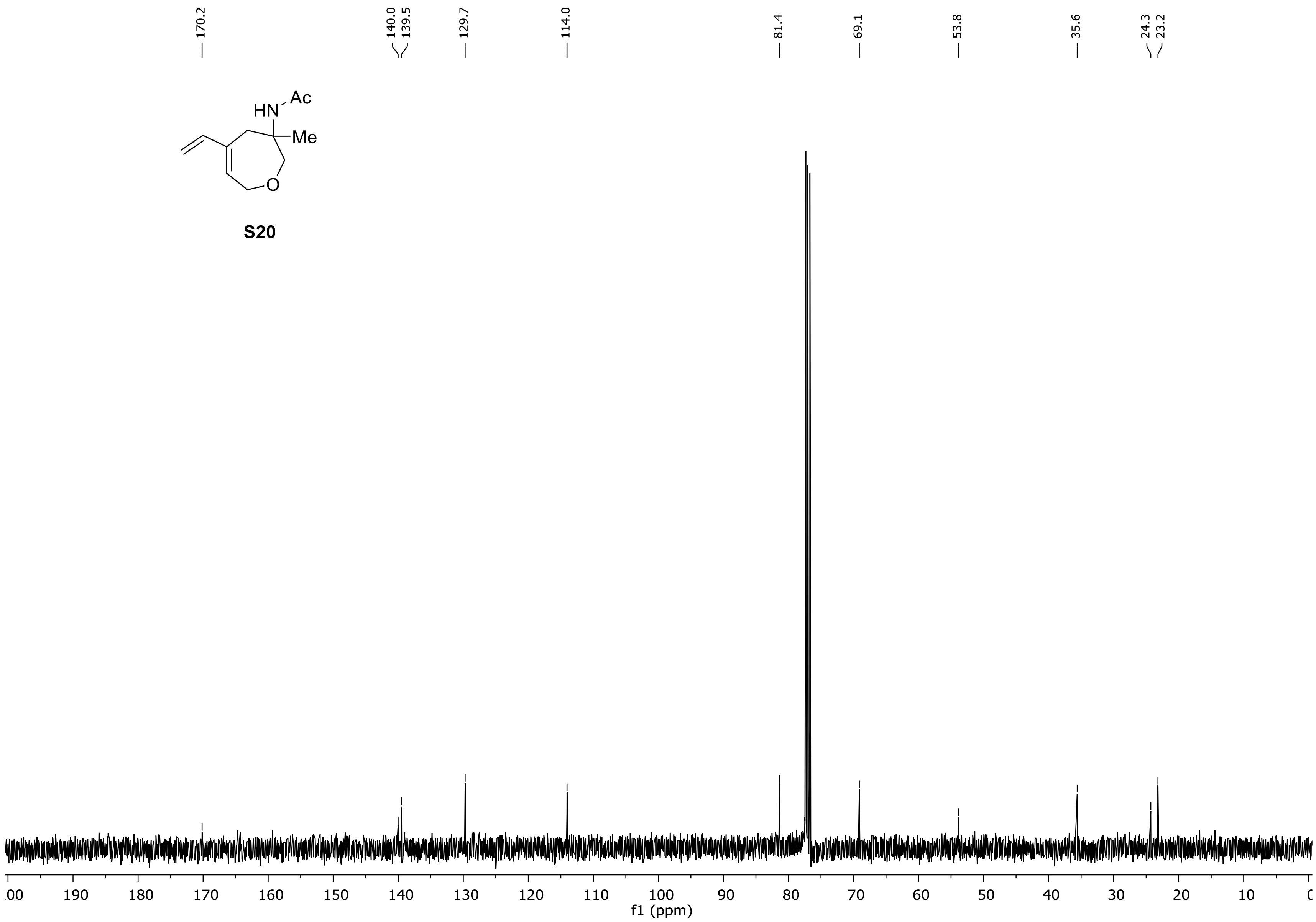
170.2
140.0
139.5
129.7
114.0
81.4
69.1
53.8
35.6
24.3
23.2
HN
Ac
Me
O
f1 (ppm)

S20

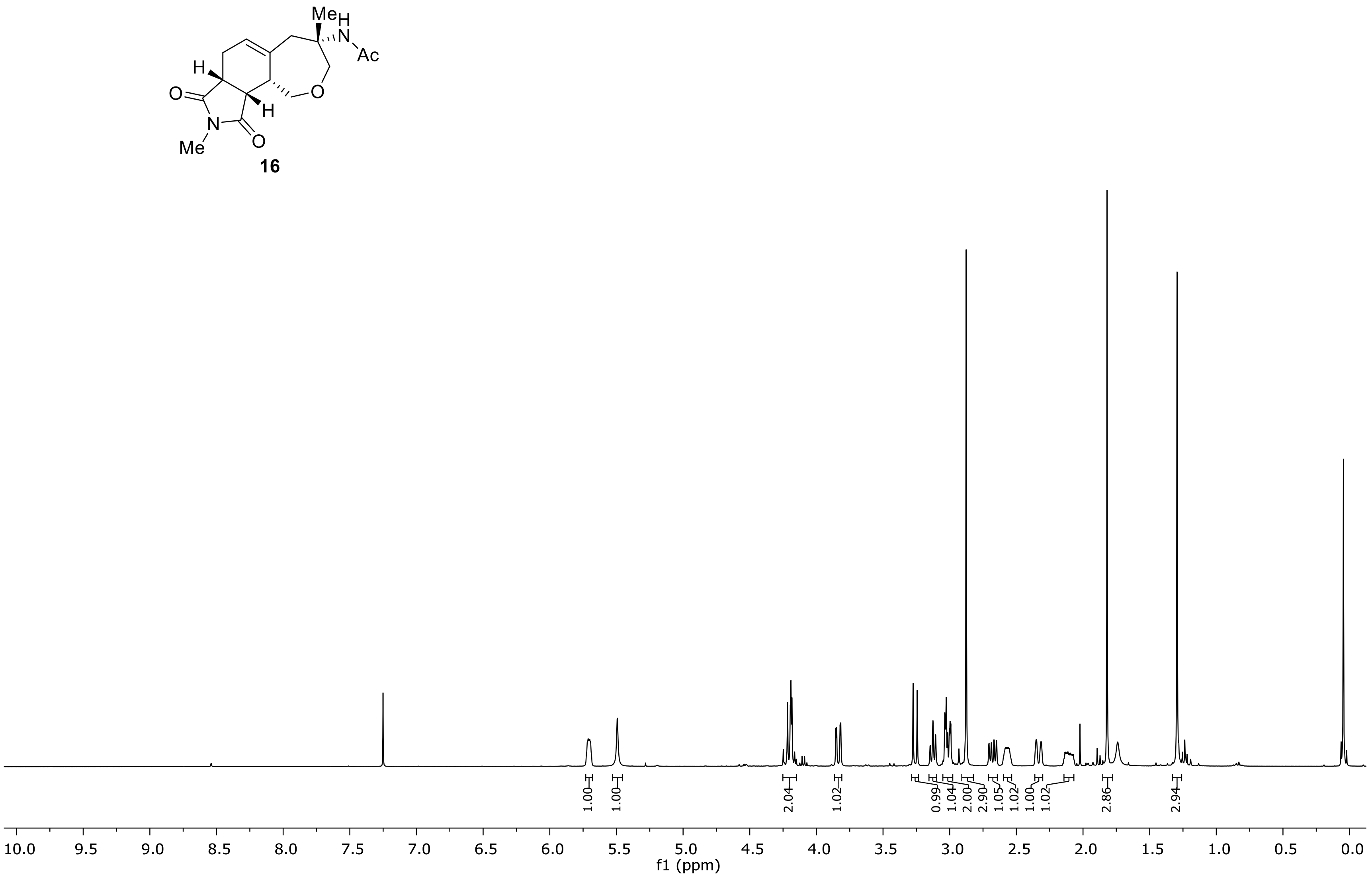
Me
H
N
Ac
H
O
O
N
Me
O
H
16
1.00
1.00
2.04
1.02
0.99
1.04
2.00
2.90
1.05
1.02
1.00
1.02
2.86
2.94
10.0
9.5
9.0
8.5
8.0
7.5
7.0
6.5
6.0
5.5
5.0
4.5
4.0
3.5
3.0
2.5
2.0
1.5
1.0
0.5
0.0
f1 (ppm)

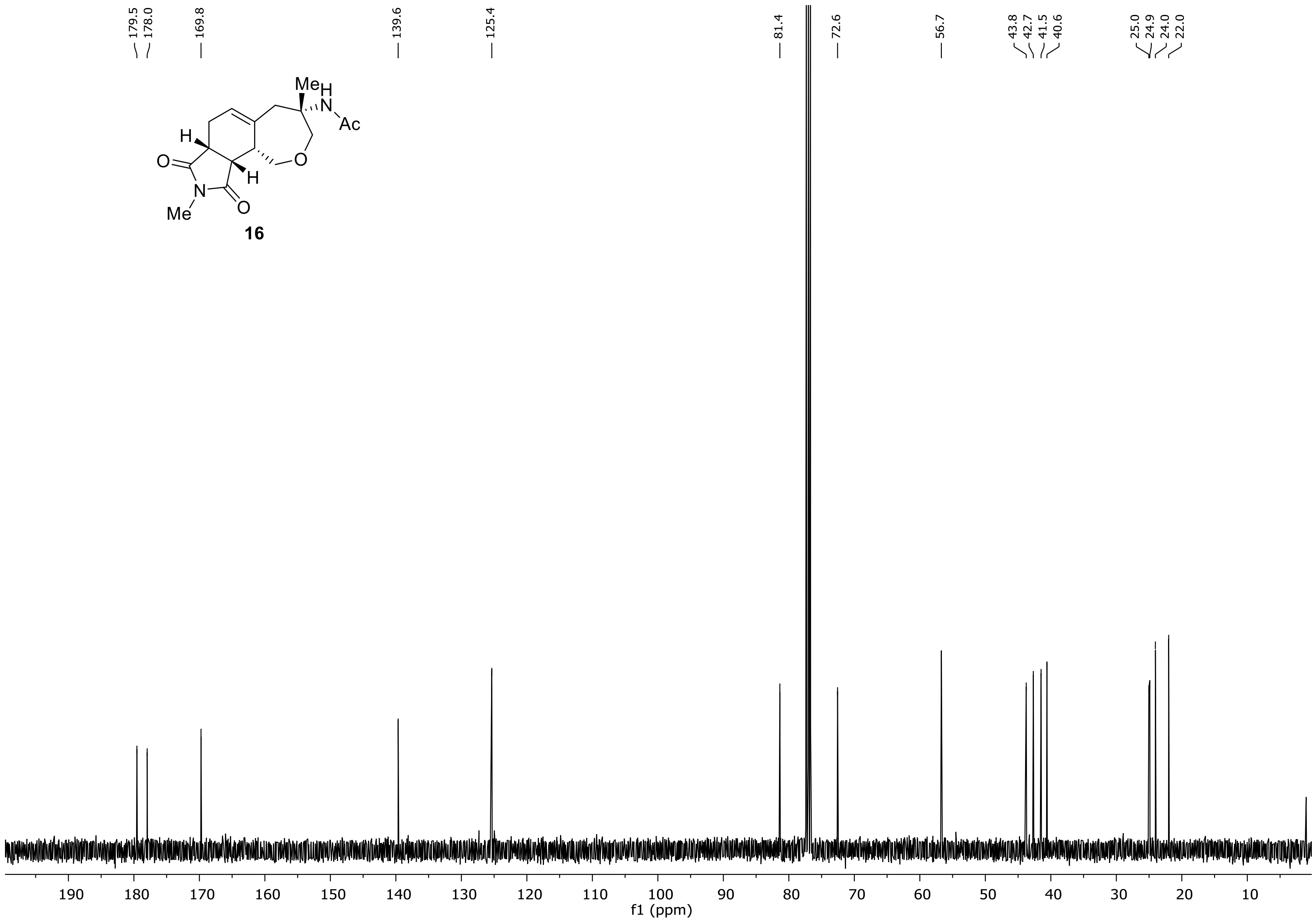
179.5
178.0
169.8
139.6
125.4
81.4
72.6
56.7
43.8
42.7
41.5
40.6
25.0
24.9
24.0
22.0
Me
H
N
Ac
H
O
O
H
N
Me
O
16
190 180 170 160 150 140 130 120 110 100 90 80 70 60 50 40 30 20 10
f1 (ppm)

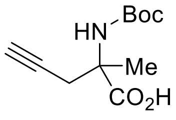
HN
Boc
Me
CO2H

S21

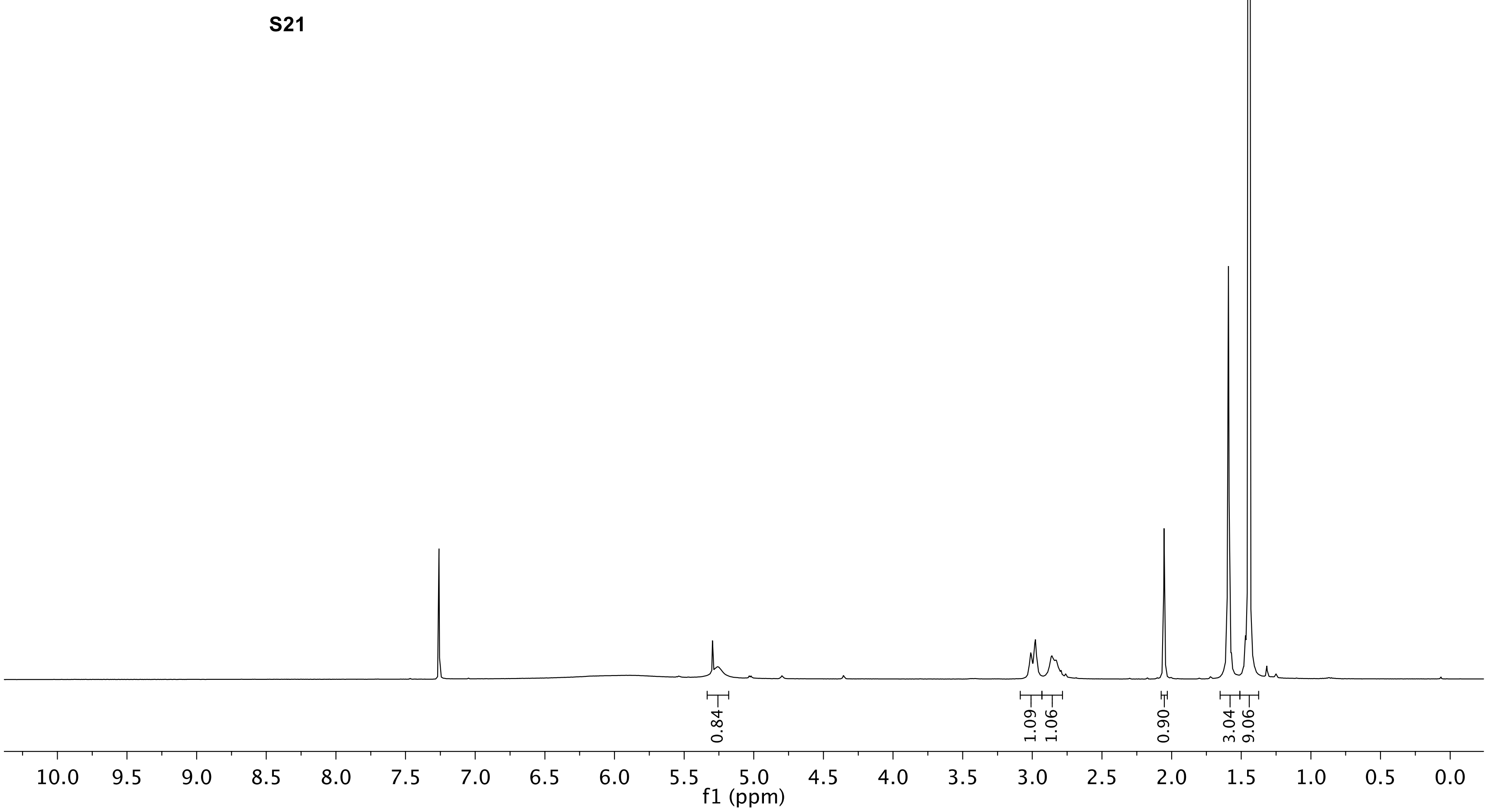
0.84
1.09
1.06
0.90
3.04
9.06
10.0 9.5 9.0 8.5 8.0 7.5 7.0 6.5 6.0 5.5 5.0 4.5 4.0 3.5 3.0 2.5 2.0 1.5 1.0 0.5 0.0
f1 (ppm)

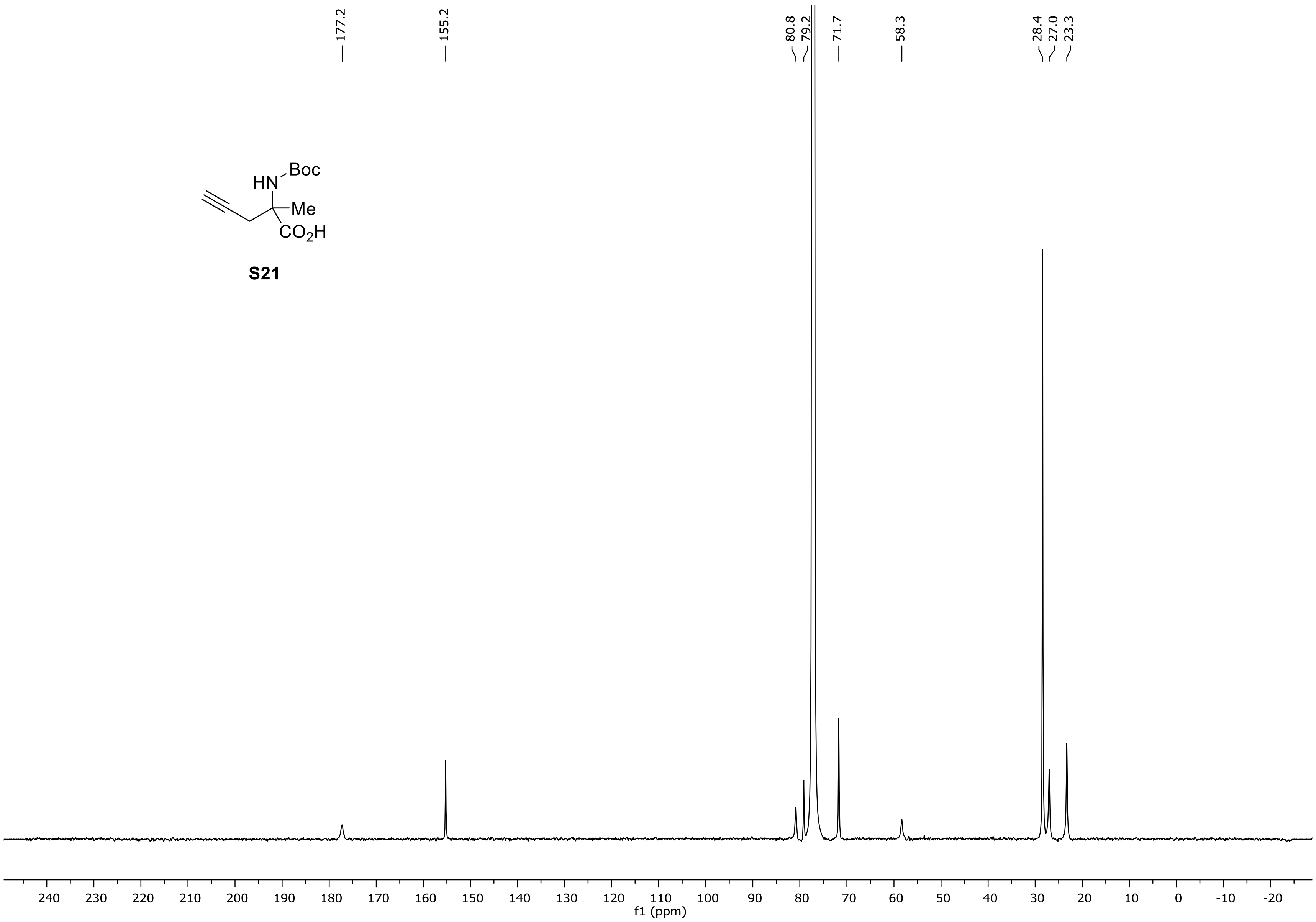
HN
Boc
Me
CO2H
177.2
155.2
80.8
79.2
71.7
58.3
28.4
27.0
23.3
240 230 220 210 200 190 180 170 160 150 140 130 120 110 100 90 80 70 60 50 40 30 20 10 0 -10 -20
f1 (ppm)

S21

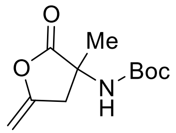
O
Me
O
N
Boc
H

17

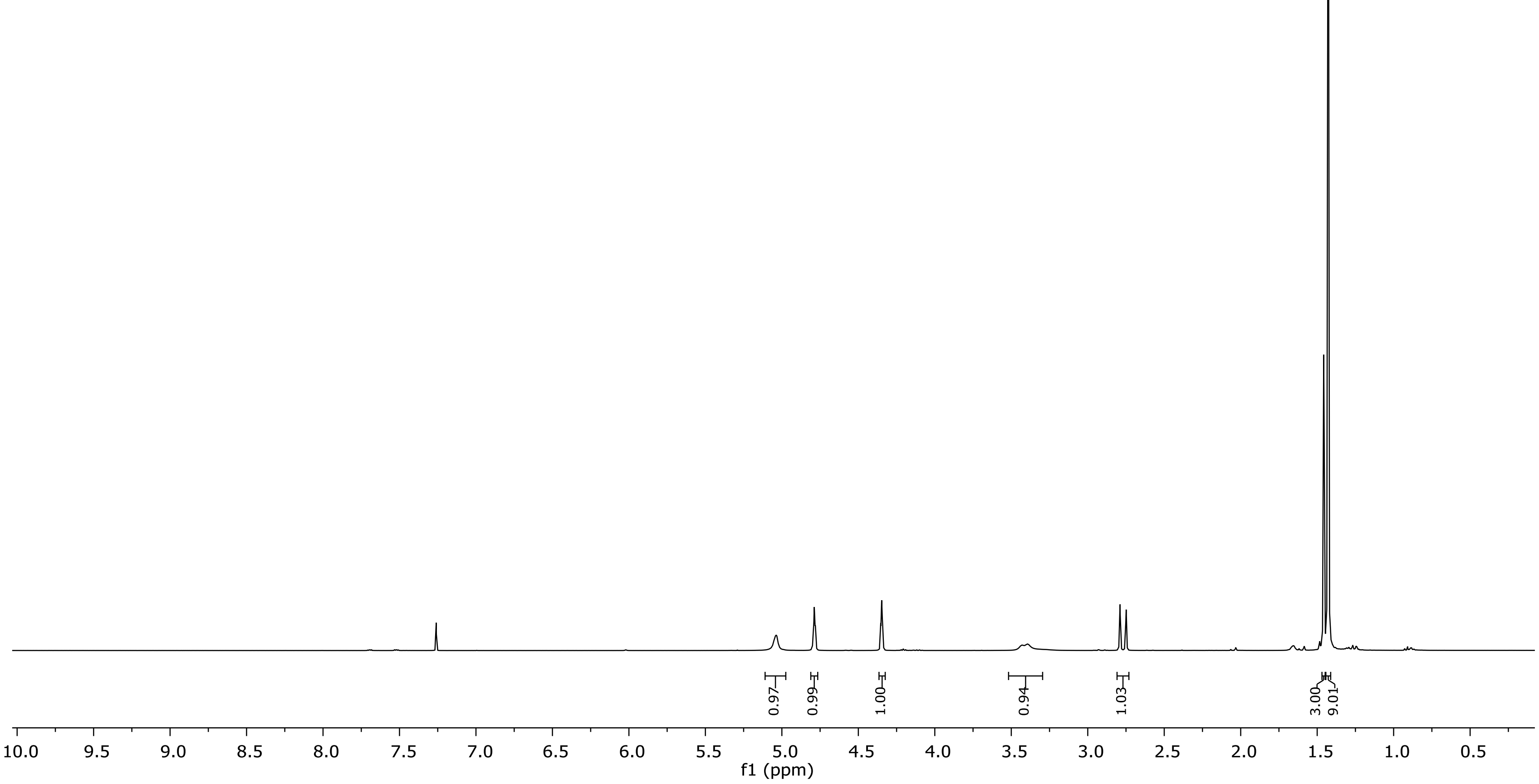
0.97
0.99
1.00
0.94
1.03
3.00
9.01
10.0
9.5
9.0
8.5
8.0
7.5
7.0
6.5
6.0
5.5
5.0
4.5
4.0
3.5
3.0
2.5
2.0
1.5
1.0
0.5
f1 (ppm)

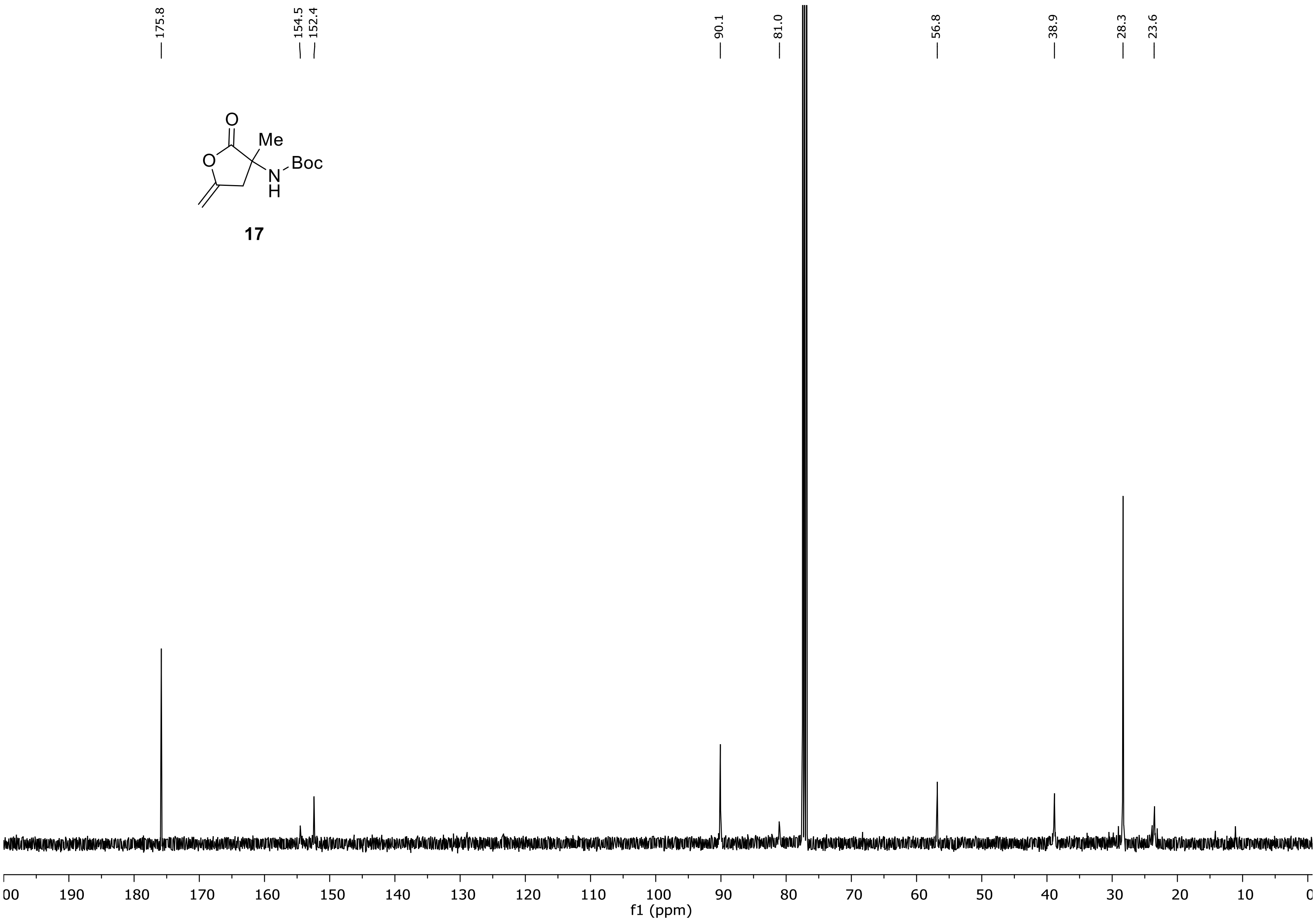

175.8
154.5
152.4
90.1
81.0
56.8
38.9
28.3
23.6
O
Me
O
N
Boc
H
17
200 190 180 170 160 150 140 130 120 110 100 90 80 70 60 50 40 30 20 10 0
f1 (ppm)

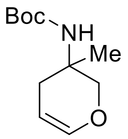
Boc
NH
Me
O

18

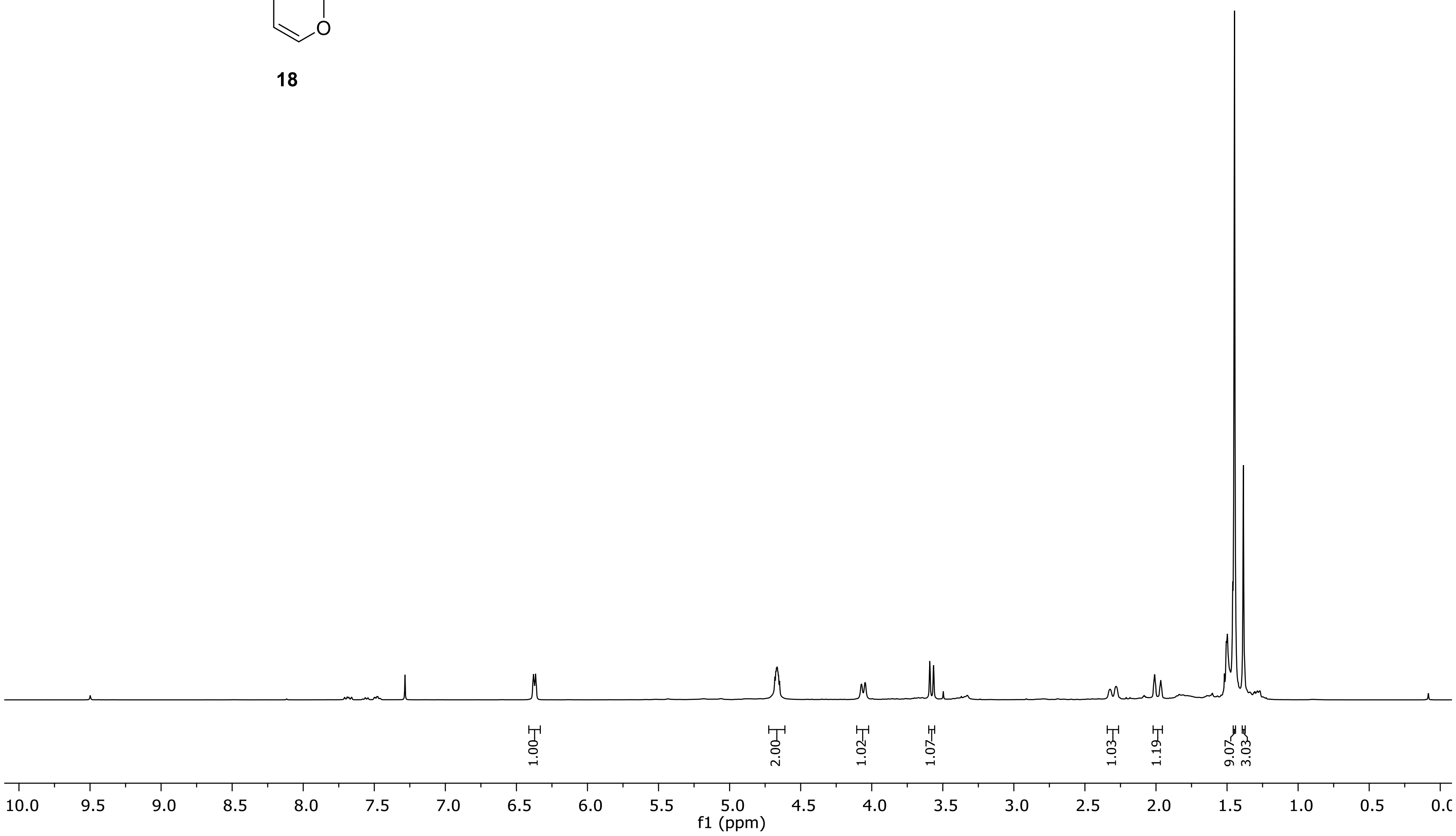
1.00
2.00
1.02
1.07
1.03
1.19
9.07
3.03
10.0 9.5 9.0 8.5 8.0 7.5 7.0 6.5 6.0 5.5 5.0 4.5 4.0 3.5 3.0 2.5 2.0 1.5 1.0 0.5 0.0
f1 (ppm)

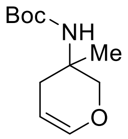
Boc
NH
Me
O

18

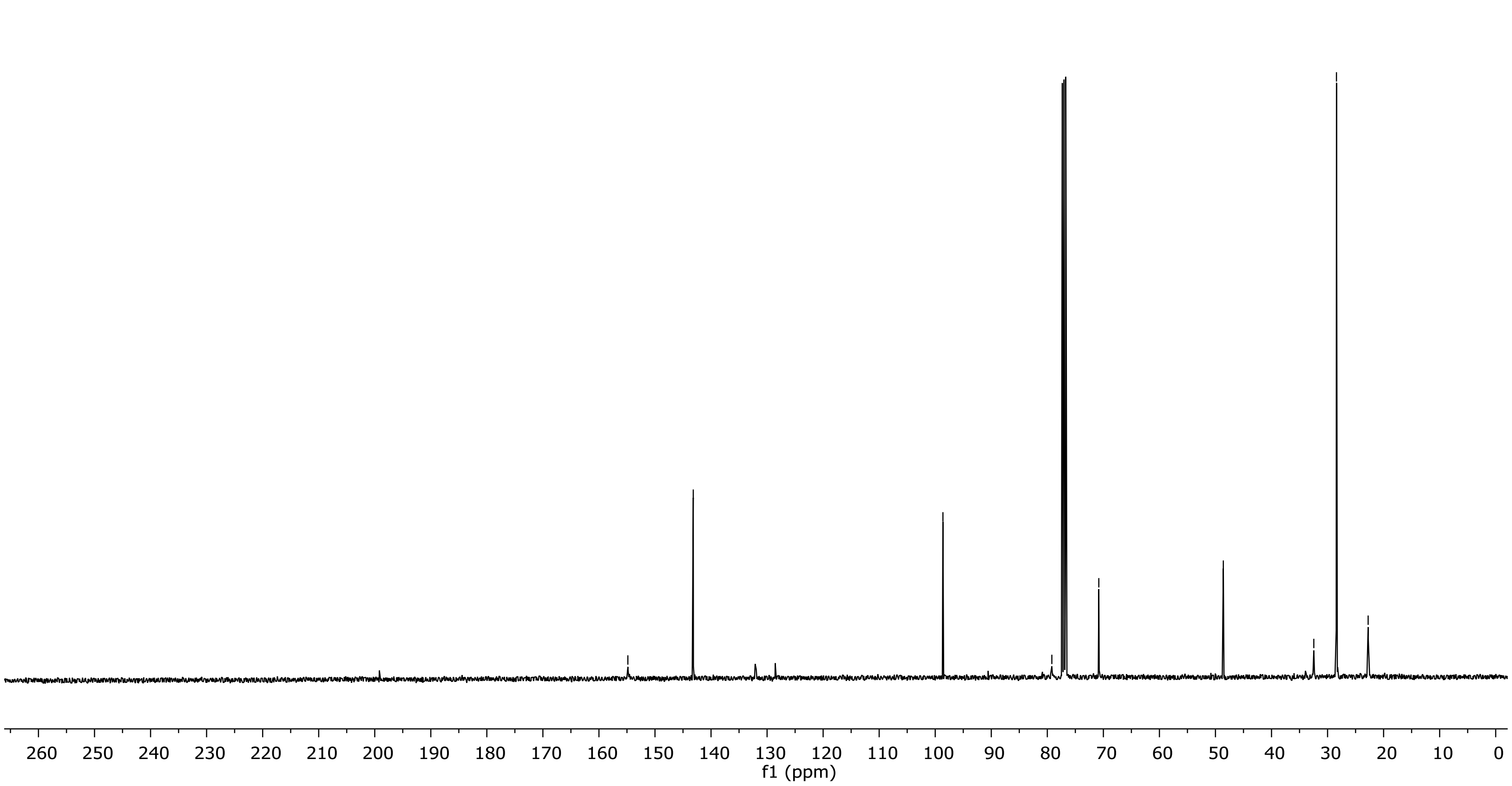
154.8
143.2
98.6
79.2
70.8
48.6
32.5
28.4
22.8
260 250 240 230 220 210 200 190 180 170 160 150 140 130 120 110 100 90 80 70 60 50 40 30 20 10 0
f1 (ppm)

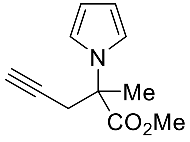
N
Me
CO2Me

19

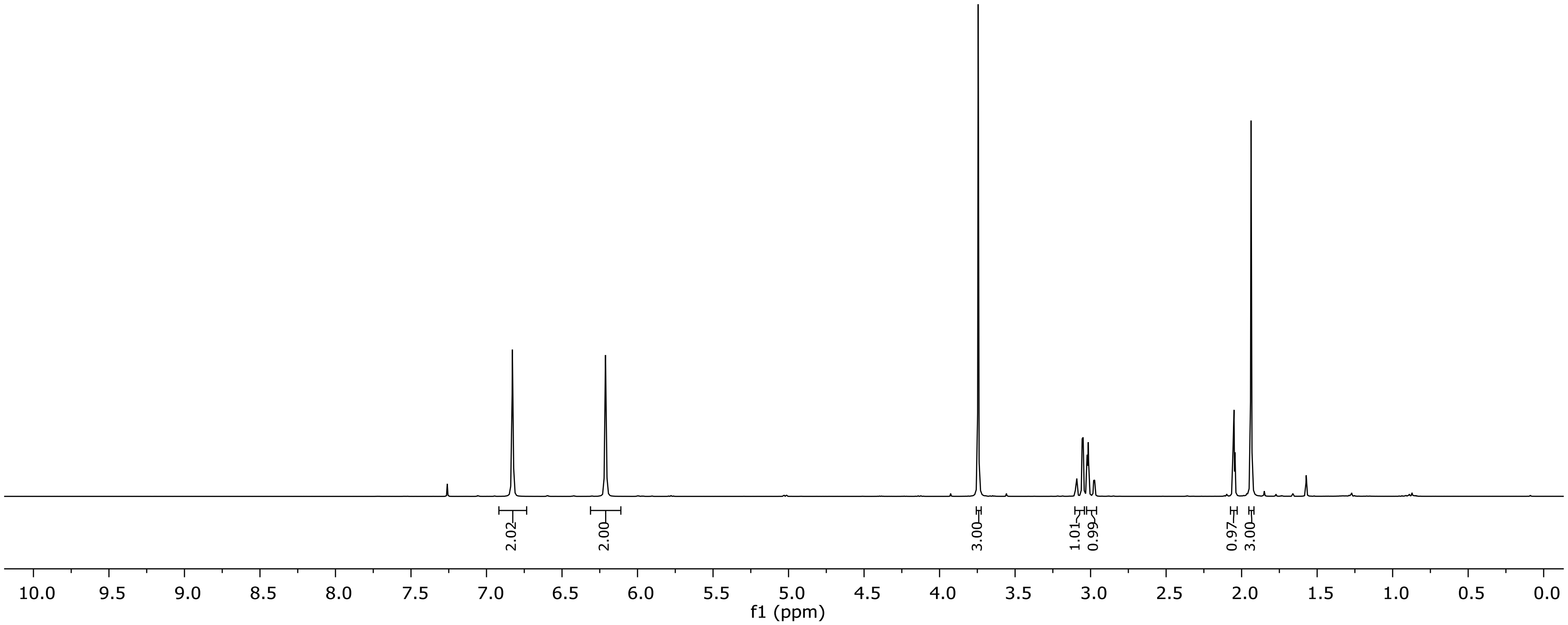
2.02
2.00
3.00
1.01
0.99
0.97
3.00
10.0 9.5 9.0 8.5 8.0 7.5 7.0 6.5 6.0 5.5 5.0 4.5 4.0 3.5 3.0 2.5 2.0 1.5 1.0 0.5 0.0
f1 (ppm)

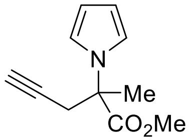

19

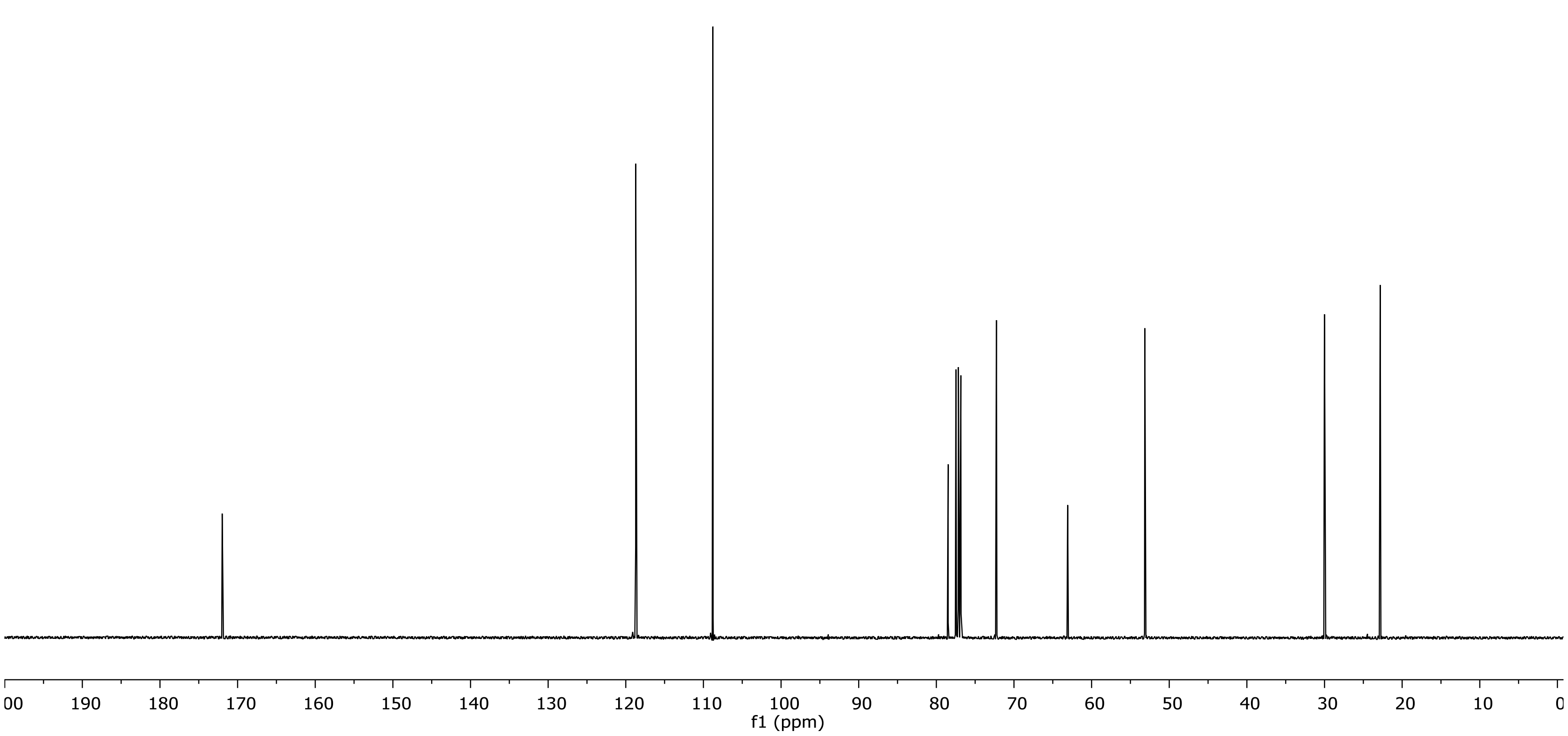

172.0
118.7
108.8
78.5
72.3
63.1
53.1
30.0
22.8
00
190
180
170
160
150
140
130
120
110
100
90
80
70
60
50
40
30
20
10
0
f1 (ppm)

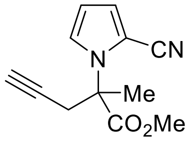
CN
N
Me
CO_2Me

20

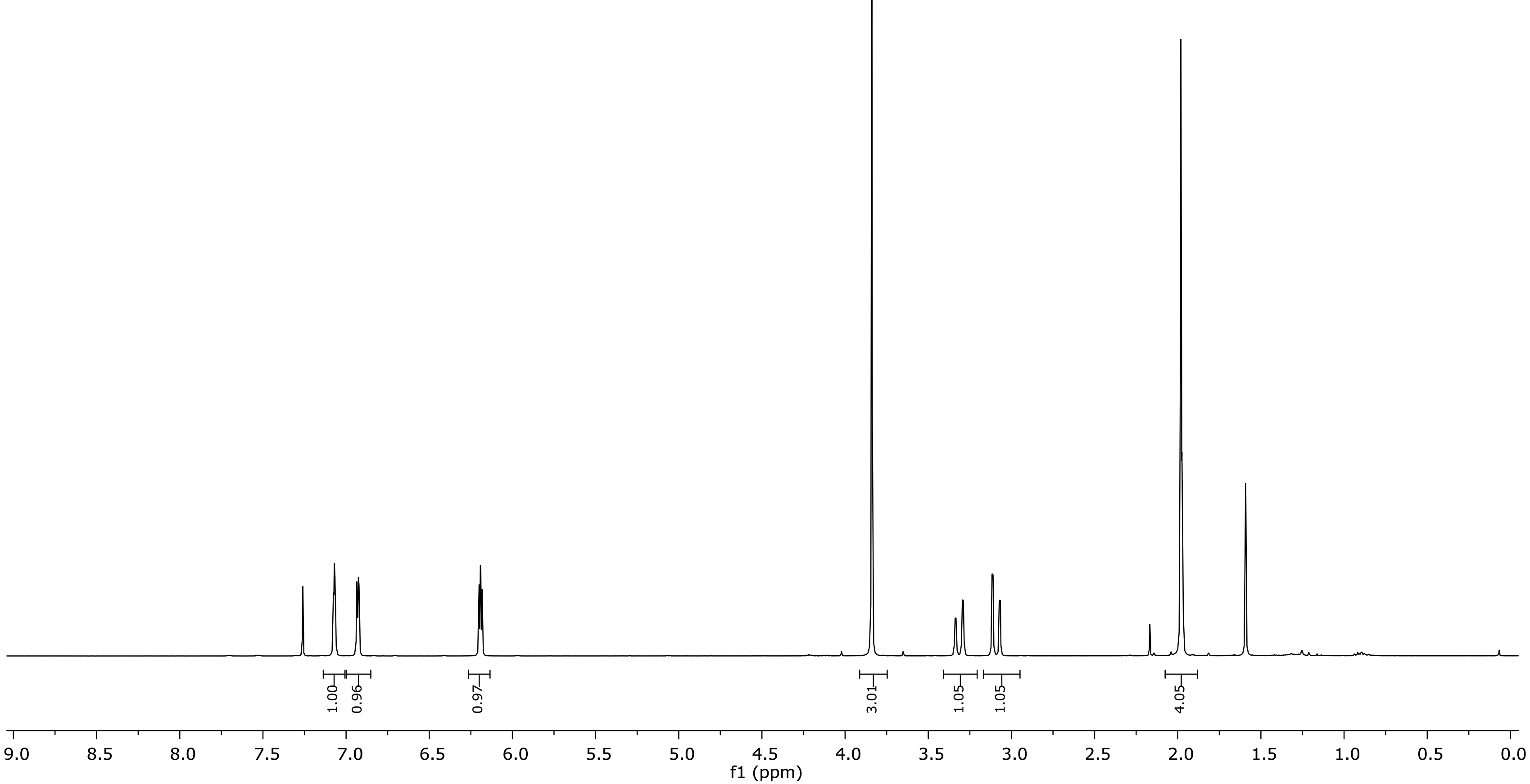
1.00
0.96
0.97
3.01
1.05
1.05
4.05
9.0 8.5 8.0 7.5 7.0 6.5 6.0 5.5 5.0 4.5 4.0 3.5 3.0 2.5 2.0 1.5 1.0 0.5 0.0
f1 (ppm)

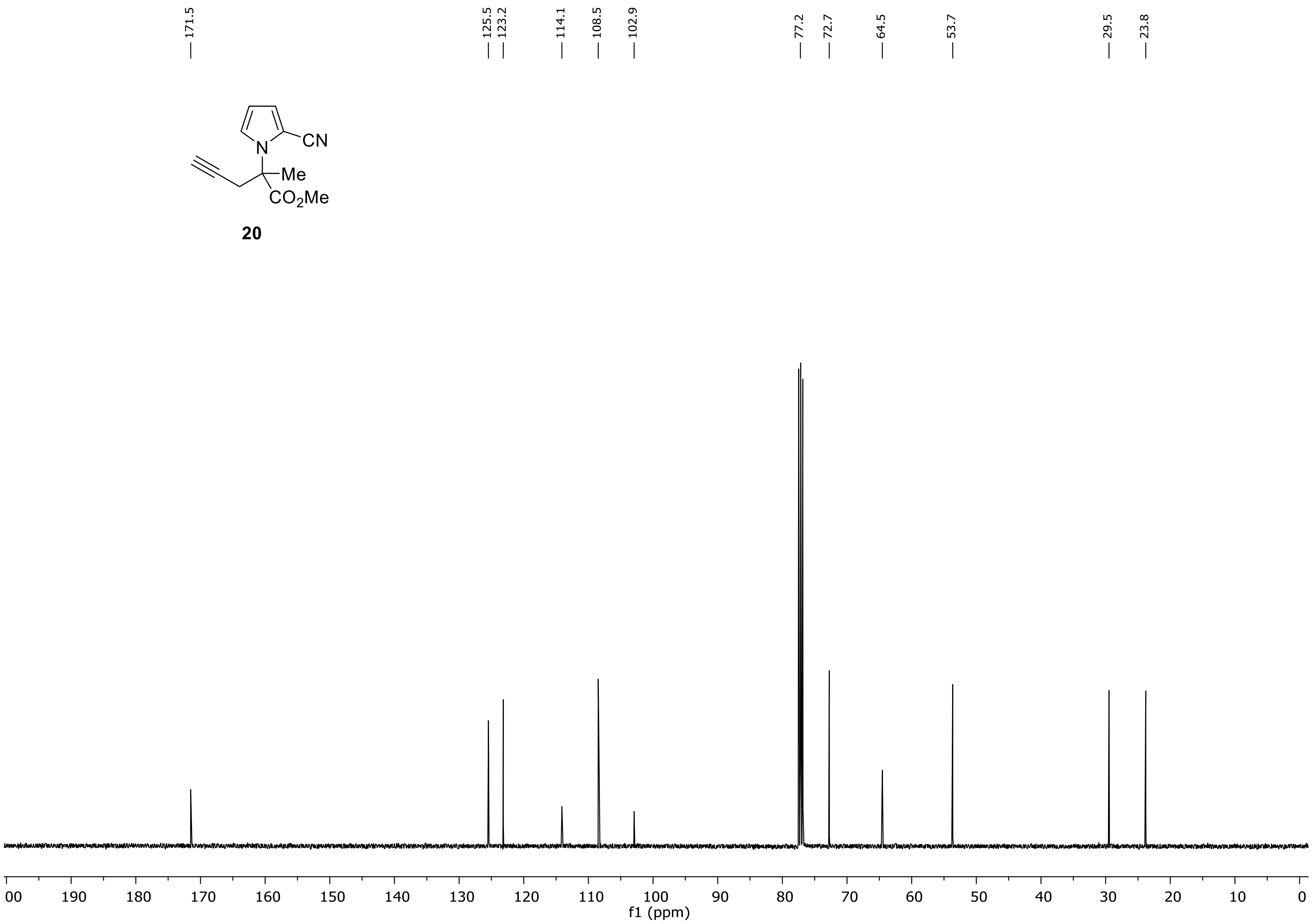
171.5
125.5
123.2
114.1
108.5
102.9
77.2
72.7
64.5
53.7
29.5
23.8
CN
N
Me
CO2Me
20
00
190
180
170
160
150
140
130
120
110
100
90
80
70
60
50
40
30
20
10
0
f1 (ppm)

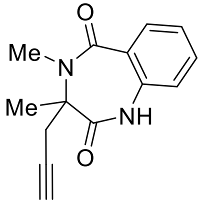
O
Me
N
Me
NH
O

21

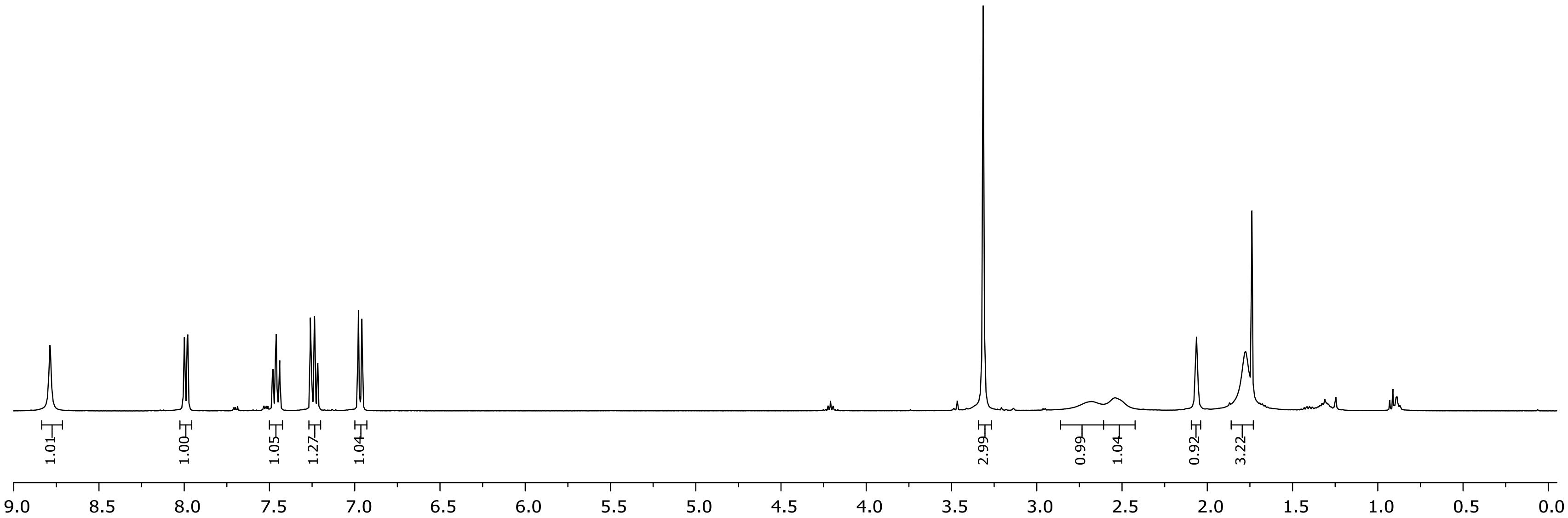
1.01
1.00
1.05
1.27
1.04
2.99
0.99
1.04
0.92
3.22
9.0 8.5 8.0 7.5 7.0 6.5 6.0 5.5 5.0 4.5 4.0 3.5 3.0 2.5 2.0 1.5 1.0 0.5 0.0
f1 (ppm)

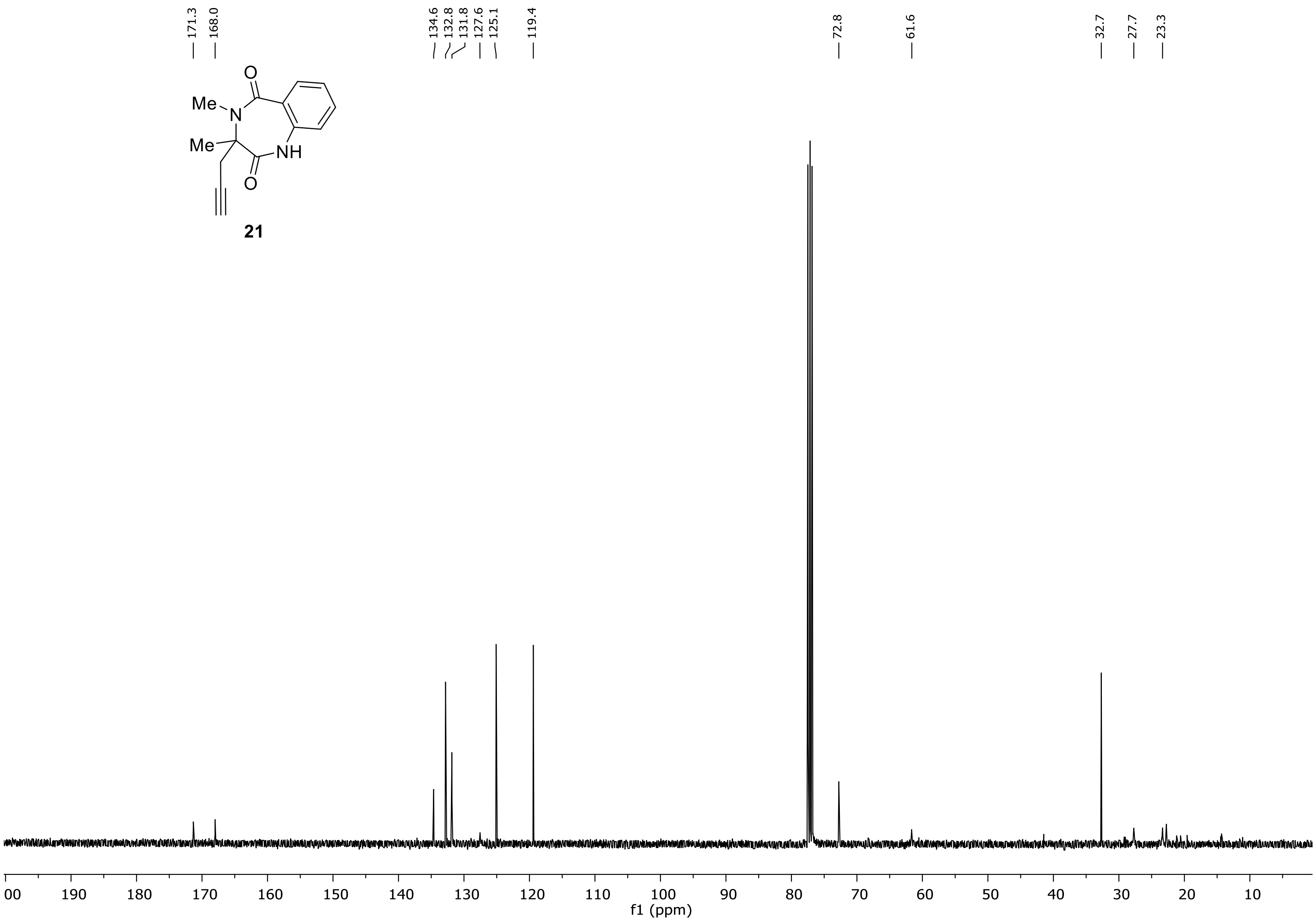

171.3
168.0
134.6
132.8
131.8
127.6
125.1
119.4
72.8
61.6
32.7
27.7
23.3
Me
N
O
Me
NH
O
21
00
190
180
170
160
150
140
130
120
110
100
90
80
70
60
50
40
30
20
10
f1 (ppm)

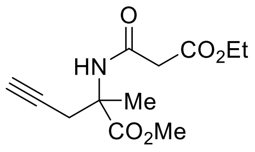

O
CO2Et
HN
Me
CO2Me

S22

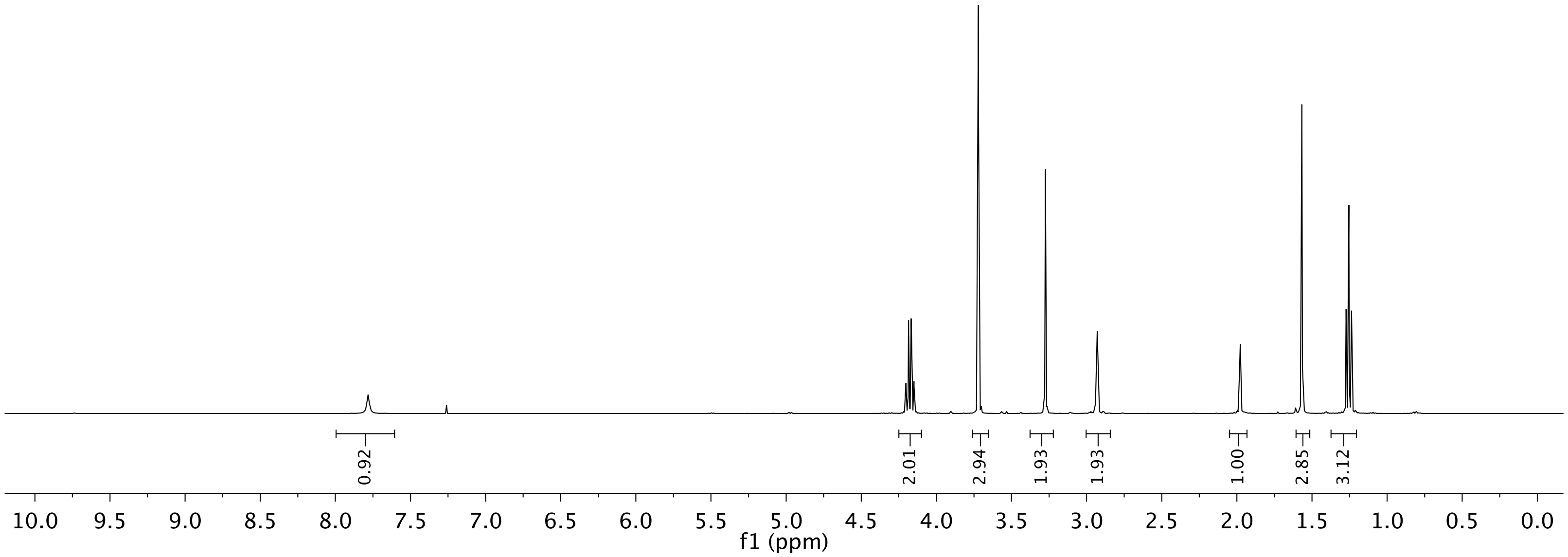

0.92
2.01
2.94
1.93
1.93
1.00
2.85
3.12
10.0 9.5 9.0 8.5 8.0 7.5 7.0 6.5 6.0 5.5 5.0 4.5 4.0 3.5 3.0 2.5 2.0 1.5 1.0 0.5 0.0
f1 (ppm)

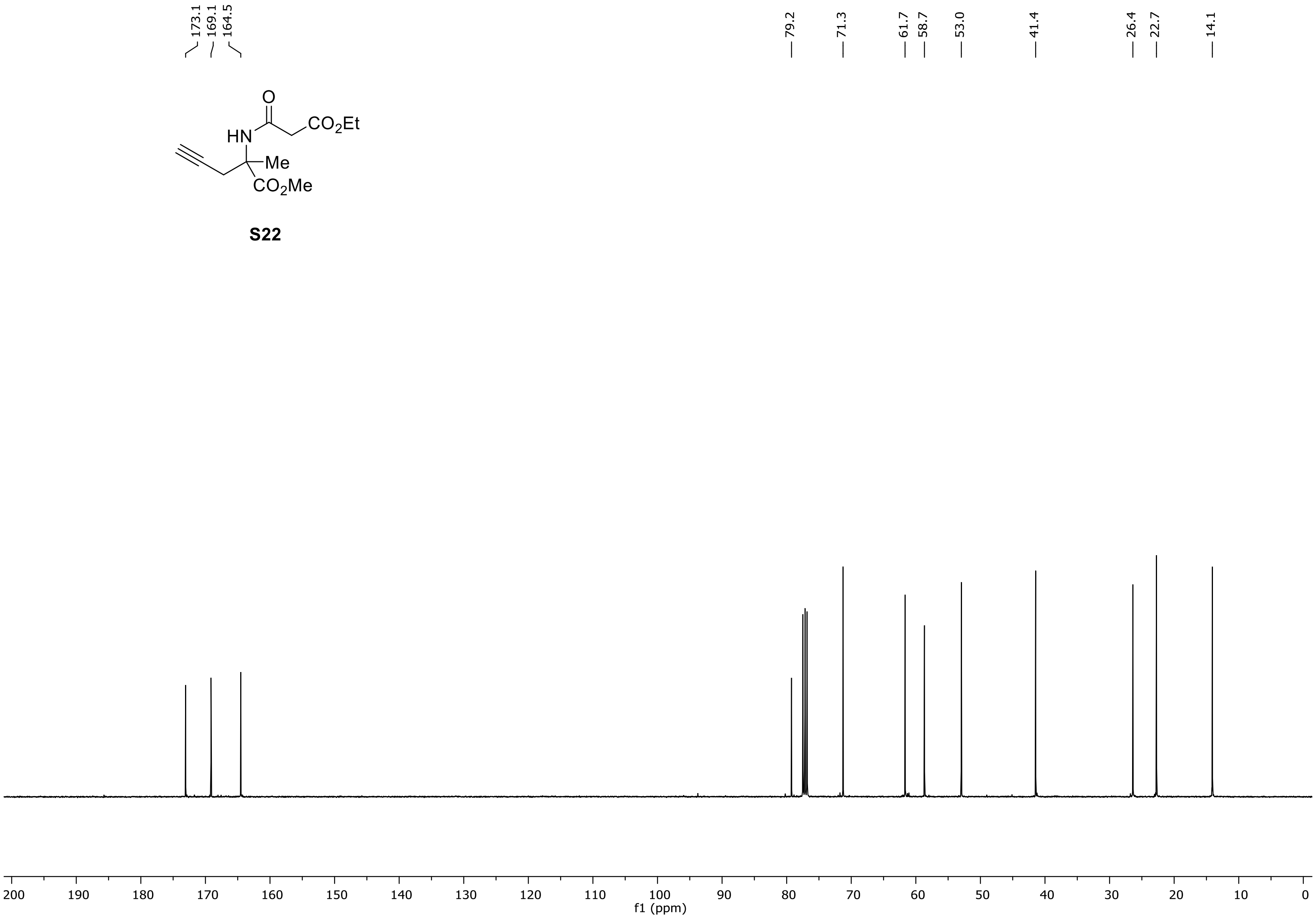

S22

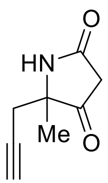
O
HN
Me
O

22

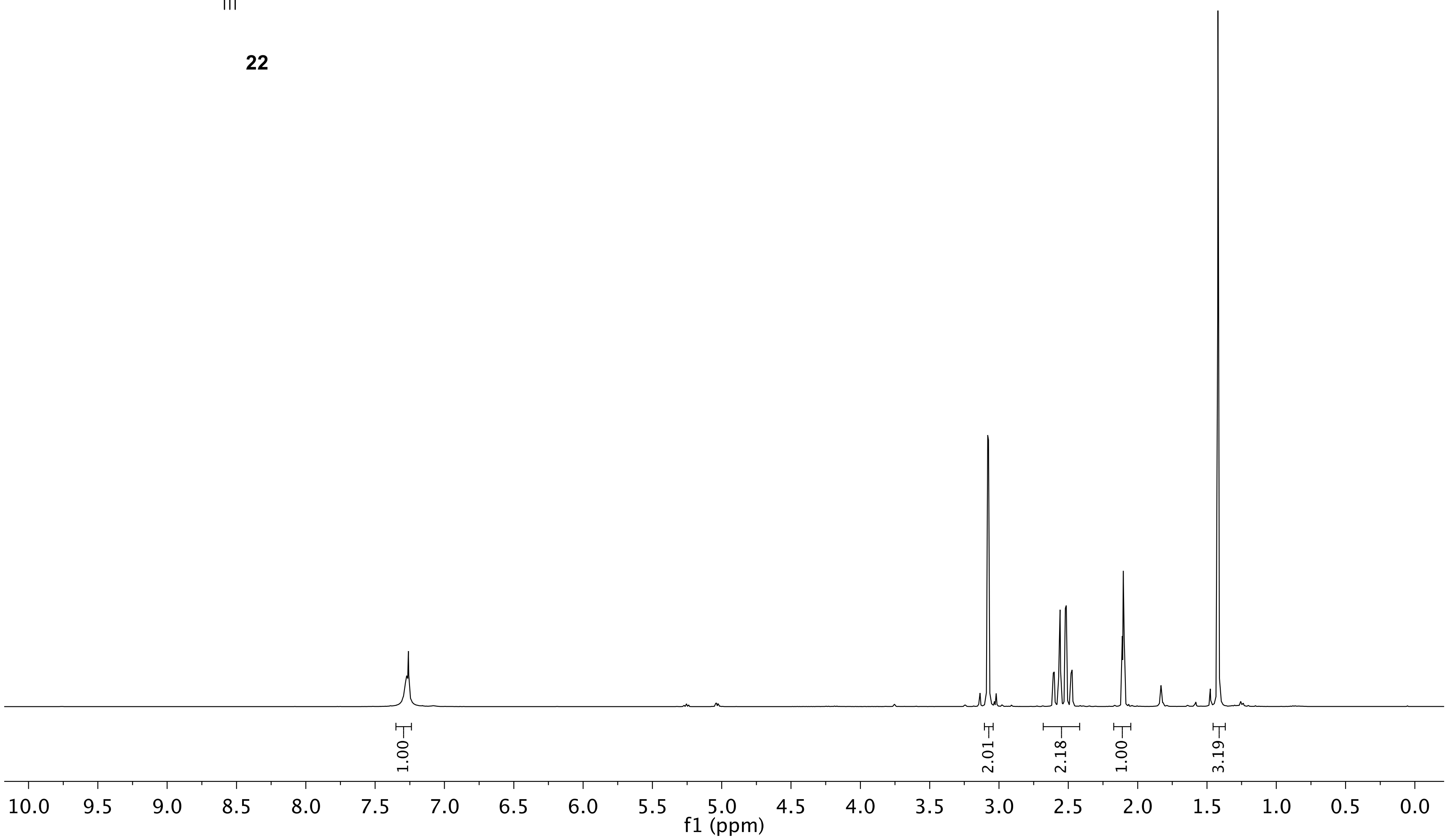
1.00
2.01
2.18
1.00
3.19
10.0 9.5 9.0 8.5 8.0 7.5 7.0 6.5 6.0 5.5 5.0 4.5 4.0 3.5 3.0 2.5 2.0 1.5 1.0 0.5 0.0
f1 (ppm)

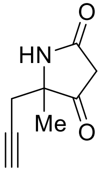

22

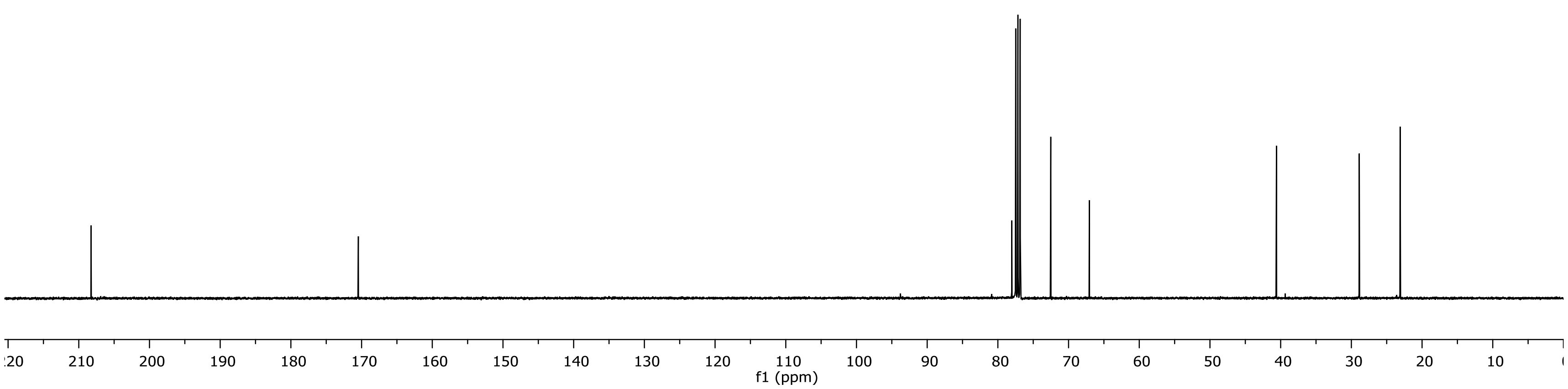

208.3
170.5
78.0
72.5
67.1
40.6
28.9
23.1
220 210 200 190 180 170 160 150 140 130 120 110 100 90 80 70 60 50 40 30 20 10 0
f1 (ppm)

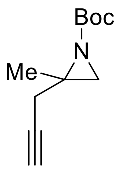
Boc
N
Me

23

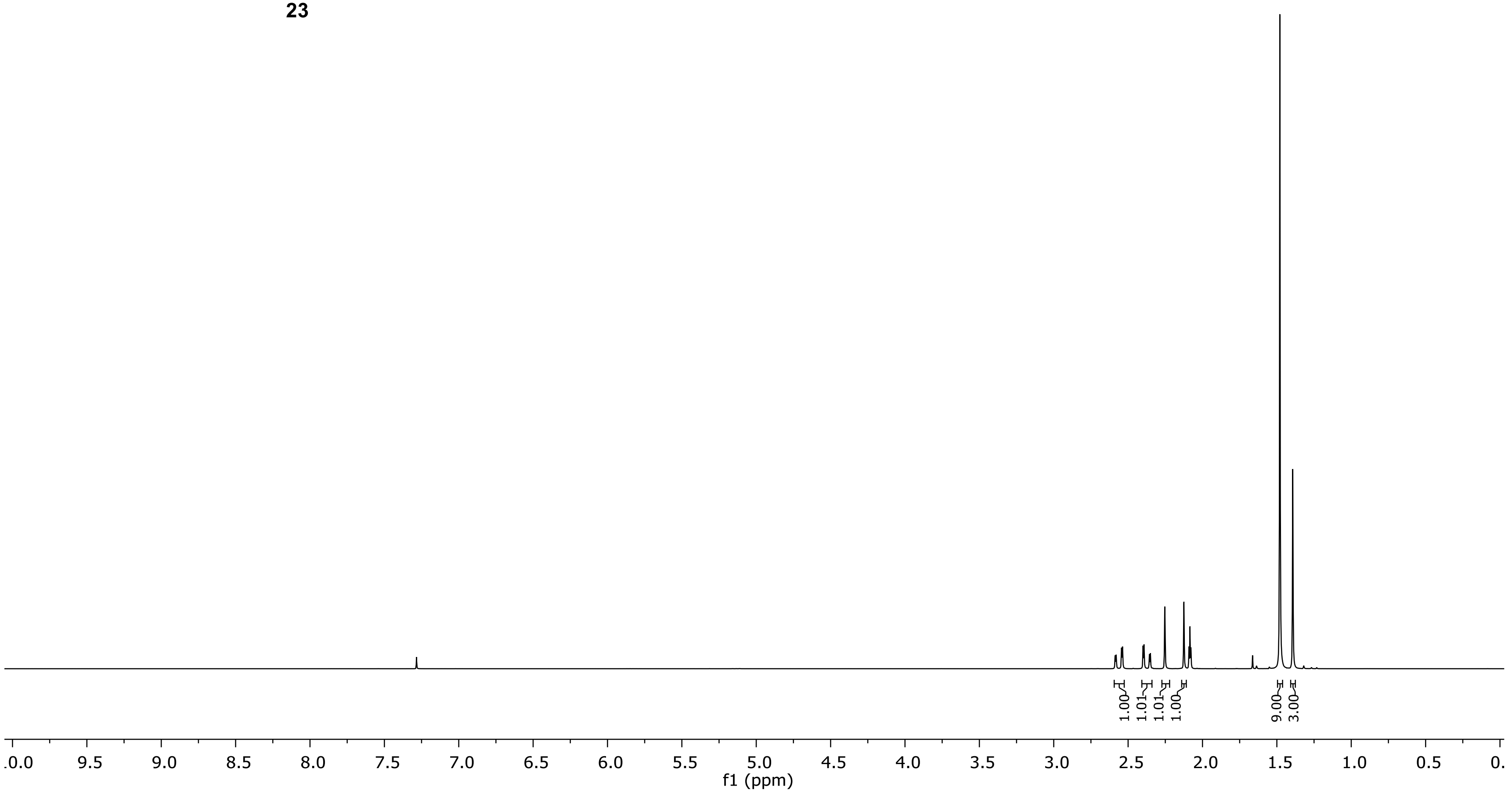
1.00
1.01
1.01
1.00
9.00
3.00
10.0
9.5
9.0
8.5
8.0
7.5
7.0
6.5
6.0
5.5
5.0
4.5
4.0
3.5
3.0
2.5
2.0
1.5
1.0
0.5
0.
f1 (ppm)

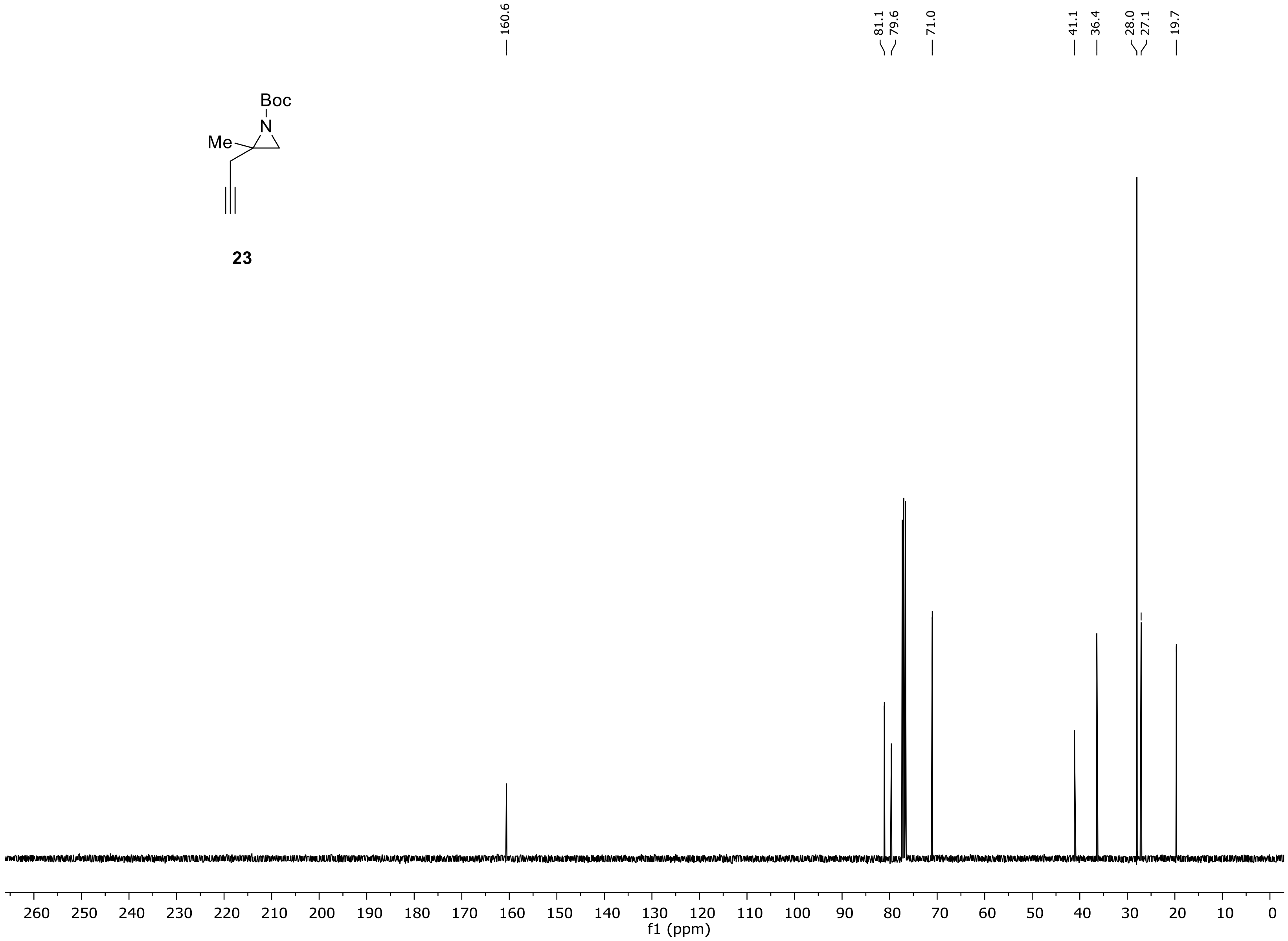

160.6
81.1
79.6
71.0
41.1
36.4
28.0
27.1
19.7
Boc
N
Me
23
260 250 240 230 220 210 200 190 180 170 160 150 140 130 120 110 100 90 80 70 60 50 40 30 20 10 0
f1 (ppm)

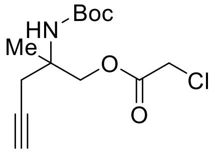
HN
Boc
Me
O
O
Cl

S23

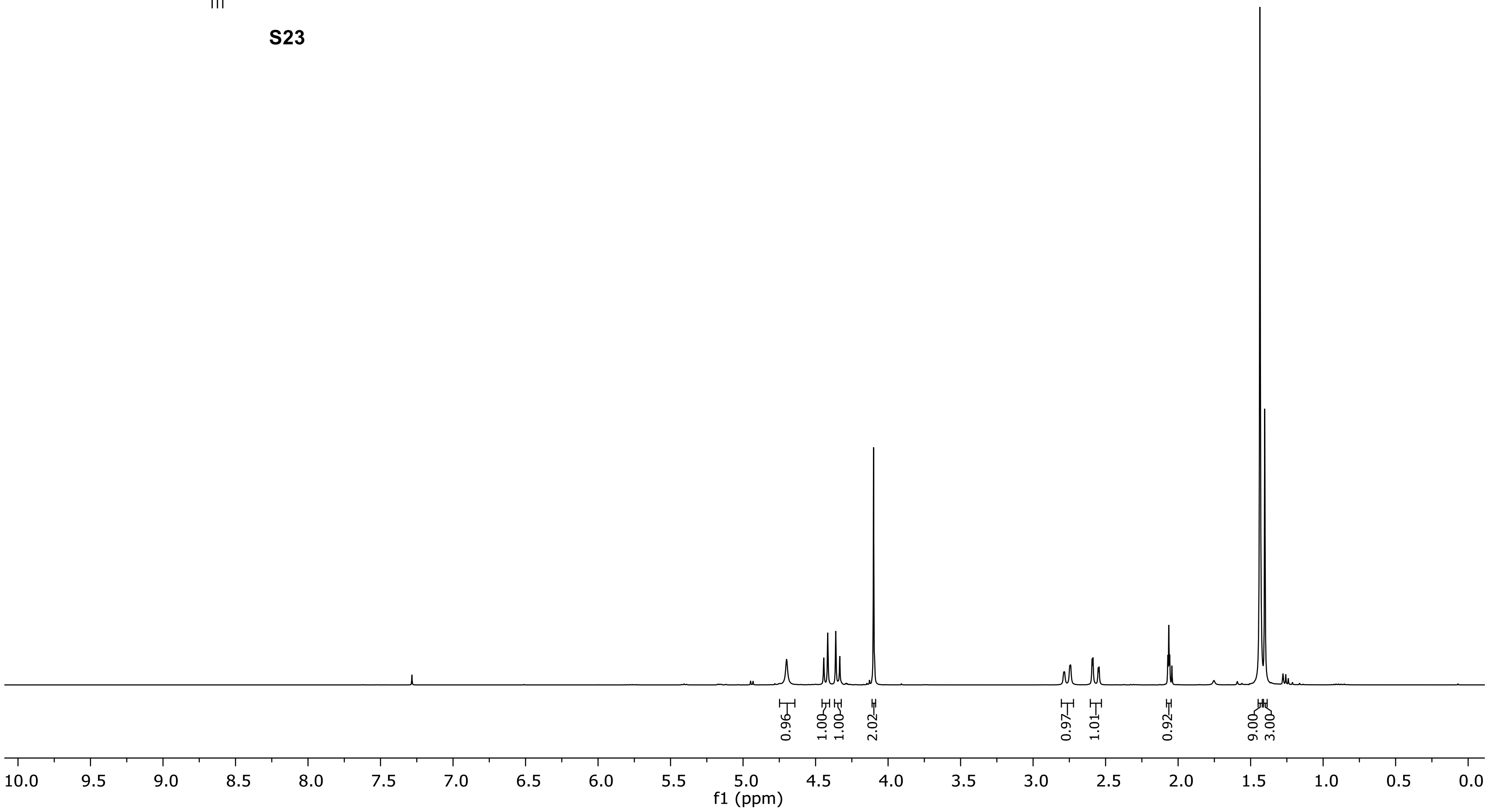
0.96
1.00
1.00
2.02
0.97
1.01
0.92
9.00
3.00
10.0 9.5 9.0 8.5 8.0 7.5 7.0 6.5 6.0 5.5 5.0 4.5 4.0 3.5 3.0 2.5 2.0 1.5 1.0 0.5 0.0
f1 (ppm)

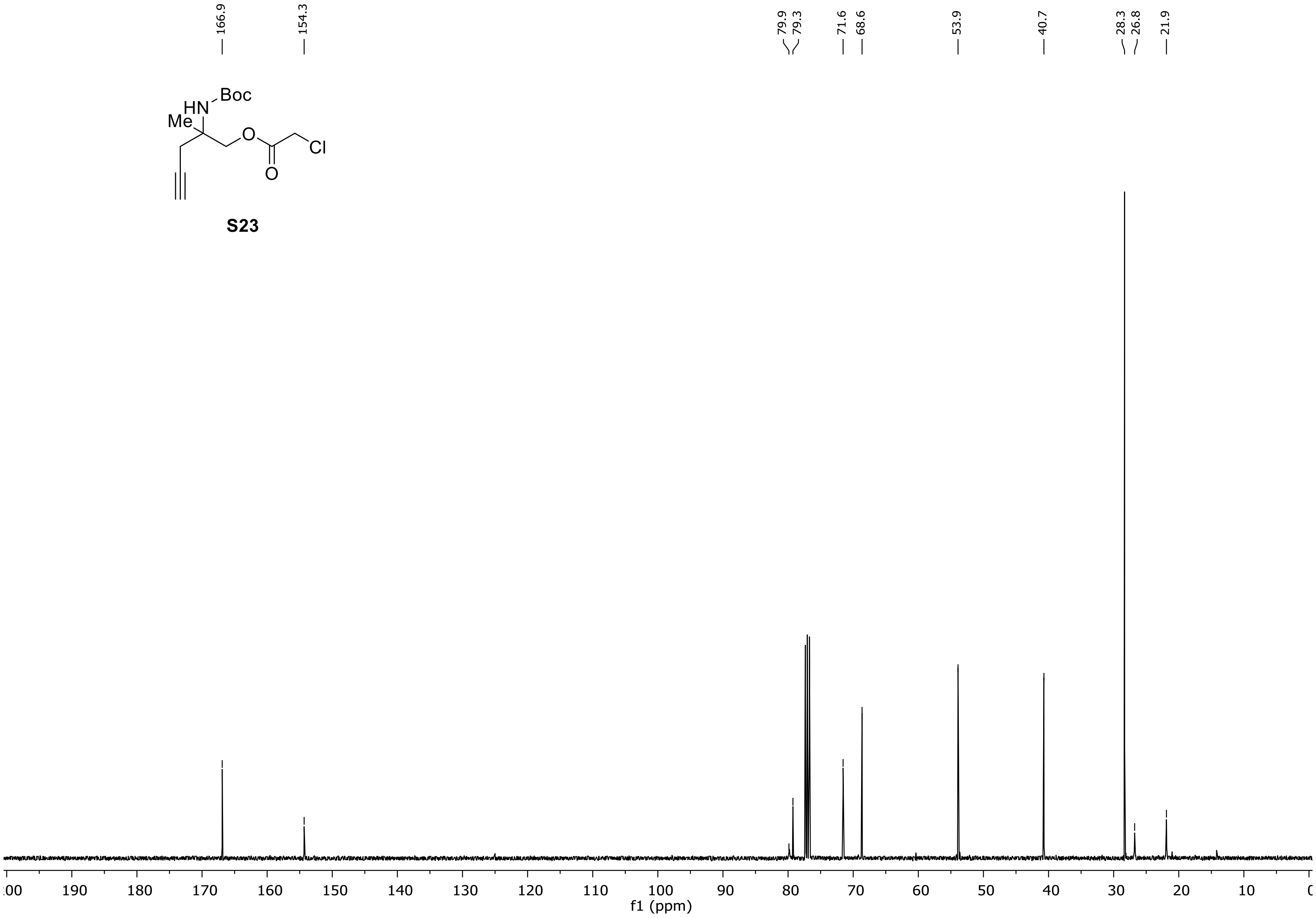
166.9
154.3
79.9
79.3
71.6
68.6
53.9
40.7
28.3
26.8
21.9
HN
Boc
Me
O
O
Cl
f1 (ppm)

S23

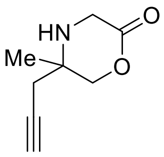
HN
O
Me
O

24

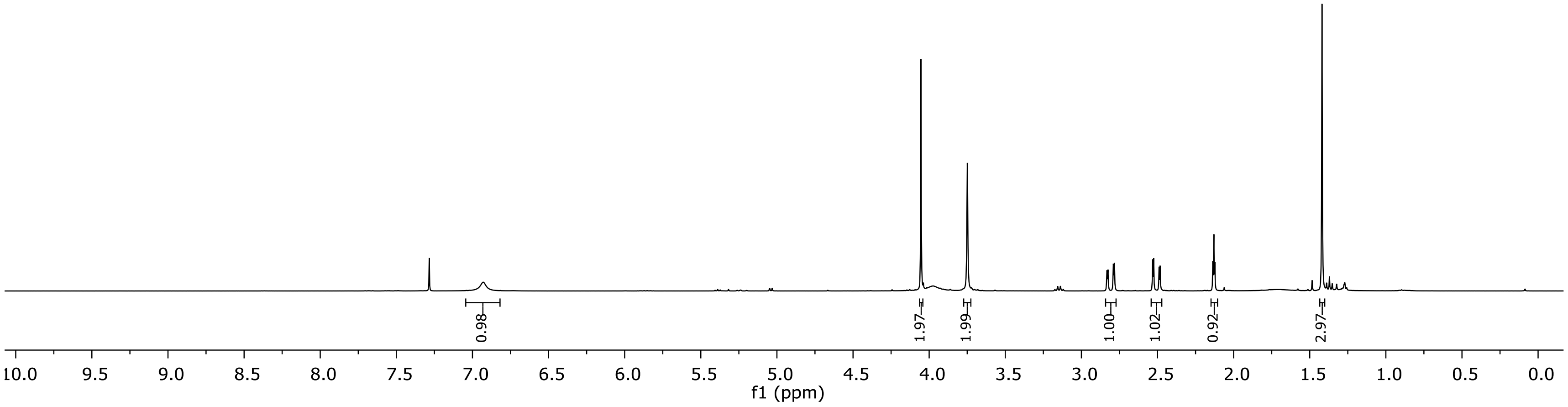
0.98
1.97
1.99
1.00
1.02
0.92
2.97
10.0 9.5 9.0 8.5 8.0 7.5 7.0 6.5 6.0 5.5 5.0 4.5 4.0 3.5 3.0 2.5 2.0 1.5 1.0 0.5 0.0
f1 (ppm)

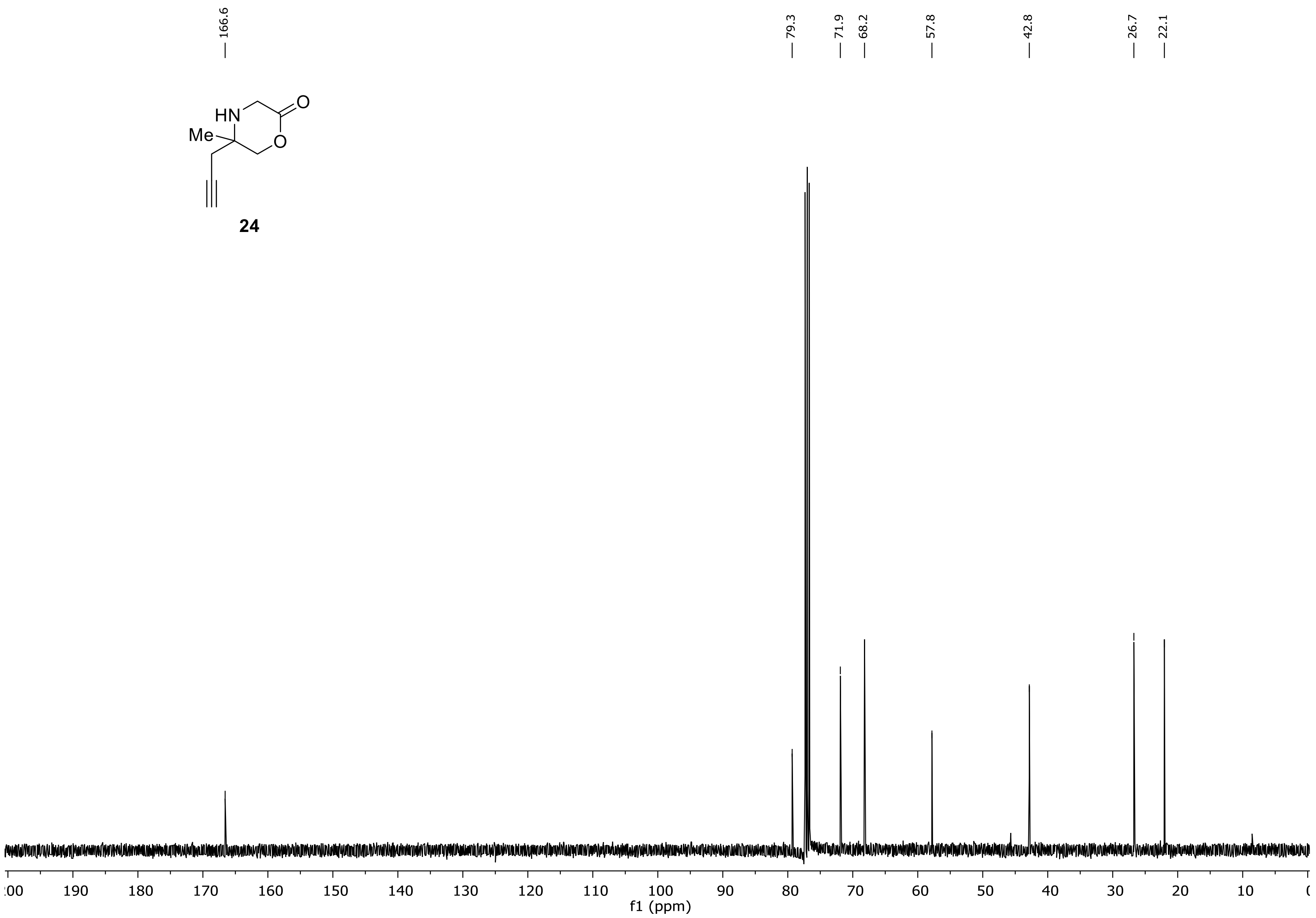
166.6
79.3
71.9
68.2
57.8
42.8
26.7
22.1
HN
O
Me
O
24
190
180
170
160
150
140
130
120
110
100
90
80
70
60
50
40
30
20
10
f1 (ppm)

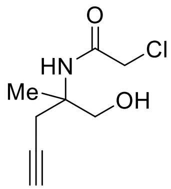
O
Cl
HN
Me
OH

S24

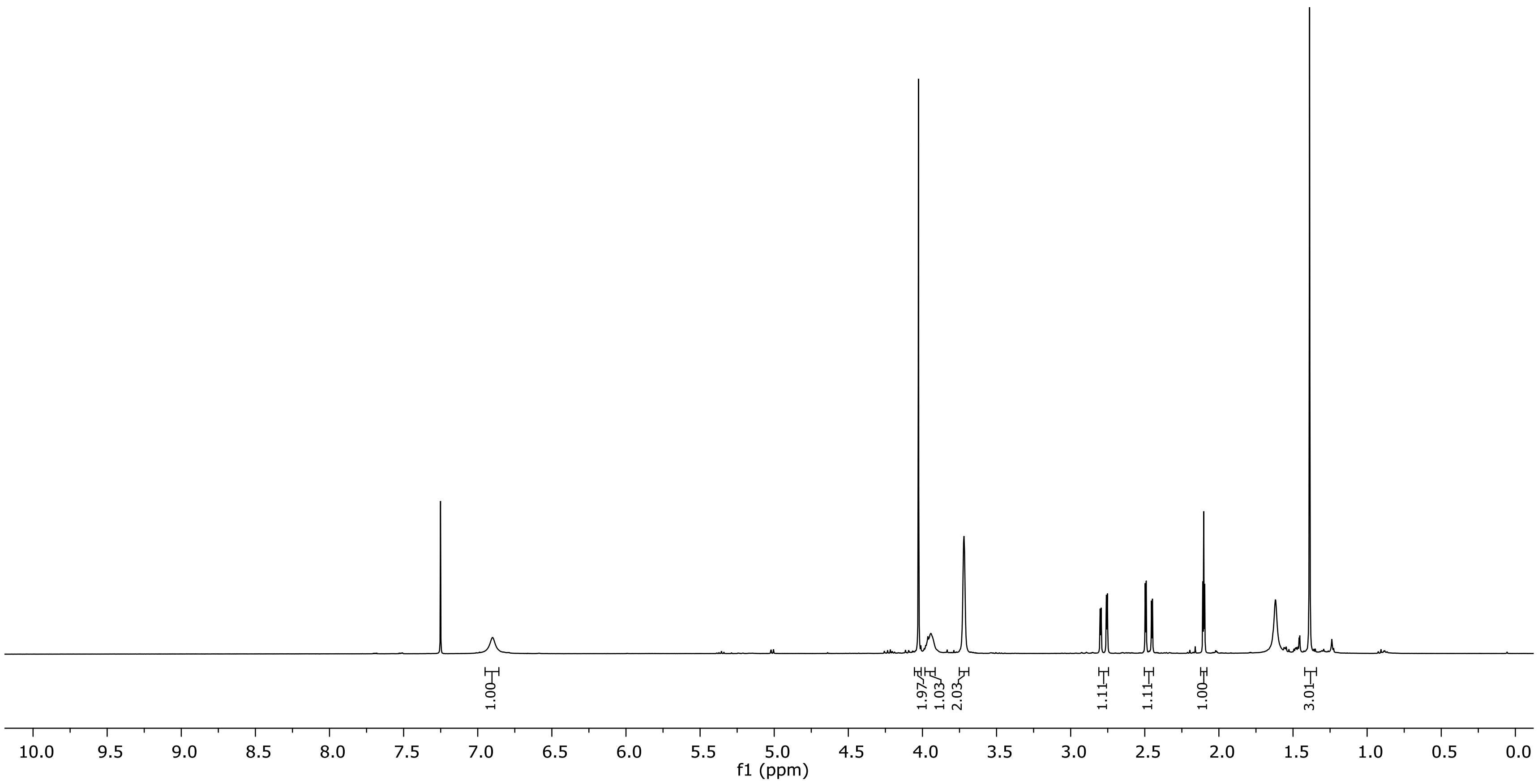
1.00
1.97
1.03
2.03
1.11
1.11
1.00
3.01
10.0
9.5
9.0
8.5
8.0
7.5
7.0
6.5
6.0
5.5
5.0
4.5
4.0
3.5
3.0
2.5
2.0
1.5
1.0
0.5
0.0
f1 (ppm)

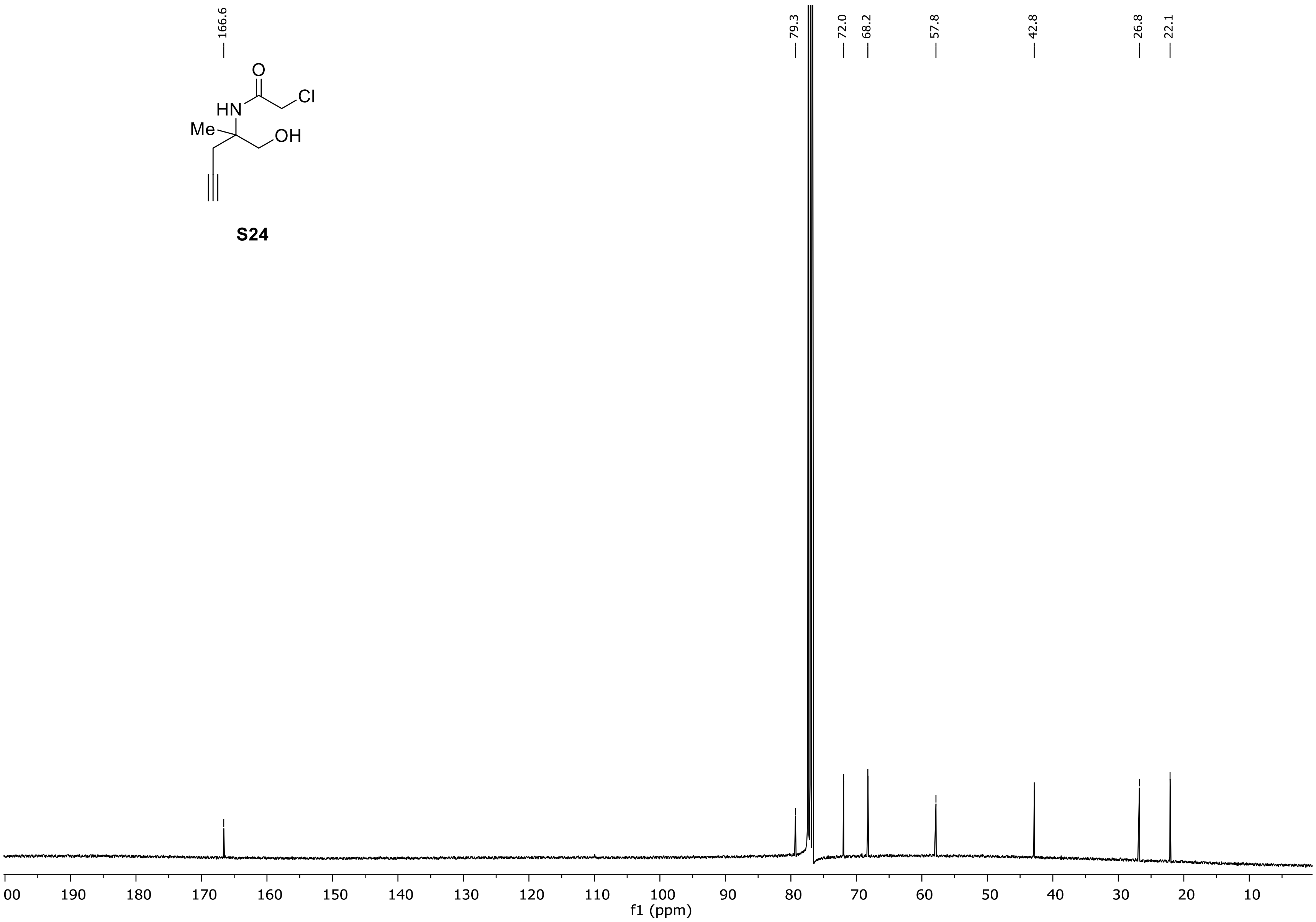
166.6
79.3
72.0
68.2
57.8
42.8
26.8
22.1
O
HN
Cl
Me
OH
00
190
180
170
160
150
140
130
120
110
100
90
80
70
60
50
40
30
20
10
f1 (ppm)

S24

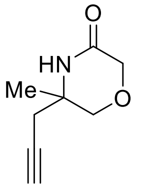
O
HN
Me
O

25

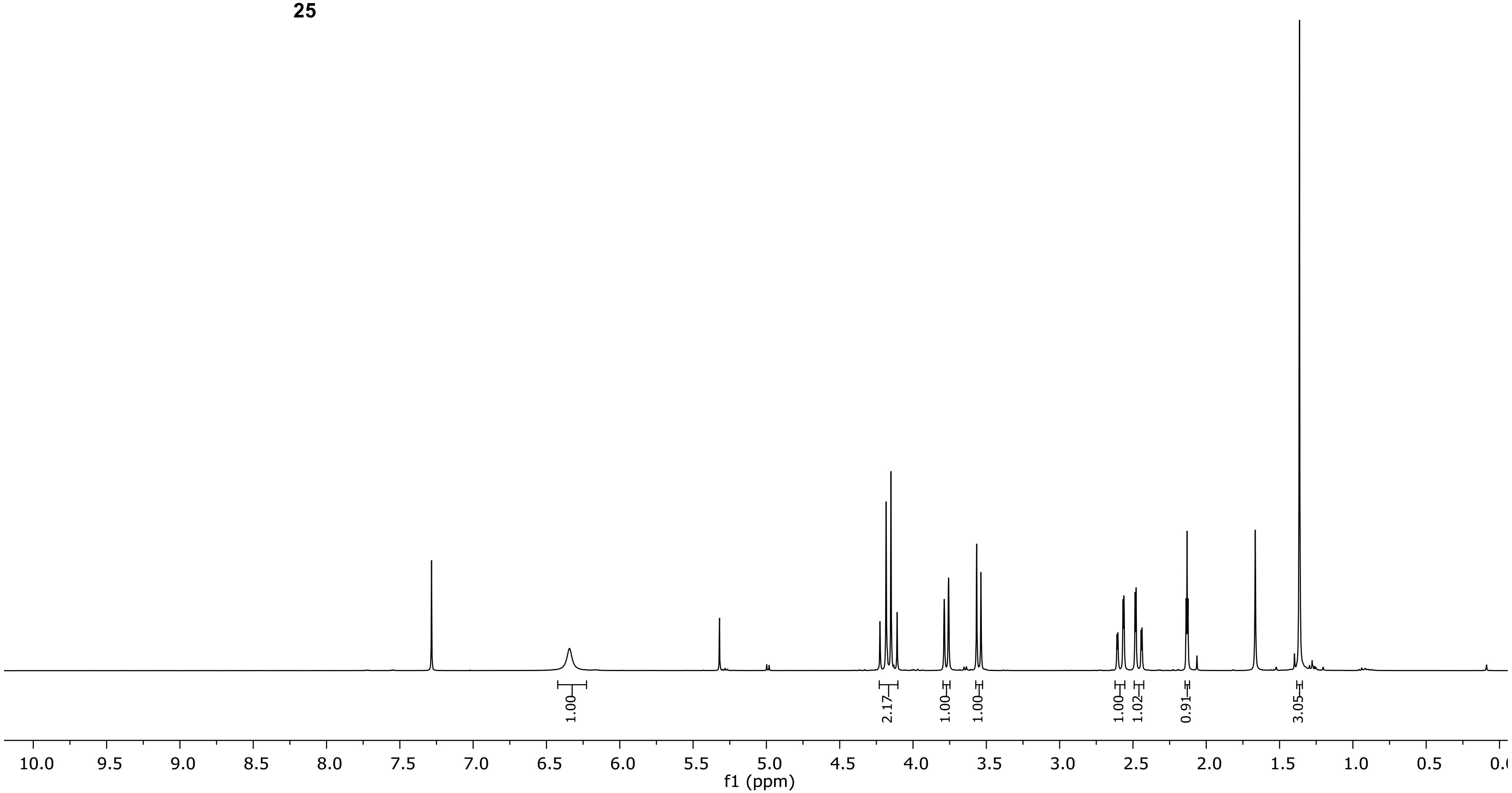
1.00
2.17
1.00
1.00
1.00
1.02
0.91
3.05
10.0 9.5 9.0 8.5 8.0 7.5 7.0 6.5 6.0 5.5 5.0 4.5 4.0 3.5 3.0 2.5 2.0 1.5 1.0 0.5
f1 (ppm)

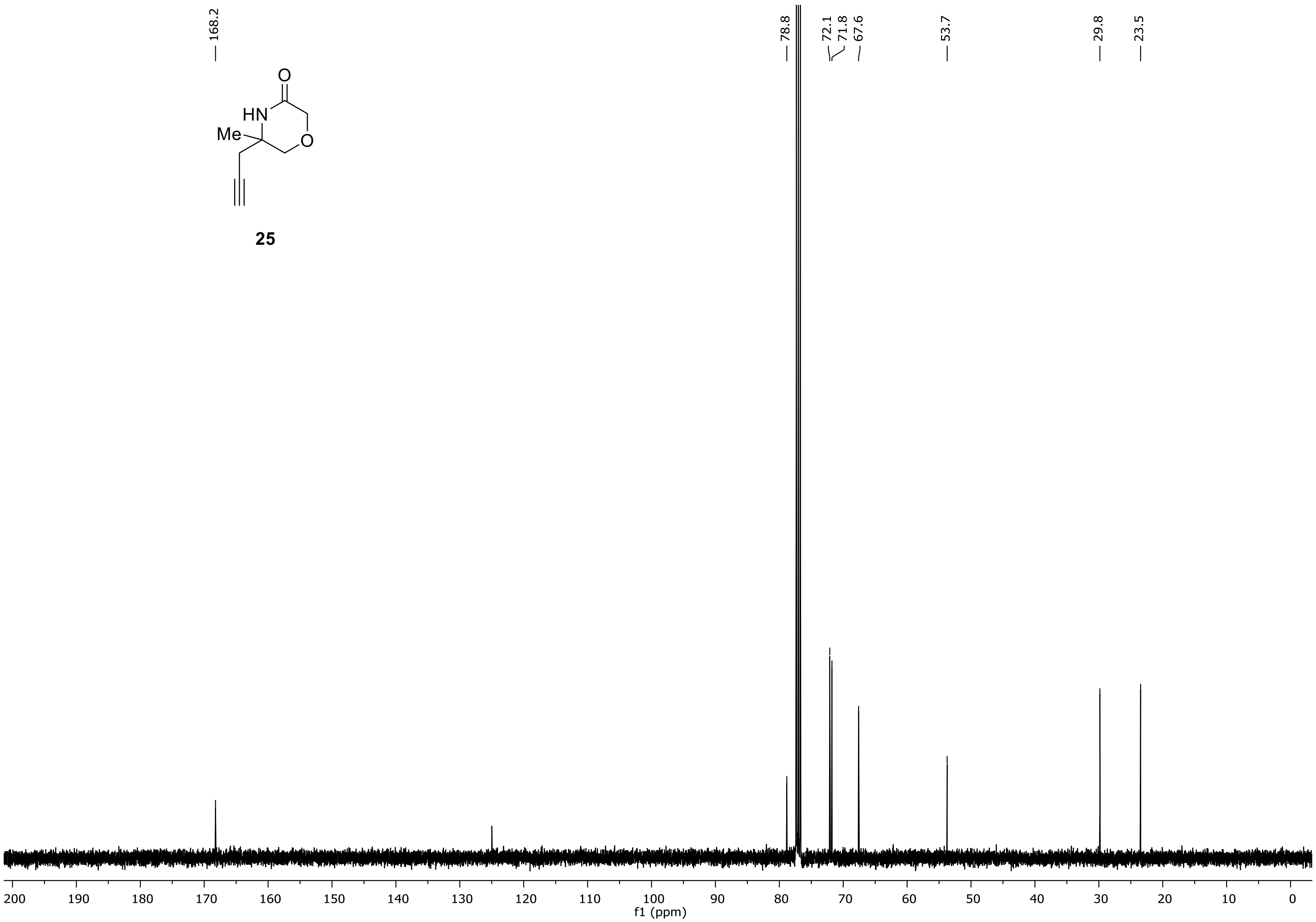
168.2
78.8
72.1
71.8
67.6
53.7
29.8
23.5
O
HN
Me
O
25
200
190
180
170
160
150
140
130
120
110
100
90
80
70
60
50
40
30
20
10
0
f1 (ppm)

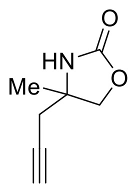
O
HN
O
Me

26

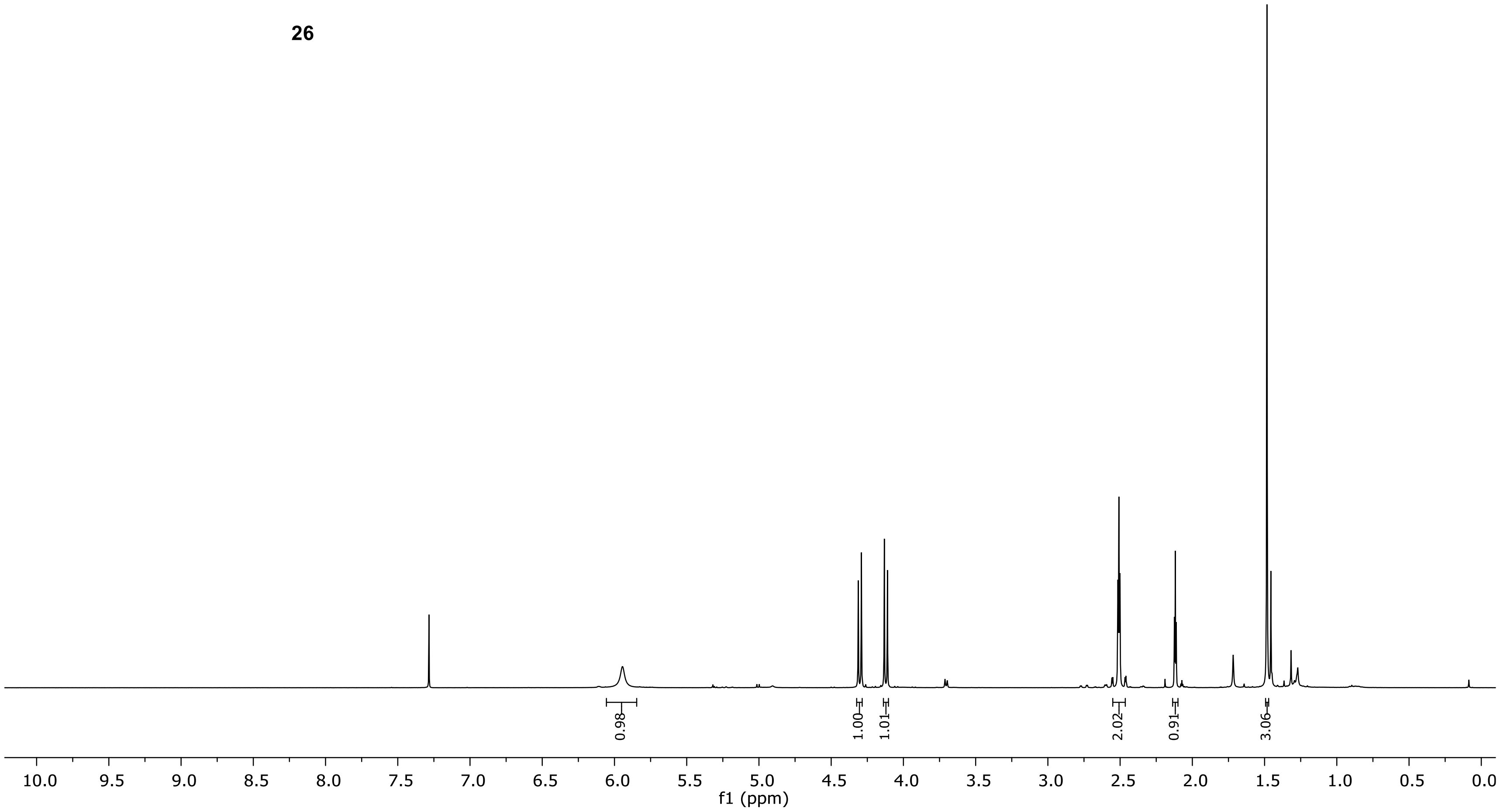
0.98
1.00
1.01
2.02
0.91
3.06
10.0 9.5 9.0 8.5 8.0 7.5 7.0 6.5 6.0 5.5 5.0 4.5 4.0 3.5 3.0 2.5 2.0 1.5 1.0 0.5 0.0
f1 (ppm)

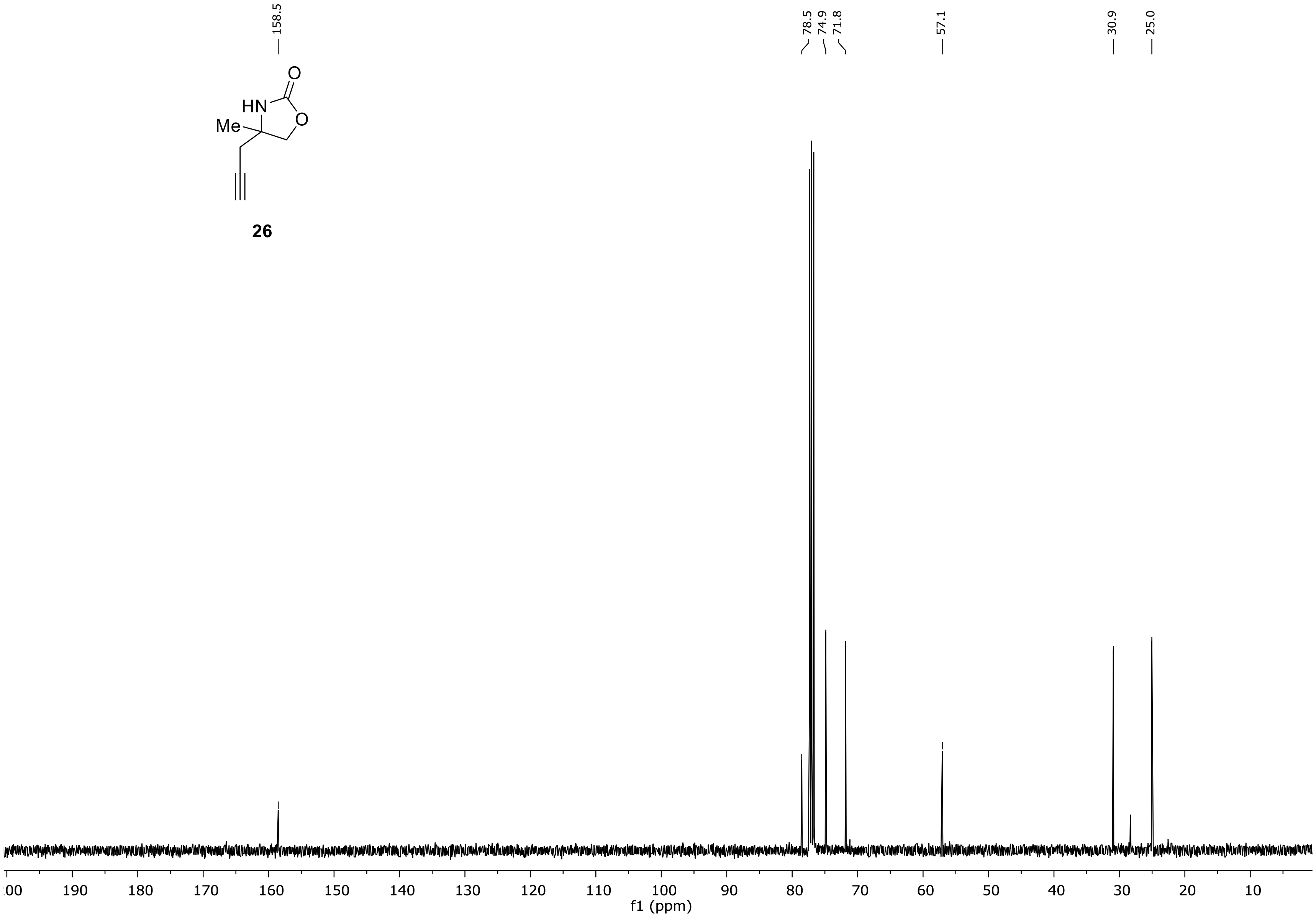
158.5
78.5
74.9
71.8
57.1
30.9
25.0
O
HN
O
Me
26
200 190 180 170 160 150 140 130 120 110 100 90 80 70 60 50 40 30 20 10
f1 (ppm)

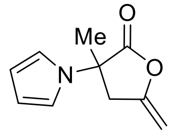

Me
O
N
O

27

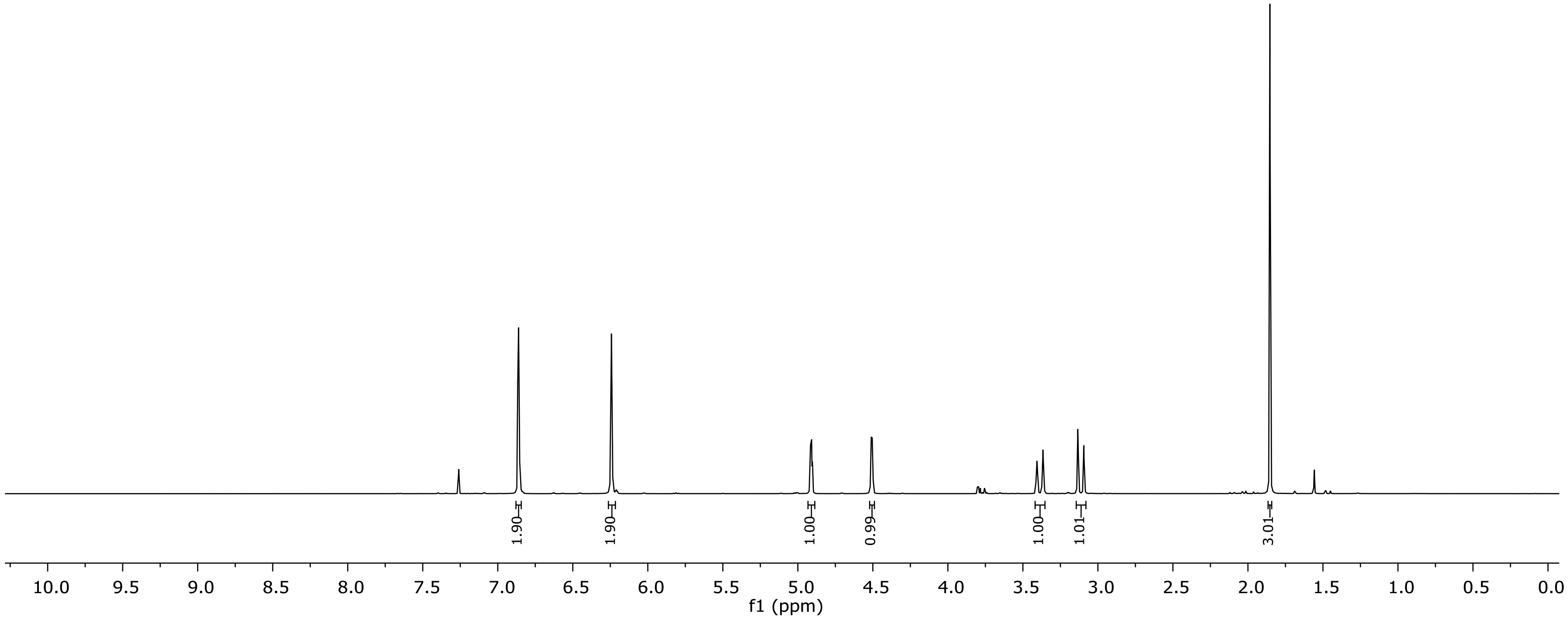

1.90
1.90
1.00
0.99
1.00
1.01
3.01
10.0
9.5
9.0
8.5
8.0
7.5
7.0
6.5
6.0
5.5
5.0
4.5
4.0
3.5
3.0
2.5
2.0
1.5
1.0
0.5
0.0
f1 (ppm)

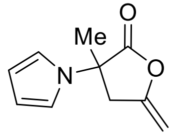

27

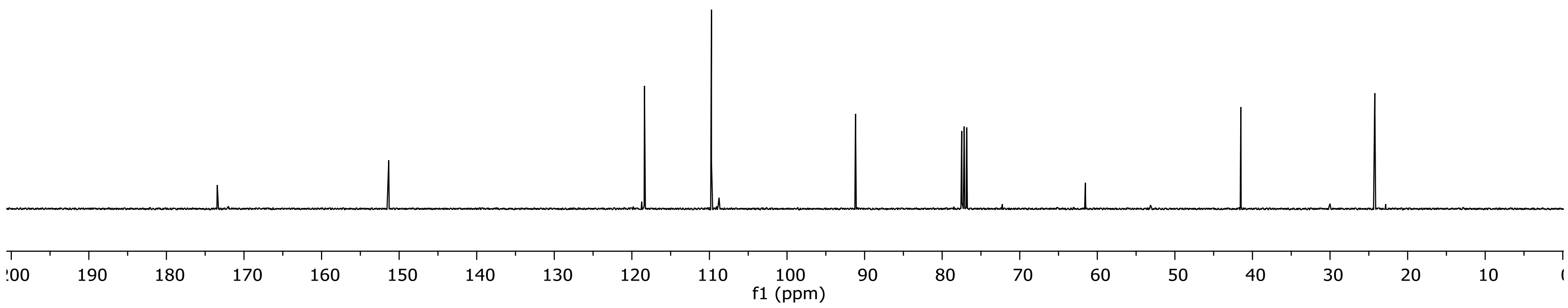

173.5
151.3
118.4
109.7
91.2
61.6
41.5
24.2
200
190
180
170
160
150
140
130
120
110
100
90
80
70
60
50
40
30
20
10
0
f1 (ppm)

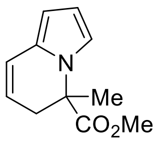
N
Me
CO2Me

28

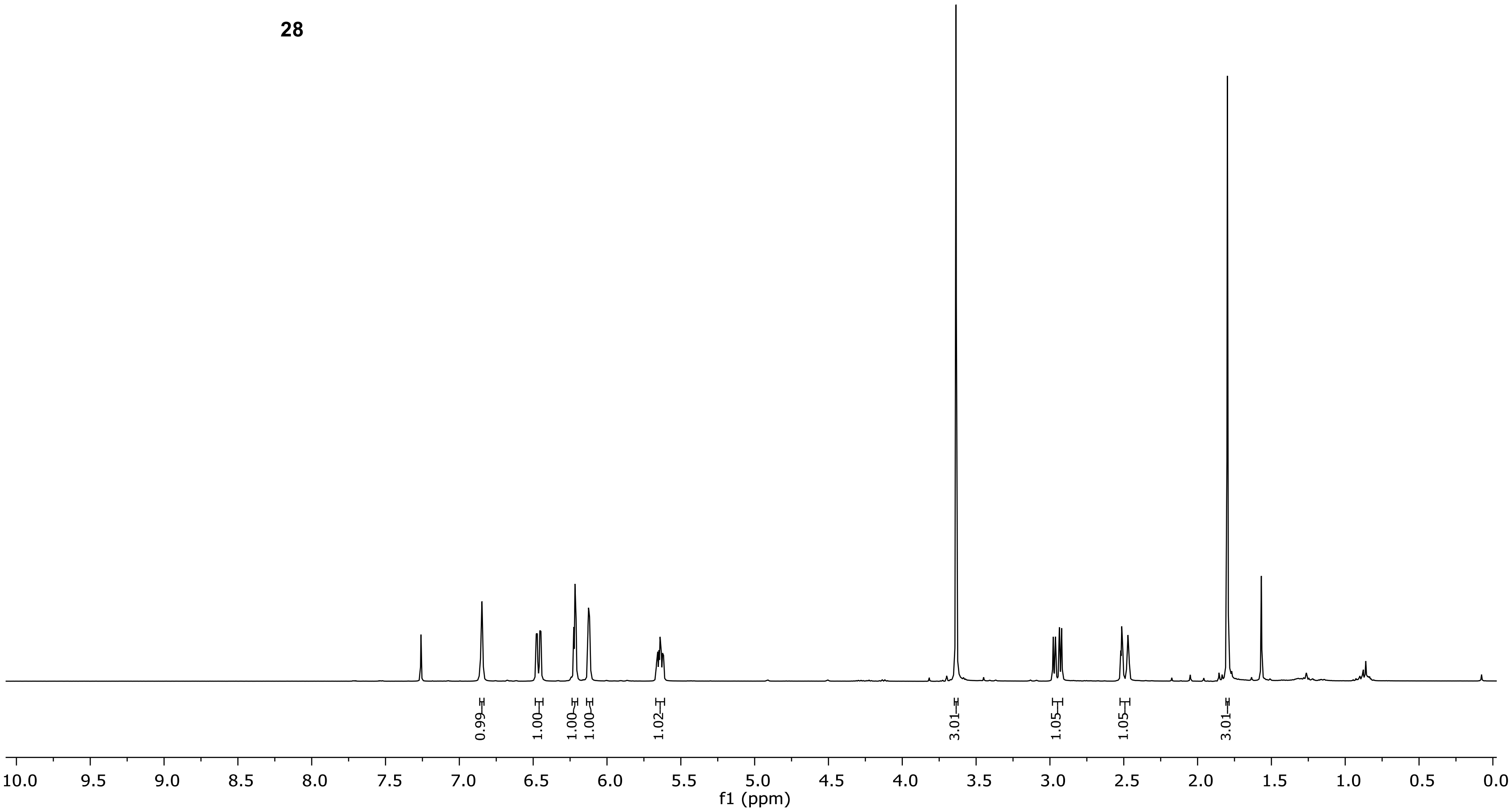
0.99
1.00
1.00
1.00
1.02
3.01
1.05
1.05
3.01
10.0 9.5 9.0 8.5 8.0 7.5 7.0 6.5 6.0 5.5 5.0 4.5 4.0 3.5 3.0 2.5 2.0 1.5 1.0 0.5 0.0
f1 (ppm)

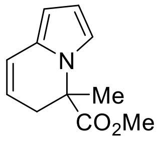
N
Me
CO_2Me

28

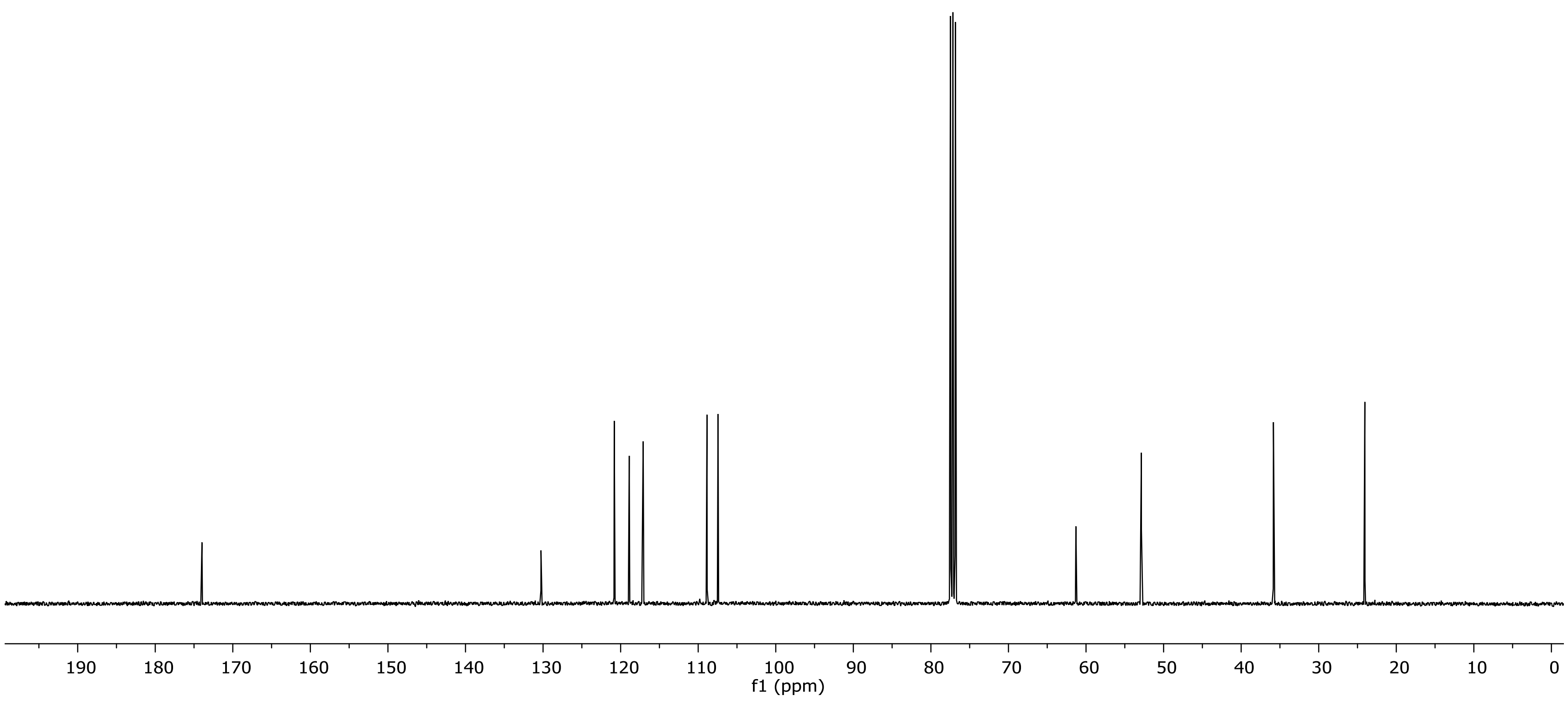
174.0
130.3
120.8
118.9
117.1
108.9
107.4
61.3
52.9
35.8
24.0
190 180 170 160 150 140 130 120 110 100 90 80 70 60 50 40 30 20 10 0
f1 (ppm)

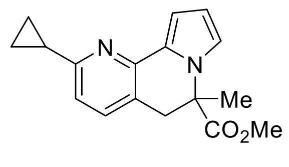
N
N
Me
CO2Me

29

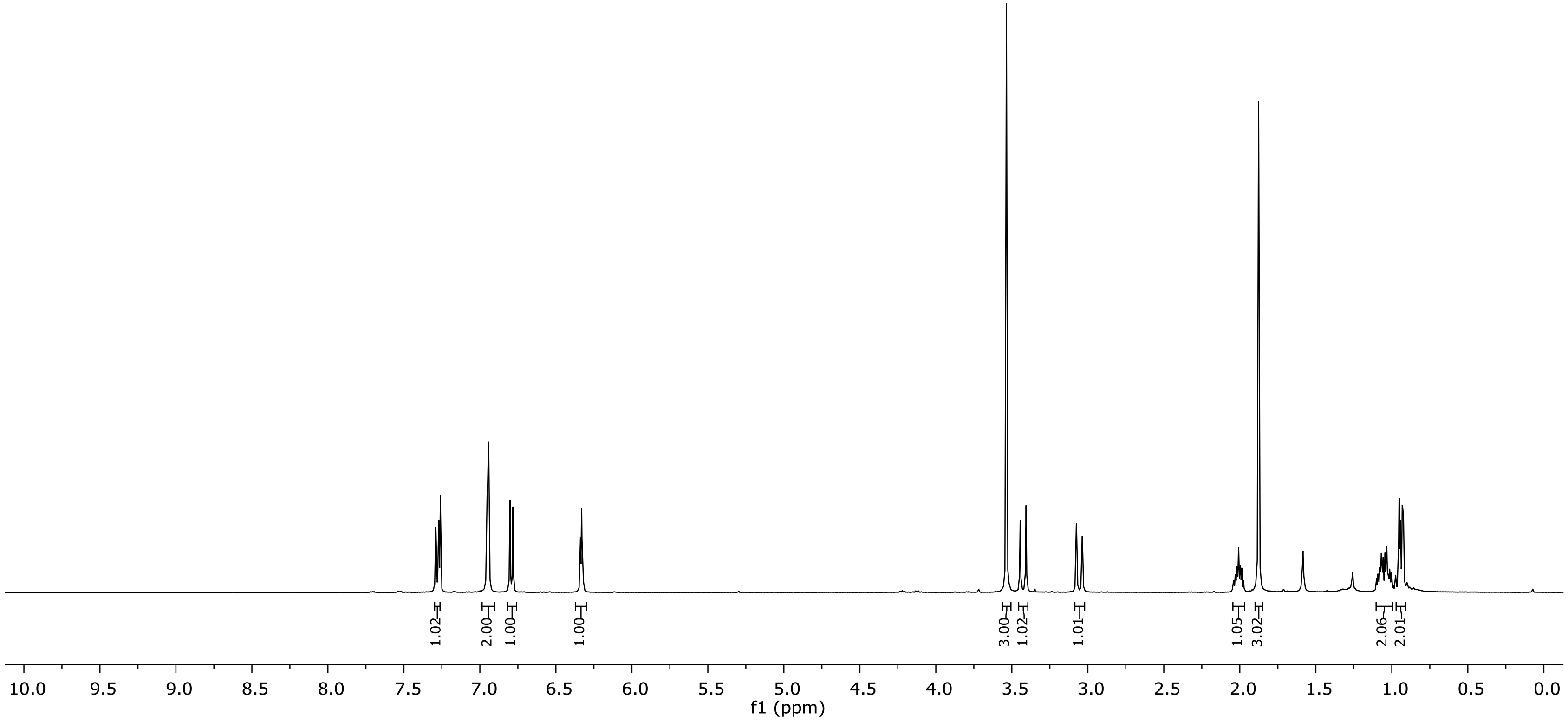
1.02
2.00
1.00
1.00
3.00
1.02
1.01
1.05
3.02
2.06
2.01
10.0 9.5 9.0 8.5 8.0 7.5 7.0 6.5 6.0 5.5 5.0 4.5 4.0 3.5 3.0 2.5 2.0 1.5 1.0 0.5 0.0
f1 (ppm)

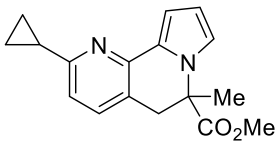

29

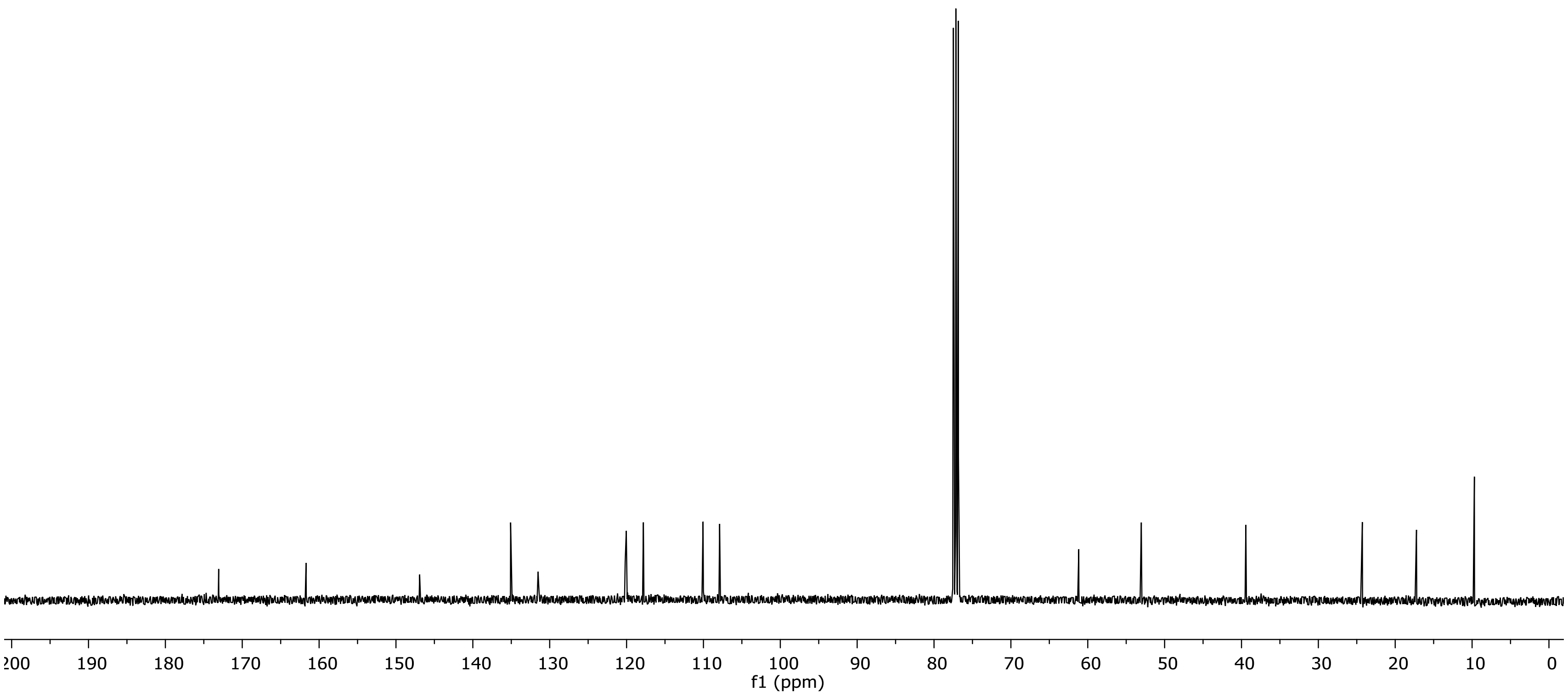
173.1
161.7
146.9
135.1
131.5
120.2
120.0
117.8
110.1
107.9
61.2
53.0
39.4
24.3
17.2
9.7
200 190 180 170 160 150 140 130 120 110 100 90 80 70 60 50 40 30 20 10 0
f1 (ppm)

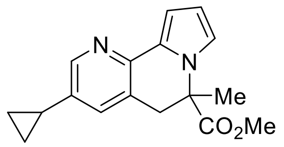
N
N
Me
CO2Me

30

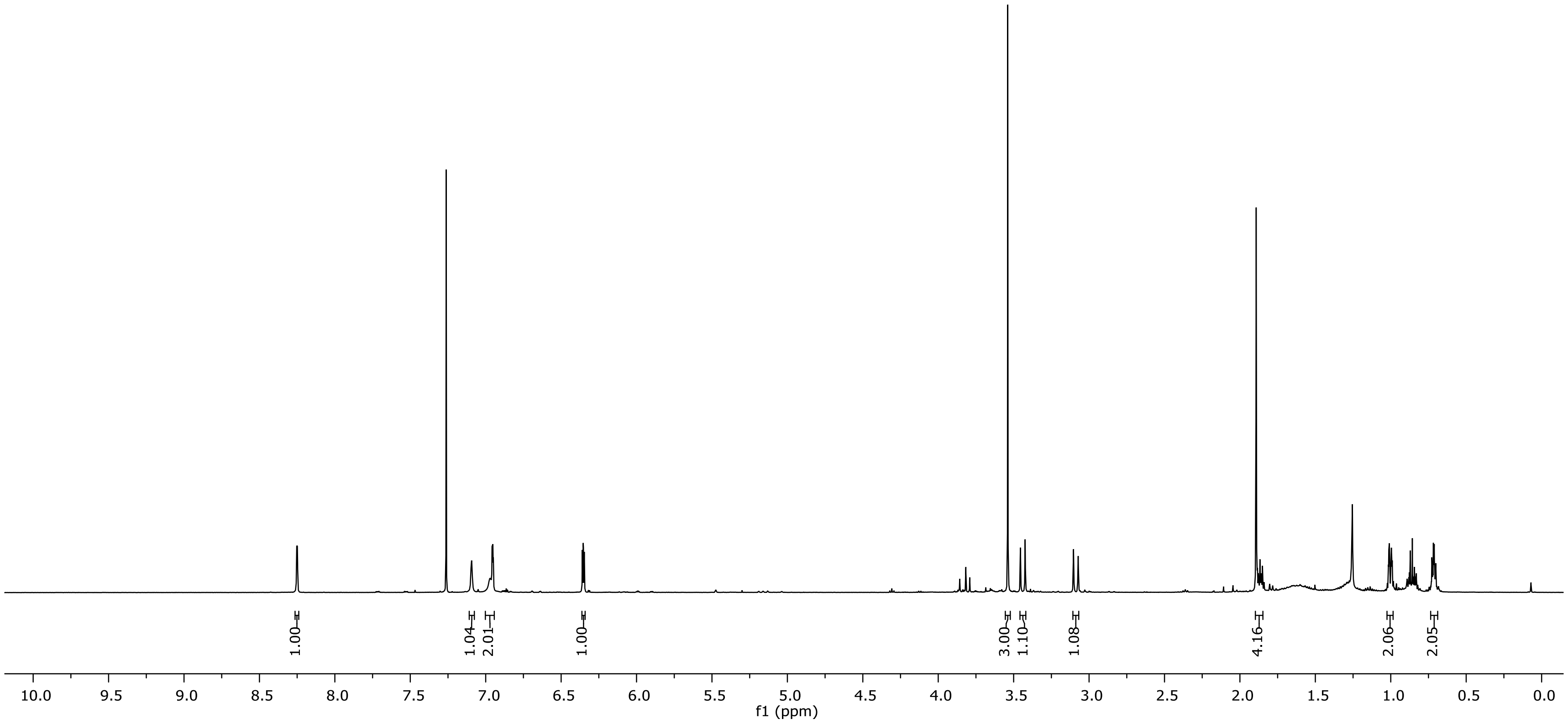
1.00
1.04
2.01
1.00
3.00
1.10
1.08
4.16
2.06
2.05
10.0
9.5
9.0
8.5
8.0
7.5
7.0
6.5
6.0
5.5
5.0
4.5
4.0
3.5
3.0
2.5
2.0
1.5
1.0
0.5
0.0
f1 (ppm)

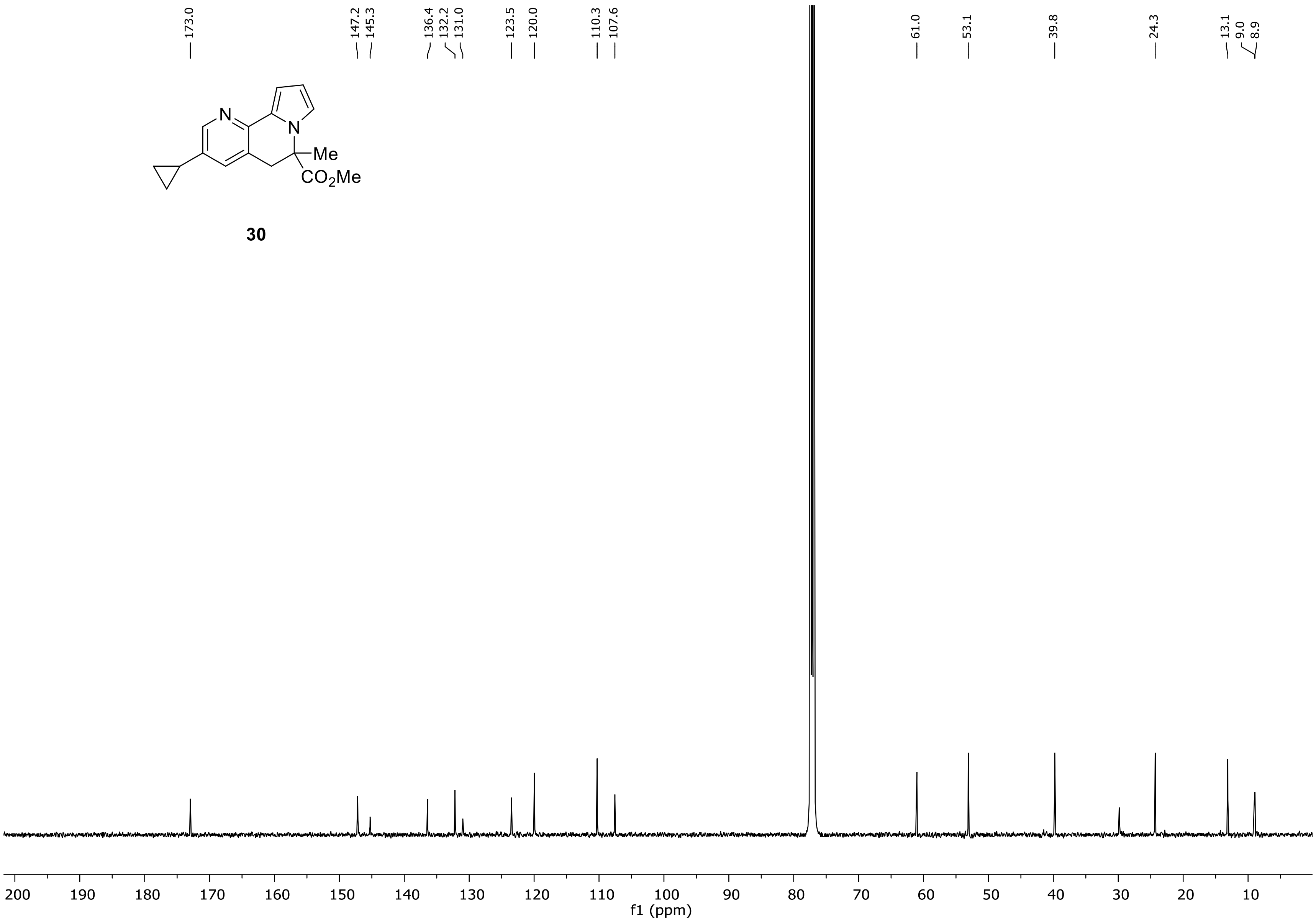

173.0
147.2
145.3
136.4
132.2
131.0
123.5
120.0
110.3
107.6
61.0
53.1
39.8
24.3
13.1
9.0
8.9
N
N
Me
CO2Me
200 190 180 170 160 150 140 130 120 110 100 90 80 70 60 50 40 30 20 10
f1 (ppm)

30

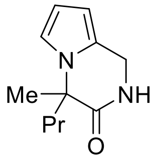
N
NH
Me
Pr
O

31

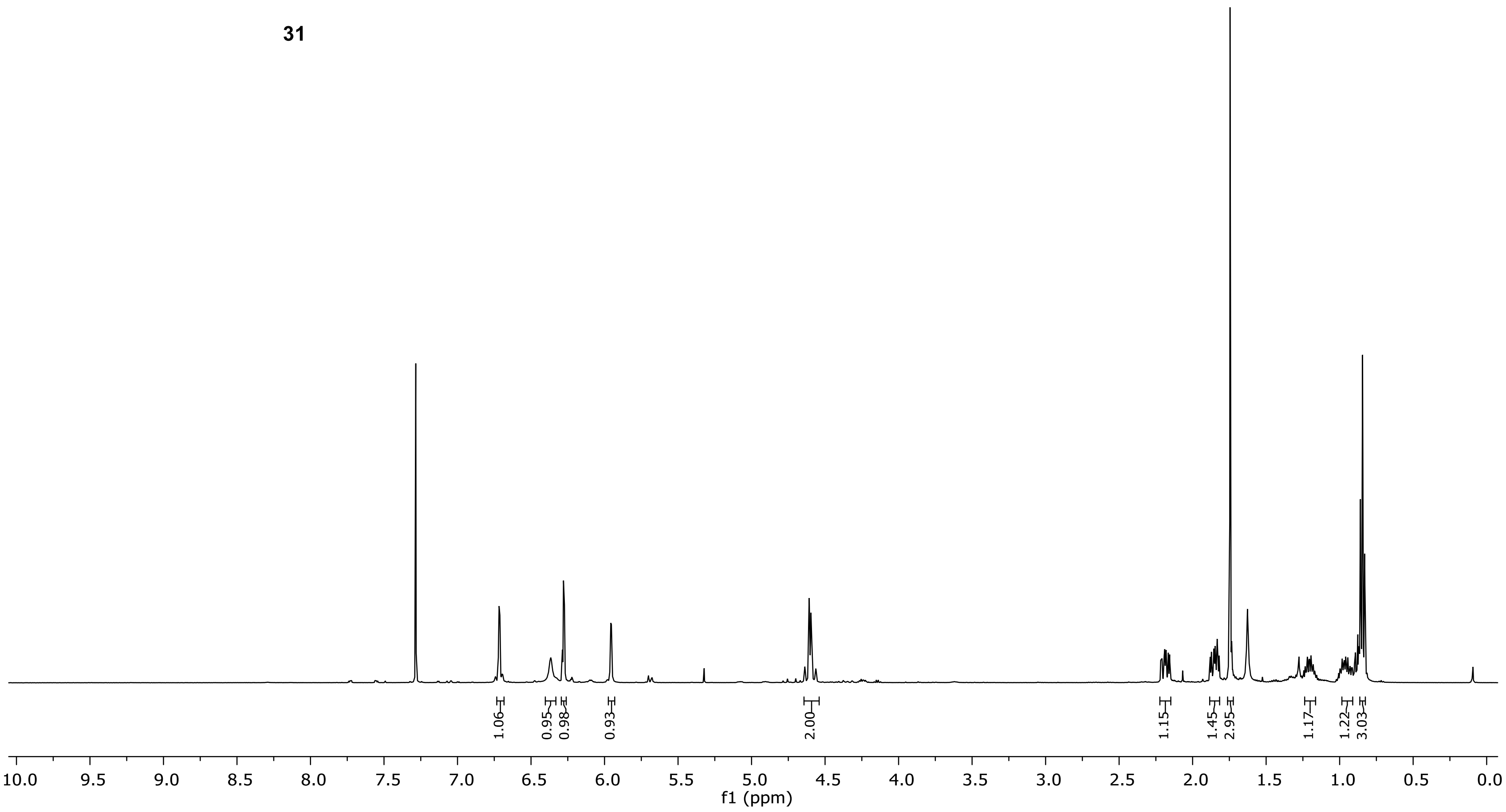
1.06
0.95
0.98
0.93
2.00
1.15
1.45
2.95
1.17
1.22
3.03
10.0
9.5
9.0
8.5
8.0
7.5
7.0
6.5
6.0
5.5
5.0
4.5
4.0
3.5
3.0
2.5
2.0
1.5
1.0
0.5
0.0
f1 (ppm)

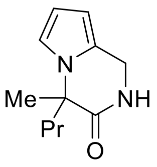
N
NH
Me
Pr
O

31

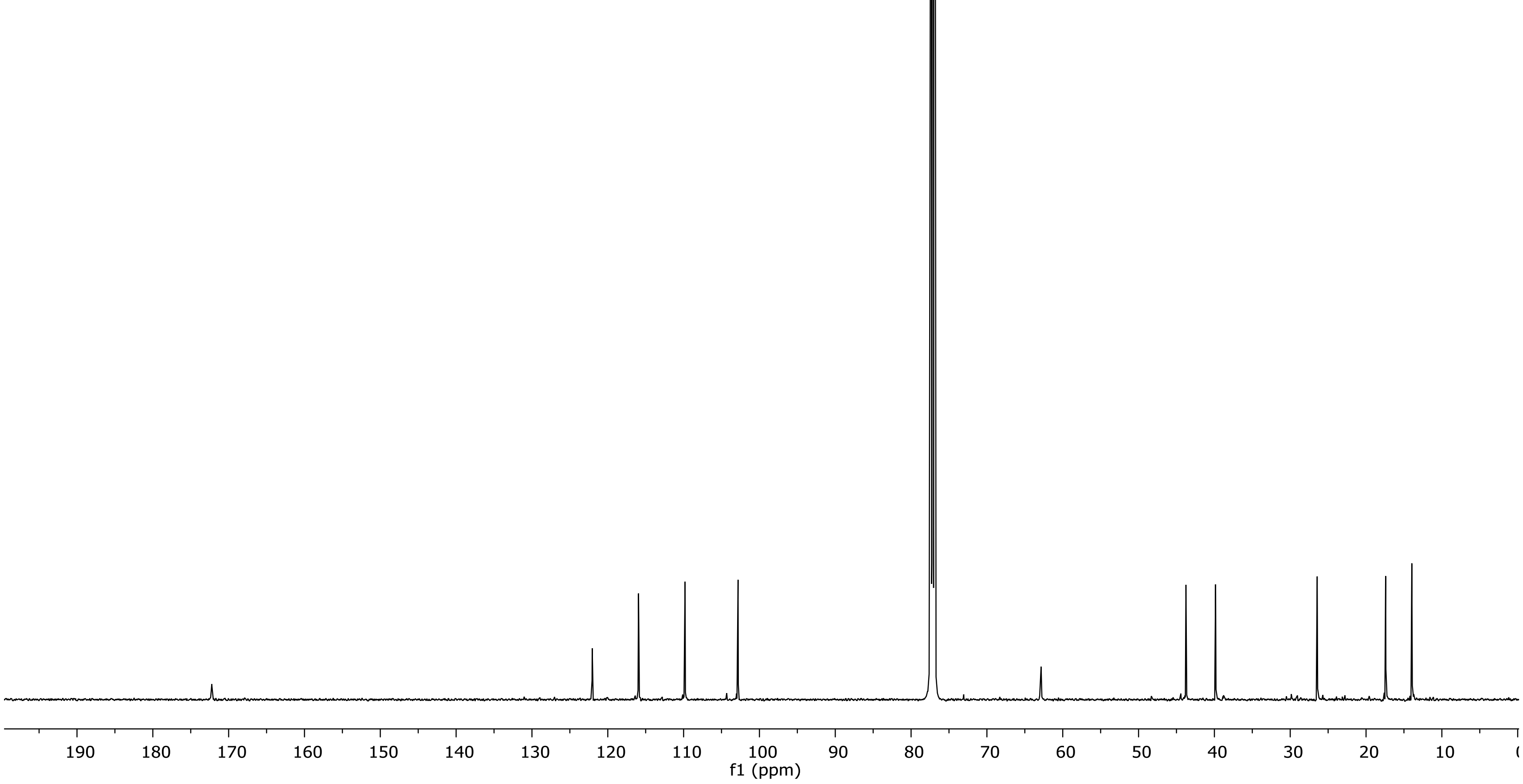
172.2
122.1
115.9
109.8
102.8
62.9
43.7
39.8
26.4
17.4
14.0
190 180 170 160 150 140 130 120 110 100 90 80 70 60 50 40 30 20 10
f1 (ppm)

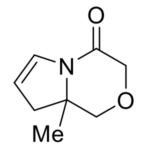
O
N
O
Me

32

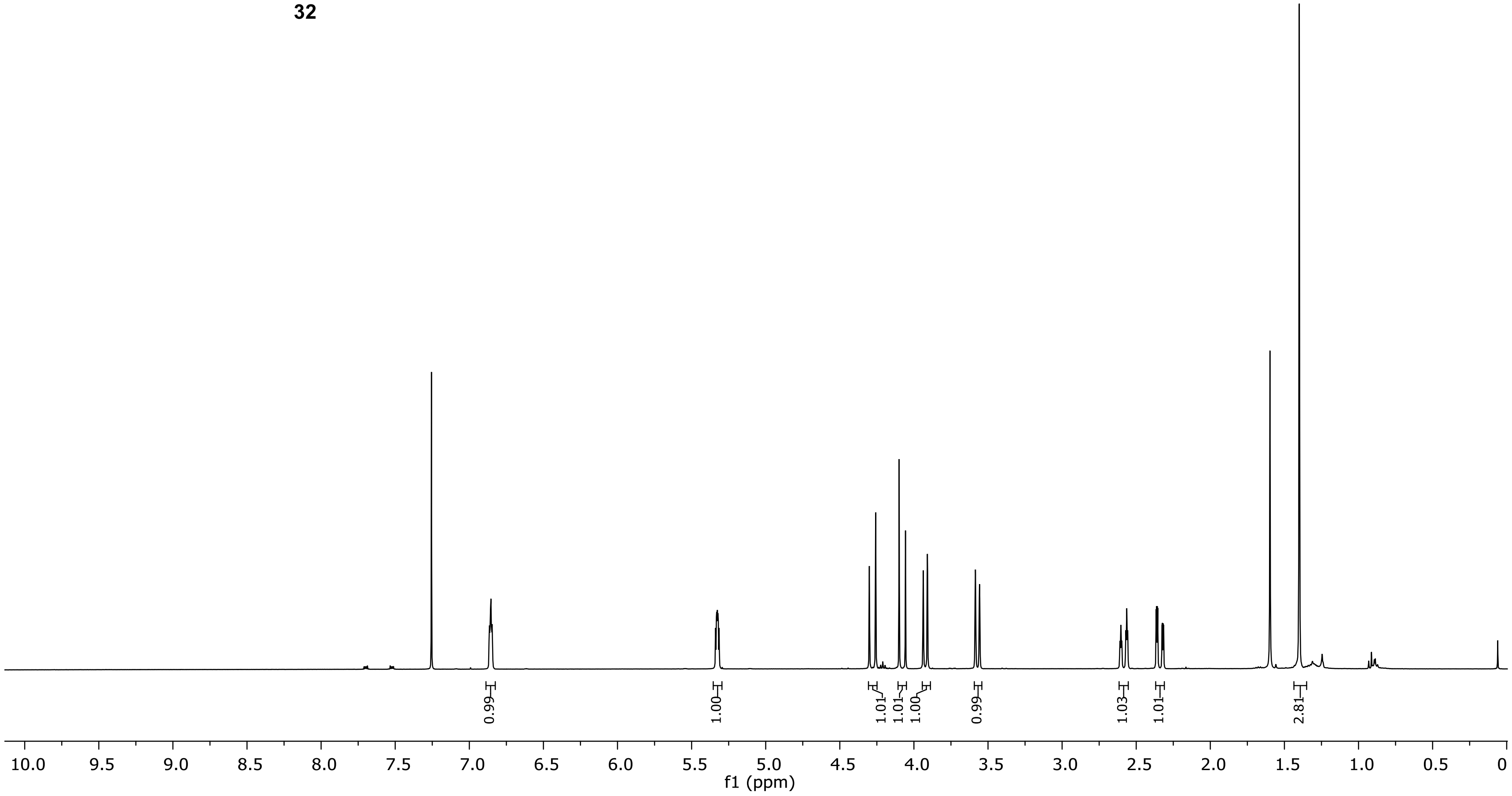
0.99
1.00
1.01
1.01
1.00
0.99
1.03
1.01
2.81
10.0
9.5
9.0
8.5
8.0
7.5
7.0
6.5
6.0
5.5
5.0
4.5
4.0
3.5
3.0
2.5
2.0
1.5
1.0
0.5
0
f1 (ppm)

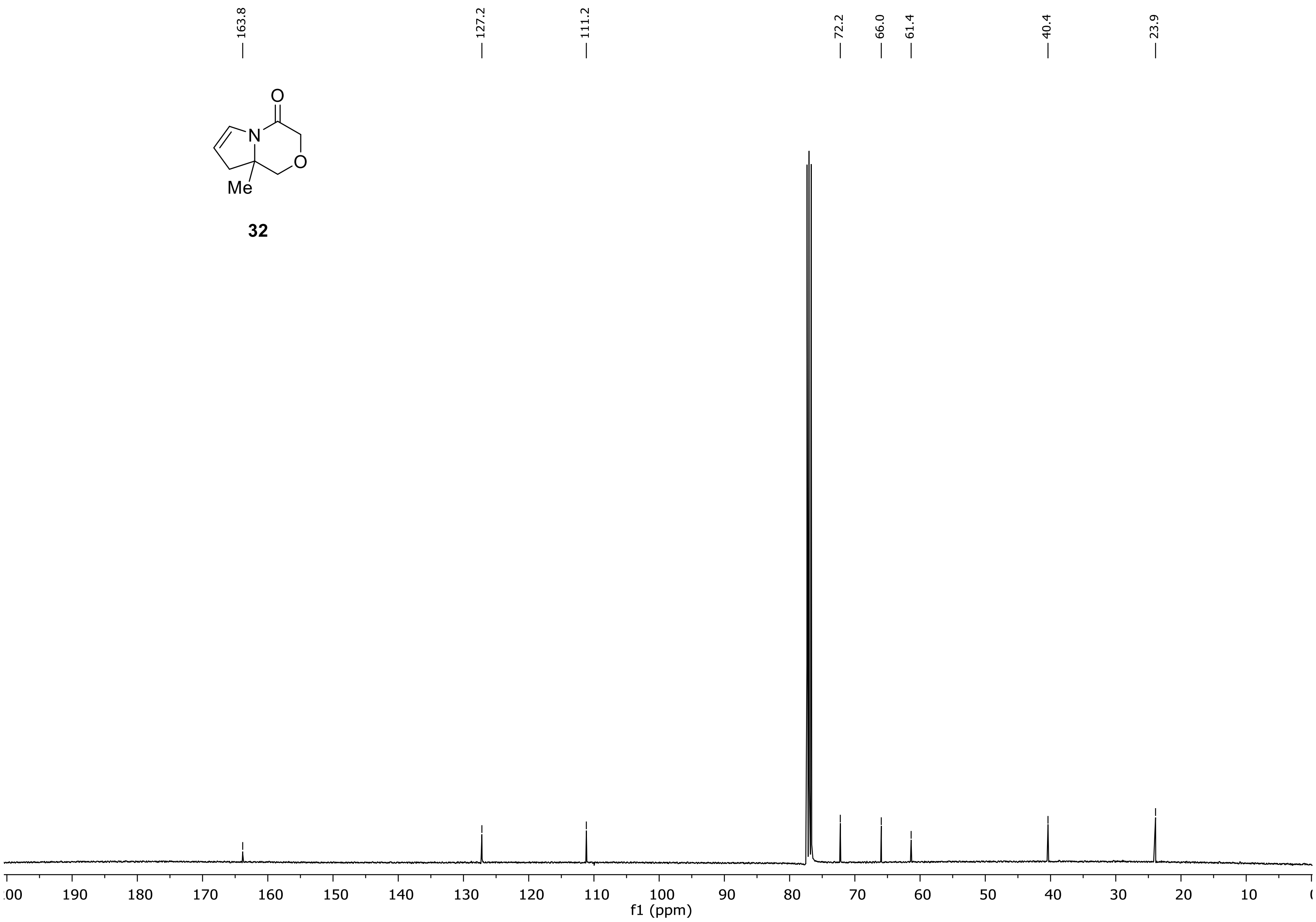
163.8
127.2
111.2
72.2
66.0
61.4
40.4
23.9
O
N
O
Me
32
190
180
170
160
150
140
130
120
110
100
90
80
70
60
50
40
30
20
10
f1 (ppm)

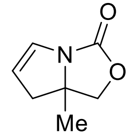
O
N
O
Me

33

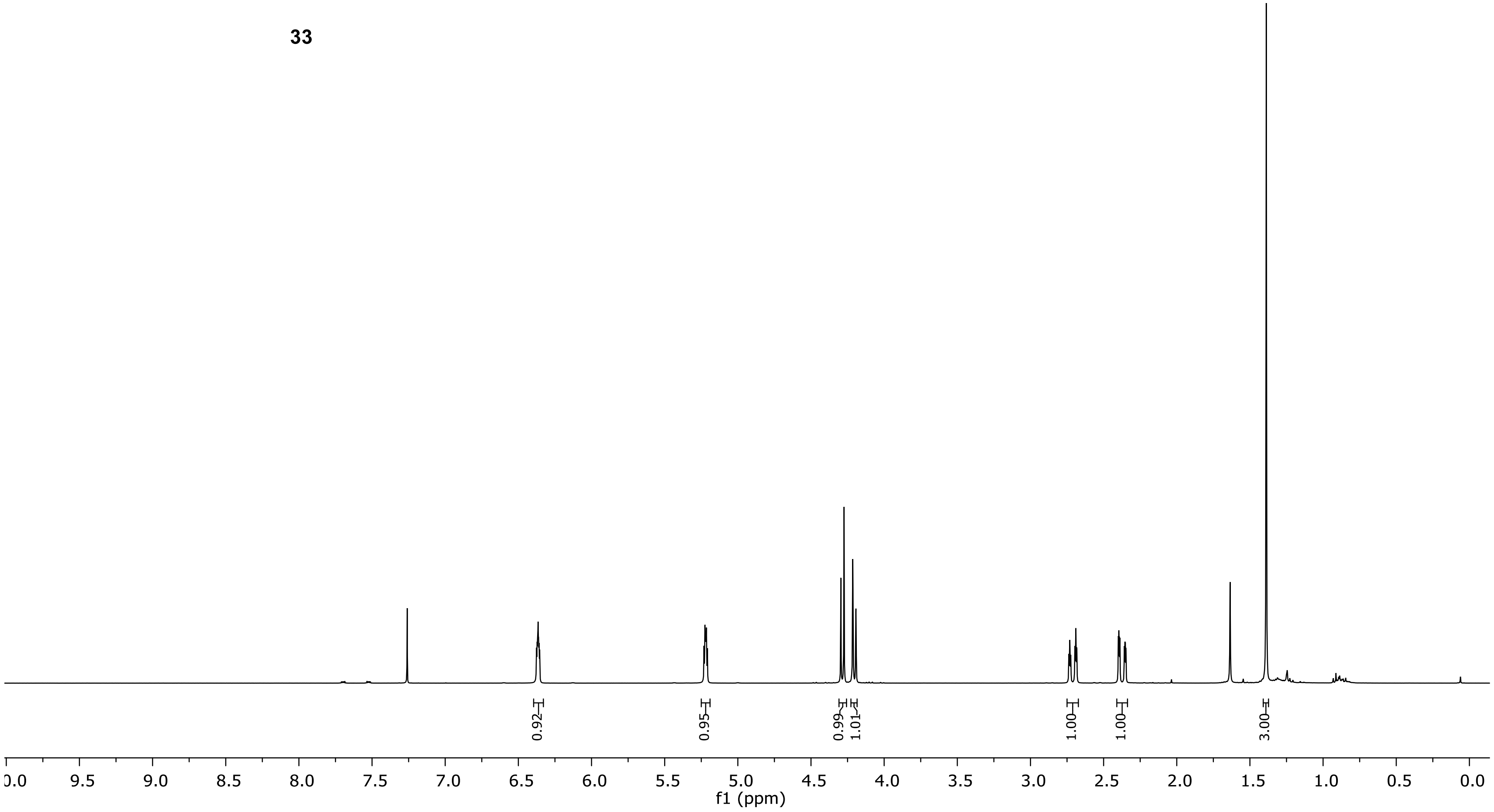
0.92
0.95
0.99
1.01
1.00
1.00
3.00
0.0 9.5 9.0 8.5 8.0 7.5 7.0 6.5 6.0 5.5 5.0 4.5 4.0 3.5 3.0 2.5 2.0 1.5 1.0 0.5 0.0
f1 (ppm)

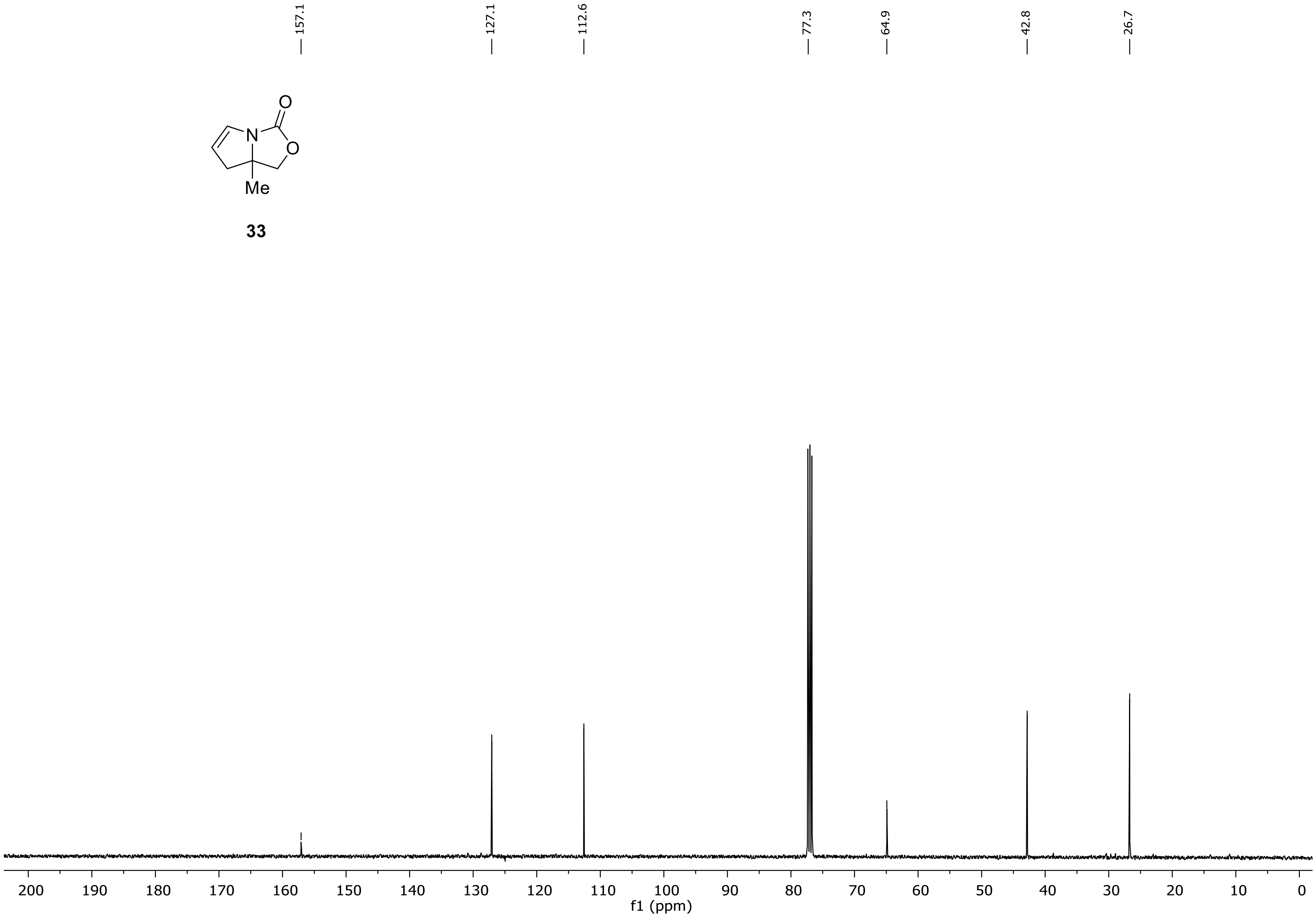
157.1
127.1
112.6
77.3
64.9
42.8
26.7
O
N
O
Me
33
200 190 180 170 160 150 140 130 120 110 100 90 80 70 60 50 40 30 20 10 0
f1 (ppm)

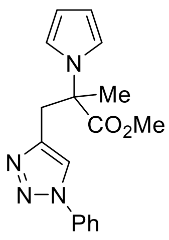
N
Me
CO2Me
N
N–N
Ph

19a

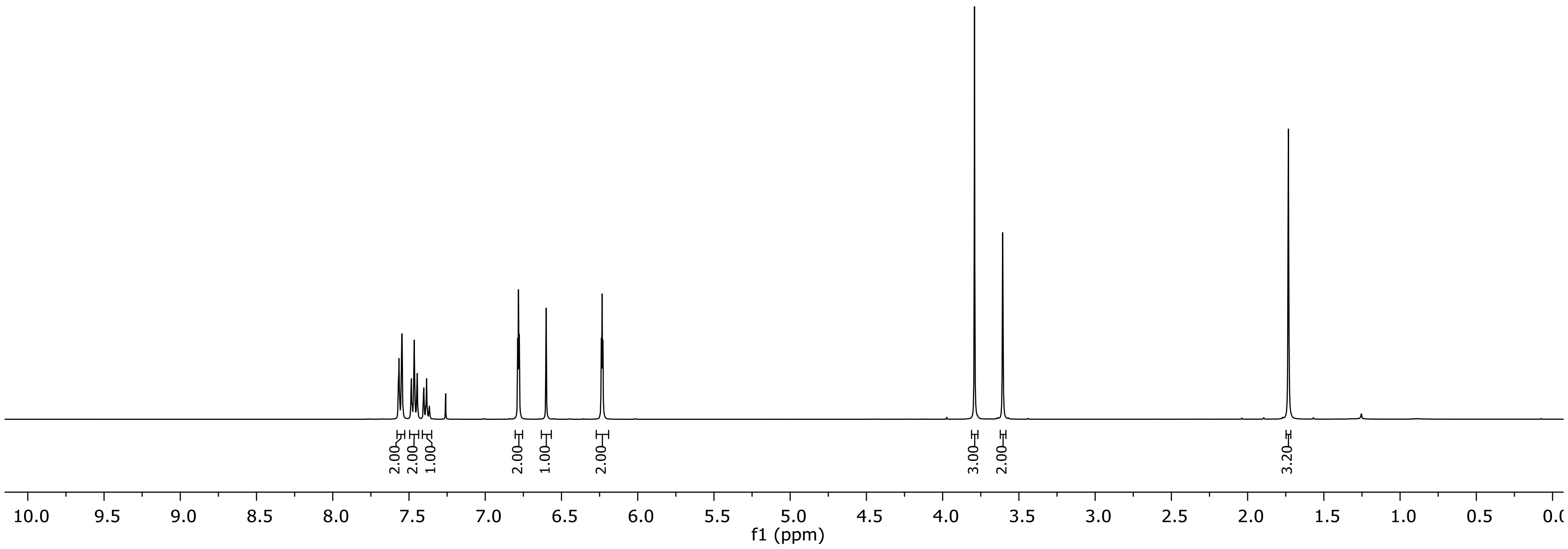
2.00
2.00
1.00
2.00
1.00
2.00
3.00
2.00
3.20
10.0
9.5
9.0
8.5
8.0
7.5
7.0
6.5
6.0
5.5
5.0
4.5
4.0
3.5
3.0
2.5
2.0
1.5
1.0
0.5
f1 (ppm)

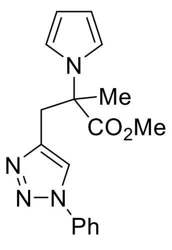

173.0
142.3
137.1
129.8
128.6
120.8
120.4
119.1
108.9
63.8
53.2
36.3
21.8
f1 (ppm)

19a

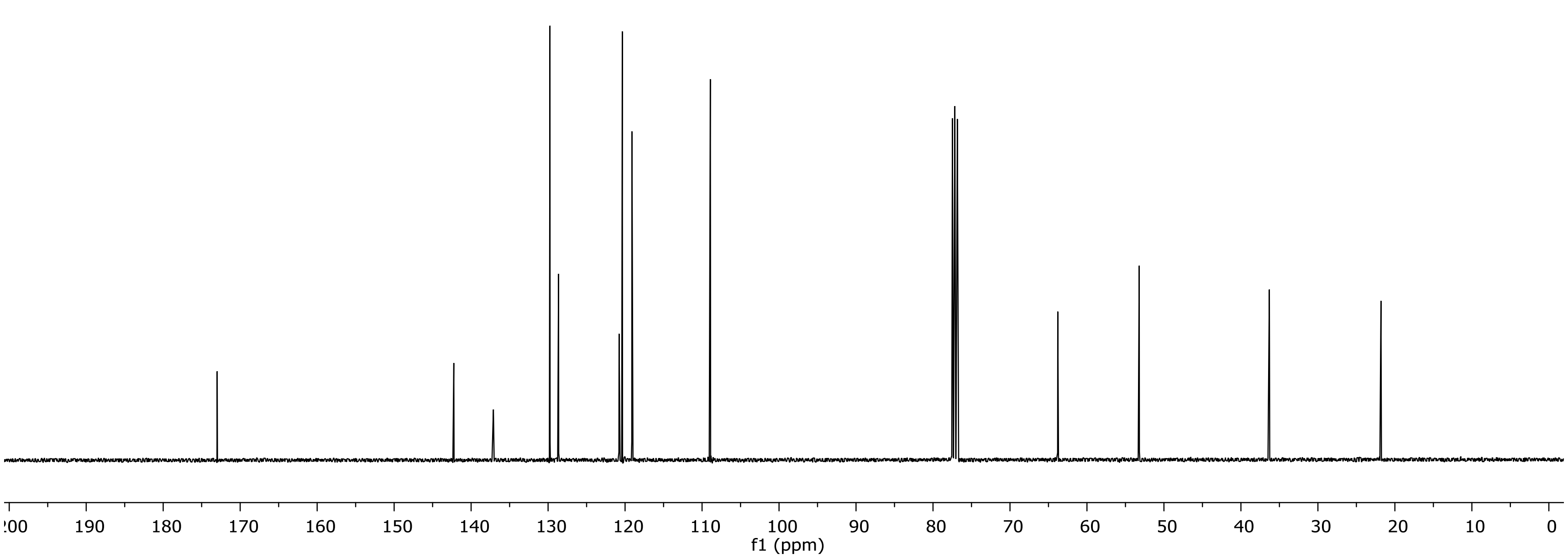

200
190
180
170
160
150
140
130
120
110
100
90
80
70
60
50
40
30
20
10
0
f1 (ppm)

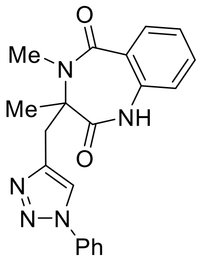
O
Me
N
Me
NH
O
N
N
N
Ph

21a

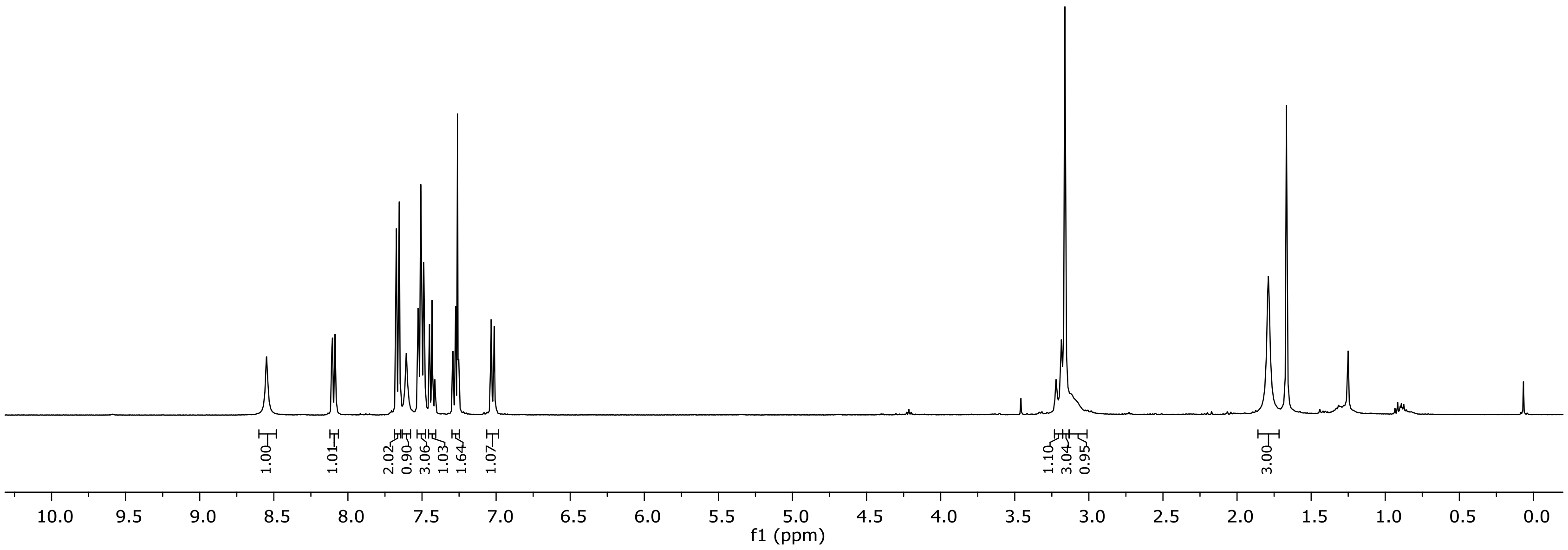
1.00
1.01
2.02
0.90
3.06
1.03
1.64
1.07
1.10
3.04
0.95
3.00
10.0
9.5
9.0
8.5
8.0
7.5
7.0
6.5
6.0
5.5
5.0
4.5
4.0
3.5
3.0
2.5
2.0
1.5
1.0
0.5
0.0
f1 (ppm)

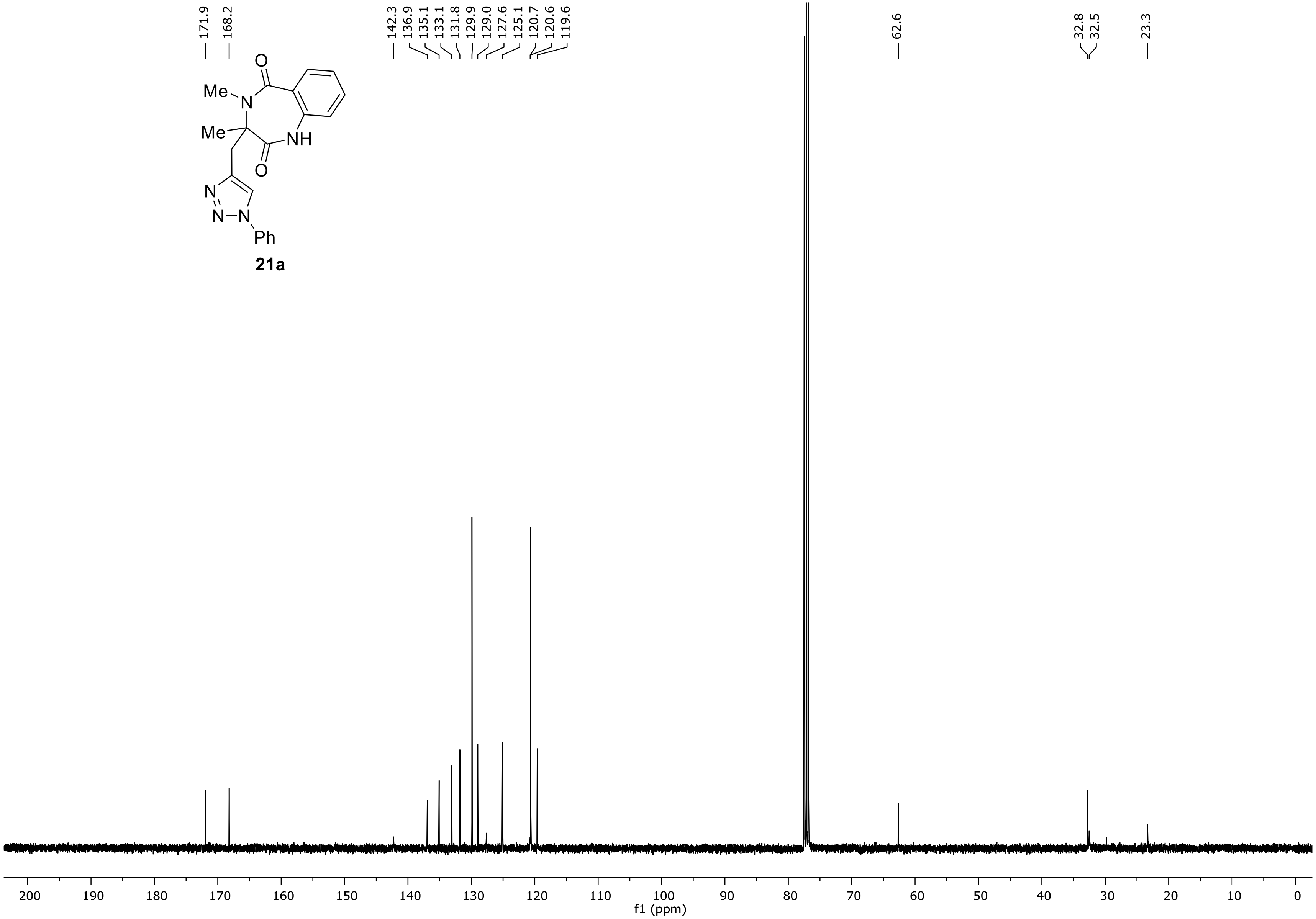
171.9
168.2
142.3
136.9
135.1
133.1
131.8
129.9
129.0
127.6
125.1
120.7
120.6
119.6
62.6
32.8
32.5
23.3
Me
N
Me
NH
O
O
N
N
N
Ph
200 190 180 170 160 150 140 130 120 110 100 90 80 70 60 50 40 30 20 10 0
f1 (ppm)

21a

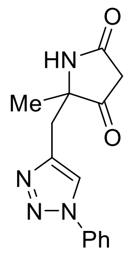
HN
Me
N
N
N
Ph

22a

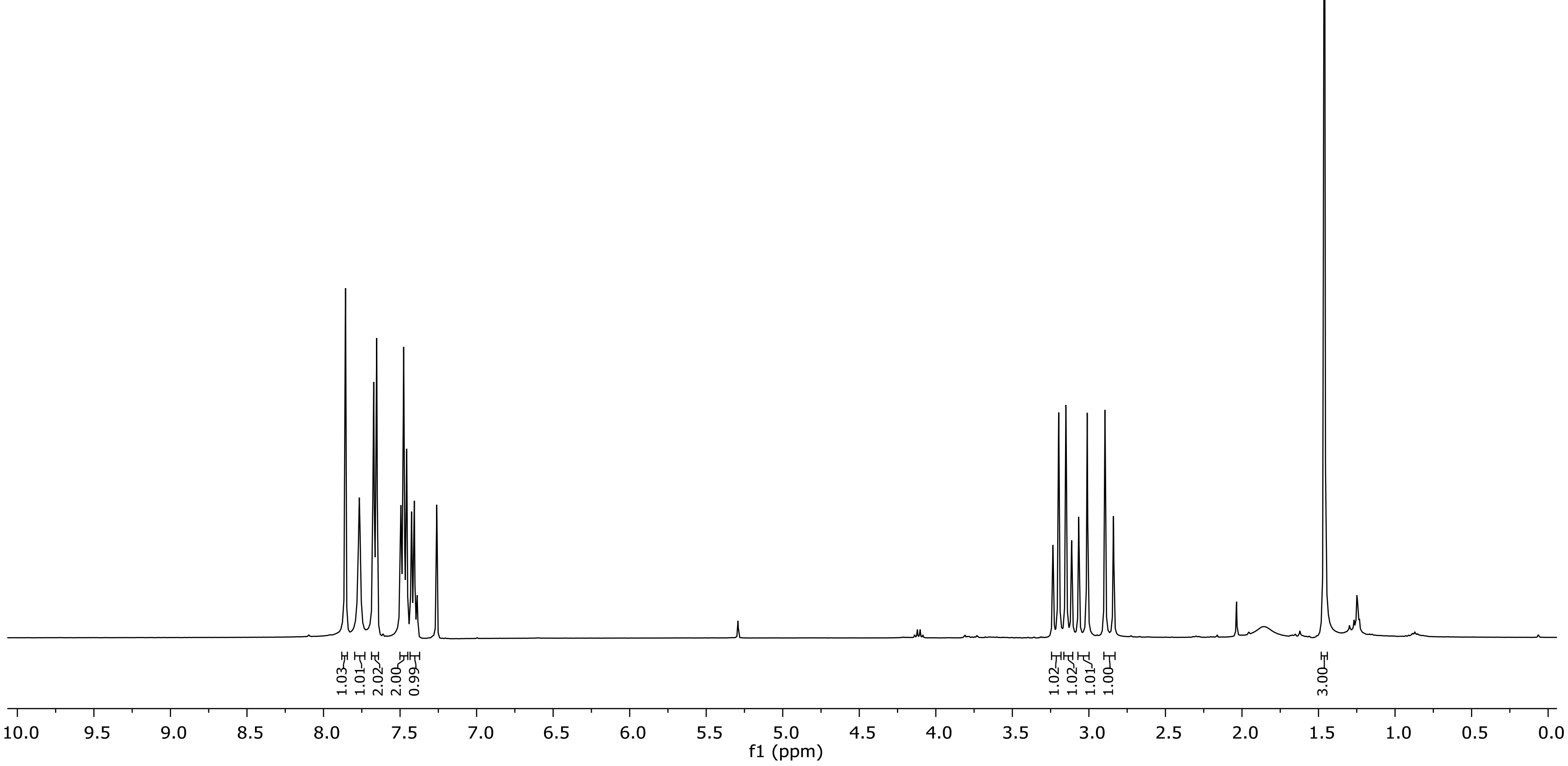
1.03
1.01
2.02
2.00
0.99
1.02
1.02
1.01
1.00
3.00
10.0 9.5 9.0 8.5 8.0 7.5 7.0 6.5 6.0 5.5 5.0 4.5 4.0 3.5 3.0 2.5 2.0 1.5 1.0 0.5 0.0
f1 (ppm)

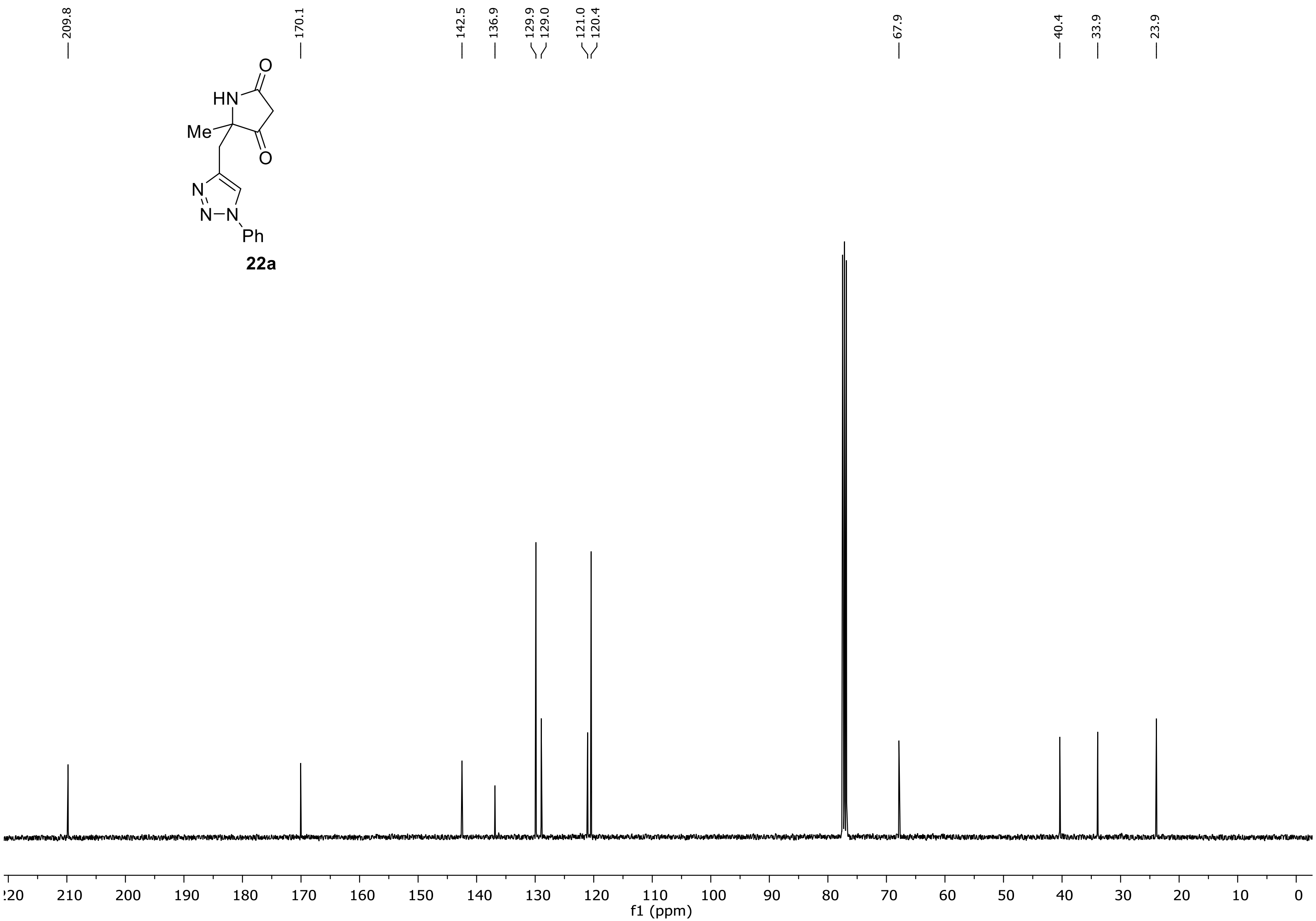
209.8
170.1
142.5
136.9
129.9
129.0
121.0
120.4
67.9
40.4
33.9
23.9
HN
O
Me
O
N
N
N
Ph
22a
220 210 200 190 180 170 160 150 140 130 120 110 100 90 80 70 60 50 40 30 20 10 0
f1 (ppm)

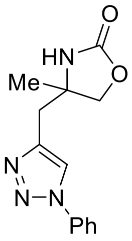

26a

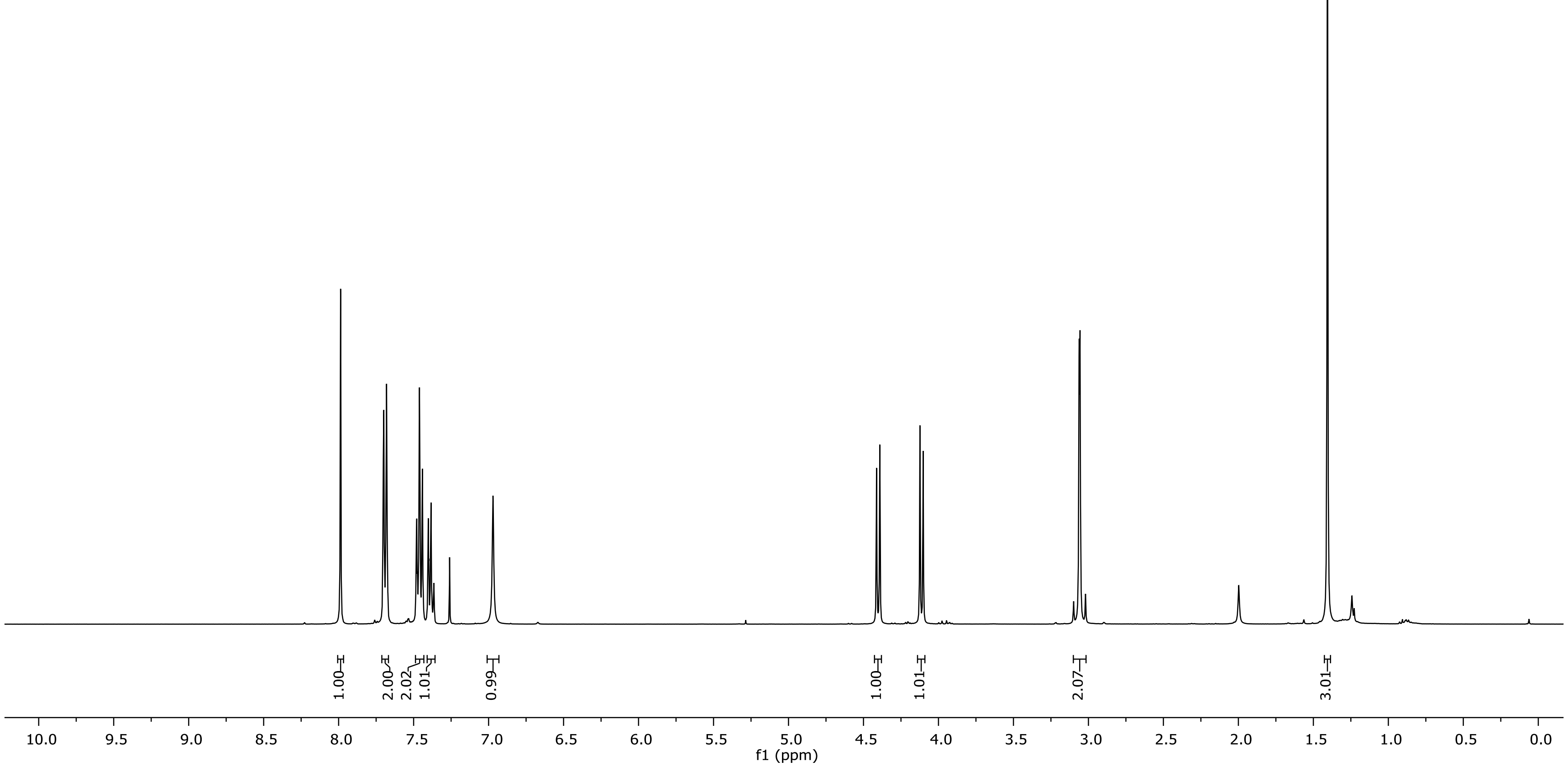
1.00
2.00
2.02
1.01
0.99
1.00
1.01
2.07
3.01
10.0 9.5 9.0 8.5 8.0 7.5 7.0 6.5 6.0 5.5 5.0 4.5 4.0 3.5 3.0 2.5 2.0 1.5 1.0 0.5 0.0
f1 (ppm)

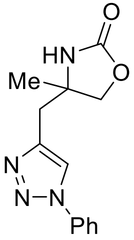
O
HN
O
Me
N
N
N
Ph

26a

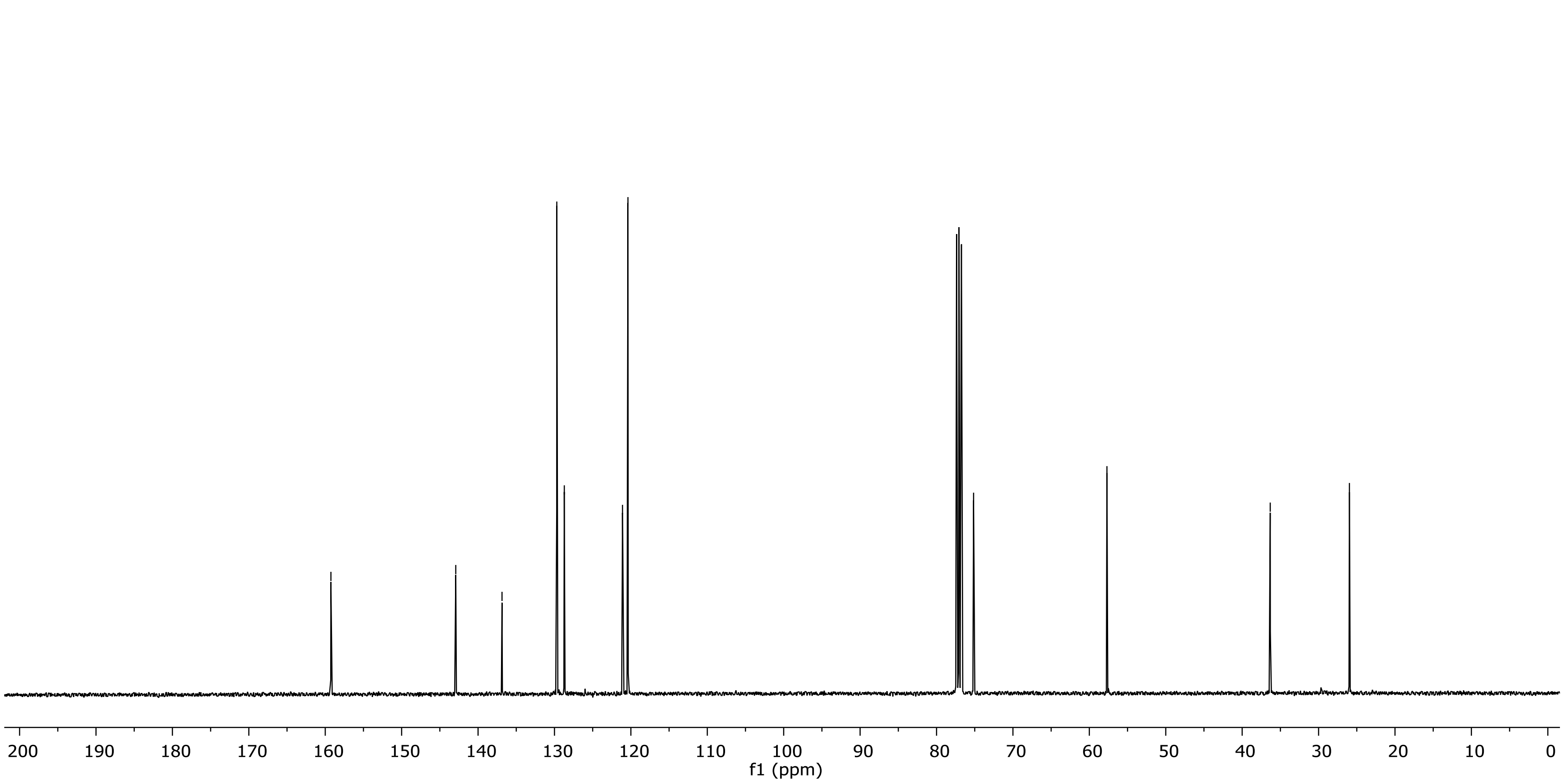
159.3
142.9
136.9
129.7
128.7
121.1
120.4
75.1
57.7
36.3
26.0
200 190 180 170 160 150 140 130 120 110 100 90 80 70 60 50 40 30 20 10 0
f1 (ppm)

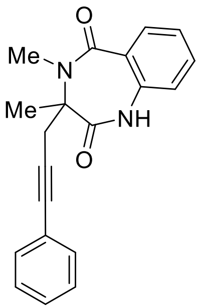
O
Me
N
Me
NH
O

21b

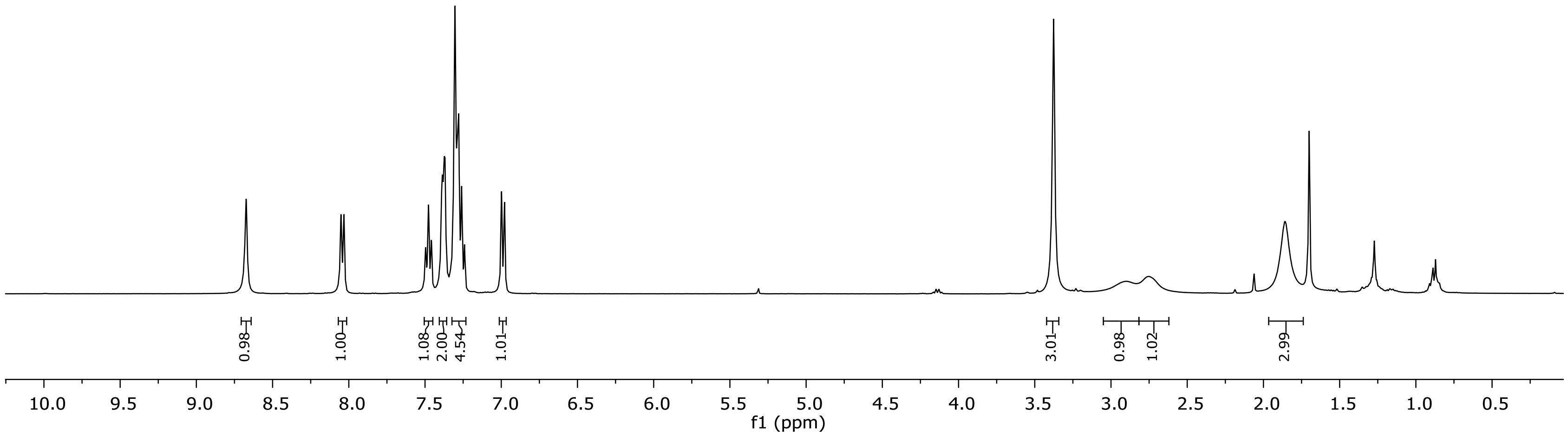
0.98
1.00
1.08
2.00
4.54
1.01
3.01
0.98
1.02
2.99
10.0
9.5
9.0
8.5
8.0
7.5
7.0
6.5
6.0
5.5
5.0
4.5
4.0
3.5
3.0
2.5
2.0
1.5
1.0
0.5
f1 (ppm)

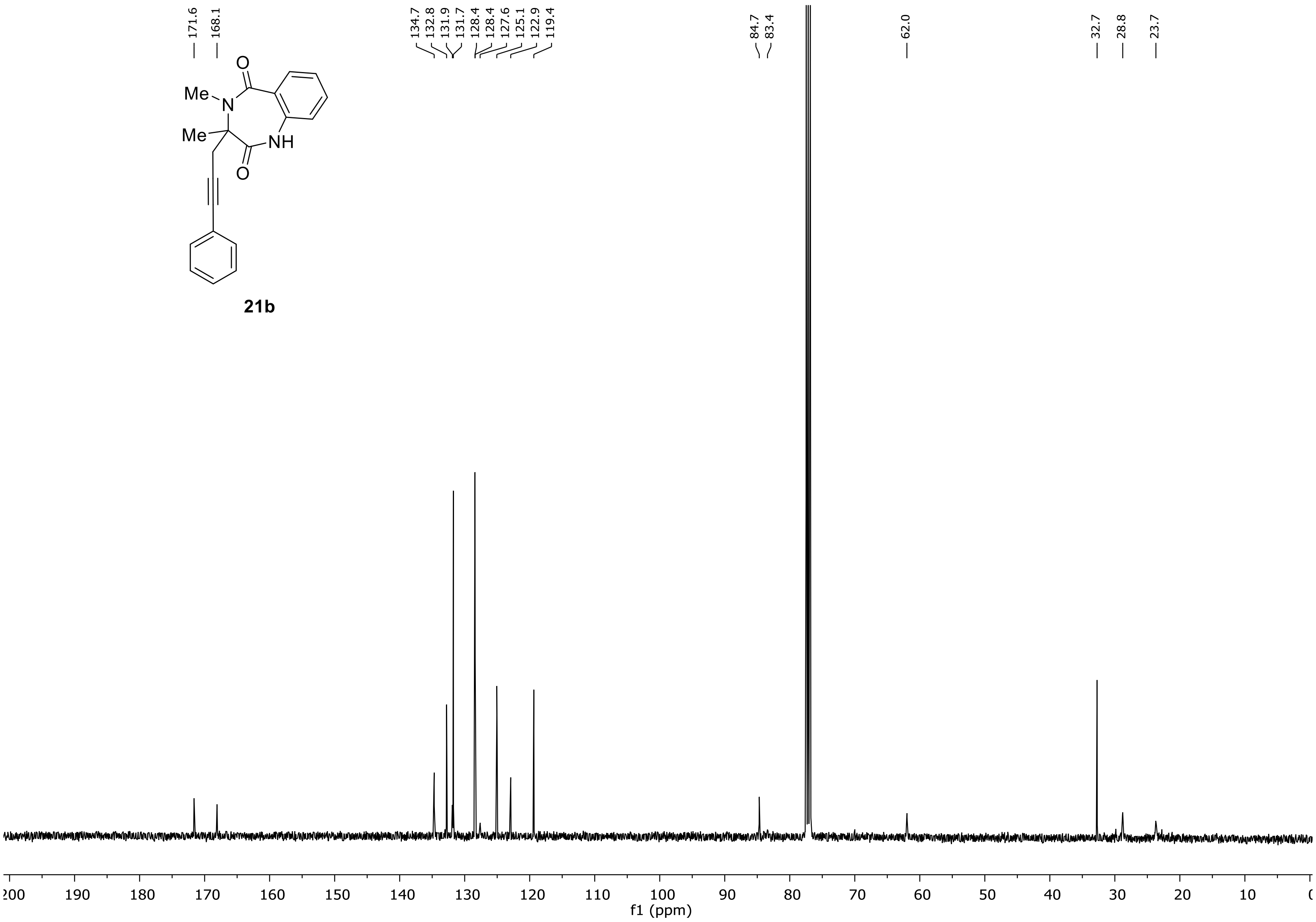
171.6
168.1
134.7
132.8
131.9
131.7
128.4
128.4
127.6
125.1
122.9
119.4
84.7
83.4
62.0
32.7
28.8
23.7
Me
N
O
Me
NH
O
21b
200
190
180
170
160
150
140
130
120
110
100
90
80
70
60
50
40
30
20
10
f1 (ppm)

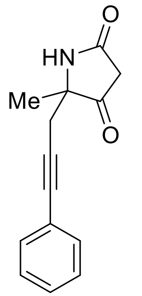
O
HN
Me
O

22b

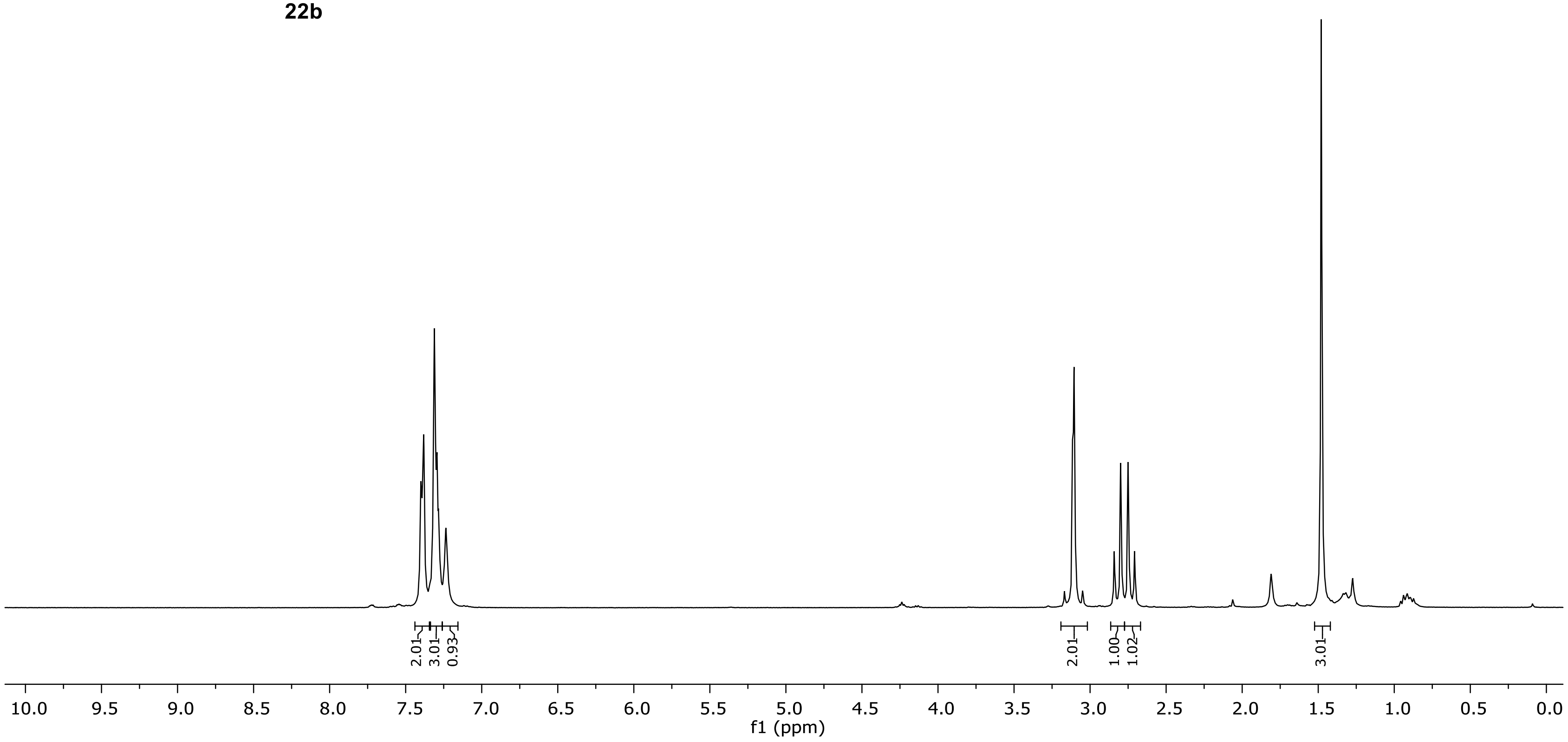
2.01
3.01
0.93
2.01
1.00
1.02
3.01
10.0 9.5 9.0 8.5 8.0 7.5 7.0 6.5 6.0 5.5 5.0 4.5 4.0 3.5 3.0 2.5 2.0 1.5 1.0 0.5 0.0
f1 (ppm)

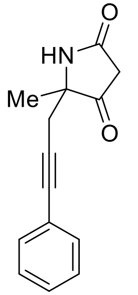
O
HN
Me
O

22b

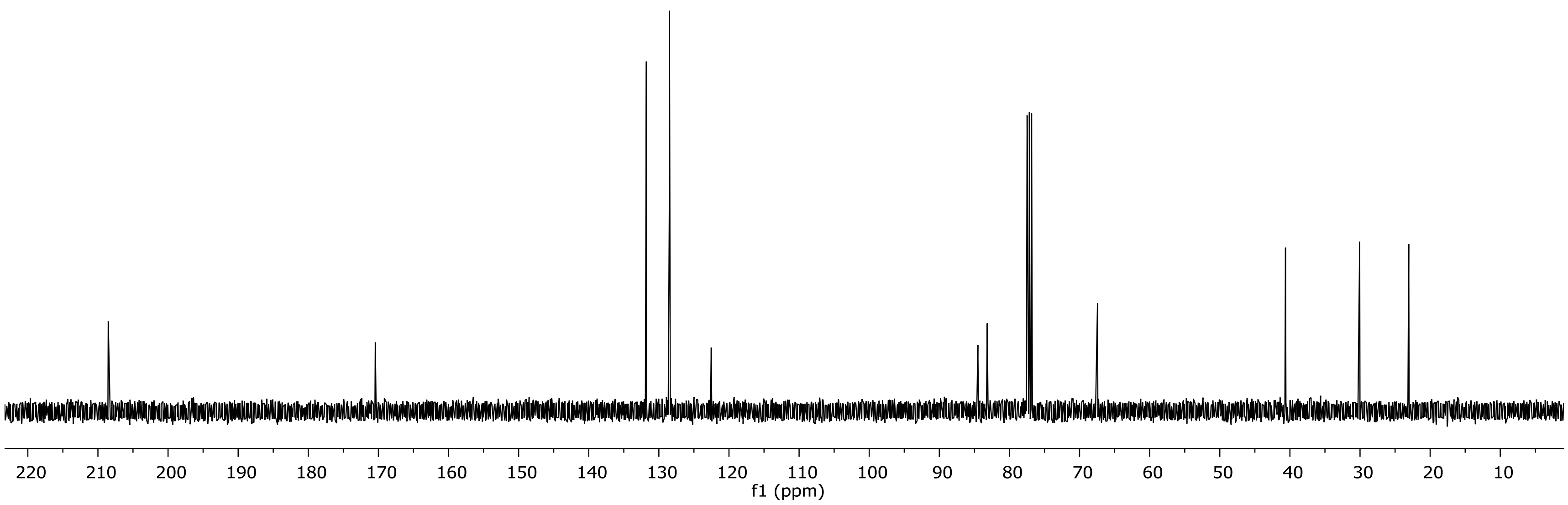
208.5
170.4
131.8
128.6
128.5
122.5
84.5
83.2
67.4
40.6
30.0
23.1
220 210 200 190 180 170 160 150 140 130 120 110 100 90 80 70 60 50 40 30 20 10
f1 (ppm)

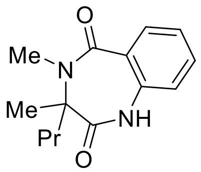
O
Me
N
Me
Pr
NH
O

21c

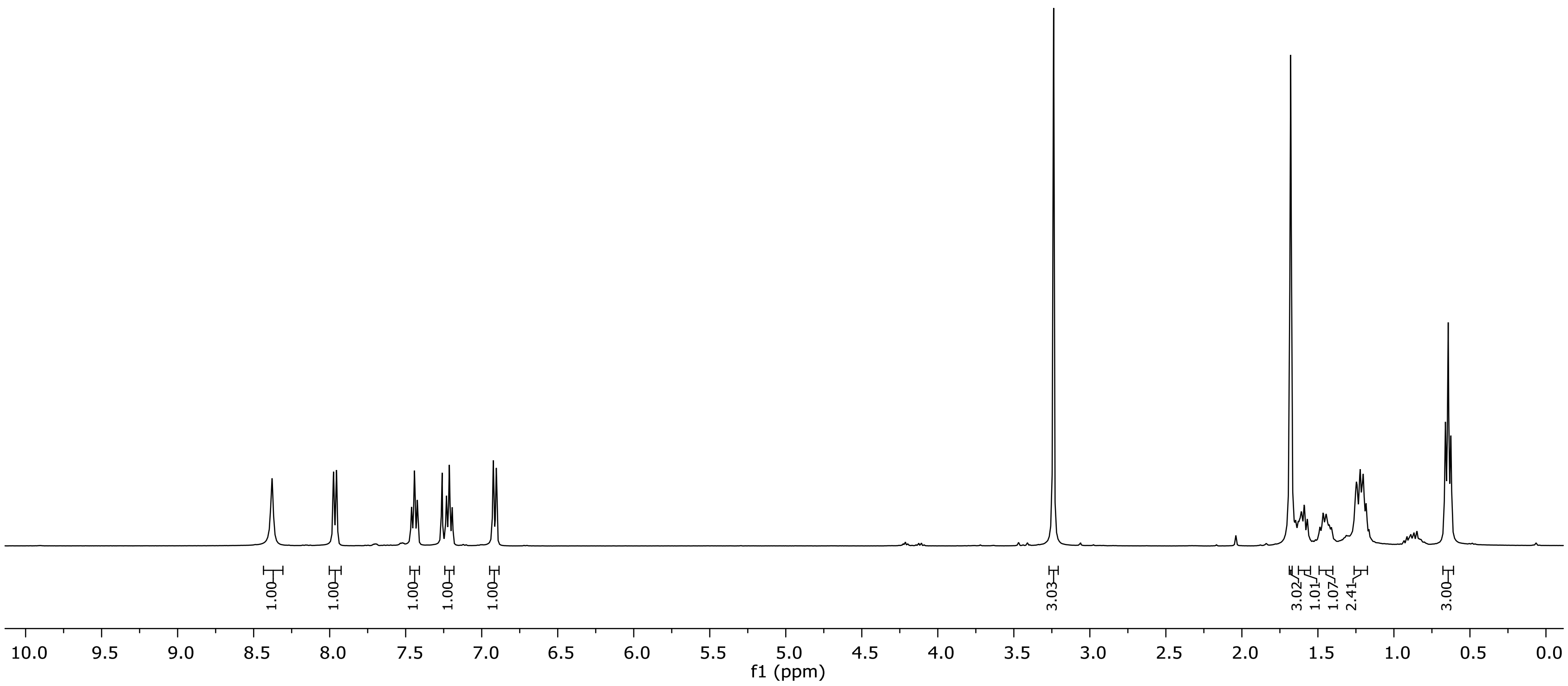
1.00
1.00
1.00
1.00
1.00
3.03
3.02
1.01
1.07
2.41
3.00
10.0
9.5
9.0
8.5
8.0
7.5
7.0
6.5
6.0
5.5
5.0
4.5
4.0
3.5
3.0
2.5
2.0
1.5
1.0
0.5
0.0
f1 (ppm)

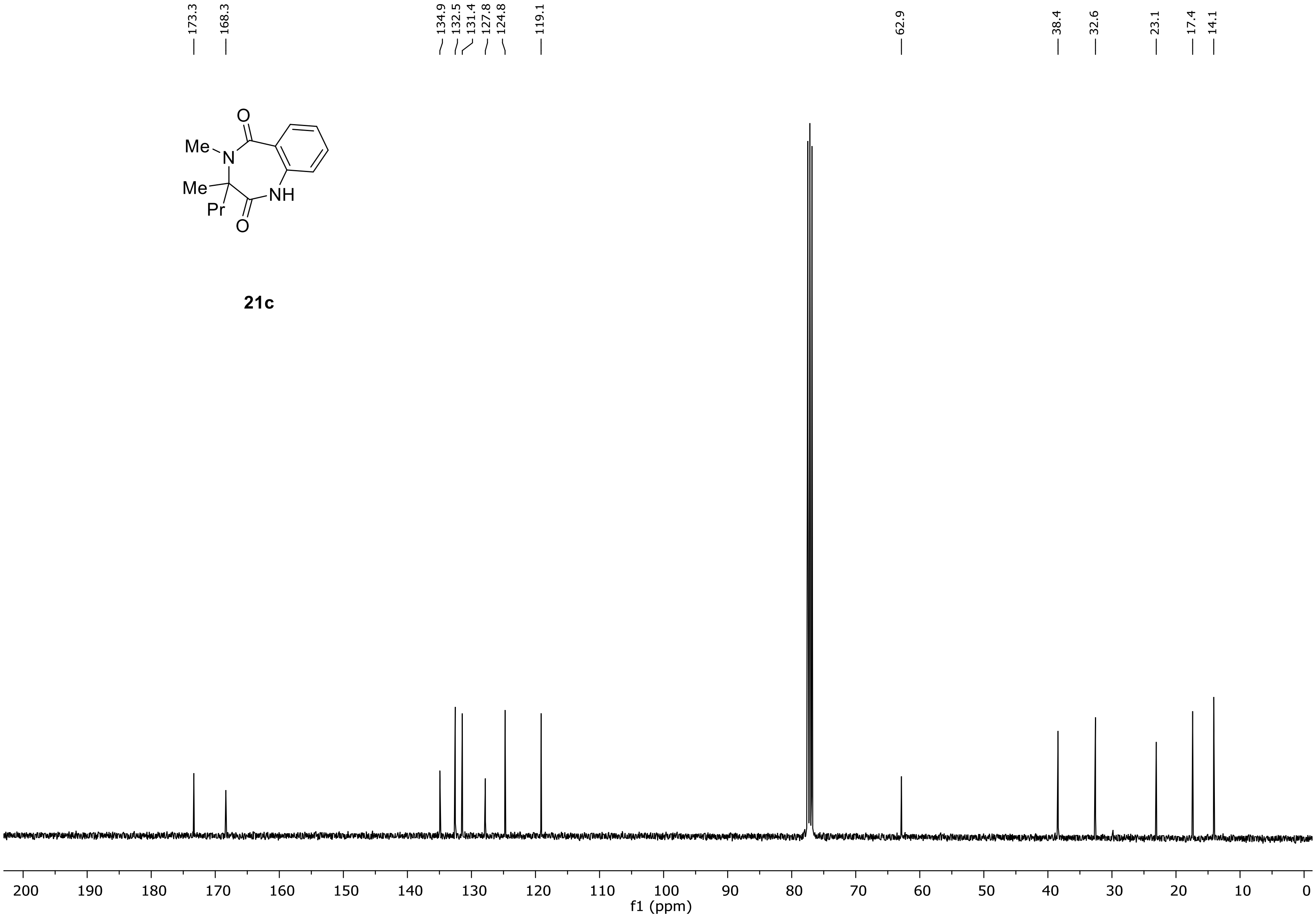
173.3
168.3
134.9
132.5
131.4
127.8
124.8
119.1
62.9
38.4
32.6
23.1
17.4
14.1
O
Me
N
Me
Pr
NH
O
21c
200 190 180 170 160 150 140 130 120 110 100 90 80 70 60 50 40 30 20 10 0
f1 (ppm)

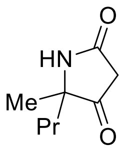
O
HN
Me
Pr
O

22c

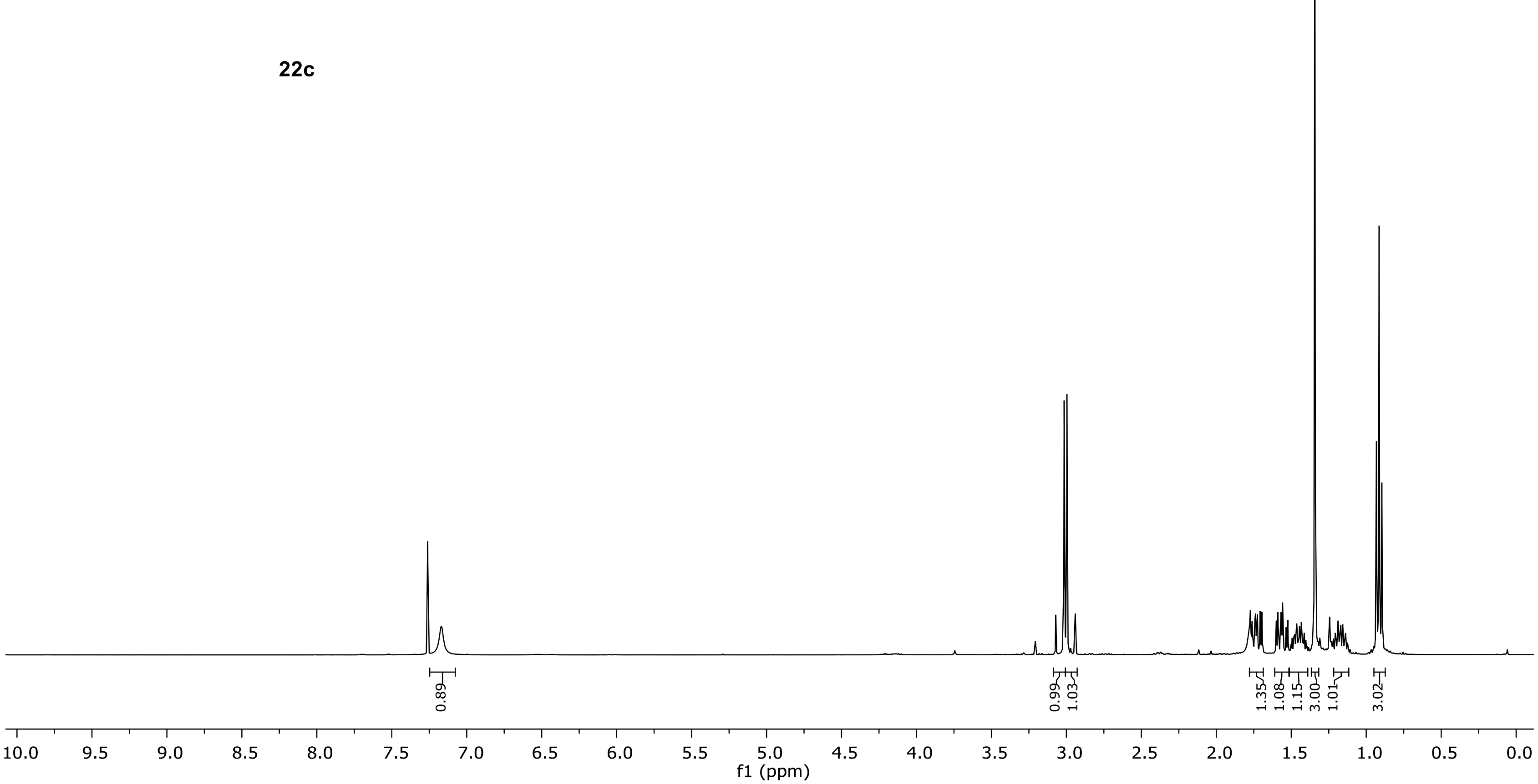
0.89
0.99
1.03
1.35
1.08
1.15
3.00
1.01
3.02
10.0 9.5 9.0 8.5 8.0 7.5 7.0 6.5 6.0 5.5 5.0 4.5 4.0 3.5 3.0 2.5 2.0 1.5 1.0 0.5 0.0
f1 (ppm)

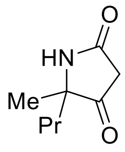

22c

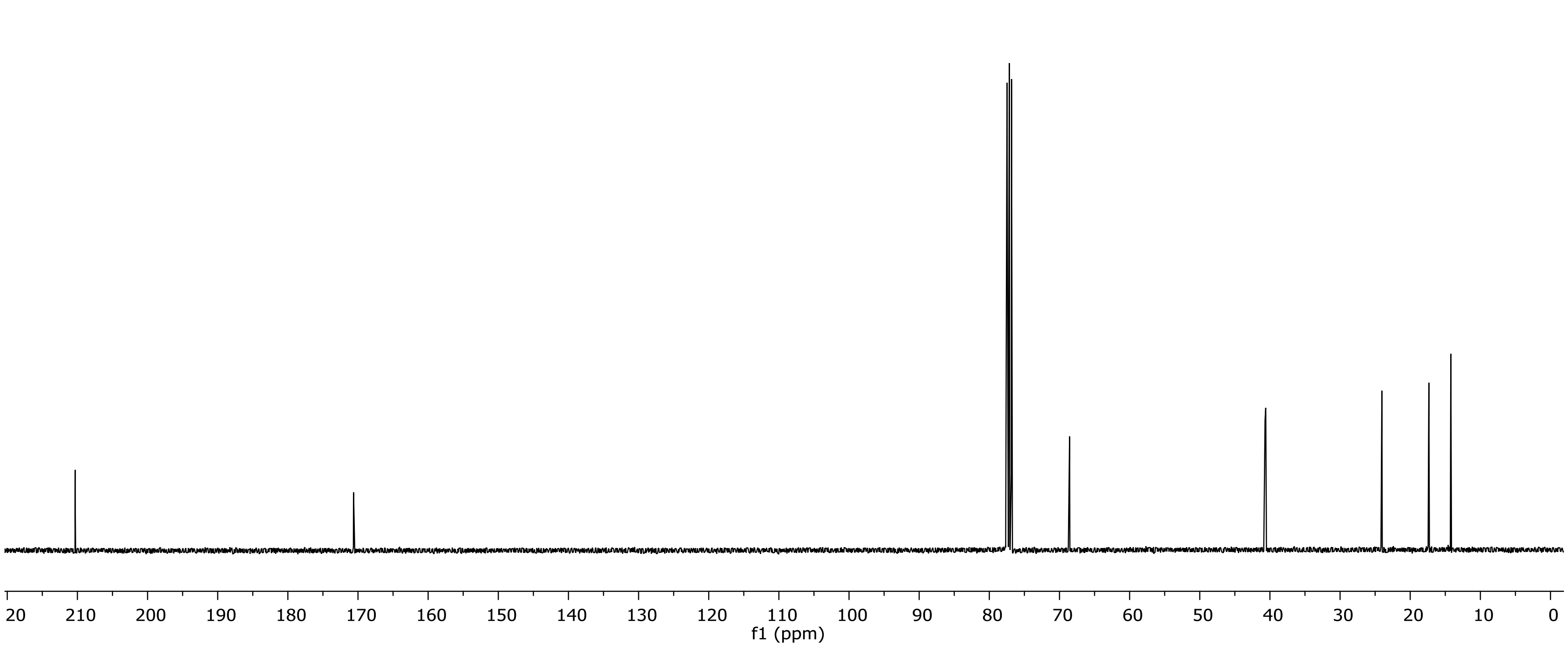

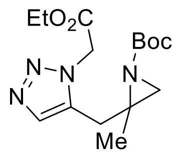

23d

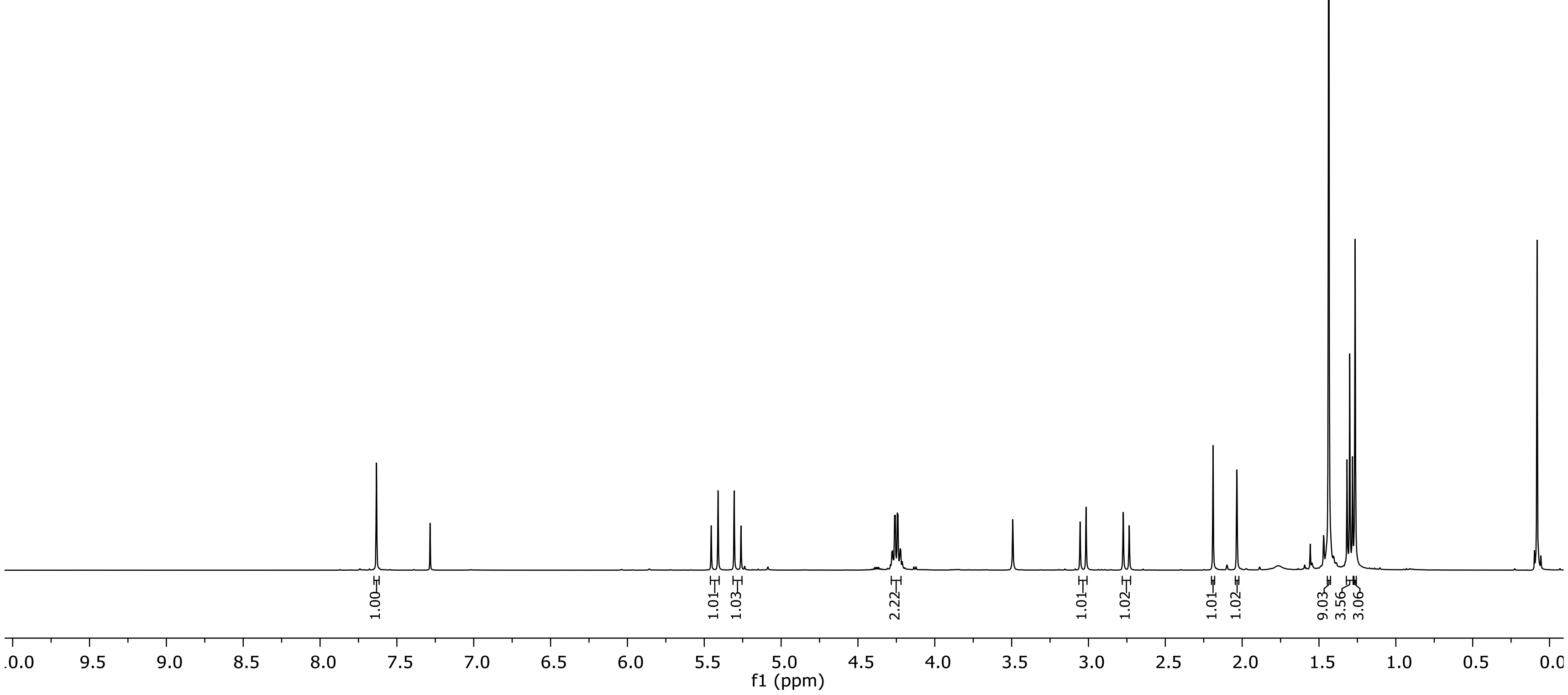

1.00
1.01
1.03
2.22
1.01
1.02
1.01
1.02
9.03
3.56
3.06
.0.0
9.5
9.0
8.5
8.0
7.5
7.0
6.5
6.0
5.5
5.0
4.5
4.0
3.5
3.0
2.5
2.0
1.5
1.0
0.5
0.0
f1 (ppm)

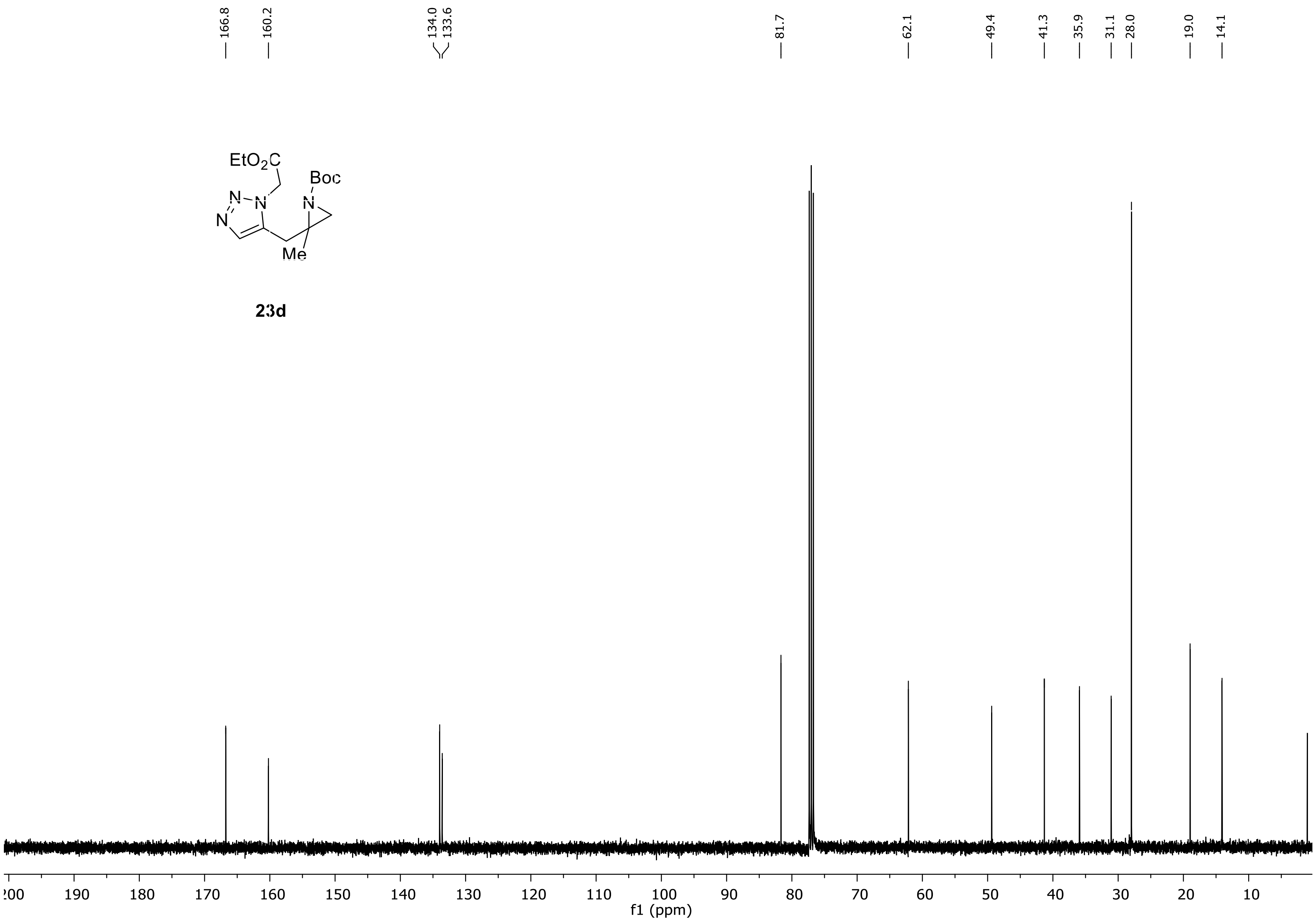
166.8
160.2
134.0
133.6
81.7
62.1
49.4
41.3
35.9
31.1
28.0
19.0
14.1
EtO2C
Boc
N
N
N
N
Me
23d
200 190 180 170 160 150 140 130 120 110 100 90 80 70 60 50 40 30 20 10
f1 (ppm)

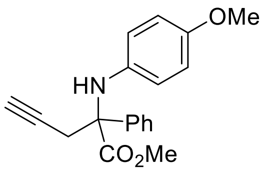
OMe
HN
Ph
CO_2Me

S25

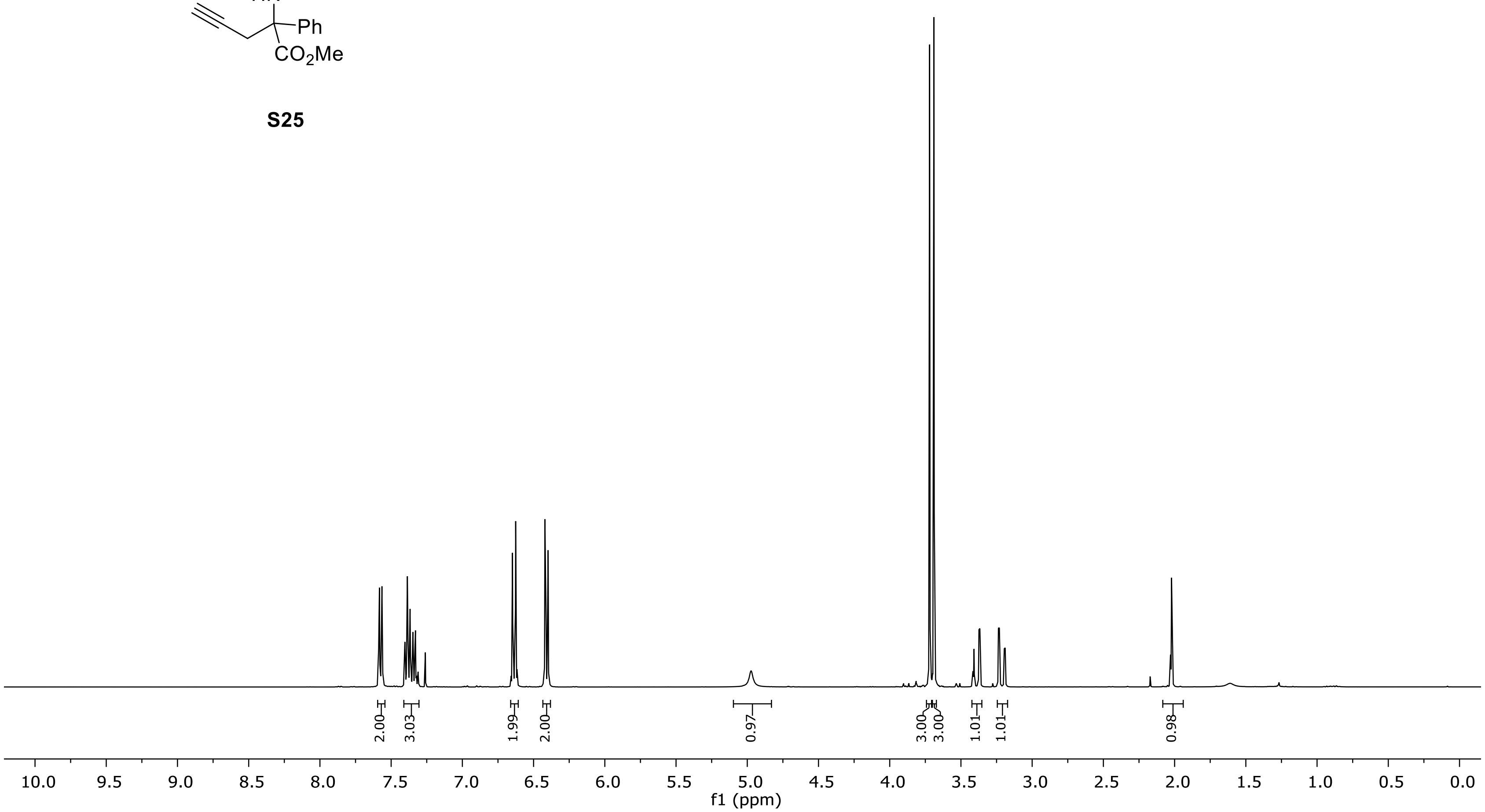
2.00
3.03
1.99
2.00
0.97
3.00
3.00
1.01
1.01
0.98
10.0
9.5
9.0
8.5
8.0
7.5
7.0
6.5
6.0
5.5
5.0
4.5
4.0
3.5
3.0
2.5
2.0
1.5
1.0
0.5
0.0
f1 (ppm)

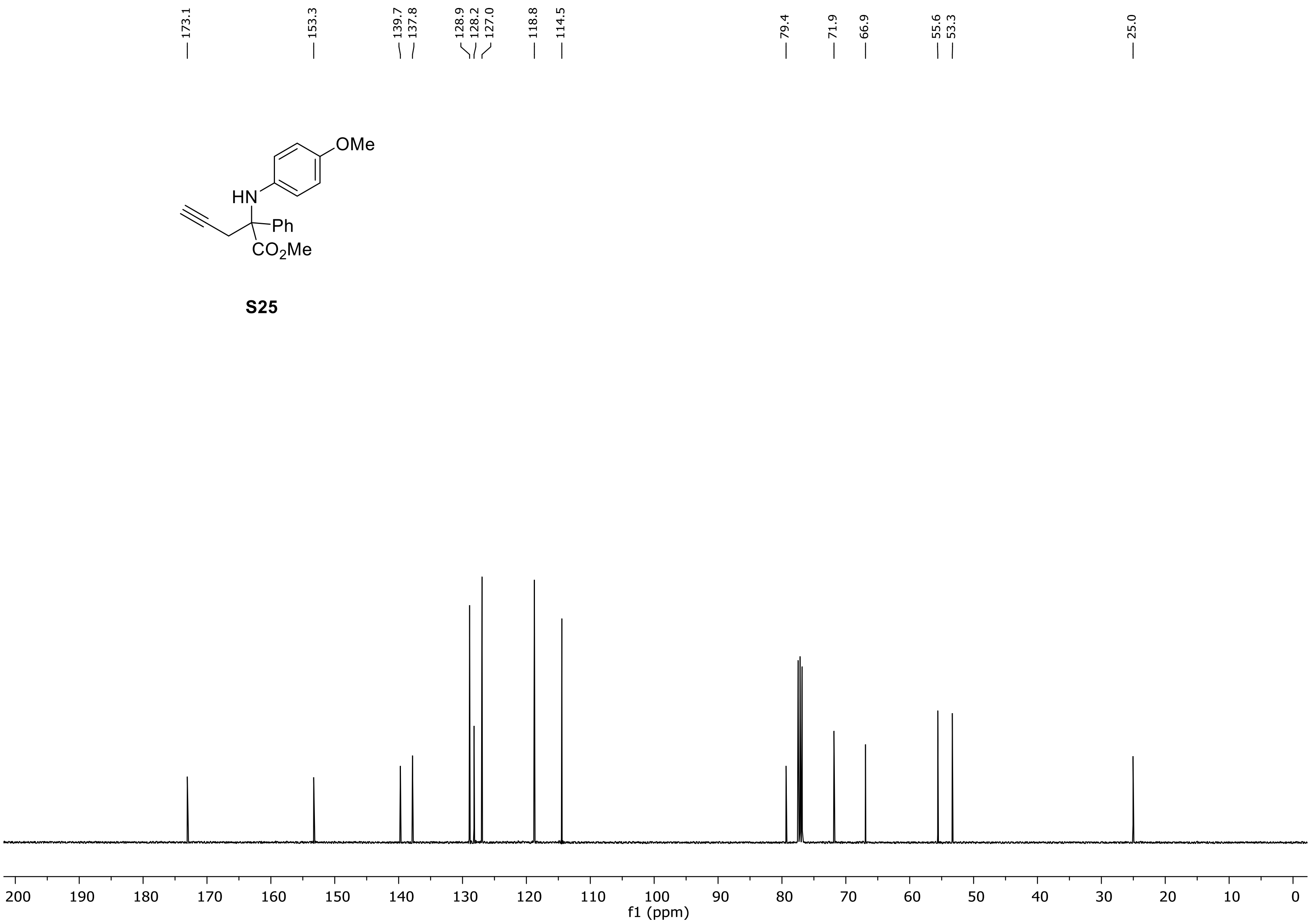
173.1
153.3
139.7
137.8
128.9
128.2
127.0
118.8
114.5
79.4
71.9
66.9
55.6
53.3
25.0
OMe
HN
Ph
CO_2Me
200 190 180 170 160 150 140 130 120 110 100 90 80 70 60 50 40 30 20 10 0
f1 (ppm)

S25

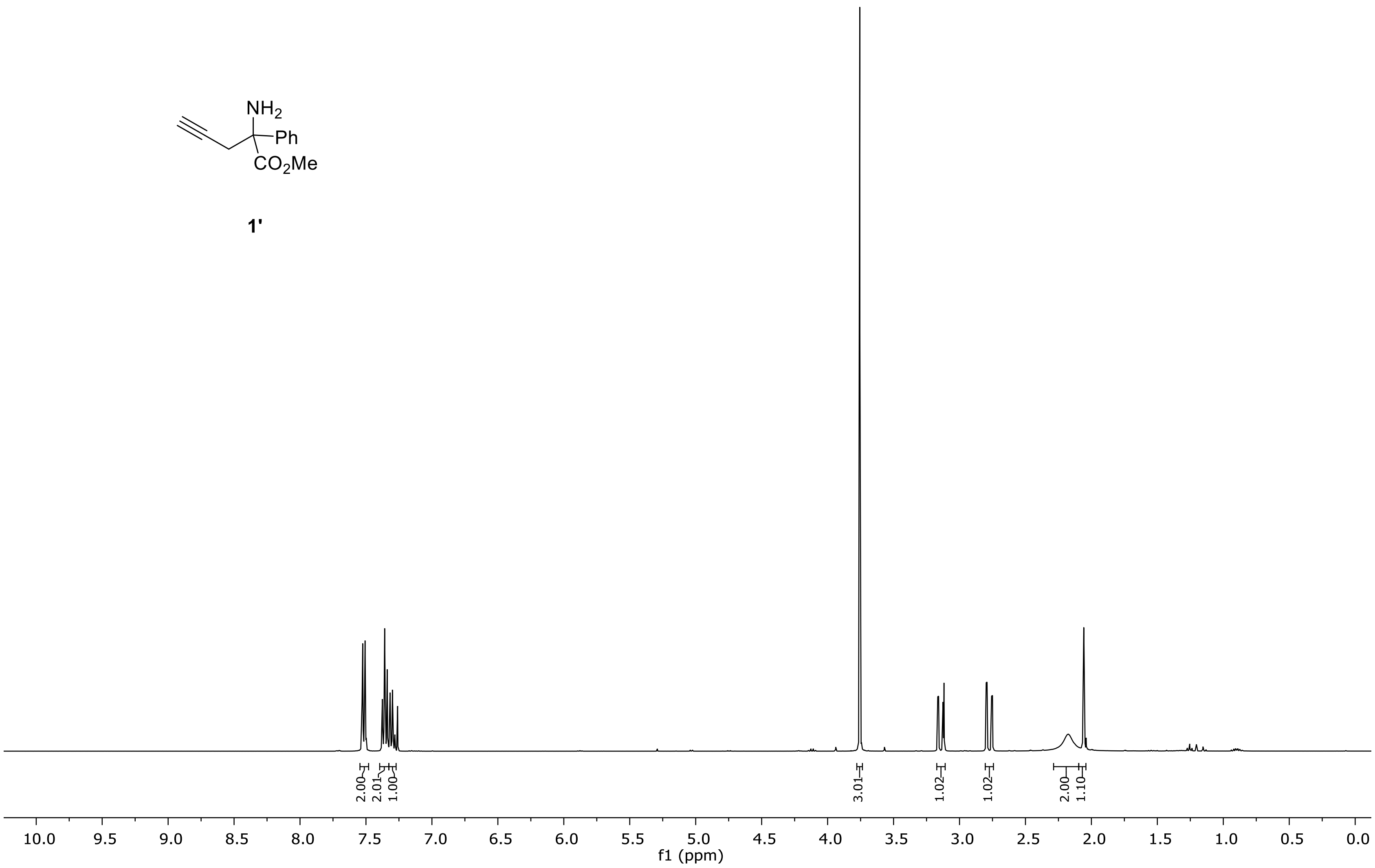

NH2
Ph
CO2Me
1'
2.00
2.01
1.00
3.01
1.02
1.02
2.00
1.10
10.0 9.5 9.0 8.5 8.0 7.5 7.0 6.5 6.0 5.5 5.0 4.5 4.0 3.5 3.0 2.5 2.0 1.5 1.0 0.5 0.0
f1 (ppm)

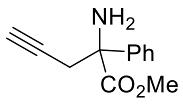
NH2
Ph
CO2Me

1'

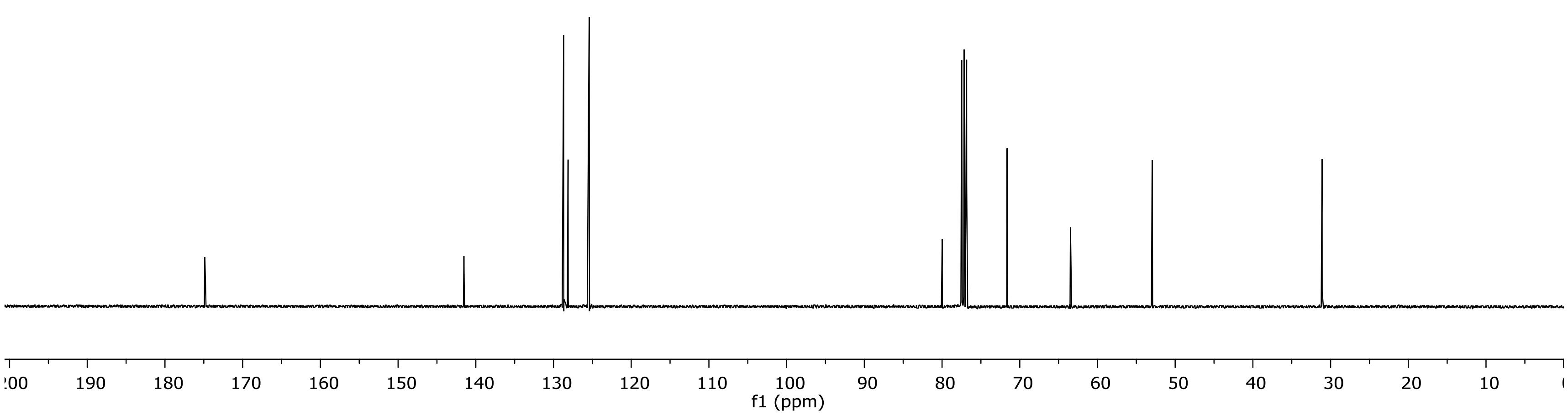
174.9
141.5
128.7
128.1
125.4
80.0
71.6
63.5
53.0
31.1
200
190
180
170
160
150
140
130
120
110
100
90
80
70
60
50
40
30
20
10
f1 (ppm)

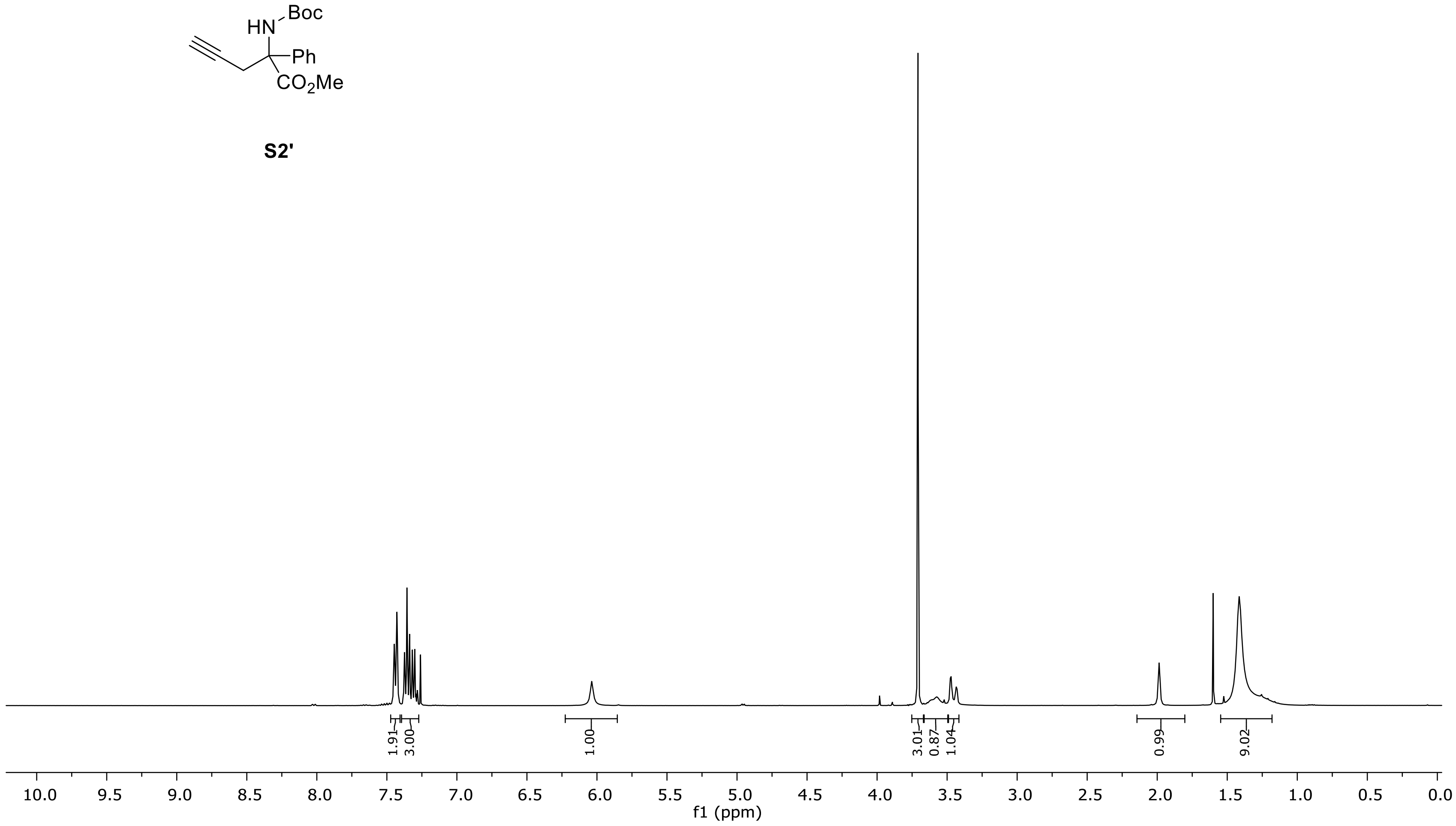
HN
Boc
Ph
CO2Me

S2'
1.91
3.00
1.00
3.01
0.87
1.04
0.99
9.02
10.0 9.5 9.0 8.5 8.0 7.5 7.0 6.5 6.0 5.5 5.0 4.5 4.0 3.5 3.0 2.5 2.0 1.5 1.0 0.5 0.0
f1 (ppm)

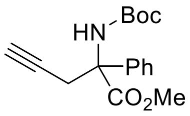

Boc
HN
Ph
CO_2Me

S2'

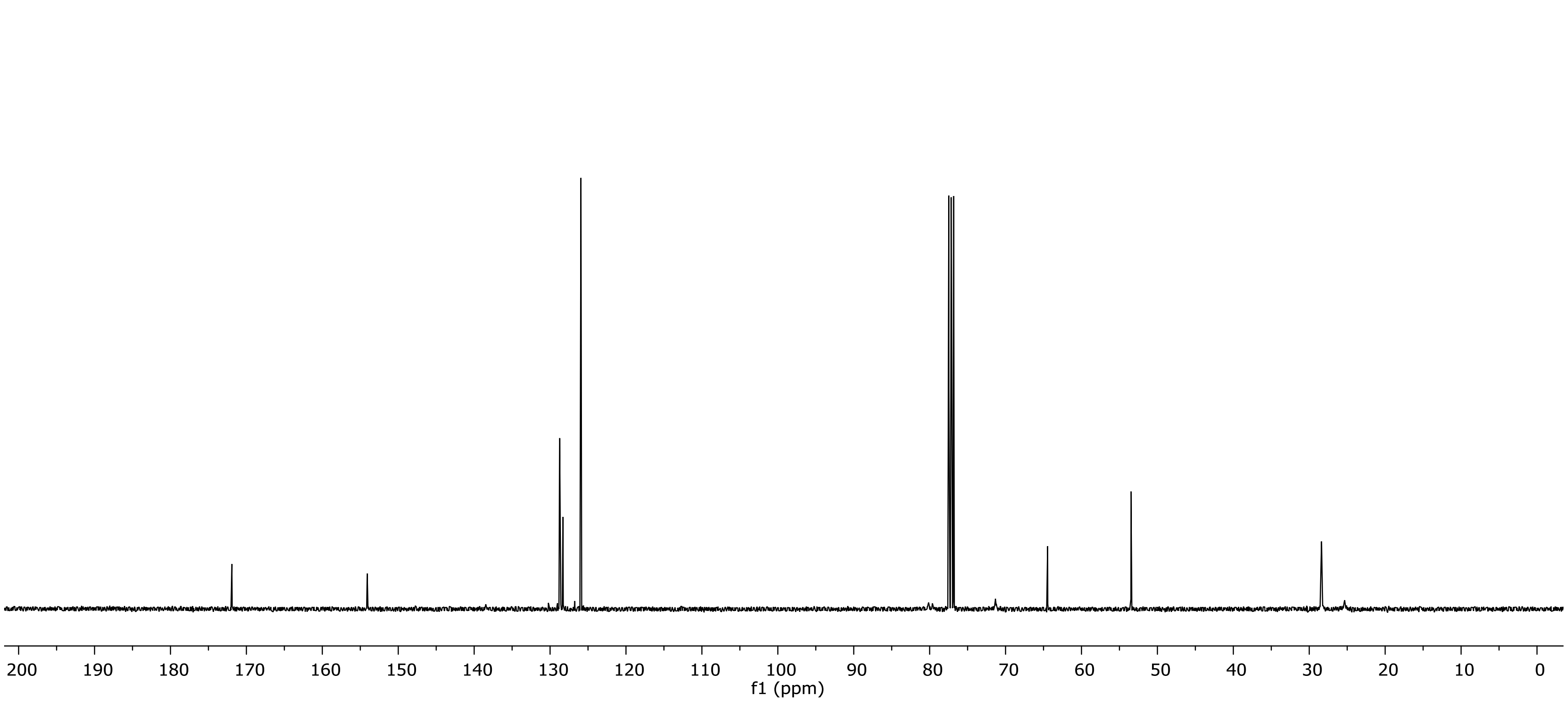

171.9
154.1
138.5
128.7
128.3
125.9
80.2
79.6
71.3
64.5
53.5
28.4
25.3
200
190
180
170
160
150
140
130
120
110
100
90
80
70
60
50
40
30
20
10
0
f1 (ppm)

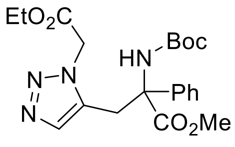
EtO2C
HN
Boc
Ph
CO2Me
N
N
N

S9'

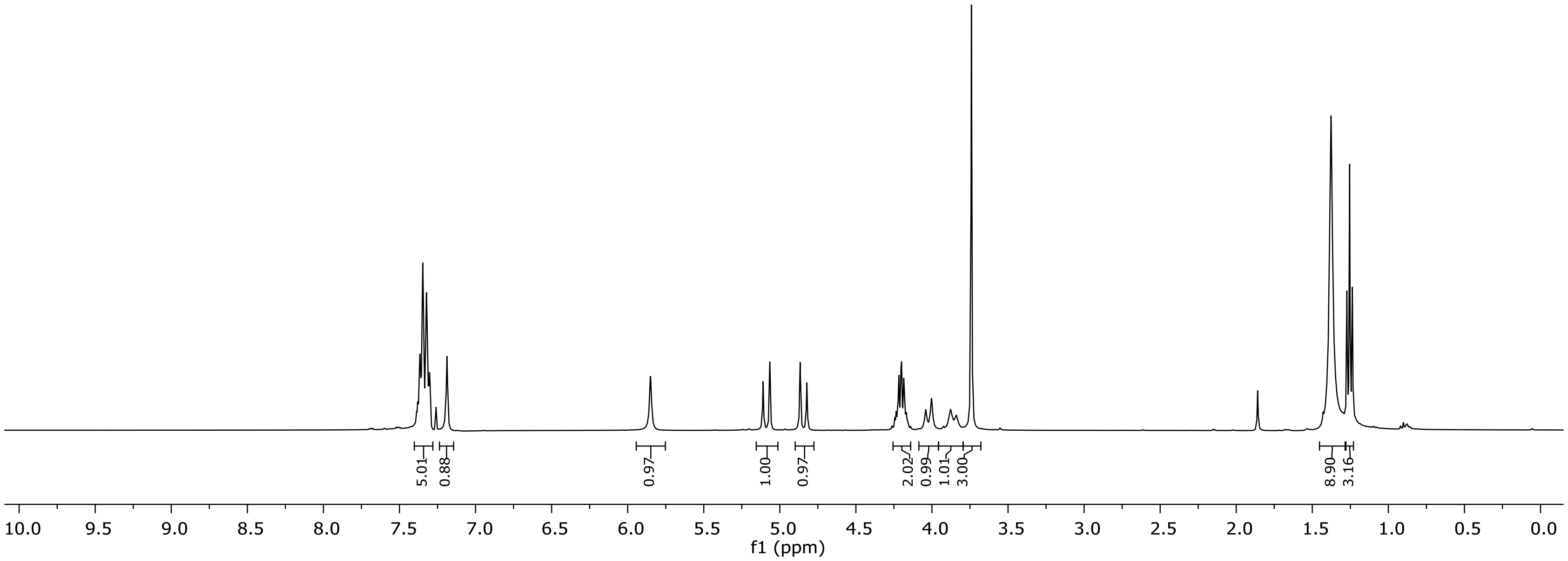
5.01
0.88
0.97
1.00
0.97
2.02
0.99
1.01
3.00
8.90
3.16
10.0 9.5 9.0 8.5 8.0 7.5 7.0 6.5 6.0 5.5 5.0 4.5 4.0 3.5 3.0 2.5 2.0 1.5 1.0 0.5 0.0
f1 (ppm)

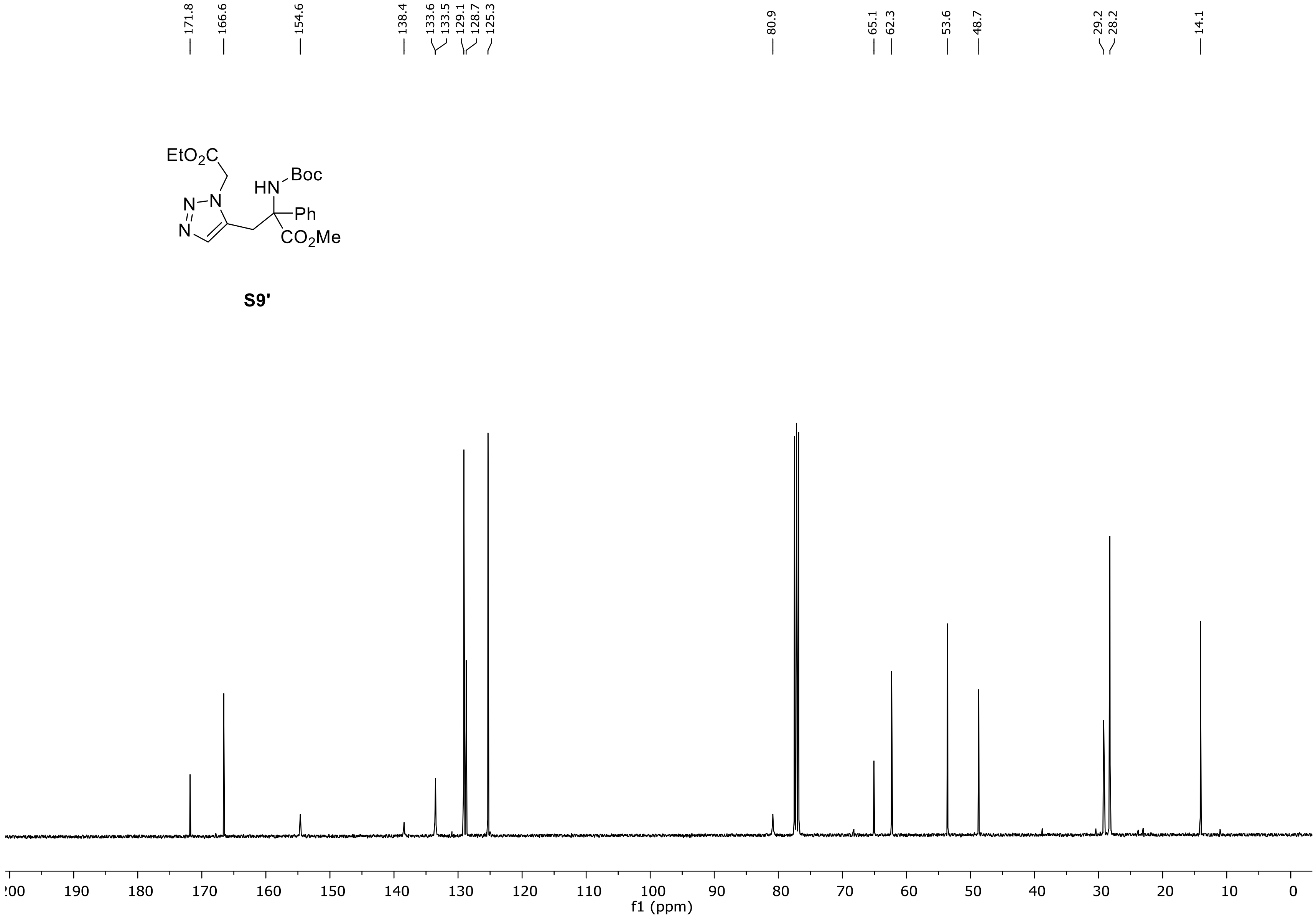

EtO2C
HN
Boc
N
N
N
Ph
CO2Me

171.8
166.6
154.6
138.4
133.6
133.5
129.1
128.7
125.3
80.9
65.1
62.3
53.6
48.7
29.2
28.2
14.1
f1 (ppm)

S9'

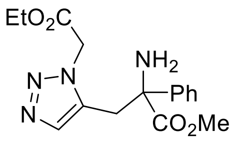

8'

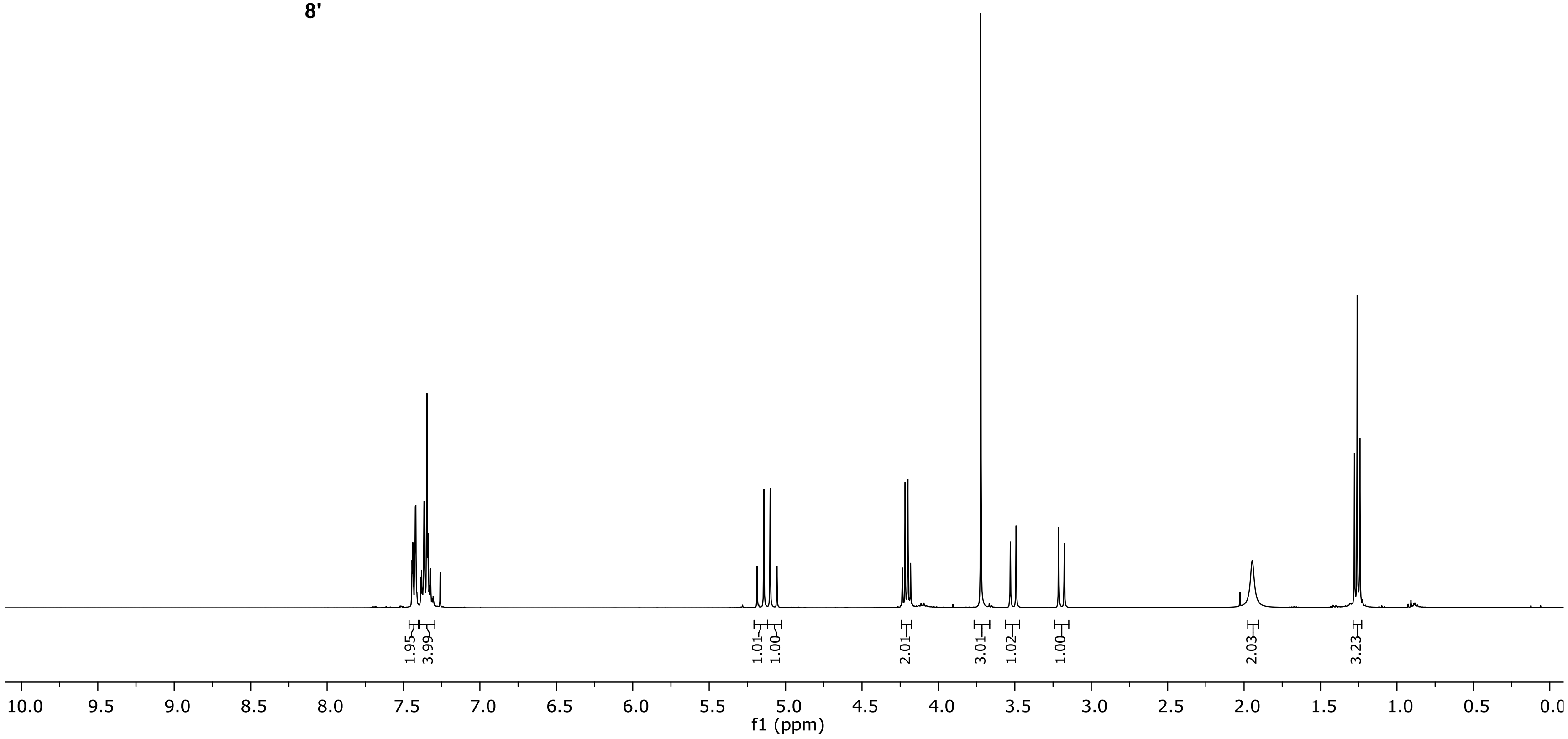
1.95
3.99
1.01
1.00
2.01
3.01
1.02
1.00
2.03
3.23
10.0 9.5 9.0 8.5 8.0 7.5 7.0 6.5 6.0 5.5 5.0 4.5 4.0 3.5 3.0 2.5 2.0 1.5 1.0 0.5 0.0
f1 (ppm)

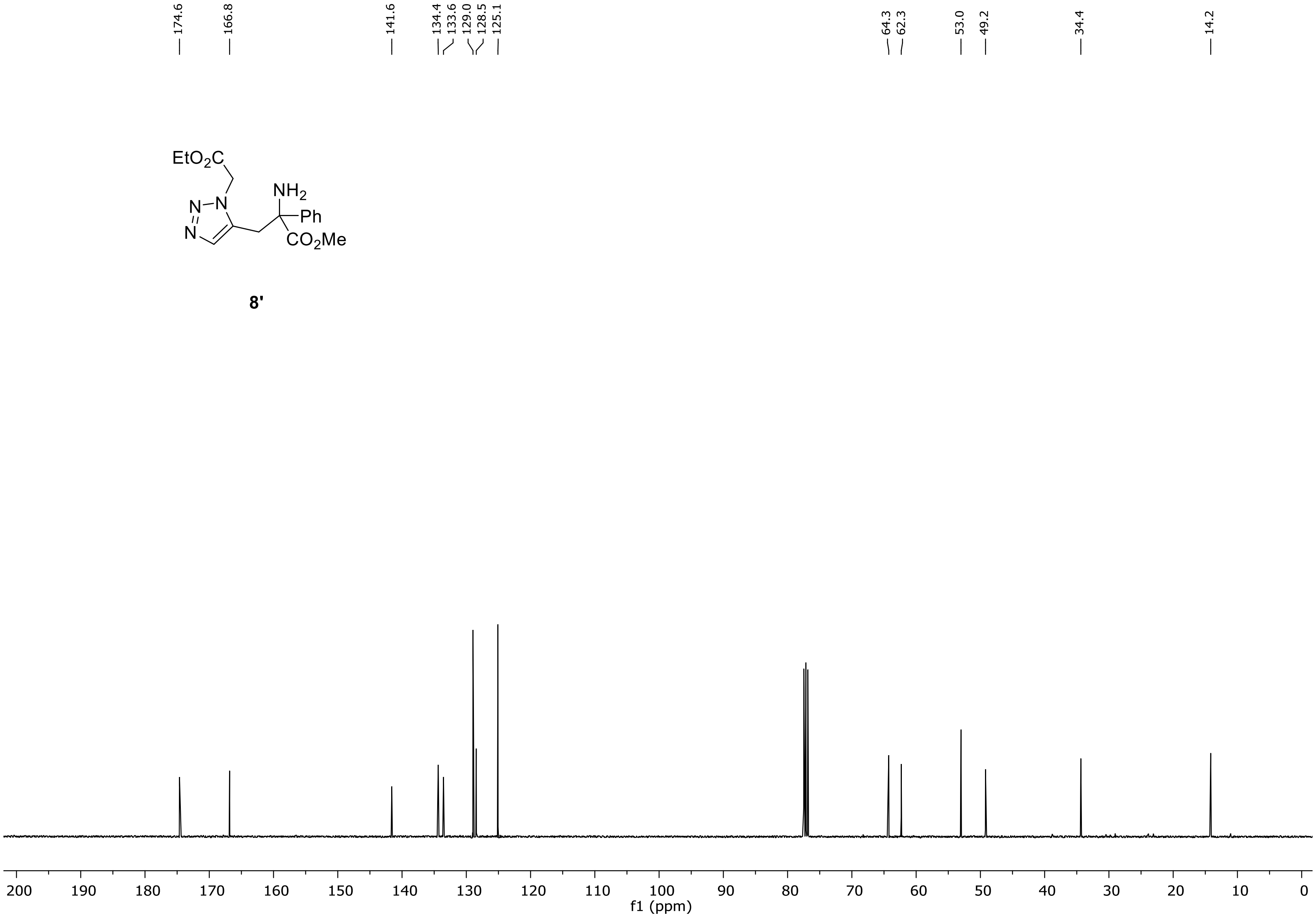
174.6
166.8
141.6
134.4
133.6
129.0
128.5
125.1
64.3
62.3
53.0
49.2
34.4
14.2
EtO2C
NH2
N
N
N
Ph
CO2Me
8'
200 190 180 170 160 150 140 130 120 110 100 90 80 70 60 50 40 30 20 10 0
f1 (ppm)

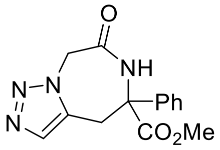
O
NH
N
N
N
Ph
CO_2Me

10'

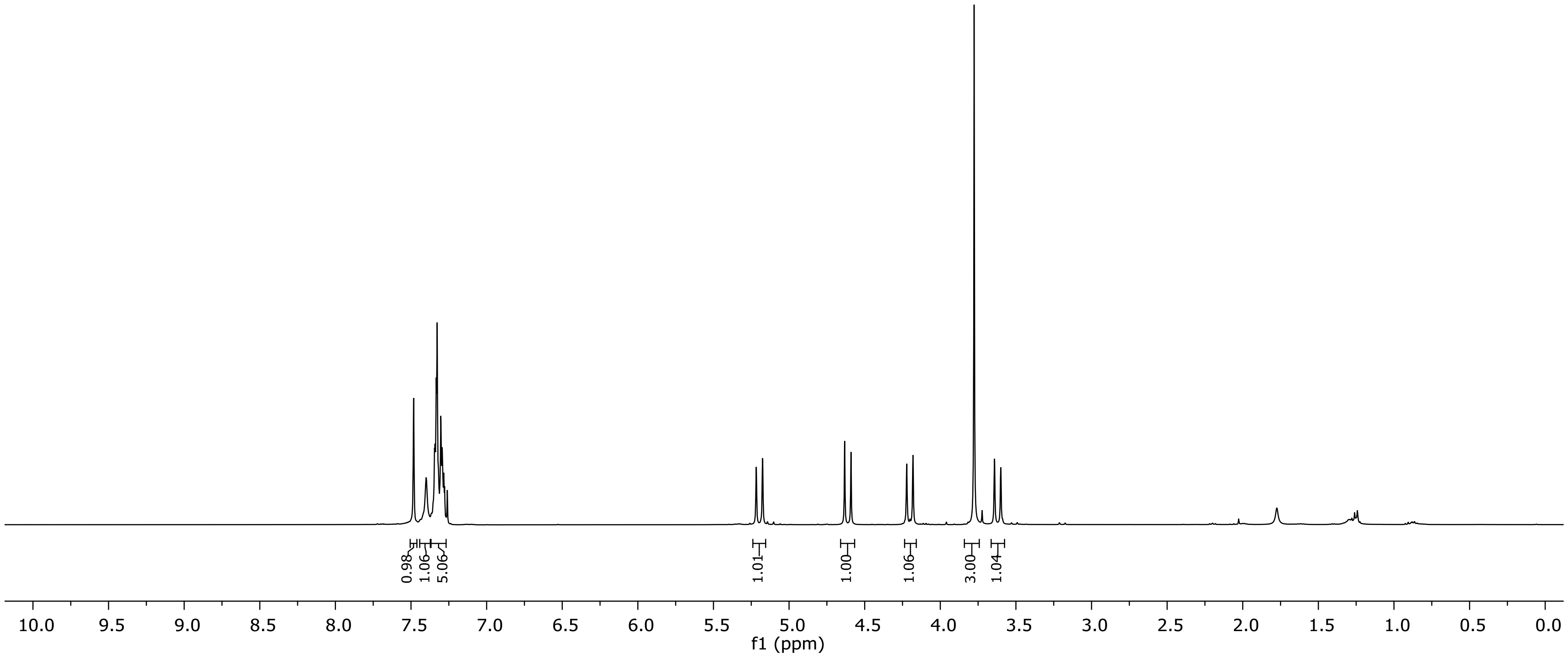
0.98
1.06
5.06
1.01
1.00
1.06
3.00
1.04
10.0 9.5 9.0 8.5 8.0 7.5 7.0 6.5 6.0 5.5 5.0 4.5 4.0 3.5 3.0 2.5 2.0 1.5 1.0 0.5 0.0
f1 (ppm)

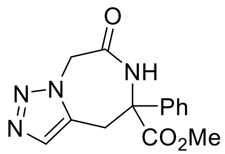
O
NH
N
N
N
Ph
CO2Me

10'

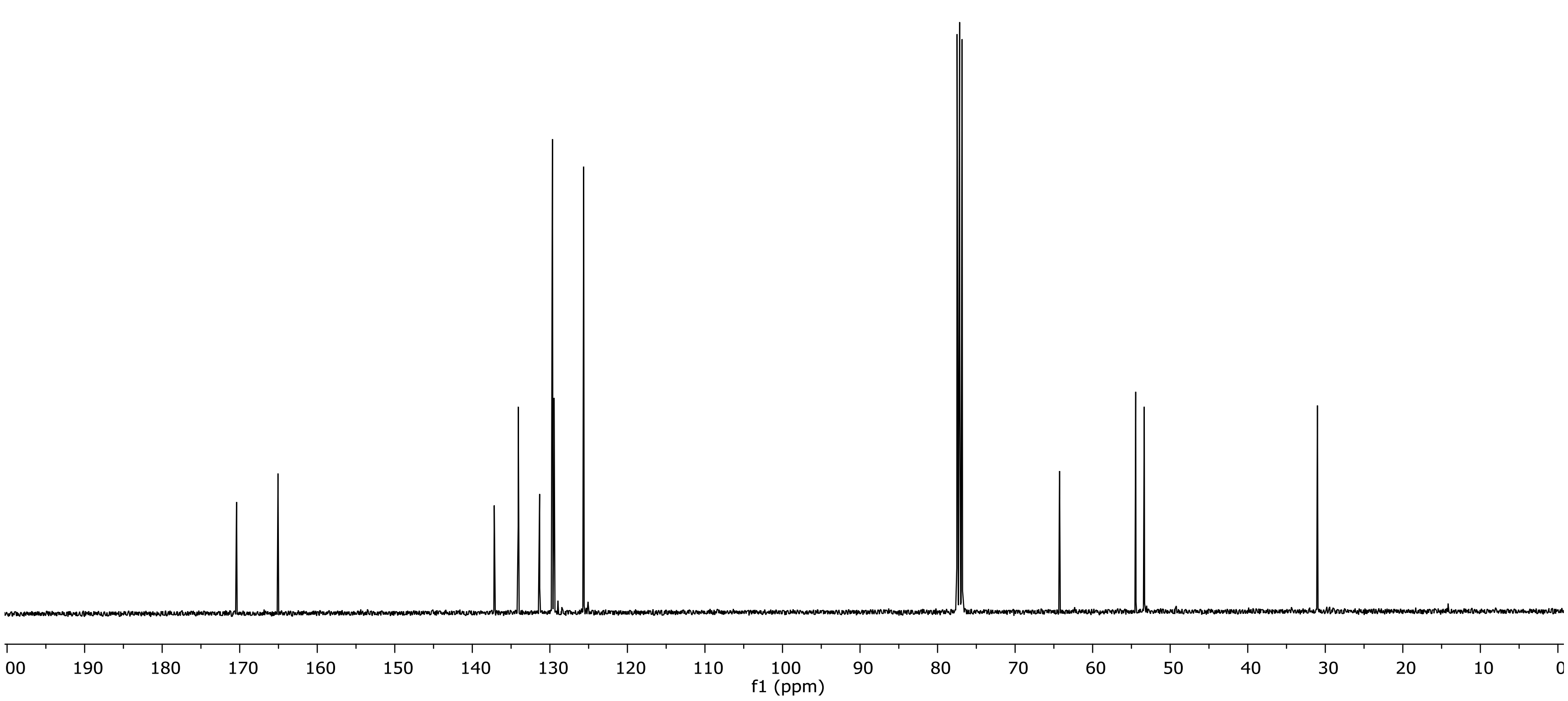
170.4
165.1
137.2
134.1
131.3
129.7
129.5
125.6
64.3
54.4
53.3
31.0
00
190
180
170
160
150
140
130
120
110
100
90
80
70
60
50
40
30
20
10
0
f1 (ppm)

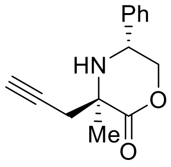
Ph
HN
O
Me
O

S28

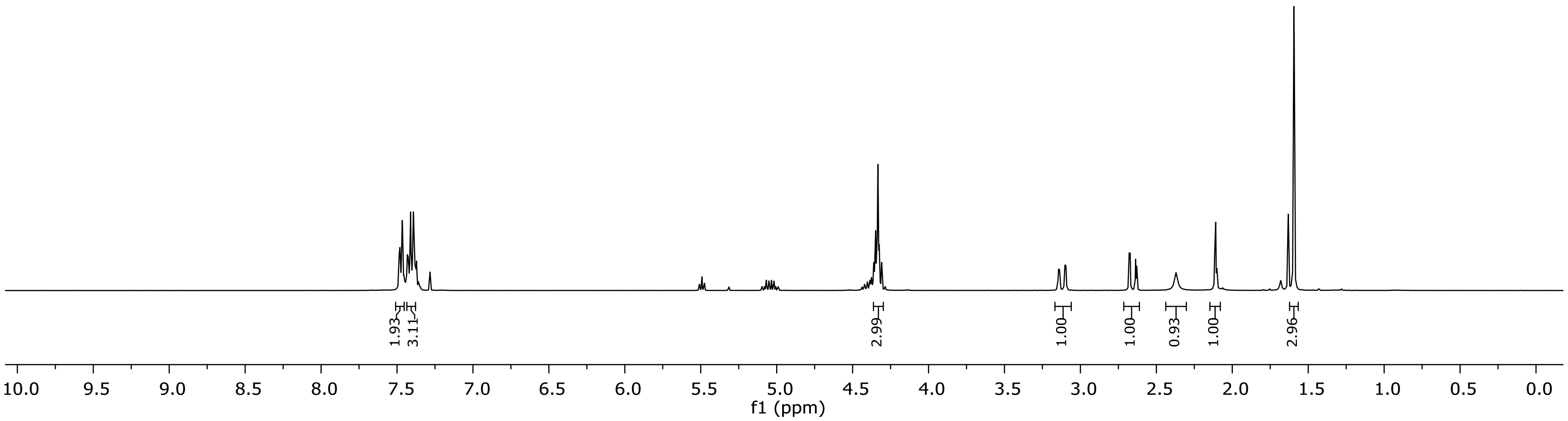
1.93
3.11
2.99
1.00
1.00
0.93
1.00
2.96
10.0 9.5 9.0 8.5 8.0 7.5 7.0 6.5 6.0 5.5 5.0 4.5 4.0 3.5 3.0 2.5 2.0 1.5 1.0 0.5 0.0
f1 (ppm)

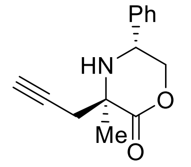

S28

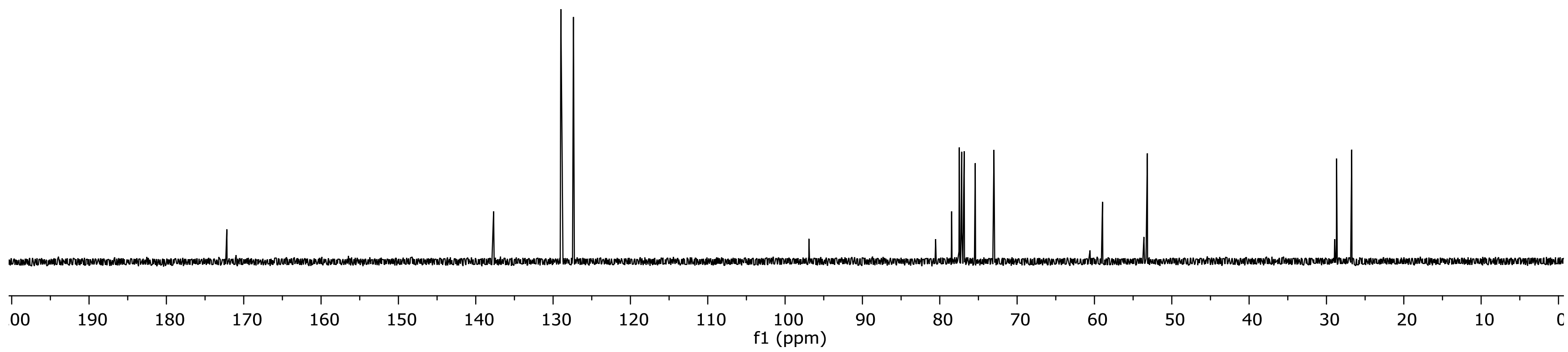

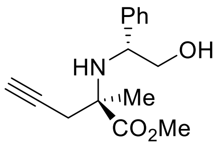
Ph
OH
HN
Me
CO_2Me

S29

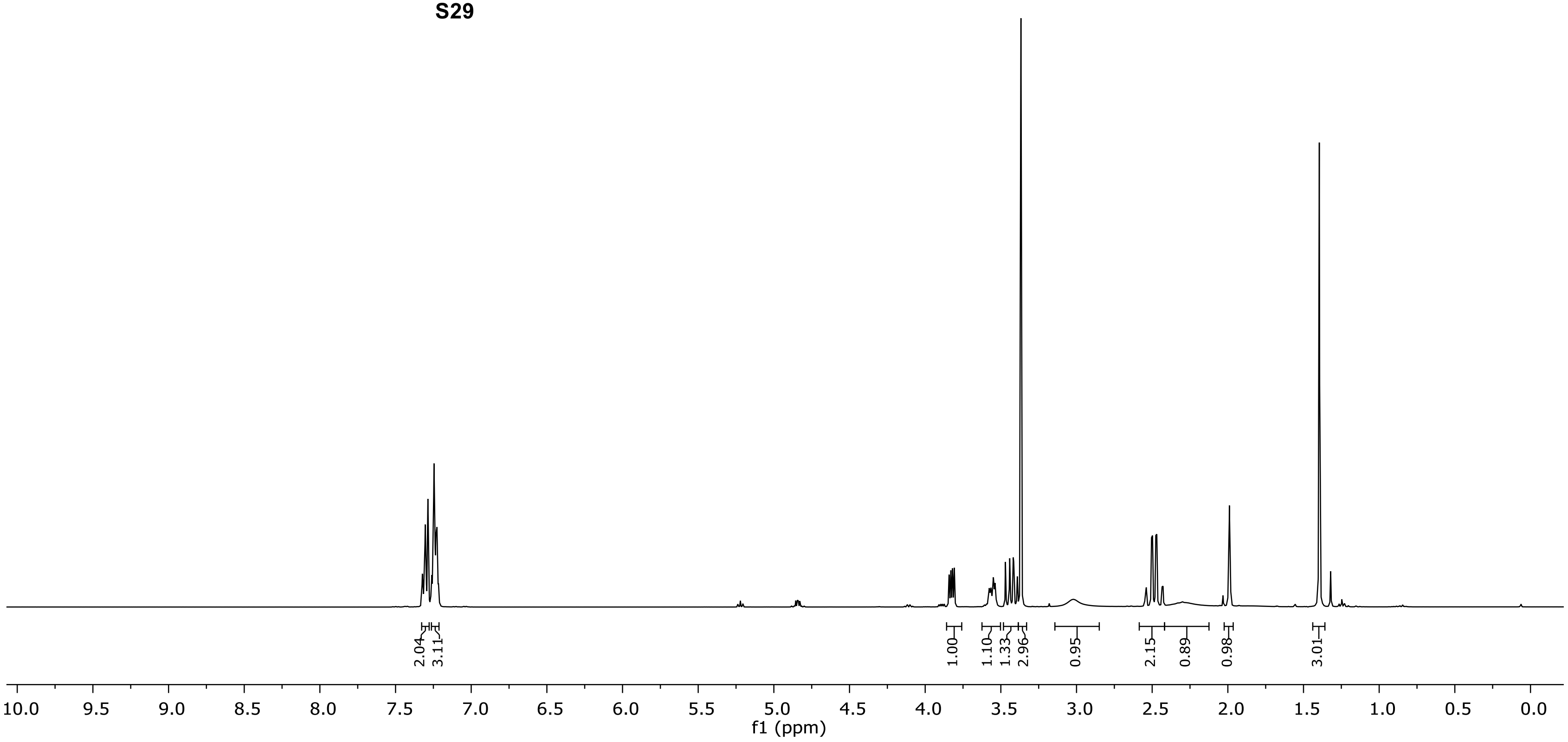
2.04
3.11
1.00
1.10
1.33
2.96
0.95
2.15
0.89
0.98
3.01
10.0
9.5
9.0
8.5
8.0
7.5
7.0
6.5
6.0
5.5
5.0
4.5
4.0
3.5
3.0
2.5
2.0
1.5
1.0
0.5
0.0
f1 (ppm)

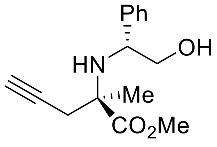
Ph
OH
HN
Me
CO_2Me

S29

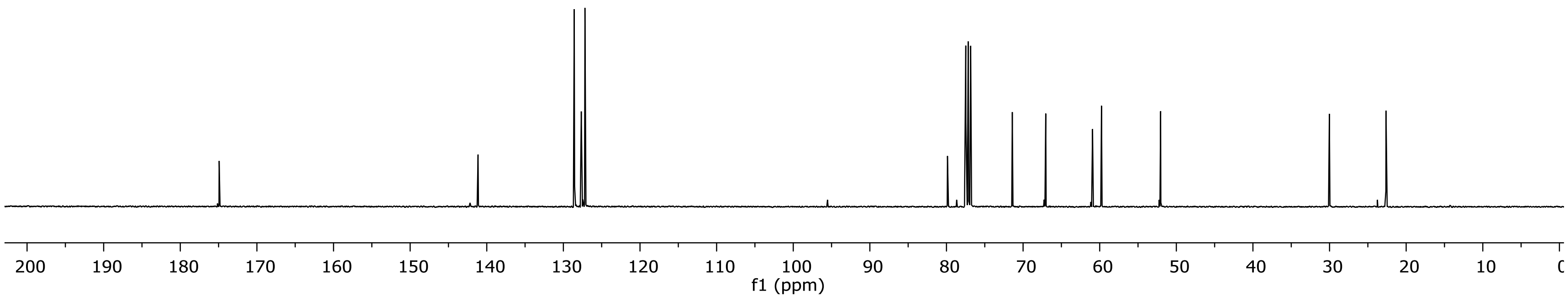
174.9
141.1
128.6
127.7
127.2
79.9
71.4
67.0
60.9
59.8
52.0
30.0
22.6
200 190 180 170 160 150 140 130 120 110 100 90 80 70 60 50 40 30 20 10 0
f1 (ppm)

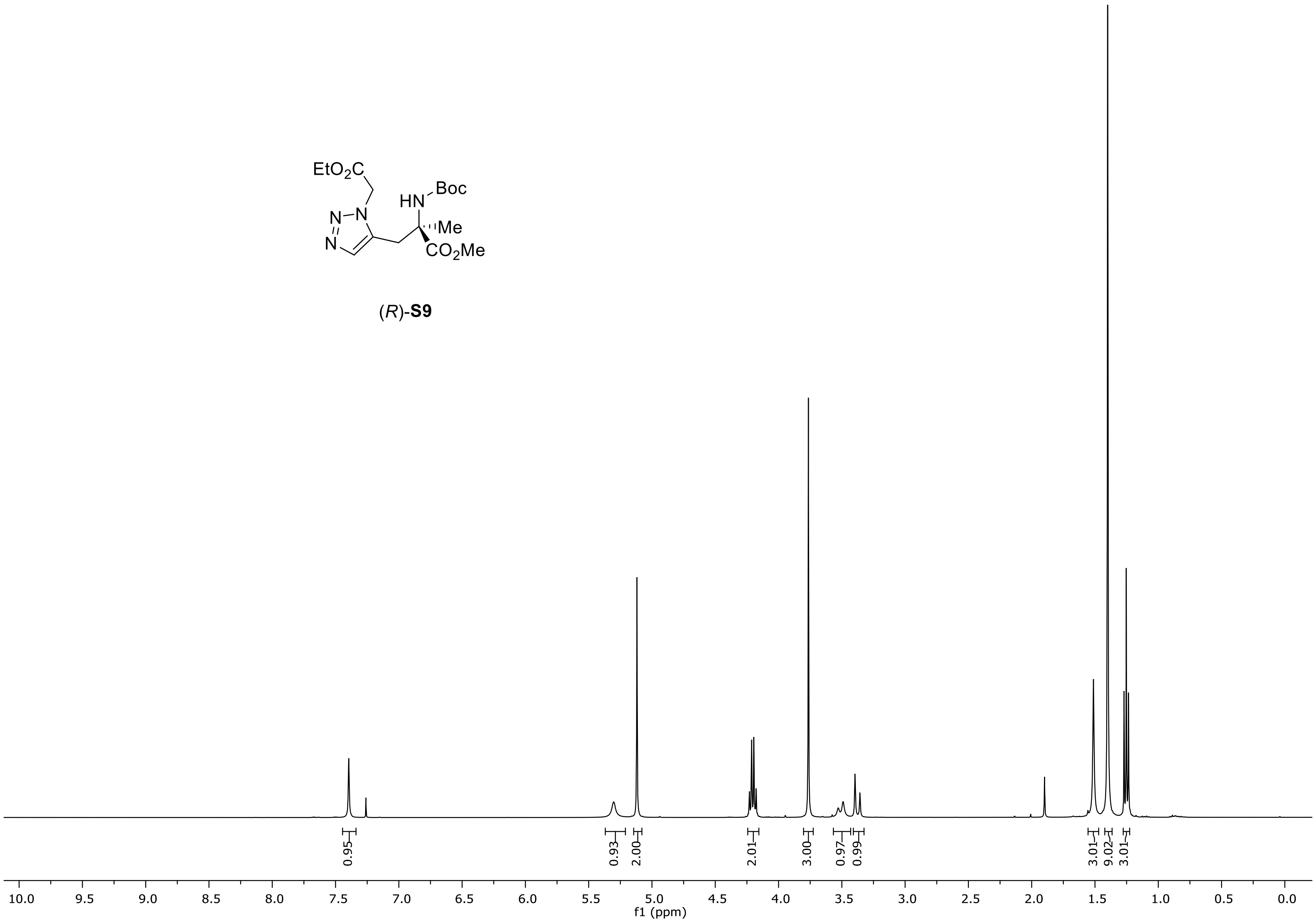
EtO2C
HN
Boc
N
N
N
Me
CO2Me
0.95
0.93
2.00
2.01
3.00
0.97
0.99
3.01
9.02
3.01
10.0
9.5
9.0
8.5
8.0
7.5
7.0
6.5
6.0
5.5
5.0
4.5
4.0
3.5
3.0
2.5
2.0
1.5
1.0
0.5
0.0
f1 (ppm)

(*R*)-**S9**

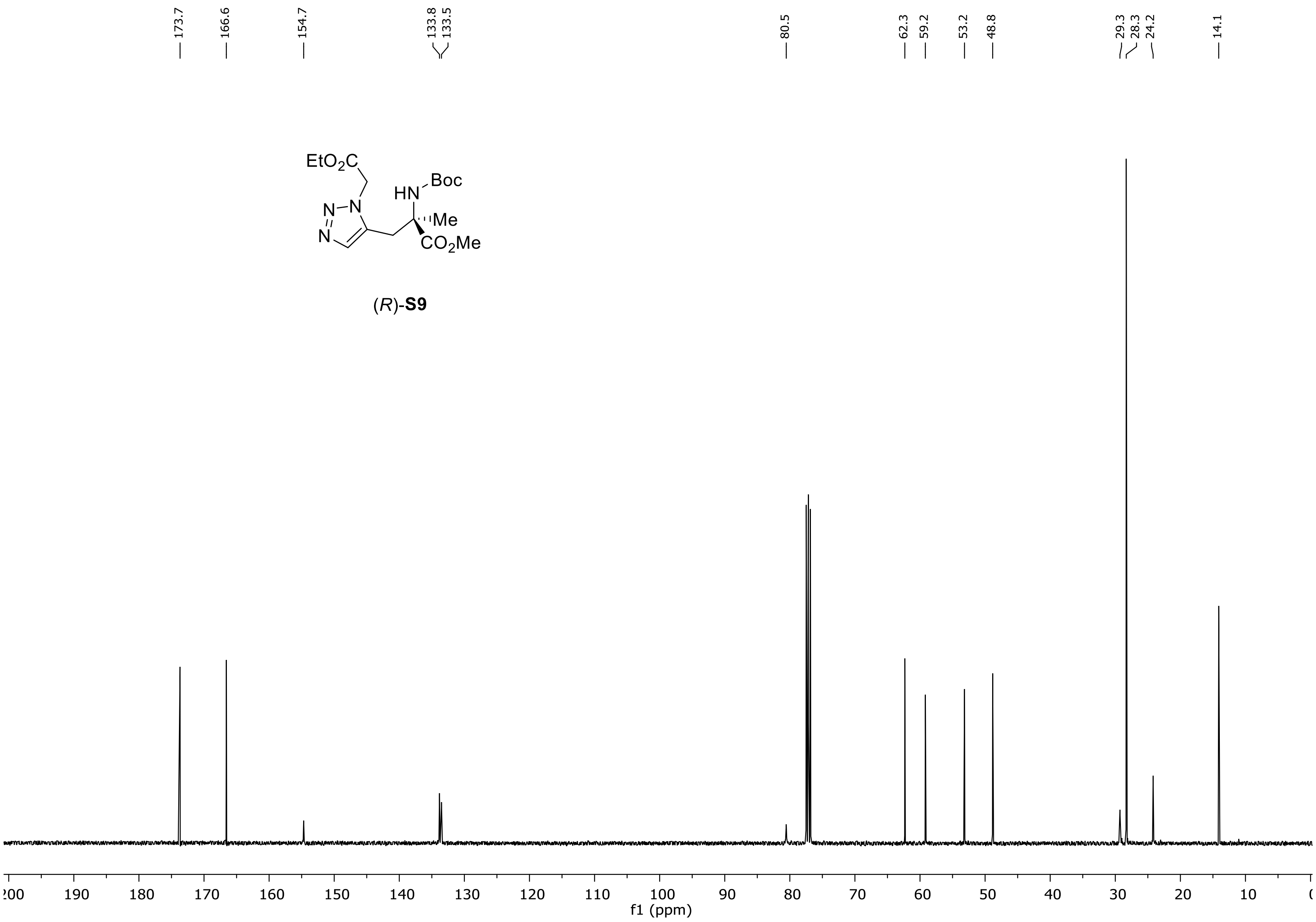
173.7
166.6
154.7
133.8
133.5
80.5
62.3
59.2
53.2
48.8
29.3
28.3
24.2
14.1
EtO2C
HN
Boc
N
N
N
Me
CO2Me
(R)-S9
200
190
180
170
160
150
140
130
120
110
100
90
80
70
60
50
40
30
20
10
0
f1 (ppm)

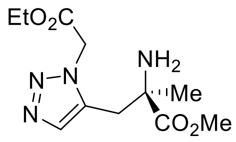
EtO2C
NH2
N
N
N
Me
CO2Me

(*R*)-**8**

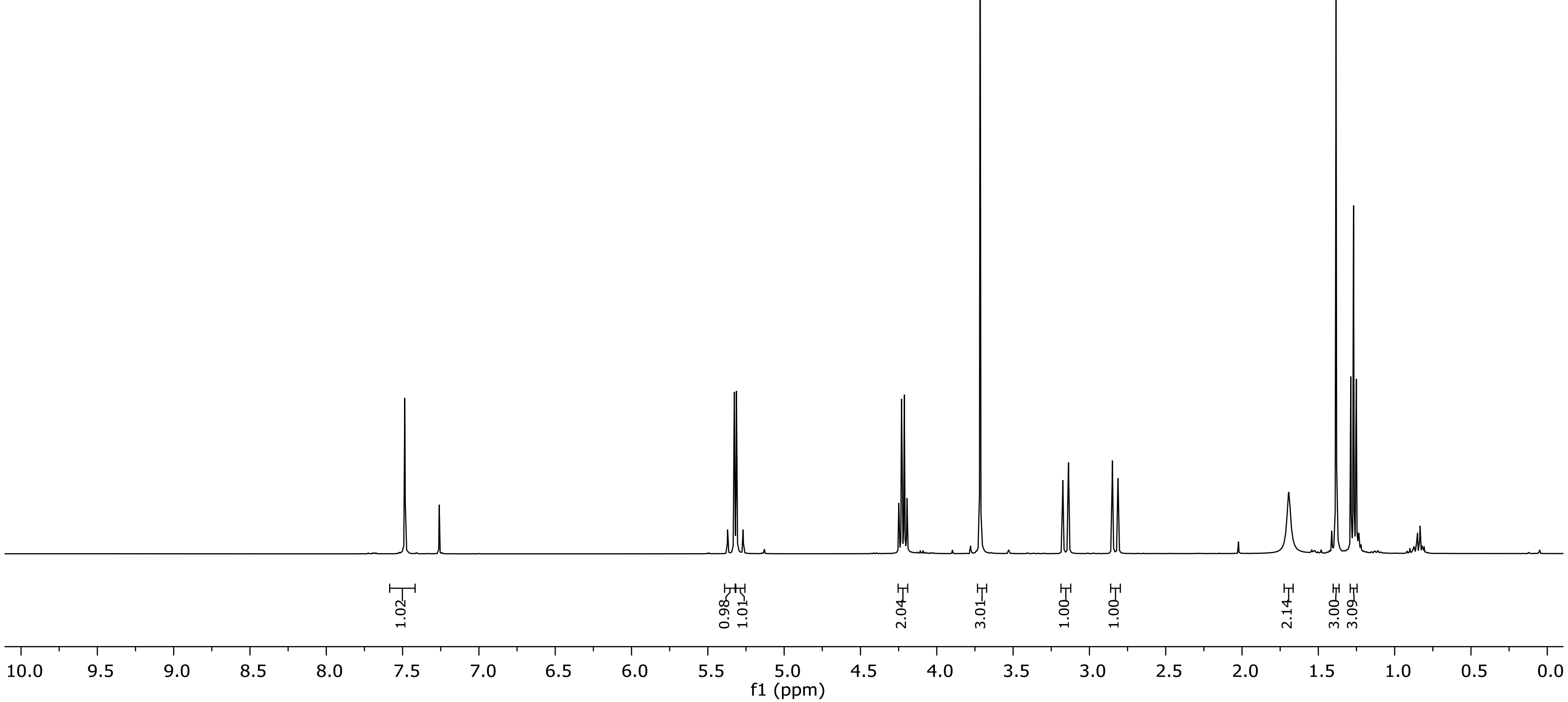
1.02
0.98
1.01
2.04
3.01
1.00
1.00
2.14
3.00
3.09
10.0 9.5 9.0 8.5 8.0 7.5 7.0 6.5 6.0 5.5 5.0 4.5 4.0 3.5 3.0 2.5 2.0 1.5 1.0 0.5 0.0
f1 (ppm)

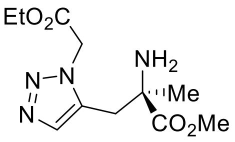
EtO2C
NH2
Me
CO2Me
N
N
N

(*R*)-**8**

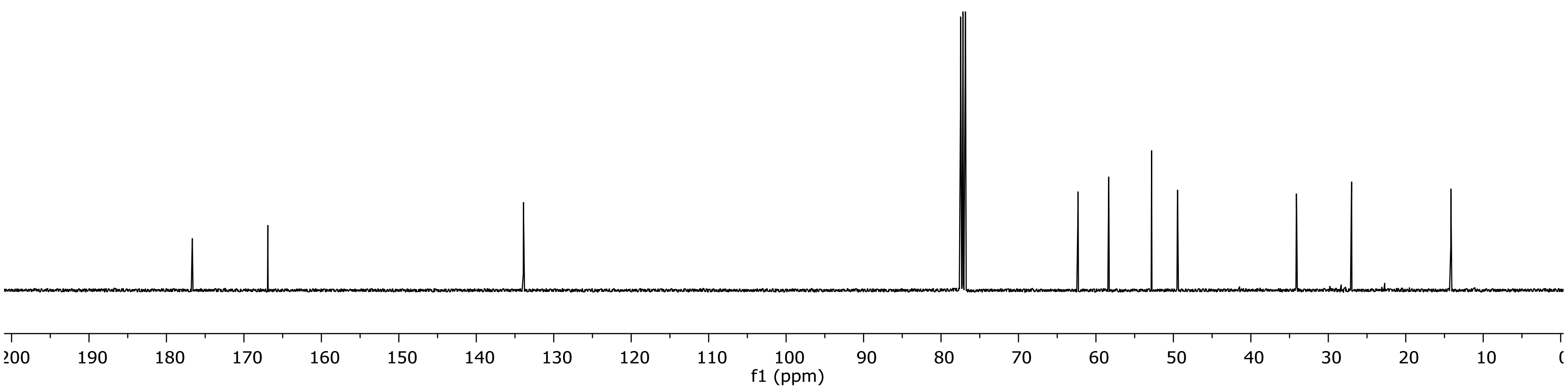
176.7
166.9
133.9
133.9
62.3
58.3
52.8
49.5
34.1
27.0
14.2
200
190
180
170
160
150
140
130
120
110
100
90
80
70
60
50
40
30
20
10
f1 (ppm)

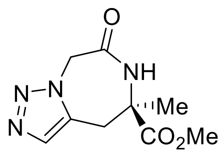
O
NH
N
N
N
Me
CO2Me

(*R*)-**10**

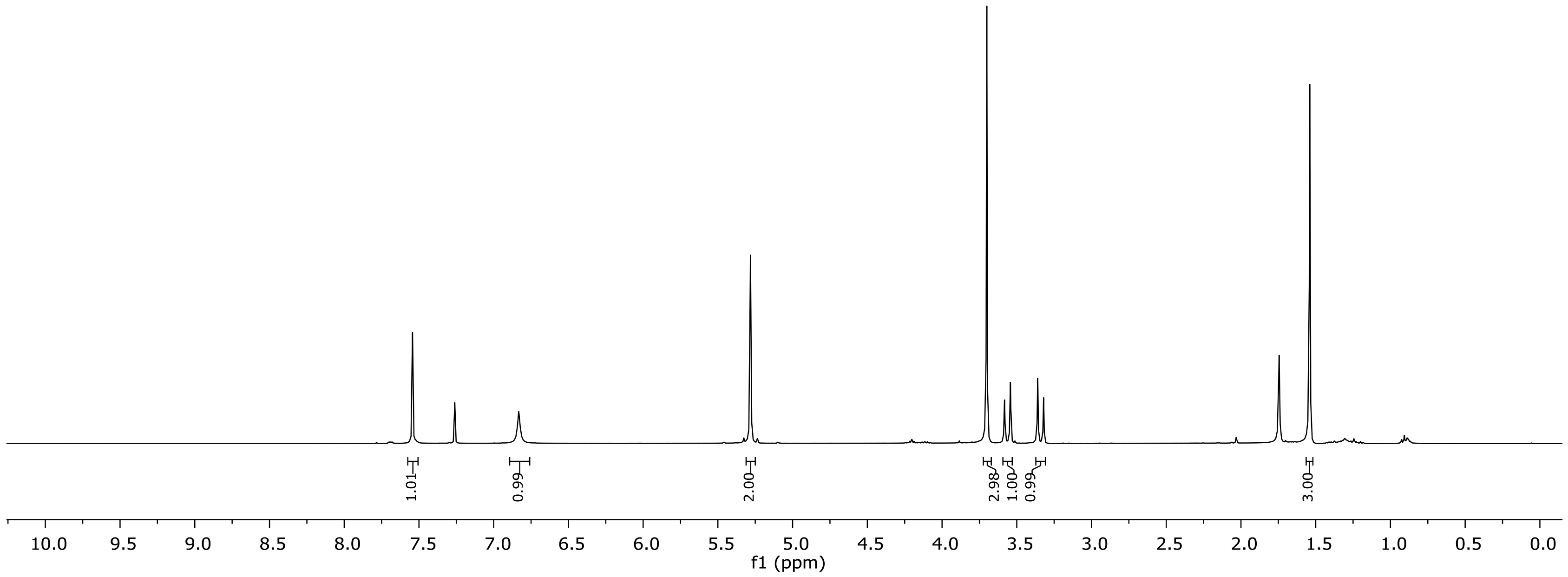
1.01
0.99
2.00
2.98
1.00
0.99
3.00
10.0 9.5 9.0 8.5 8.0 7.5 7.0 6.5 6.0 5.5 5.0 4.5 4.0 3.5 3.0 2.5 2.0 1.5 1.0 0.5 0.0
f1 (ppm)

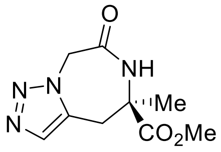

(*R*)-**10**

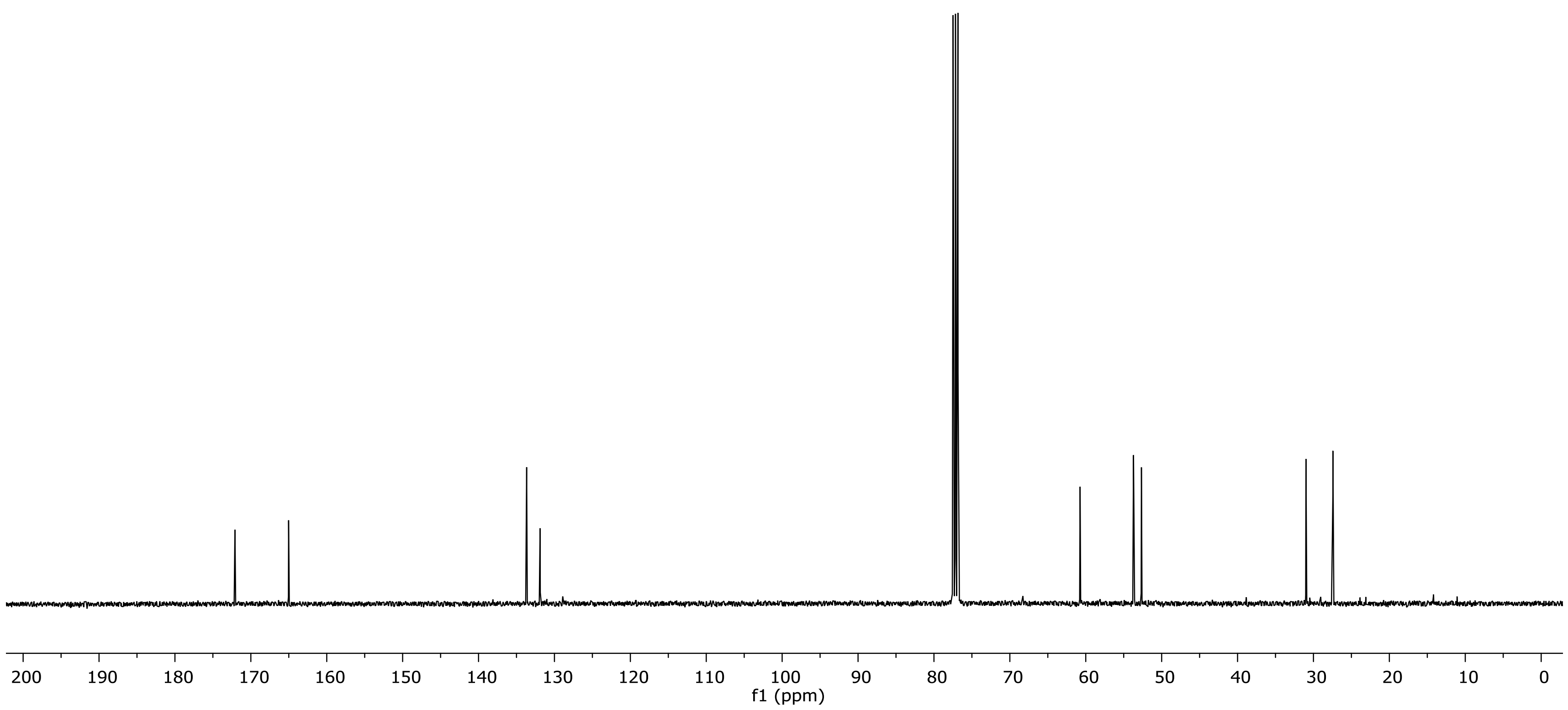

172.1
165.0
133.7
131.9
60.7
53.7
52.7
31.0
27.4
200
190
180
170
160
150
140
130
120
110
100
90
80
70
60
50
40
30
20
10
0
f1 (ppm)
